# Free Papers

**DOI:** 10.1155/1990/72404

**Published:** 1990


**FP001** SURGICAL MANAGEMENT OF HEPATIC CYSTS, POLYCYSTIC LIVER DISEASE & CYSTADENOMAS.

H. SANCHEZ, M. GAGNER, R.L. ROSSI,
R.L. JENKINS, W.D. LEWIS, J.L. MUNSON,
J.W. BRAASCH. DEPARTMENT OF SURGERY,
LAHEY CLINIC & NEW ENGLAND DEACONESS
HOSPITAL, BURLINGTON & BOSTON, MA, USA

Simple hepatic cysts (SHC), polycystic liver disease (PCLD) and cystadenomas (CA) share in common clinical features, difficulties in diagnosis, radiological investigations and surgical management. The surgical management of non-parasitic cystic liver disease (NPCLD) remains controversial. The aim of the study was to compare and assess the surgical management in the 3 groups. We reviewed the records of 42 patients with NPCLD who had 66 surgical procedures over a 25 year period. There were 12 cases of SHC, 11 PCLD and 19 CA. Mean age was 47 years and 88% were females. The most common symptom was chronic abdominal pain in all groups. Polycystic kidney disease was associated with 45% of PCLD. CT and US were diagnostic in 80% for SHC, 100% for PCLD and only 60% for CA. Cyst(s) were right-sided in 83% of SHC and 57% of CA. Disease was bilobar in all PCLD with a right lobe predominance in 18%. Of all solitary cystic lesions in the left lobe, 75% of them were CA.

Surgical Treatments and Recurrences

| | Aspiration | Partial Excision | Excision | Resection | OLTx |
|---|---|---|---|---|---|
| SHC | 1 (100%) | 8 (38%) | 4 (0%) | 1 (0%) | |
| PCLD | 6 (100%) | 7 (57%) | | 2 (0%) | 2 (0%)* |
| CA | 9 (100%) | 8 (88%) | 5 (0%) | 12 (0%) | 1* |
| TOTAL | 16 (100%) | 23 (61%) | 9 (0%) | 15 (0%) | 3 (0%) |

(%) = Percental of Recurrence * = Operative Death

The mean follow up (excluding deaths) for all patients is 33 months. All cysts recurred after aspiration. Partial excision procedures were unsuccessful in the majority of CA and 38% of SHC. Anatomical resection was mostly performed for CA. Total excision or liver resection with the cyst(s) is the treatment of choice for all NPLCD, and partial excision can be of benefit to relieve symptoms in approximately 40% of patients with PCLD.

FP002 **BENIGN TUMORS OF THE LIVER - DIAGNOSTIC PROCEDURE AND INDICATION FOR SURGERY**

Weimann,A., Ringe,B., Barg-Hock,H.,Böker,K*
Gratz,K.F**, Grote,R***, Lamesch,P.,
Roling,A., Pichlmayr,R.
Dpts. of Abdom.- and Transpl. Surg., Gastro-enterol.*, Diagn. Radiol.***,Nucl. Med.**,
Hannover, Medical School.

Between 1982 and 1989 in 204 pts. benign tumors of the liver were diagnosed after presentation in outpatient clinic. These were hemangioma n=129 (M:F=1:2,6), focal nodular hyperplasia (FNH) n=65 (M:F=1:6,6) and adenoma n=9 (M:F=1:8). In 41 pts. (20%) resection of the tumor was performed. In 22 (54%) of these patients diagnosis could only be verified by laparotomy. Related to symptoms and due to observed enlargement of known hemangioma or FNH surgery was performed in 19 (46%) pts.

In case of AFP negativity diagnostic noninvasive workup included the following:

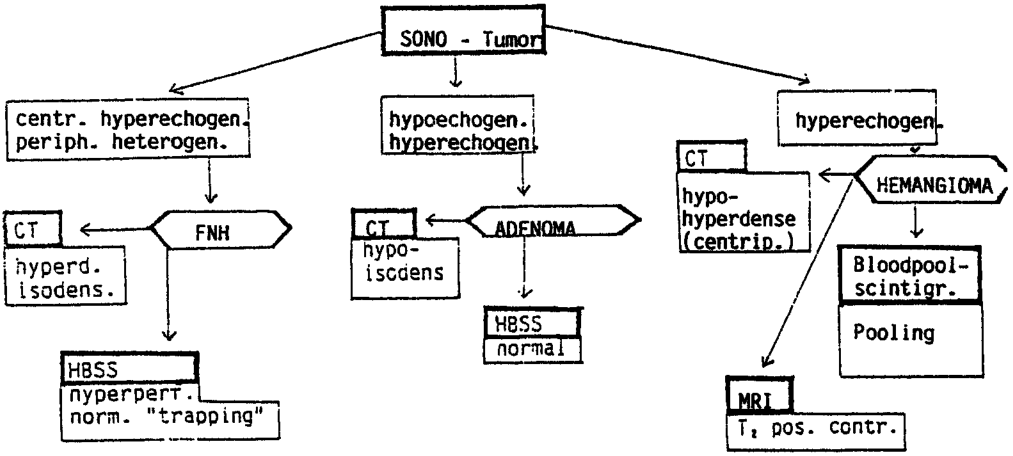

For the risk of hemorrhage fineneedle biopsy or cytology should be avoided. In consideration of the risk for the patient angiography cannot differentiate dignity of the tumor adequately.
Additionally to the results in nuclearmedicine at least CT or sonography should support the diagnosis. Otherwise diagnosis has not been verified. Indications for surgery are: 1. no verified dignity of the tumor, 2. adenoma due to the risk of malignancy and hemorrhage,3. symptomatic hemangioma/FNH with observed enlargement.

FP003

### FOR THE RESECTION OF BENIGN LIVER CELL TUMORS

J. Belghiti, D. Patteron, J.P. Benhamou, F. Fékété

Service de Chirurgie Hôpital Beaujon 92118 Clichy France

Surgical treatment of benign liver cell tumors including focal nodular hyperplasia (F.N.H.) and adenoma is not well established. The aim of this work was to evaluate the results of resection performed from 1984 in 37 patients in whom preoperative established diagnosis was benign liver tumor.

Preoperative assessment including ultrasonography, dynamic computed tomography, technetium sulfure colloid liver scan, and biological tests suggested a precise diagnosis of FNH (n=11) and of adenoma (n=10). In 16 cases no precise diagnosis was established.

Operative procedures were 10 major hepatectomies, 3 trisegmentectomies, 8 bisegmentectomies, 9 segmentectomies and 7 enucleations. There was no postoperative death. Twenty patients had no intra or postoperative transfusion. Minor complications occured in 3 patients. Histologic definitive diagnosis was: FNH in 25; adenoma in 10; in one case areas of hepatocellular carcinoma were demonstrated in an adenoma and in another case a fibrolamellar carcinoma was identified.

In conclusion: (a) precise preoperative diagnosis of benign liver tumors is difficult, a carcinoma can be unrecognized; (b) resection of these tumors can be safely performed in specialized centers. Therefore resection of all so-called benign liver tumors should be performed.

FP004 **CAVERNOUS HEMANGIOMA OF THE LIVER: WHEN TO OPERATE ?**

**S. Karagiulian and E. Galperin**

**1st Moscow Medical Institute, USSR**

Between 1972 and 1989 we operated on 36 patients aged from 17 to 62 years with cavernous hemangioma of the liver. Fifteen of them were hospitalized with already palpable liver or tumour, 27 had complaints and 9 patients were asymptomatic. Resection of the liver was performed in 26 patients, including 10 extensive (more than 50 % of liver mass): right extended hemihepatectomy in 2, right hemihepatectomy in 7 and left hemihepatectomy in 1. Controlled hepatectomy was the method of choice, in four patients a method of finger exposure of hepatic pedicles was used. Exploration only was performed in 10 patients with giant hemangioma or with affection of both liver lobes. Three deaths in the early postoperative period were observed after extensive resections. Without operation or after exploration only 20 patients are under control during 1 - 16 years. We have not observed spontaneous rupture or malignant changes of their cavernous hemangioma. Last years we incline more and more to expectancy with sonographic control in cases with asymptomatic as well as with giant hemangiomas. Only pronounced complaints of the patient or a danger of traumatizing the tumour used to be indications to surgery in our recent series.

**FP005** TREATMENT OF GIANT CAVERNOUS HEMANGIOMAS OF THE LIVER: AN INSTITUTION REPORT

M.Montorsi, J.Spiropoulos, U.Fumagalli, S.Bona, G.Torzilli and G.Pezzuoli
1st Surgical Department
University of Milan, Milan, Italy

In the years 1982-1989 a total of 36 patients with giant liver hemangiomas (diameter > 4 cm) were observed at the First Surgical Department of the University of Milan. There were 25 females and 11 males with a mean age of 52 years (range 23-75). Fourteen patients had symptoms (mean lesion diameter:12.8 cm) while 22 had not (mean lesion diameter:6.8 cm). Tumours were right-sided in 20 cases, left-sided in 7 and bilateral in 9 (three with diffuse angiomatosis). Ultrasonography had an overall accuracy of 56%, confirming the low diagnostic value of this exam for large lesions. CAT scan and MRI carried a 86% and 94% accuracy respectively; in case of multiple angiomas MRI accurately detected more lesions than any other technique. The overall pre-operative diagnostic accuracy was 95%.

Fifteen patients (42%) were submitted to surgical treatment because of severe pain (13), rapid mass enlargement (1) and septic fever after an hepatic artery ligation performed in another hospital (1). Eight major and 7 minor hepatectomies were performed with 6.5% operative mortality and 13% operative morbidity (1 pulmonary embolism and 1 subphrenic fluid collection). All patients are well and asymptomatic after a mean follow-up of 36 months. In the same period 21 patients were not operated on; they all have giant angiomas incidentally discovered by ultrasound. None of these lesions enlarged or became symptomatic after a mean follow-up of 14 months.

In our experience: 1) MRI is the diagnostic procedure of choice for liver hemangiomas; 2) large but asymptomatic lesions can be safely observed without operative treatment; 3) surgery is indicated in symptomatic lesions, when rapid enlargement occurs (2.7% in our series) and, rarely, in diagnostic doubt.

### FP006 SURGICAL TREATMENT IN HEPATIC CAVERNOUS HEMANGIOMAS

G. Văideanu, I. Câmpeanu,

V. Constantinescu, N. Neagu, C. Iurea,

V. Bîtcă. Central Military Hospital,

Bucharest, Romania

The authors report on 5 cases of large hepatic cavernous hemangioma treated in the 1st Section of Surgery of the Central Military Hospital in Bucharest, in the 1985-1989 period.

Subjective symptomatology was scarcely significant in general; the main symptom recorded was related to pain in the right hypochondrium. Objective symptomatology was significant, being evinced in the hepatomegalia or in the tumoral mass of the right hypochondrium.

Pre-operative diagnosis was specified by modern imagistic means, ie echography, scintigraphy, computerized tomography, selective arteriography.

In cases of large hemangiomas covering almost completely a hepatic lobe, adjusted hemihepatectomies were performed as follows: a right hemihepatectomy twice and a left one, once. The excision restricted to bisegmentectomy (segments 5 and 6) was performed in a case of a large hemangioma with lesions of chronic hepatitis. Surgery was contra-indicated in a mean-sized hemangioma localized in segment 7, which forbade a limited excision; likewise, the sacrifice of the uninjured hepatic parenchyme would have been much too great in a larger hepatectomy.

No operative and postoperative mortality was recorded, but the incidence of complications was of 60 per cent.

In the authors' opinion, surgery is indicated in unique hemangiomas extending to the hepatic lobe almost completely and also, in hemangiomas that involve risks of trauma, rapid tumoral growth, persistent epigastric pains and discomfort. Surgery is also indicated on purposes of diagnosis or in marginal sites. Spontaneous ruptures that are scarce, but extremely serious, require surgery of extreme urgency.

### FP007 CHOLECYSTECTOMY AND PREGNANCY.

G.Grunblatt; M.León; D.García.

Hospital M.Perez Carreño, Cirugía III

Caracas, Venezuela.

This series is composed of 25 pregnant women that were cholecystectomized over a period of 5 years, 1983-1987. They were refered to us from the Obstetrics Departament based on their clinical suspicion of biliary tract disease.

Mean age was 26, ranging from 19 to 42 years. 5 patients had a diagnosis of cholelithiasis 1 year before becoming pregnant. 18 patients were similarly diagnosed during their pregnancy. 2 patients had their diagnosis more than one year before their current pregnancy.

The symptomatic presentation was acute with right upper quadrant pain and tender mass. 5 patients presented symptoms during the 1st. trimester, 6 within the 2nd., and 14 during the last trimester. No other pathology was taken into account since they were basically healthy women carrying on a basically normal pregnancy.

After completing diagnostic testing (clinical, ultrasound, and laboratory), we divide the patients in two groups. Group (I) with 12 patients that had inmediate surgery, and group (II) with 13 patients that had medical treatment for 48 hours before surgery. The surgical procedure consisted of a simple cholecystectomy thru a Kocher incision.

We had no operative complications. Among group (II) we did have two abortions on their 2nd. and 3rd. post operative days. Both patients were on the 1st. trimester. 5 patients on the third trimester had to undergo cesarean sections, 3 from group (I), and 2 from group (II). There were no further obstetric complications.

### FP008 CHOLECYSTECTOMY AND COMMON BILE DUCT EXPLORATION IN PATIENTS OVER 75

M.Mariani,F.Baticci,S.Noto,M.Massi and P.Chiapponi

Dept.of Surgery,Ospedale Niguarda,

Milano,ITALY

The authors report the results of a series of 84 patients aged 75 years or more operated on electively for gallstones over a 5years period.
All patients underwent cholecystectomy and intraoperative cholangiography.The incidence of common bile duct exploration was 43 per cent comparable to other series reports(Morrow 1978-Ligidakis 1983-Duron 1985).The high incidence of choledocholithiasis emphasizes the importance of routine cholangiography in agreement with other authors(Houghton 1985).
The overall mortality was 2.4 per cent and the morbility rate was 12 per cent;both were directly related to the age of the patient,jaundice,common duct exploration and preoperative associated diseases.Furthemore,reduced plasma albumin level and increased serum transaminase level,as a consequence of the age of the patient and of chronic biliary lithiasis,seem to be associated with increased postoperative morbility.
The low mortality and morbility suggest that elective biliary surgery is a rather safe procedure even in the aged.

References:

Morrow Arch Surg 1978:113:1149

Ligidakis Surg Gyn and Obst 1983:157:15

Duron Ann Med Interne 1985:136:547

Houghton Br J Surg 1985:72:220

### FP009 CHOLECYSTECTOMY IN HIGHER RISK PATIENTS

S.M. Strasberg, J. Sanabria, and P Clavien.
Dep't of Surgery, Mount Sinai Hosp.
University of Toronto Toronto Canada.

Alternate treatments for cholelithiasis are proposed to benefit patients at higher risk for conventional surgery. The purpose of this study was to evaluate results of standard elective cholecystectomy (EC) in higher risk patients treated in a recent time period. APACHE II was used to rate patients. APACHE II scores 6-9 indicate increased risk since, in patients having elective surgery, a score of >6 equates with a serious medical problem (usually cardiopulmonary) combined with age over 45 or age over 75 alone. Scores >10 are an even higher risk level.

71 patients out of a consecutive group of 644 patients having EC from 1984-1989 had a score >6; in 34 the score was 6-9 (Group 1) and in 37 it was >10. (Group 2). The mean age was 63 years, range 45-88yr. 32% of group 1 and 73% of group 2 patients had non-surgical preoperative evaluations and treatments directed toward optimizing medical status prior to surgery. In the subgroup of patients with cardiac disease, all had reevaluation prior to surgery by a cardiologist, but none was turned down for surgery. 49% of patients went to the ICU postoperatively for a median time of 1 day. Significant complications occurred in 9% of group 1 (3/34) and 28% of group 2 (11/39) patients. Most were cardiopulmonary with by far the commonest being postoperative arrhythmia (n=7). There were no recognized postoperative myocardial infarctions. There was no in-hospital or 30 day mortality in the 71 patients in the increased risk groups (or in the series of 644 patients).

We conclude that standard EC is very safe when intercurrent serious medical problems are identified and the patient is appropriately prepared preoperatively, and when patients with cardiac problems are monitored intensively in the early postoperative period.

FP010

### PRIMARY GALLBLADDER CANCER
### :Review of 130 cases

Chung-Han Lee, Kyoung-Hyun Choi, Sung-Do Lee, Jae-Kwan Seo and Young-Hoon Park. Department of Surgery, Gospel Hospital, Kosin Medical College Busan, Korea.

The prognosis of primary gallbladder cancer (PGC) is very poor but patients with PGC whose tumor are an incidental finding at cholecystectomy done for cholecystitis are more fortunate.
This study is a clinical analysis of our surgical experience with PGC at Busan Gospel Hospital in Korea during 20 years from 1970 to 1989. We have surgically treated 130 PGC patients among 2997 operated biliary diseases. The sex ratio (M:F) was 1:1.2 and mean age was 57 years. According to the TNM system by the manual of AJCC (1987):15 patients (11%) belonged to stage I, 19 patient (15%) stage II, 65 patients (50%) stage III, and 31 patients (24%) stage IV. Accuracy of disgnosis prior to surgery was 35%. Incidental cancer of the gallbladder found after simple cholecystectomy for cholecystitis (n=5) and gallbladder polyp (n=1) were 6 cases. Resection was done in 91 cases and gallstone was found 22 cases (24.2%) among them. Of the radical operations performed, simple cholecystectomies were done in 6 cases, curative cholecystectomies in 28 cases, curative cholecystectomies with wedge hepatic resection in 15 cases, curative cholecystectomies with right hepatic lobectomy in 2 cases and curative cholecystectomy with pancreatoduodenectomy in 1 case. Thirty day-postoperative deaths were 9 cases (7%). The overall 1-, 2-, 3-, and 5-year survival rates were 21%, 14%, 8%, and 6%, respectively. In radically resected patients, these rates were 37%, 27%, 19%, and 14%, respectively. Almost all of the patients with palliative resection died within one year.
Of the 6 incidental cancers of the gallbladder, all cases are still alive and 4 cases are alive over 5 years.
With this study it is suggested that the incidental cancer of the gallbladder found after simple cholecystectomy for presumed benign cholecystopathy has good prognosis even though without subsequent radical reoperation and although with radical operation, PGC advanced beyond stage II has poor prognosis. Incidence of associated gall stone was very low in comparison with western reports.

FP011 THE IMPACT OF SURGICAL RESECTION ON GALLBLADDER CANCER

J. Scheele, R. Stangl, A. Altendorf-Hofmann
Department of Surgery, University Hospital, Erlangen, FRG

Carcinoma of the gallbladder is lately recognized despite improved diagnostic capabilities, and therefore remains a terminal illness. Apart from an incidentally discovered early cancer, the value of aggressive surgery is still controversial.

The records of 140 gallbladder cancer patients seen between 1970 and 1988 were reviewed. Forty-one percent of patients had distant metastases, and 65 percent had regional lymph node involvement. The tumour was removed in 73 patients (52 %), with a potentially curative resection in 30 cases (21 %). Eleven patients underwent drainage procedures, 51 had surgical exploration only, and five did not undergo laparotomy.

Within the curative settings, 16 patients had simple cholecystectomy, accompanied by partial gastrectomy and removal of the common bile duct, respectively, in three cases. The other 14 patients underwent additional hepatic resections combined with a Whipple procedure in two, and a colon resection in three cases. In the group with non-radical tumour removal 13 patients underwent hepatic resection, and eight had resection of colon, stomach, extrahepatic bile ducts, and pancreas, respectively.

Median survival for curative and palliative procedures was 23.2 and 1.6 months, respectively, with no palliative survivor at three years. In contrast, following a potentially curative intervention cumulative five-year survival was 45 percent, and eight of 15 patients operated prior to 1985 lived longer than 60 month. The pathological stage (UICC/AJCC 1987) did not significantly affect prognosis. The 13 patients with stage III/IV tumours had a 50 percent five-year survival compared to 29 percent and 57 percent in stages I and II, respectively. In contrast to the recent experience of others (Ouchi et al. 1987, Donohue et al. 1990), the favourable outcome after aggressive surgery for advanced tumour stages in our series suggests that a more radical approach with enbloc resection of hepatic segments IVb+V (Scheele 1989), and regional lymphadenectomy, may improve results in patients with early cancer.

References:
Ouchi K, Owada Y, Matsuno S, et a.: Surgery 1987; 101: 731-737
Donohue JH, Nagorney DM, Grant CS, et al: Arch Surg (in press)
Scheele J: In Lygidakis NJ, Tytgat GNJ, Thieme 1989: 219-247

### FP012 GALLBLADDER CANCER : RESECTION FOR CURE ?

M C ALDRIDGE, D CASTAING, H BISMUTH
Hepatobiliary Surgery and Liver Transplant Research Unit
South Paris Faculty of Medicine, Hopital Paul Brousse,
94800 Villejuif, FRANCE.

Patients living over 5 years following the diagnosis of gallbladder carcinoma are usually those in whom the cancer was an unexpected histological finding following cholecystectomy for presumed benign disease. Controversy exists as to whether simple cholecystectomy is sufficient or whether these patients should undergo more radical surgery with hepatic resection and dissection of regional lymph nodes. We present our experience with the radical approach.

Between 1964-1988, 137 patients underwent surgical treatment of gallbladder cancer and curative resection (cholecystectomy plus bisegmentectomy (IV/V) and node dissection) was performed in 14 (10%). They comprised 10 women and 4 men of median age 57 yr (range 28-77 yr). In 4 (Group A), resection was performed for cancers known to be invading the liver (Nevin Stage V). In 10 patients (Group B),gallbladder cancer was an unexpected finding following cholecystectomy. These 10 patients underwent routine bisegmentectomy (IV/V) 1 to 3 months later. In Group A, 1 patient had a recurrence at the liver resection margin at 7 months and died, 1 is alive at 1 year with local recurrence and 2 are alive without recurrence at 9 years. In Group B, residual tumour was found in 4 patients at the time of bisegmentectomy (in the hepatectomy specimen in 3 and 1 had peritoneal deposits). All 4 patients developed local recurrence and died between 15 and 24 months following surgery. The remaining 6 patients in Group B without residual tumour are all alive without recurrence at between 15 months and 11 years follow-up. Overall 5-year survival for curative resection was 75%.

Bisegmentectomy (IV/V) and node dissection offer the chance of cure in a selected group of patients and worthwhile palliation in others. The finding of residual hepatic disease, however, inevitably leads to local recurrence.

### FP013 THE ROLE OF MACROPHAGES IN IN VITRO PANCREATIC CARCINOGENESIS

H Bradpiece Royal Postgraduate Medical School
Hammersmith Hospital, London.
J Li, R Malt Massachusetts General Hospital
Boston, USA.

Macrophage derived oxygen radicals have been shown to produce cytotoxic and mutagenic effects in cultured cells, on this basis, inflammatory cells may cause malignant transformation of exocrine pancreatic cells in vitro. An organotypic model was established in which a single cell suspension of hamster pancreas was inoculated onto Gelfoam squares and cultured for seven days. Peritoneal macrophages in a separate group of hamsters were cultured with and without phorbol myristate acetate (PMA), a macrophage activator.

Macrophages and pancreatic cells were co-cultured in three groups comprising activated, non-activated and activated but heat killed macrophages. A control group with no macrophage exposure was similarly studied. Following macrophage exposure, the Gelfoam was digested and the resulting cell suspension was cultured in a soft agarose bilayer. Colony formation in this model is an index of malignant transformation.

Results showed a significant difference in the colony counts between all three macrophage exposed (0.875 - 1.1/100m$^2$) and control (0.088/100mm$^2$) groups $P<0.001$.

The model has shown that inflammatory cells and or their mediators are capable of transforming pancreatic exocrine cells in vitro and that PMA might have a similar effect. These findings may have relevance for pancreatic carcinogenesis in vivo as a sequel to inflammation and or environmental toxin exposure.

FP014 **GLUTAMINE MEDIATED REGULATION OF PANCREATIC HORMONE SECRETION**

E.C. Opara, W. Burch, I.L. Taylor, O.E. Akwari
Duke University Medical Center, Durham, NC, USA

*It is well known that the amino acid glutamine is metabolically important for gut function (Souba 1988). Although it has also been shown to be a major fuel for pancreatic islets under physiologic conditions (Malaise, Sener and Carpinelli 1980), the effect of glutamine on the secretion of pancreatic hormones is not clear. AIM: In this study our goal, therefore, was to evaluate the direct effect of l-glutamine on insulin and glucagon secretion by isolated perifused islets. METHOD: In each experiment two islets were isolated by microdissection from each of three mice, and pooled into a plastic perifusion chamber. The islets were preperifused at the rate of 1ml/min for 1 hour at 37°C with Krebs-Ringer-Bicarbonate buffer pH 7.4, containing 5.5mM glucose (basal), 2% bovine albumin and 100 KIU/ml trasylol, that was gassed continuously with 95%/5%, $O_2/CO_2$ mixture. Basal samples were then collected at 2 mins intervals on ice for 20 minutes before l-glutamine was added at increasing concentrations (2mM, 5mM, 10mM, 20mM). The perifusion was conducted for 20 minutes at each concentration and solutions were changed using a stopcock. Effluent samples were also collected and stored frozen until radioimmunoassay for insulin and glucagon. RESULT: L-Glutamine suppressed the secretion of insulin as the mean integrated area under the curve/20 minutes (AUC/20mins) for insulin output decreased from a basal 3673.6±424.7pg to 2361.3±44.3pg, 1699.0±167.8pg, 1721±208.4pg, 1410.6±155.1pg, respectively n=4, for the above given glutamine concentrations. In contrast, glucagon output was stimulated by the addition of l-glutamine. The mean basal glucagon secretion assessed as AUC/20 mins was 508.3±471 pg and increased in a dose-dependent manner up to a mean increment of 1011.7±72.9pg ($p<0.05$) above basal at 20mM glutamine. CONCLUSION: L-Glutamine is an important regulator of endocrine pancreatic function since it suppresses insulin but stimulates glucagon output under basal glucose conditions.*

*References:*
*Souba WW. The gut as a nitrogen processing organ in the metabolic response to critical illness. Nutr Support Services 8:15-22, 1988.*
*Malaise WJ, Sener A, Carpinelli AR, et al. The stimulus-secretion coupling of glucose induced insulin release: physiological role of l-glutamine as a fuel for pancreatic islets. Molecular and Cellular Endocrinology, 20:171-189, 1980.*

### FP015 STIMULATORY ACTION OF DEOXYCHOLATE ON ISOLATED PANCREATIC ACINI

Y. Takeyama, H. Nakanishi, K. Kaneda,
H. Ohyanagi, Y. Saitoh, Y. Takai
Kobe University School of Medicene,
Kobe, JAPAN

A preceding report from our laboratories (Takeyama et al. 1986) has described a stimulatory action of bile acids on the exocrine pancreas, which is presumably involved in the development of biliary pancreatitis. Deoxycholate (DCA), a secondary bile acid, sensitize the isolated rat pancreatic acini and enhance the cholecystokinin-octapeptide ($CCK_8$)-induced phospholipase C (PLC) reaction, and thereby potentiate amylase release. DCA does not affect the binding of $CCK_8$ to its receptors on acini, and does not modulate the secretory process subsequent to the protein kinase C activation and the intracellular $Ca^{2+}$ mobilization.

Recently, accumulating evidence suggests that a GTP-binding protein(s) (G protein(s)) is involved in the coupling of receptors to PLC in the exocrine pancreas. In this paper, the mode of this action of DCA was investigated using Sodium Fluoride (NaF), which is a direct activator of GTP-binding proteins.

NaF alone induced PLC reaction and amylase release in the pancreatic acini as obserbed with $CCK_8$. DCA also enhanced the NaF-induced PLC reaction and amylase release. These stimulatory effects of DCA on the NaF-induced reactions of pancreatic acini were comparable to those on the secretagogue-induced reactions reported previously. These results suggested that DCA acts on the coupling of G protein(s) to PLC in the membrane transduction mechanism in isolated rat pancreatic acini.

References

Takeyama Y, Nakanishi H, Ohyanagi H, Saitoh Y, Takai Y. J Clin Invest 1986: 78; 1604.

### FP016 RATE OF LIQUEFACTION OF NECROTIC RETROPERITONEAL TISSUE IN ACUTE PANCREATITIS

J. Howard
Medical College of Ohio, Toledo, Ohio, USA

Patients with massive tissue necrosis resulting from acute pancreatitis have been managed conservatively by the author, delaying laparotomy until operation appeared essential to continued improvement. Operation consisted of debridement and external drainage of the necrotic tissues. It has been determined that in most patients, when massive amounts of necrotic retroperitoneal tissue develop, debridement is technically difficult and incomplete. It would technically be easier and more complete if the necrotic tissue were completely liquified at the time of operation. How long does complete liquefaction require? Observtions have been made at the time of laparotomy and by CT scanning to delineate the period required for liquefaction to occur.

Thirty-four patients have been studied, operative observations being made between 3 weeks and one year after onset of the pancreatitis.

The surprising observation has been the prolonged period during which retroperitoneal necrotic fat may lie dormant with minimal or incomplete liquefaction. Exploration of two patients, as late as 11-12 months after the attack and 9-10 months after clinical recovery, revealed large deposits of retroperitoneal dead tissue. Several patients with the longest periods of observation revealed no evidence of continuing (long-term) toxicity. In all patients liquefaction was incomplete at the time of laparotomy.

It is proposed that the necrotic retroperitoneal tissue, predominantly adipose tissue, has limited exposure to the capillary circulation, thus liquifying quite slowly over a period of undetermined duration.

### FPO17 THE EFFECT OF DIETARY STEARIC AND OLEIC ACIDS IN PANCREATIC NEOPLASIA

D E Khoo, *B Flaks, C B Wood, R C N Williamson, N A Habib

Several studies have shown that high fat diets may promote cancer. We studied the effect of diets containing either 20% stearic acid (SA) or 20% oleic acid (OA) on the development of L-azaserine (AZA)-induced preneoplastic atypical acinar cell nodules (AACN) in the rat pancreas. Leeds strain rats (10 per group) were randomised to the following groups: untreated control group, SA only, OA only, AZA only, AZA + SA and AZA + OA. The animals were killed at 26 weeks and several indices of pancreatic AACN were determined. The Kruskal-Wallis test was used to analyse the data.

OA significantly increased all AACN indices (No of AACN/$mm^3$: OA=2.79±0.95* vs control=0.44±0.44* ($p<0.001$), No of AACN/pancreas: OA=3.18±1.02* vs control0.55±0.55* ($p<0.001$), Vol AACN as %pancreas: OA=1.95±0.53* vs control 0.93±0.71* ($p<0.003$))but there were no such changes in the AZA + SA, AZA, SA or OA only group compared with control. This is the first report describing the effect of these dietary fatty acids in pancreatic carcinogenesis, and it demonstrates that oleic acid promoted AACN whereas stearic acid had no such effect.

*(Mean±sem)

### FP018 THE IMMUNE FUNCTION OF RED CELLS IN PATIENTS WITH PANCREATIC CARCINOMA

L. Rui, G. Feng, T. Yan, W. Ben-mao,
W Men-cao. Dept of General Surgery,
Chang Hai Hospital, Shanghai,
People's Republic of China

Evidence that indicate red cells have both an immune and respiratory function has been obtained recently. Immune adherence of red cells and erythrocyte enhancement of phagocytosis of tumor cells by neutrophils and lymphocytes have also been observed. (1,2,3)

This study showed that there were significant changes of red cell immune function in patients with pancreatic carcinoma. Standard tests for C3b receptor, inhibition of C3b receptor, immune complex and adherence of red cells rosette to tumor cell were performed in 3 groups of patients, respectively: 20 patients with pancreatic carcinoma (group A), 20 patients with benign pancreatic diseases (group B) and another 20 normal adult (group C). Erythrocyte enhancement of tumor cell phagocytosis by neutrophils and lymphocytes was also studied. Immune functions of group A were significantly reduced as compared with that of group B and group C. Furthermore, it was also noticed that immune function of red cells changed significantly after operative intervention.

These observations suggested that human erythrocytes have played an important role in the human immune system. Both the release of immune complex and adherence of tumor cells by red cells could be inhibited by pancreatic tumor and operative intervention. The inhibition and enhancement mechanism of red cell immune function in this study had also been investigated.

References:
1. Siegel I. et al. Lancet 1981;2(8246):556
2. Guo F. et al. Zhong Hua Yi Xue Za Zhi 1982;12:715
3. Forslid J. et al. Immunology 1985;55:97

### FP019 MESH WRAP FOR HEPATIC HEMOSTASIS IN SWINE

S Stevens, J Meadors, T Lewis,
F Hopkins, L Elkins, K Maull
University of Tennessee
Medical Center at Knoxville, USA

Eight miniature swine were submitted to midline laparotomy. Uniform stellate hepatic injuries were made in the dome of the right and left hepatic lobes. The lacerations were crossed at right angles, 3cm deep and 8cm long. The animals were randomly divided into two groups. Group A, control animals, (N=4): stellate liver lacerations without mesh wrap or other measures for hemostasis, and Group B (N=4): absorbable mesh wrap applied for hepatic hemostasis. Except for mesh application, all variables including anesthetic, intravenous fluids and medications were held constant for both groups. The mean preoperative hematocrit (HCT) was 28$\pm$1.5 and 29$\pm$1.5 for the control and mesh groups, respectively. Preoperative liver function values (LFT) were normal in both groups. All animals in the control group died. Mean elapsed time from injury to death was 65 mins. (range; 20-120 mins.). All animals in the mesh wrap group survived (p=0.029). Mean values for total bilirubin, alkaline phosphatase and HCT were 0.45$\pm$0.4, 95$\pm$61 and 32$\pm$8 on postoperative day one and 0.1$\pm$0.05, 60$\pm$14 and 36$\pm$1 on postoperative day seven. At one week postoperatively, the livers were harvested for gross and microscopic examinations. Although adhesions to the mesh were encountered, no abscess, bile leak or hematoma was noted. Histologic evaluation revealed a vigorous foreign body reaction to the mesh and no evidence of ischemic, thrombotic or obstructive damage to the liver remote from the injury.

In conclusion, the hepatic mesh wrap: 1) is geometrically, technically and mechanically feasible, 2) was not associated with thrombotic, obstructive or infectious complications in this series, and 3) can effectively secure hemostasis following liver injury.

**FP020** CONSERVATIVE TREATMENT OF TRAUMATIC SPLENIC INJURY

Man-yuan Xu
The Hospital of Xin Zhou Prefecture
Xin Zhou City
The People's Republic of China

Since February 1980, we have treated 52 cases of traumatic splenic injury conservatively, the diagnosis was confirmed by peritoneal puncture, ultrasonography and scintigraphy.

The conservative therapy included strict bed rest, fast, hemostasis, expanding blood volume, using antibiotics etc. After the status of patients was stable, the patients began to administer the Chinese herbal medicine orally. The prescription is 'Ge Xia Xhuo Yu Tang'.

Through this therapy, the injured spleens recovered completely, which was confirmed by ultrasonography and scintigraphy. There were no deaths and no complications. The patients could take part in ordinary work and study.

This study shows that this prescription has the effect of producing hemostasis, absorbing hemoperitoneum, reducing intestinal adhesion, promoting peristalsis and shortening the course of the disease.

The Chinese herbal medicine non operative treatment not only has excellent results, but also avoids the suffering and danger of patients' lives during operation and preserves the function of the injured spleen.

**FP021** **MODIFICATION OF BURHENNE METHOD IMPROVES SUCCESS RATE FOR REMOVAL OF RETAINED BILIARY CALCULI**

**D. McCrory, E. McIlrath, B.J. Rowlands,**
**Department of Surgery,**
**The Queen's University of Belfast, UK.**

Burhenne extraction of retained calculi following surgical exploration of the biliary tree has been employed for many years. Stone extraction from cystic duct remnant (CDR) or curved mid portion of the common bile duct (CBD) is the most difficult. If the T tube track is too tight, tortuous, or has too acute an angle of entry into the CBD the Burhenne instrument can not be manipulated.

Our modification to overcome these problems uses balloon catheters with preformed curvatures. The curve on the catheter allows its manipulation into all areas of the biliary tree. Stones are pushed ahead of the balloon into the duodenum. If the stone will not pass the ampulla is dilated with an angioplasty balloon.

35 patients over 26 months were referred for Burhenne procedure. 43 stones were removed. 33 from CBD, 3 from left hepatic duct, 3 from right hepatic duct, 3 from common hepatic duct and 1 from CDR. 17 were extracted using classical Burhenne technique: 21 pushed into the duodenum using our modification: 5 crushed and removed.

32 patients (91.4%) had removal of all stones after 1 or 2 sessions. 3 had ERCP and sphincterotomy. 3 developed minor complications; 1 had a single rigor, 1 had an elevated amylase for two days and 1 had a false track created. There was no mortality and no surgical intervention. Mean hospital stay was 2.05 days.

Conclusions:
This is a safe technique with low morbidity and no mortality which allows the removal of all stones, some of which were previously inaccessible to Burhenne extraction.

FP022 PANCREATIC ABSCESS

R.N. Katariya, S.K. Khanna, N.M. Gupta,
S.M. Bose, J.D. Wig, S. Suri
Departments of Surgery & Radiology
P.G.I.M.E.R, Chandigarh, India

Pancreatic abscess though rare is one of the major complications of acute pancreatitis and remains a highly lethal complication.

We report our experience of 37 patients (26 men, 11 women, age range 17-30 years) with pancreatic abscess seen over the last 8 years. Patients with infected peripancreatic fluid collections in association with significant tissue necrosis were studied. Gall stones and alcohol were responsible in 30 patients. 35% of patients presented with acid-base abnormalities. Plain X-ray abdomen revealed gas bubbles in pancreatic area in 3 patients. Contrast studies (n=14) showed displacement of stomach,colon, or fistulization. CT findings (n=21) noted were loss of normal gland contours, obliteration of surrounding soft tissue planes, gas bubbles in pancreatic bed or gas spaces within the fluid collections. Preoperative percutaneous catheter drainage of abscesses was performed in 9 patients. Surgery was performed in 36 patients (transperitoneal route n=30; extra-peritoneal route n=6). Extensive debridement and external drainage were the mainstays of therapy. 7 patients were treated with open packing. Two patient had colonic involvement in the form of colopancreatic fistula and gangrene of the colon. Postoperative complications were frequent. 20 patients (53%) died. The cause of death were overwhelming sepsis, multiple organ failure, and haemorrhage.

### FP023 PERITONEOVENOUS SHUNTING (PVS) IN CIRRHOTIC AND MALIGNANT ASCITES

EF Foley, WD Lewis, R Rossi, and RL Jenkins, New England Deaconess Hospital and Lahey Clinic Medical Center, Boston, MA, USA

Although aggressive diuretic management remains the cornerstone of therapy for both malignant and cirrhotic ascites, PVS has evolved as an effective palliative procedure for the management of intractable ascites. We have reviewed our PVS experience in 103 patients (53 cirrhotic, 50 malignant) undergoing 160 shunt procedures since 1979. The clinical courses of cirrhotic and cancer patients undergoing PVS were different, cirrhotic patients enjoying lower operative mortality (9.4% vs. 24.0%, $p<.05$) and longer survival (one year- 45% vs. 8%, $p<.001$), despite more complications per procedure ($1.6 \pm 0.1$ vs. $0.7 \pm 0.1$, $p<.001$), longer postoperative hospital stays, and more frequent shunt revisions. Approximately 50% of all patients maintained long term ascites palliation. Advanced age, the presence of cancer, and preoperative bilirubin elevation all independently predicted poorer patient survival. Because of the overall high mortality and morbidity associated with this procedure, we currently reserve PVS for patients with incapacitating ascites who have failed aggressive medical therapy and have exhausted all other therapeutic options, including transplantation in appropriate candidates.

### FP024 LE VEEN PERITONEOVENOUS SHUNT

S Vassilev, S Baev. Institute of
Surgery, Sofia, Bulgaria

For the last 15 years a new surgical procedure has been applied for ascites (Le Veen et al. 1974). During the period 1987-1989, 19 patients with liver cirrhosis, complicated by refractory ascites received a Le Veen peritoneovenous shunt.

Good immediate results, demonstrated by change of the abdominal girth (18cm mean decrease), decreased body weight (11 kg mean change) and increased urine flow were observed in 14 cases. There was no response in 2 cases. Intravascular disseminated coagulopathy has been reported recently in connection with this shunt (Tawes et al. 1981). Clinical features of this state were observed 4 times. There were 4 deaths, forming postoperative mortality of 21.1%. The follow-up period of the surviving patients is from 2 to 30 months.

Analysis of the causes of the mortality brought to a revision of the indications for this procedure. In order to improve the immediate and long term results, the shunt should be considered in patients even before they prove to be refractory to diuretic therapy.

References:

Le Veen H., et al., Ann Surg. 1974; 180:580
Tawes R L, et al., Am. J. Surg., 1981; 142:51

### FP025 EXPERIMENTAL TRANSPLANTATION OF BIOARTIFICIAL PANCREAS; IMMUNOISOLATION OF ISLETS USING MESH - REINFORCED POLYVINYL ALCOHOL TUBE

K.Inoue, S.Sumi, Gu Y-J, S.Fujisato*, S.Higashide, H.Minote, K.Takaori, M.Yun, K.Uchida, T.Tobe, Y.Tabata*, Y.Ikada*
First Department of Surgery, Faculty of Medicine, *Research Center for Medical Polymers and Biomaterials, Kyoto University, Japan

Although islet transplantation has lots of advantages, all clinical attempts with islets allografts have been unsuccessful probably because of the recurrence of the original disease in the unprotected islet cells. In order to overcome this problem, the present study was undertaken to perform experimental transplantation of islets that are isolated from the immune system by means of the unique biomaterial tube.

Methods: Islets were isolated from the pancreas of male S-D rats (220-260g) by a collagenase digestion method. About 2000 isolated islets obtained from 3 rats were enclosed into the mesh-reinforced polyvinyl alcohol tube. This bioartificial pancreas was transplanted into the peritoneal cavity of male Wister diabetic rats (200-240g), induced by the intravenous injection of 50mg/kg of streptozotocin (n=4).

Results: In one case, the nonfasting blood glucose levels dropped from 436 to 156 mg/dl within two days after transplantation. The longest normoglycemic period in these rats was 60 days [at present, still remains normoglycemic (160±38mg/dl; M±SD)], whereas the normoglycemic period in the control group was less than 7 days.

Conclusions: The present study leads us to speculate that this new bioartificial pancreas could be a promising therapeutic device for diabetes mellitus, since this is xenogenically applicable to humans as well as there is no need for any kind of immunosuppression.

Supported by a grant from the Ministry of Education, Japan (#A 61440060)

### FP026 MODIFIED PLASMA PROTEIN FRACTION (MPPF) VS SUPPLEMENTED MODIFIED PPF (SMPPF) FOR THE 24 HR COLD STORAGE OF THE HUMAN PANCREAS PRIOR TO ISLET ISOLATION

N.J.M. London, S.P. Lake, *M. Slapak,
*M.H. Wise, P.R.F. Bell, R.F.L. James,
Department of Surgery, Leicester Royal
Infirmary, * Portsmouth, UK

We have compared in situ hyperosmolar citrate (HOC) perfusion alone (7 'local' pancreata, cold ischaemia 3 (0.75-3) hrs) with in situ HOC perfusion plus arterial perfusion with MPPF (n=7) or SMPPF (n=7) for the 24 hr storage (4 $^{o}$C) of the human pancreas prior to islet isolation. The SMPPF was supplemented with Glutathione (3 mM), Raffinose (30 mM) and Lactobionate (100 mM). Islets were isolated by intraductal collagenase and the number of islets in the digest measured. Viability was assayed by insulin release in response to glucose (20 mM) stimulation. The median yield of islets from the 7 local pancreata was 3,955 (2402-8000) islets/g pancreas, for the SMPPF 24 hr stored pancreata 1,576 (1030-3063), for the 24 hr stored MPPF pancreata 594 (345-1025); p=0.002, SMPPF vs MPPF. The insulin release for the local pancreata was 23.0 (7.7-37) mU/islet/hr. SMPPF=6.40 (3.90-23.7), MPPF=0.0 (0.0-3.70); p=0.002, SMPPF vs MPPF. In conclusion, although the islet yield and viability of the SMPPF perfused pancreata was not as good as that of the local pancreata, the SMPPF perfused pancreata yielded significantly more islets whose viability was markedly better than islets from the MPPF pancreata.

**FP027** The influence of the site of venous drainage and management of exocrine drainage on the function of rat pancreas grafts

Timmermann, W., Bergemann, R., Schubert, G., Schang, T., Thiede, A.
Dept. of General Surgery, University of Kiel, D-2300 Kiel 1, West-Germany

Two technical variations for pancreatic transplantion in the rat were investigated and their influence on post-operative glucose tolerance tests was measured:
1.) management of the exocrine part of the gland (occlusion vs. drainage
2.) site of venous drainage of the graft (portal vein vs. V.C.I. Grafts of the same amount of islets (whole organs) were grafted in rats using microsurgical techniques.
Materials and Methods: Isografts (Lew-Lew) were transplanted on streptozocin-diabetic recipients. Glucose-tolerance tests were performed i.v. 28 and 100 days p.op.

| | Exocrine management | venous drainage | Test d28 | Test d100 |
|---|---|---|---|---|
| 1 | drainage | v. cava | n = 12 | n = 12 |
| 2 | drainage | portal vein | n = 10 | n = 9 |
| 3 | Ethibloc occlusion | v. cava | n = 11 | n = 7 |
| 4 | control | - | n = 22 | n = 22 |

Results: At day 28 glucose assimilation in animals with venous drainage in the portal system (Gr. 1) is superior to controls and systemically drained grafts (Gr. 1,2,3). After 100 days there is no difference between exocrine drained grafts no matter what venous diversion is performed (Gr. 1,2). In Ethibloc occluded grafts (Gr.3) glucose assimilation is impaired.
Conclusion: Duct occlusion leads to impaired graft function. The diversion of venous blood of the graft in the portal vein does not show advantages in the test system.

**FP028** ***THE EFFECT OF NEUROPEPTIDE Y (NPY) ON GLUCOSE-MEDIATED INSULIN AND GLUCAGON OUTPUT BY ISOLATED ISLETS OF LANGERHANS***

*E.C. Opara, W. Burch, I.L. Taylor, O.E. Akwari*
*Duke University Medical Center, Durham, NC, USA*

*Although NPY is localized in nerve fibres within the pancreas, the role of this neuropeptide in insulin output is not clear since both stimulatory and inhibitory effects of NPY on insulin secretion have been reported. Furthermore, the direct effect of NPY on glucagon secretion has not been adequately evaluated. Our aim in this study, therefore, was to examine the direct effect of NPY on insulin and glucagon secretion using isolated islets. METHOD: Six islets microdissected from three mice were preperifused for 1 hour at 37°at the rate of 1 ml/min with Krebs-Ringer-Bicarbonate (KRB) buffer, pH 7.4, containing 5.5mM glucose (basal), 2% albumin and 100 KIU/ml trasylol that was gassed continuously with 95%/5%, $O_2/CO_2$. Basal samples were then collected and the perifusion continued with the addition of NPY in the face of 5.5mM or 27.7mM glucose. Effluent samples were radio immunoassayed for insulin and glucagon. RESULTS: NPY suppressed both basal and 27.7mM glucose stimulated insulin secretion. Thus, basal insulin release assessed as mean integrated area under the curve (AUC/20min) decreased from 1446±143pg to 651±112 pg ($p<0.05$) with the addition of $2x10^{-8}$M NPY and the AUC/20 min for stimulated insulin secretion decreased from 1973±248pg to 1426±199pg ($p<0.05$). In both cases, the inhibitory effect of NPY was followed by a prompt "rebound off response" in insulin secretion. In contrast, NPY exerted a stimulatory effect of basal glucagon release and significantly reversed the suppressive effect of 27.7mM glucose on glucagon secretion. Hence, the basal glucagon AUC/20min increased from 212±103pg to 579±316pg ($p<0.05$) while glucagon secretion at 27.7mM glucose increased from 75±26pg to 255±28pg ($p<0.01$). Also, an off response in glucagon response was observed in the post NPY perifusion period in both cases. The effect of NPY to stimulate glucagon secretion was dose-related. CONCLUSION: The direct effect of NPY on these islets hormones is to suppress insulin but stimulate glucagon secretion.*

FP029 **PERCUTANEOUS BIOPSY IN HUMAN PANCREAS TRANSPLANTATION**

RDM Allen, TG Wilson, JM Grierson, MJ Greenberg, M Earl, JR Chapman, JM Little.
Westmead Hospital, Sydney, Australia.

Lack of reliable and specific independent monitoring of pancreas allograft function prompted assessment of the value and safety of previously unreported percutaneous pancreas transplant biopsy. In 11 consecutive combined bladder drained whole pancreas and kidney transplants and 1 pancreas transplant alone, ultrasound directed 22 G fine needle aspirate biopsy (FNAB) and 20 G needle core biopsy (NCB) of the pancreas were attempted at time of routine kidney biopsy 7, 21 and 90 days after transplantation or when clinically indicated.

Pancreas FNAB were adequate for cytological assessment on 28 of 42 occasions (67%) and adequate pancreas NCB were obtained on 24 of 27 occasions (89%). There was 100% diagnostic agreement on the 15 occasions when concurrent FNAB and NCB were both adequate for assessment. When concurrent with clinical biopsy proven kidney rejection, adequate pancreas FNAB or NCB diagnosed concommitant rejection on 9 of 11 occasions (82%), whereas a significant fall in the 24 hour urinary amylase level occurred on only 3 of these occasions (27%). Biopsies were complicated by macroscopic haematuria in 1 patient and transient hyperamylasaemia on 4 occasions.

We conclude that pancreas transplant percutaneous FNAB and NCB are low morbidity procedures providing reliable and useful diagnostic material. In the combined transplant procedure, pancreas rejection may occur less frequently, or more probably, later than in the kidney.

### FP030 ULTRASOUND AND DUPLEX DOPPLER IMAGING OF PANCREAS GRAFT TRANSPLANT

Castoldi R., Ferrari G., Angeli E.,Paesano P.L., Staudacher C., Secchi A., Del Maschio A., Pozza G., Di Carlo V.

University of Milan, San Raffaele Hospital - Milan (ITALY)

We evaluated the role of US-scan for pancreas morphological examination and peripancreatic surgical complications visualization and the possibility to use duplex-doppler for pancreas vascular thrombosis monitoring.
In University of Milan - San Raffaele Hospital in period July 1985-January 1990, 31 pancreas transplants in 29 patients (two patients were retransplanted), were performed. In 28 cases pancreas was transplanted simultaneously to a kidney graft and in 3 cases nonsynchronously. A segmental pancreas was duct injected with Neoprene and engrafted intraperitoneally to the iliac vessels in 28 cases and a duodenal-whole pancreas graft was bladder drained in two cases . The overall one-year patient survival was 93% and one-year pancreas graft survival 70%. Five cases were unsuccessful for venous or arterial thrombosis, one case for chronic rejection and in two cases no immediate function was observed for harvesting or preservation causes. Two patients died for myocardial infarction and septic bleeding.
The morphological evaluation of the pancreas graft by US-scan at five three years showed a progressive decrease of graft size and a decreasing ecogenicity of parenchima. In the early postoperative period US-scan allowed to detect in 10 cases (30%) a thin peripancreatic fluid collection without any clinical correspective. In 2 cases a blood collection and in 1 case an abscess were observed, which were drained under US-guidance. In 3 cases an asymptomatic pancreatic graft pseudocyst was detected at long-term control.
From December 1987 duplex doppler pancreas graft monitoring was started. From that time 17 grafts out of the 19 transplanted (90%) could be studied throughout the postoperative period proving the feasibility of a proper thrombosis monitoring. In this period four vascular thrombosis occurred. In three cases the exam could detect no flow signal in the graft splenic vein or artery and in the other case the graft vessels, whose visualization is necessary for duplex doppler evaluation, could not be found out due to the technique of engraftment (pancreas was placed deeply intraperitoneally through a laparotomic access so that vessels were masked by small bowel).
In this preliminary study US-scan demonstrated as an excellent diagnostic tool for pancreas graft morphological follow-up and in the early postoperative period duplex doppler may be considered a primary diagnostic tool for surgical vascular and nonvascular complications.

### FP031 ORTHOTOPIC LIVER TRANSPLANTATION FROM LIVING DONORS IN THE DOG

D. Cherqui, J. C. Emond, A. Pietrabissa,
M. Michel, M. Roncella and C. E. Broelsch
Department of Surgery, University of Chicago,
Chicago Il., USA

Orthotopic liver transplantation (OLT) using partial grafts harvested from living donors would represent a further alternative to the limited supply of hepatic grafts, especially in pediatrics. We report herein the results of an original technique of living donor OLT that we have developed in the dog.

This study was conducted in male mongrel dogs weighing 25-30 kg for the donors and 10-15 kg for the recipients. The donor operation consisted in haryesting the left lobe of the liver as a graft. The recipient operation consisted in the implantation of the graft in the orthotopic position after total hepatectomy with preservation of the inferior vena cava.

Ten survival experiments were undertaken. The first donor died of infected liver necrosis of the quadrate lobe. All other donors survived without major complication. Among the 10 grafts, only 9 were used. Substantial survival could be obtained in 3 dogs. One recipient survived for 48 hours and 2 for 24 hours but their graft was functionning and producing bile. Two dogs died intraoperatively. The 4 other recipients developed an outflow block of the graft after reperfusion leading to lethal hemorrhage from the transected surface.

This work is, to our knowledge, the first experimental study of OLT using living donors. It provides a technical basis to the clinical use of living hepatic allograft donors which now depends on ethical issues.

**FP032** Effect of HLA-compatibility on human Liver allograft rejection episodes.

W. Lauchart, M. Hunke and R. Pichlmayr.

Dep. of Surgery Tübingen University and Div. of Abdominal- and Transplantation Surgery, Hannover Medical School, F.R.G.

The influence of HLA-compatibility and acute allograft rejection episodes (ARE) in kidney and bone marrow transplantation is well established, its role in liver transplantation is still controversially discussed. We therefore performed non-parametric regression analysis in a consecutive series of 97 adult liver allograft recipents, analysing the occurence of ARE and their concomitantclinical and laboratory features; diagnosis of ARE being defined as : (a) histology proven, (b) requiring treatment, and (c) response to treatment.
Complete information on ABO-, and HLA-A/B/Dr-compatibility were available in 77 donor-recipient combinations their records were evaluated for the occurence of ARE for the first 4 weeks posttransplant.

The occurence of 72 ARE correlated with HLA-Dr- and B/Dr- (p=0.031 and 0.036 respectively), but not with HLA-A-locus or ABO-blood group compatibility. Only elevated values of bilirubin and transaminases correlated with ARE (p=0.026 and 0.005 respectively), bilirubin was the only relevant parameter, correlating with ARE in the first posttransplant week.

We conclude, that in the first 4 weeks after liver transplantation, there is a close correlation between ARE and HLA-B- and Dr-compatibility.

### FP033 CAN APROTININ REDUCE BLOOD LOSS IN ORTHOTOPIC LIVER TRANSPLANTATION?

W O Bechstein, H Riess, P Neuhaus
R Slama, R Steffen, B Lefebre
Universitatsklinikum Rudolf Virchow
Berlin, FRG

Aprotinin, an antifibrinolytic agent, has been reported to reduce blood loss in repeat open heart surgery (Royston et al 1978). The effect of aprotinin on blood loss in liver transplantation was investigated in a prospective, non-randomized study.

From September 1988 until January 1990 60 consecutive orthotopic liver transplantations were carried out in 56 adult patients according to standard surgical techniques with regular use of veno-venous bypass during the anhepatic phase. Indications for liver transplantation were mainly postnecrotic cirrhosis (36),cholestatic disease (6), acute hepatic failure (5), primary hepatobiliary malignancy (4), re-transplantation (4), and others (5). The first group of 10 patients (Group I) was treated without aprotinin, for the next 50 transplantations (Group II), Aprotinin was applied in a dose of 2.0 Mio. i.v. intraoperatively. Retransplant procedures were excluded from the analysis.

Currently, 52 of 56 patients (92 %) are alive, 30-day mortality is 0%. Intraoperatively, a mean of $9.7\pm5.5$ units red packed cells were substituted in group I vs. $7.5\pm4.6$ units in group II (n.s.). Fresh frozen plasma was substituted with $10.4\pm6.2$ (group I) vs. $9.1\pm6.7$ (group II) units (n.s.). However, with use of aprotinin the operative field at the end of the procedure was usually dry and often, postoperative drainage was omitted altogether.

Even though a beneficial effect of aprotinin on blood loss in OLT could not be proven statistically in this study, the trend towards less blood loss and clinical impression of reduced fibrinolysis prompted us to continue routine use of aprotinin.

References:

Royston D et al. Lancet 1987; II:1289

### FP034 LIVER TRANSPLANTATION IN HBsAG POSITIVE PATIENTS

P. Neuhaus, G. Blumhardt, H. Keck,

W.O. Bechstein, Th. Steinmüller, A. Müller,

U. Hopf

Liver transplantation in HBsAg positive patients is associated with increased morbidity and mortality from perioperative complications and reinfection.

PATIENTS AND METHODS
61 OLT were performed in 56 patients, 16 patients (29 %) were HBsAg positive. For passive immunization HBsAg-positive recipients received 10 000 IU anti-Hepatitis B hyperimmunoglobulin (HBH) in the anhepatic phase and 1000-2000 IU/day for the first week. (Protocol design by Lauchart 1987). The protocol was extended to give further HBH for 12 months as soon as serum antibody levels dropped below 100 IU/l. After OLT patients received interferon therapy for 3 months with 3 x 1-5 mio.U/week.

RESULTS
At present 14 of 16 patients (87,5 %) are alive, 6 patients (38 %) so far permanently eliminated HBsAg. In 7 patients (44 %) only transient antigen elimination could be achieved, 2 patients in this group developed fulminant hepatitis and were retransplanted. Both patients died after reinfection of their new grafts. 3 patients (18 %) permanently retained HBsAg after OLT. 13 patients received IFN, which was in general tolerated.

CONCLUSION
Mortality in our HBsAg-positive recipients is higher than in other patients, graft reinfection being the main single risk factor. Treatment with HBH appears to be useful, the influence ot IFN has to be further evaluated.

### FP035 GLUCOSE METABOLISM AND HORMONAL STATUS IN THE JAUNDICED FREELY MOVING RAT

C SMADJA, J MORIN, P RUEL, D FRANCO. Groupe de Recherche sur la Chirurgie du Foie et de l'Hypertension Portale, Hôpitaux Bicêtre, Le Kremlin Bicêtre ; Paul Brousse, Villejuif, France.

Obstructive jaundice (OJ) is associated with major nutritional disorders. The alterations of glucose metabolism during OJ have been poorly defined. OJ was created in male Sprague-Dawley rats by common bile duct ligation. Chronic catheters were inserted into the portal and jugular veins. Control rats (CR) underwent a sham operation. Experiments were performed in freely moving rats on the 8th postoperative day after 6-7 hours of food removal. Glucose turnover rate was assessed with ($3-^3H$) glucose. Peripheral blood glucose and non esterified fatty acids and portal blood glucose were similar in jaundiced (JR) and CR, whereas insulinemia was lower in peripheral (66 ± 13 µU/ml vs. 116 ± 16 µU/ml ; $p < 0.05$) and portal (85 ± 9 µU/ml vs. 140 ± 18 µU/ml ; $p < 0.02$) blood of JR. Glucagonemia was higher in peripheral (216 ± 23 pg/ml vs. 138 ± 24 pg/ml ; $p < 0.05$) and portal (258 ± 30 pg/ml vs. 142 ± 35 pg/ml ; $p < 0.05$) blood of JR. Molar insulin to glucagon ratio was lower in peripheral ($p < 0.01$) and portal ($p \leq 0.01$) blood of JR. Glucose turnover rate (24.4 ± 2.3 mg $kg^{-1}$ $min^{-1}$ vs. 14.9 ± 0.8 mg $kg^{-1}$ $min^{-1}$ ; $p < 0.01$) was higher in JR. Liver glycogen content was negligible in JR and CR. Using the 2-deoxyglucose technique, it was shown that the increased glucose utilization in JR was localized mainly in working muscles (soleus, $p < 0.02$ ; adductor longus, $p < 0.01$) diaphragm ($p < 0.001$) and myocardium ($p < 0.02$) whereas glucose consumption was normal in non working muscles and splanchnic tissues. These results indicate that OJ induce a catabolic state. The metabolic profile observed in the present study is compatible with a septic state.

FP036 MEASUREMENT OF BODY WATER COMPARTMENTS IN OBSTRUCTIVE JAUNDICE IN HUMANS

X. Carulla, L. Oms, X. Guirao, F. Martínez-Ródenas,

References:

Martínez-Ródenas F, Oms L, Carulla X et al. Br J Surg 1989; 76:461-464.

FP037

### ENDOTOXAEMIA AND ENDOTOXIN-RELATED MORTALITY IN OBSTRUCTIVE JAUNDICE

**T. Diamond, S. Dolan, R.L.E. Thompson, B.J. Rowlands. Department of Surgery, The Queen's University of Belfast.**

**Introduction:** Gut-derived endotoxaemia has been implicated in post-operative complications in jaundiced patients. It is thought that absence of bile in the gut predisposes to portal absorption of endotoxin and that endotoxaemia is reversed by oral bile salt therapy or internal biliary drainage and return of bile to the gut, but not by external drainage. We postulate that biliary obstruction and the integrity of hepatocyte and Kupffer cell function are more important factors than GI bile flow in the development and reversal of endotoxaemia.

**Experiment 1:** Serum endotoxin concentrations were measured in control rats, following choledochovesical fistula (CVF) and bile duct ligation (BDL) and following relief of biliary obstruction by internal drainage (ID - choledochoduodenostomy) and sterile external drainage (ED - choledochovesical fistula) (Quantitative Limulus Assay - pg/ml).

| | Portal Conc (mean±SE) | Systemic Conc (mean±SE) |
|---|---|---|
| Controls (n=10) | 224± 85 | 240±77 |
| CVF 2 wks (n=15) | 493±171 NS | 272±115 NS |
| BDL 2 wks (n=15) | 1304±129 p<0.005* | 918±110 p<0.005* |
| BDL 2 wks+ID 2 wks (n=8) | 442± 87 NS | 328± 67 NS |
| BDL 2 wks+ED 2 wks (n=8) | 383±165 NS | 376± 94 NS |

**Experiment 2:** Mortality was measured following administration of IV lead acetate (5mg/100g) and oral endotoxin (5mg/100g).

| | | |
|---|---|---|
| Controls (n=15) | No deaths | |
| CVF 2 wks (n=15) | 3 deaths NS | |
| BDL 2 wks (n=15) | 13 deaths p<0.005# | *Mann Whitney |
| BDL 2 wks+ID 2 wks (n=10) | 3 deaths NS | #Chi-squared |
| BDL 2 wks+ED 2 wks (n=10) | 1 death NS | |

Bilirubin was elevated in BDL compared to Controls, CVF, ID and ED*

**Conclusions:** Significant endotoxaemia and mortality occurred in BDL but not in CVF. Relief of obstruction by both internal and external drainage reversed endotoxaemia and mortality. This confirms that biliary obstruction is a more important factor than GI bile flow in the development and reversal of endotoxaemia.

**FP038** IMMUNOSUPPRESSION IN SURGICAL JAUNDICE

S. Dahanukar, N. Rege, R. Bapat Dept. of
Pharmacology and Dept. of G.E. Surgery
Seth G.S.M. College & K.E.M. Hospital, Bombay, India.

Despite the aseptic precautions and judicious use of antimicrobial agents, incidence of biliary sepsis is high in patients with surgical jaundice. An animal model of surgical jaundice was therefore set up to determine the immune status in cholestasis. The phagocytic and microbicidal functions of neutrophils in peripheral blood and peritoneal macrophages were assessed. A significant depression of these functions compared to normal rats was observed. A defect in the serum of cholestatic rats, responsible for the depressed activity of phagocytic cells was also dectected. These rats showed an increase susceptibility to Escherichia coli infection (mortality 77.78%). An attempt was made to modulate immunosuppression by treating the rats with Tinospora cordifolia an Indian medicinal plant, 100 mg per Kg. for 7 days, following development of cholestasis. The extract improved the cellular immune functions. Mortality following Escherichia coli infection was significantly reduced to 16.67%. This study shows that cholestasis results in immunosuppression and indicates the need for an immunomodulator in management. The plant Tinospora cordifolia fulfils this need by consolidating host defenses.

References

Thatte U.M., Dahanukar S.A. Phytotherapy Research 1989 : 3 : 43

Dahanukar S.A., Thatte U.M. Ayurveda Revisited : 1989
Popular Prakashan, India.

### FP039 MORPHOLOGICAL CHANGES OF EXTRAHEPATIC BILE DUCTS DURING OBSTRUCTION AND SUBSEQUENT DECOMPRESSION BY ENDOPROSTHESIS

*T.Karsten, P.P.Coene, A.Bosma, T.van Gulik, J.van Marle, N.Lygidakis, J.James and M.van der Heyde. Dept. of Surgery, Academic Medical Centre, Amsterdam, The Netherlands*

The purpose of this study was to document the morphological changes of the extrahepatic biliary tract during obstruction and the effects of biliary decompression by means of an endoprosthesis on the bile duct wall. The pathological changes were studied by light microscopy and scanning electron microscopy.

Human common hepatic ducts specimens were obtained during surgery from patients with a distal common bile duct obstruction due to tumor. 10 patients had obstructed bile ducts of 4 weeks duration (Group A) and 10 patients had undergone endoscopical stenting for an average period of 8 weeks (Group B). Three autopsy specimens from subjects without biliary pathology were examined as controls.

Group A specimens showed dilated ducts, approximating three times the control size with moderately thickened ductal walls. A mild, proliferative chronic inflammation with edema throughout the ductal wall was present without any clear evidence of fibrosis. The epithelium was flattened but still intact. In Group B, the ducts were narrowed, approximating the diameter of the endoprosthesis with markedly thickened ductal walls. Chronic inflammatory changes were prominent with fine bundles of newly formed collagen in the ductal wall, indicative of fibrosis. The mucosa showed ulceration and areas denuded from epithelium.

In conclusion, the initial dilatation and thickening of the extrahepatic bile ducts during biliary obstruction are associated with a mild chronic inflammation in the absence of fibrosis. The presence of an endoprosthesis however, induces severe chronic inflammatory changes with evident fibrosis. Further studies are necessary to evaluate the biocompatibility of different types of biliary endoprosthesis in relation to the severity of the induced inflammatory reaction.

### FP040 NEW INSIGHTS IN SPHINCTER OF ODDI DISORDERS

A. Montori, L. Masoni, G. Miscusi
III Patologia Chirurgica- University
"La Sapienza" Rome - Italy

The diagnostic approach to the so-called sphincter of Oddi (SO) motility disorders has been greatly influenced by the results of ERCP manometry which allows direct investigation of SO motor activity.
So far 23 patients with CBD stones have been investigated by this method because the radiological examination indicated SO disorders might be present (Group I). Moreover, 18 patients without CBD stones, in whom any other organic biliary and pancreatic disease had been excluded, were investigated as well because of recurrent episodes of biliary-like pain (Group II); in only a few of these patients this symptom was accompanied by one or more of the following: CBD dilation (larger than 12 mm); emptying of the ERCP contrast medium took longer than 45 min; abnormal liver function tests. Noticeably, 13 patients in Group II (72.2 %) had already undergone cholecystectomy, being classified as post-cholecystectomy syndrome patients.
Moreover, 8 healthy volunteers served as controls.
Manometric anomalies could he demonstrated in 3 patients in Group I (13%) and in 10 patients in Group II (55.5%), of which all but one were without gall-bladder "in situ"; in these last 10 patients endoscopic sphincterotomy was performed for cure.
Our results show that the incidence of SO motor anomalies is very low in the presence of choledocholithiasis, while it is substantial in patients with unexplained biliary-like pain, especially if they have already undergone cholecystectomy. These observations would suggest that, unlike the traditional view, SO disorders are rarely secondary to biliary lithiasis.
In our opinion ERCP manometry should be considered the final step investigation in the diagnostic process of unexplaned biliary-like pain which can be related in many instances to an abnormal SO motility pattern.

### FP041 PRIMARY SCLEROSING CHOLANGITIS IN PATIENTS WITH ULCERATIVE COLITIS.

**HH Rasmussen, J Fallingborg, PB Mortensen, U Tage-Jensen, V Kruse, L Freund and SN Rasmussen.**
**Departments of Medical and Surgical Gastroenterology and Diagnostic Radiology, Aalborg, DK.**

From January 1st 1976 to December 31st 1987, 295 patients with ulcerative colitis (UC) were registrered. Twenty-four of these demonstrated alkaline phosphatases persistently above the upper reference value and were investigated further with liver chemistry, ultrasound of the liver/bile ducts and endoscopic cholangiography (ERC). Primary sclerosing cholangitis (PSC) was diagnosed in 11 patients. Female/male ratio was 7/4, age 17-56 years, mean 32 years. Two patients with debut of PSC before UC was diagnosed. Duration of disease was 1-16 years, mean 6,0 years; two died after 1 and 9 years respectively, from cholangiocarcinoma. Initial symptoms were most often weakness, abdominal pains, pruritus, fever and alkaline phosphatases were raised to about 5 times normal value; ASAT marginally raised; bilirubine was normal. ERCP revealed 10 patients with intra and extrahepatic changes (in 1 patient only the extrahepatic bile ducts were visualized). Sixty-four percent were asymptomatic and 36% were complicated with cholestasis/cholangitis. The UC was with low activity (90%). Extent of disease was extensive or pancolitis in 73% and left sided in 27%.
**Conclusion:** Prevalence of PSC in this population of patients with UC was 3,7% (95% confidence limits : 1,84 - 6,49%). More information is needed in regard to optimum management and surveillance of liver function, malignancy, complications, and selection of cases for liver transplantation.

### FP042 OUTCOME OF PRIMARY SCLEROSING CHOLANGITIS

**T Ismail, L Angrisani, J Buckels, P McMaster**
**Queen Elizabeth Hospital, Birmingham, UK.**

Primary Sclerosing Cholangitis (PSC) is an idiopathic disease characterised by chronic inflammatory obliterative fibrosis affecting the intra- and extra-biliary tree. The natural history is unknown and treatmentschedules are poorly defined. We reviewed our experience to define the prognostic variables and outcome of different surgical options.

48 patients (30 male; median age 39 years; range 8 - 67) with symptomatic PSC were reviewed. 44% (n=21) died; overall 5 year actuarial survival was 30%. 8.5% (n=4) developed or had associated hepatobiliary malignancy. 27 biliary operations (16 specifically for PSC) were performed in 23 patients of whom 11 died. Serum bilirubin was the only parameter that improved postoperatively. 17 patients (35%) underwent orthotopic liver transplantation (OLT); 9 are currently alive (1 year projected survival of 55%). Previous biliary surgery correlated with a poor outcome ($p<0.0001$) after OLT. Males, cirrhotics, duration of disease (>3 years) and a serum bilirubin of >100 at presentation were associated with a poor outcome ($p<0.05$).

These data provide evidence that PSC is a progressive disease and conventional surgical procedures have little influence on outcome. Reconstructive biliary surgery should be reserved for localised extra-hepatic disease, as it adversely affects outcome following OLT.

### FP043 INFLUENCE OF INDIVIDUAL BILE ACIDS IN E.COLI PERITONITIS

R. Andersson, K.-G. Tranberg, J. Lillienau, C. Schalén, U. Srinivas, L. Larsson, A. Sonesson, S. Bengmark. Departments of Surgery, Medical Chemistry, Medical Microbiology, Clinical Chemistry, Technical Analytical Chemistry, Medical Center, Lund University, Sweden

Earlier studies from the present group have shown that in experimental E.coli peritonitis the intraperitoneal (i.p.) injection of whole bile increases growth of bacteria. The purpose of the present study was to evaluate the influence of individual bile acids and bilirubin on pathophysiological mechanisms in E.coli peritonitis.

274 male Sprague-Dawley rats weighing 300-330 g were used. E.coli peritonitis was induced by i.p. injection of 3 x $10^8$ colony forming units of E.coli. Whole bile, individual bile acids (cholic, deoxycholic or chenodeoxycholic acids), bilirubin or saline were added to the i.p. injection in the various experiments. Mortality was noted. Bacterial viable counts were determined in peritoneal fluid and in blood at 1, 4 and 10 h following induction of peritonitis. Superoxide release from peritoneal phagocytes were determined from cells obtained at 10 h after induction of peritonitis. Clearance of bile from the peritoneal cavity was estimated after i.p. injection of radiolabelled deoxycholic acid together with E.coli and bile. Possible effects of bile or bile salts on bacterial growth and endotoxin release were determined in in vitro experiments.

Each of the bile acids aggravated the E.coli peritonitis with increased bacterial counts in the peritoneal cavity and in blood and increased mortality. Deoxycholic acid was the most deleterious of the bile acids, causing suppression of superoxide release by peritoneal phagocytes, like whole bile. In vitro, bile acids did not affect growth of E.coli, but cholic and deoxycholic acid increased the release of endotoxin.

Bile acids are responsible for the noxious effect of bile in E.coli peritonitis. Through their detergent properties, bile acids might aggravate the peritonitis by solubilizing the cell membranes of both bacteria and phagocytes.

### FP044 ELECTROMYOGRAPHIC EVALUATION OF THE GASTRO-INTESTINAL TRACT IN PATIENTS WITH ROUX-EN-Y LIMB

J.C.U. Coelho, L. Clemente, J.E.F. Matias

A.C.L. Campos

Federal University of Paraná, Curitiba,

Paraná, Brazil

A Roux-en-Y stasis syndrome was described recently in few patients following gastric operations. The motility of the Roux-en-Y biliary limb has not been evaluated yet. In the present study, we report electromyographic studies of the gastrointestinal tract of 10 patients with Roux-en-Y hepaticojejunostomy. Seven pairs of bipolar extracellular electrodes were implanted in the antrum, duodenum, proximal jejunum (15 cm proximal to the jejunojejunostomy), distal jejunum (15 cm distal to the jejunojejunostomy), ileum, proximal Roux-en-Y limb and distal Roux-en-Y limb. All four phases of the MMC (migrating motor complex) were identified in the antrum and small bowel, including the Roux-en-Y limb. The duration of the MMC in the duodenum varied from 72 to 192 minutes, with an average of 95,4 ± 34 min. Phase III migrated along the Roux-en-Y in all recordings. In five recordings, phase III started in the duodenum and migrated sequencially to the proximal jejunum, Roux-en-Y, distal jejunum and ileum. In ten recordings, phase III started in the Roux-en-Y and then migrated to the duodenum, proximal jejunum, distal jejunum and ileum. In six recordings, phase III started symultaneously in the duodenum and Roux-en-Y and then migrated to the jejunum and ileum. Food ingestion caused substitution of the MMC by the fed activity pattern in all recordings in the antrum and small bowel, including the Roux-en-Y limb. The findings of the present study indicate that the electromyographic activity of the Roux-en-Y may remain normal during fasting and fed states even many years after its creation.

### FP045 EFFECT OF COLLOID OSMOTIC PRESSURE ON THE FORMATION OF INTESTINAL EDEMA DURING WHIPPLE-OPERATION

F. Pelster, Th. Prien, U. Sulkowski, W. Pircher, H. Bünte, P. Lawin

Dept. of General Surgery and Anaesthesiology of Münster Univ. Hospitals, Münster, F. R.G.

The effects of intraoperative changes in colloid osmotic pressure (COP) on the formation of intestinal edema were studied in patients during hemipancreato-duodenectomy according to Whipple. 18 patients (ASA I-II) were randomly assigned to one of three groups. They received either lactated Ringer's (RL-GROUP, n=6), 10% hydroxyethyl starch (HES-GROUP, n=6), or 20% human albumin (HA-GROUP, n=6) as volume replacement solution (VRS). VRS was given to maintain central venous pressure at the preoperative level. Jejunal specimens were obtained after the first transsection of the jejunum (J1) and prior to the jejuno-jejunostomy (J2). Their water fraction (g $H_2O$/g tissue dry weight = WF) was measured gravimetrically. COP was determined prior to induction of anesthesia (PRE) and upon removal of the second jejunal sample (J2). In the RL-GROUP 3,850 ± 584 ml (data are mean ± SEM) VRS were infused from PRE to J2, in the HES-GROUP 1,358 ± 45 ml, and in the HA-GROUP 463 ± 49 ml. From PRE to J2, COP decreased from 20.3 ± 0.5 (mmHg) to 14.1 ± 0.6 in the RL-GROUP, remained at 22.0 ± 0.9 in the HES-GROUP, and increased from 20.7 ± 0.9 to 28.1 ± 0.9 in the HA-GROUP. From J1 to J2, WF increased from 4.45 ± 0.13 to 4.79 ± 0.2 in the RL-GROUP, remained unchanged with 4.18 ± 0.16 and 4.10 ± 0.26 in the HES-GROUP, and decreased from 4.13 ± 0.15 to 3.68 ± 0.1 in the HA-GROUP. All changes are statistically significant different (Student's t-test for paired data; $p \leq 0.05$).

### FP046 IMPROVED RESULTS FOLLOWING RADICAL OPERATIONS FOR PANCREATIC ADENOCARCINOMA

F. Michelassi, G.E. Block, University of Chicago, Chicago, Illinois, U.S.A.

More than half a century after the first radical operation for treatment of carcinoma of the head of the pancreas, its long-term efficacy remains controversial. Many authors over the last decade have reported five year survival rates between 0 and 12.5%. On the other hand, recent reports of five year survival rates as high as 18% have been published (i.e., Cameron, Trede). In an effort to confirm a possible improvement in outcome over time, we analyzed the results of 549 adenocarcinoma of the head of the pancreas diagnosed at the University of Chicago Medical Center between 1946 and 1987. Operability rate was 87% (n=478). A combination of laparotomy, biopsy and bypass was performed in 399 patients, only one of whom survived five years (0.2%). Perioperative mortality and five year survival after radical resections are highlighted by the enclosed table in four different consecutive periods between 1946 and 1987. Perioperative mortality has decreased from 55% in the first twelve years to 5% in the last six (1946-69, 53%; 1970-87, 8%; p<0.02). Similarly, long term survivals were achieved only in the last 18 years of the series, with a crude five year survival of 12.5% in the third period and an actuarial five year survival of 15.8% the last six years. These results suggest that a curative resection offers the best chance of long term survival and that results of radical surgery for pancreatic adenocarcinoma have improved over time.

| | Resections n | Perioperative deaths (%) | 5 yr Survival n | (%) |
|---|---|---|---|---|
| 1946-57 | 20 | 11 (55%) | 0 | ( 0%) |
| 1958-69 | 8 | 4 (50%) | 0 | ( 0%) |
| 1970-81 | 31 | 3 (10%) | 4 | (12.5%)* |
| 1982-87 | 20 | 1 ( 5%) | 3** | (15.8%)*** |

* Crude 5 yr survival

** Still living, NED, <5 yrs

*** Actuarial 5 yr survival

**FP047** PYLORUS-PRESERVING PANCREATODUODENECTOMY: FURTHER CLINICAL EXPERIENCE IN 90 CASES

F. Mosca, P. C. Giulianotti, G. Sartoni, A. Pietrabissa,

A. Guadagni, T. Balestracci, A. Costa, J. Romagnoli

II Department of Surgery Pisa University- Italy

Pylorus-preserving pancreatoduodenectomy (PPPD) introduced a considerable technical progress in surgery during the last ten years. Our experience with 90 procedures is here reported. PPPD does not adversely affect morbidity or survival as compared to Whipple resection, even dealing with pancreatic cancer, moreover improving the patient postoperative quality of life. 90 PPPD (54 M; 36 F), including 8 total pancreatectomies, were performed between January 1982 and December 1989. 13 pts had chronic pancreatitis, 50 pancreatic cancer and 27 periampullary cancer. The mean age was 63.6 ± 11.6 years in the cancer group and 44 ± 9.2 in the pts with chronic pancreatitis. Operative mortality rate was 10% ( 4.3% in the last 3 years ), half of the deaths occuring not for abdominal causes. The morbidity rate was 24.6% and in 4 pts a reoperation was required. Postoperative nasogastric suction was mantained for an average of 12 days per pt ( 5 -26), the delay in gastric emptying lasting for more than 2 weeks in only 15% ( no addictional surgery required ). Reintroduction of food was satisfactory accomplished by the majority of pts. Weight gain six months after surgery ( not including cases with early cancer recurrence ) average 3.5 Kg, being 7 to 10 Kg in some instances. Specific late complications: bleeding from duodenal peptic ulcer, successfully treated with conventional medical therapy, occurred in 1 pt 42 months after surgery. 2 pts bled from antral gastritis and were medically managed. 12 pts operated on for pancreatic cancer are still alive: 1 is free of recurrence after 7 years; 1 is alive more than 4 years after resection; 2 more than 3 years ( 1 with liver metastases ) and the remainig 8 survivors are between 6 and 12 months from surgery. 27 pts died by cancer recurrence: the mean survival in this group was 10.27 ± months. 9 pts out of 27 resected for periampullary cancer were elegible for a 5 years follow-up: their mean survival was about 72%. All pts operated on for pancreatitis are alive; late complications in this group were: 1 stricture of the hepaticojejunostomy, successfully treated with percutaneous dilatation and 1 small bowel subocclusion due to adhesions spontaneously relieved. Pain relief was satisfactory obtained in every pt. Survival analysis, retrospectively comparing 2 omogeneous groups of pts who underwent resection for pancreatic cancer, failed to demonstrate any significant difference between those in whom a Whipple procedure was performed as compared to the ones resected according to Longmire's tecnique.

### FP048 GASTRIC EMTYING AND ENTERO-GASTRIC REFLUX FOLLOWING PANCREATICODUODENECTOMY WITH PYLORUS PRESERVATION

G.Clemente,S.Furgiuele,R.Bellantone,G.B. Doglietto,F. Crucitti - Rome (Italy)

Preservation of the pylorus following pancreaticoduodenectomy represents an attractive alternative to the standard Whipple's procedure, in order to improve clinical and functional results (1,2).

Six male patients(mean age 62,6 yrs, range 57-72) submitted to pancreaticoduodenectomy with pylorus preservation for ampullary (4 cases:cefalopancreasectomy) or pancreatic tumours (2 cases: total pancreasectomy) were submitted 4-8 months after surgery to the following examinations: upper digestive endoscopy; esophageal manometry; determination of gastric emptying rate by scintigraphic method; esophago-gastric 24-hour pH monitoring. The results of the last two examination were compared to the data obtained from a control-group of six subjects.

Endoscopy revealed signs of esophagitis and/or gastritis in 4 patients. At the manometry, LES pressure was lower than 20 mm Hg in three patients that showed, in addition, an aspecific motility disturbance. No significative differences were found between patients and controls regarding to gastric emptying. pHmetric data showed a pathological gastro-esophageal reflux (according to Johnson-DeMeester criteria) in 4 patients. Entero-gastric reflux (percentage of time with gastric pH greater than 4) was significantly increased in all patients (mean 26,38%, range 12,5-39,8) in comparison to the control-subjects (mean 5,96%, range 3,2-11,2) ($p < 0,01$).

Our data showed that entero-gastric reflux is significantly increased following Traverso-Longmire procedure. A continuous medical treatment with $H_2$blocking agents is necessary to avoid gastric ulceration.

References:
1) Traverso L, Longmire WP. Surg Gynecol Obstet 1978:146,959
2) Braasch JW, Rossi RL. Ann Surg 1986:204,411

### FP049 CURATIVE PROSPECTIVES IN CASE OF LOCALLY ADVANCED PANCREAS CANCER

J. Jeekel, J.H.G. Klinkenbijl
Erasmus University Rotterdam, Rotterdam,
The Netherlands.

Treatment of patients with locally advanced pancreas cancers is generally regarded to be of no use. We studied our treatment results in patients with irradical resection of the pancreas tumour and in patients with locally unresectable pancreas cancer.
Between 1978 and 1987 377 patients were treated for pancreas- and peri-ampullary cancer. A curative resection could be performed in 106 patients. Resections were considered non-curative in 30 cases, either because resection site proved to contain tumour (T3) or because of positive lymphnodes outside the resection area. Survival in this group (1 year 64%, 2 years 19%, 3 years 10%) was not different from patients with a curative resection for pancreas cancer (1 year 95%, 2 years 23%, 3 years 15%, 5 years 10%; p = 0.91), and significantly better than after drainage procedures. Treatment with radiotherapy and 5-FU was given to patients with unresectable cancer (T3NxMO). Survival in this group was not different from patients with curative resection and also significantly better than after drainage procedures.
In conclusion: survival of patients with locally advanced pancreas cancer, treated with irradical resection or radiotherapy and 5-FU, was comparable to that of patients who had a radical resection.Thus patients with localized pancreas cancer that can not be radically resected, should either undergo palliative resection or, in case of locally unresectable cancer, be treated with radiotherapy and 5-FU.

### FP050 RESECTION VERSUS PALLIATIVE SURGERY IN THE TREATMENT OF PANCREATIC CANCER: A COST-BENEFIT ANALYSIS

A.Zerbi, M.Carlucci, R.Castoldi, F.Motta, E. Fiacco, D.Parolini, C.Staudacher, V.Di Carlo - Pat. Chirurgica, IRCCS San Raffaele, University of Milan, Italy.

Recent reports debated the cost-effectiveness of resective procedures in pancreatic cancer. A cost benefit analysis has been undertaken in order to compare the different therapeutical approaches to pancreatic cancer.
Between October '80 and December '88 177 patients with cytologically or histologically proved pancreatic adenocarcinoma were observed : 34 patients underwent nonsurgical therapy (group A), 29 laparotomy (group B), 68 biliary and/or gastric by pass (group C), 46 pancreatic resection (38 pancreatoduodenectomy, 7 distal pancreatectomy and 1 total pancreatectomy) (group D). For each treatment group multiple parameters were analyzed: length of hospitalization, postoperative complication rates, number of reoperations, survival, hospital charges. All patients were classified according to Hermreck's staging. Hospital charges were analyzed by utilizing standard parameters of our Institute which included hospital stay, diagnostic and surgical procedures, adjusted to december 1989 dollars.
Median hospital stay was 21 days in group A, 27 days in group B, 32 days in group C, 36 days in group D. We observed a complication rate of 13.8% in group B, 38.2% in group C, 39.1% in group D. Six patients of group C (8.8%) underwent reoperation, as well as 4 patients of group D (8.7%). Overall operative mortality was 6.9% (10 patients): 6.9% in group B, 8.8% in group C, 4.3% in group D. Non surgically treated patients and those who underwent a laparotomy survived respectively an average of 3 and 5 months; patients who underwent by pass survived an average of 6 months and those who underwent resection 12 months. The average total charges resulted $ 9.230 in group B, $ 12.307 in group C and $ 15,384 in group D.
Our results show that resection of pancreatic cancer allows a substantial improvement in prognosis in respect to palliative surgery, and it is not related to significantly higher costs in terms of hospital stay, complications, operative mortality and hospital charges.

**FP051** CA 19-9: POSTOPERATIVE PROGNOSTIC INDEX IN PANCREATIC CANCER.

C. Pasquali, C. Sperti, S. Catalini, C. Filipponi, A. Alfano D'Andrea, A. Piccoli*, S. Pedrazzoli
Clinica Chirurgica 1 & Medicina Interna*, Univ. of Padua, Italy

Ca 19-9 as tumoral marker in pancreatic cancer showed a good sensitivity and its usefulness in differential diagnosis with chronic pancreatitis has been previously established. In this study we evaluated the reliability of pre and postoperative Ca 19-9 determination in pancreatic cancer both as prognostic index and marker of cancer recurrence after resective surgery.
53 patients (33 M and 20 F) with histologically proven pancreatic carcinoma (PK) were investigated. Ca 19-9 serum determination (n.v. $< 37$ U/ml) were performed before and after operation for each patient (15, 30 days and every 3 months after surgery). Three monthly follow up investigations included chest X rays, abdominal CT scans or US scans and routine laboratory tests.
24 PK patients underwent resective surgery (19 partial and 5 total pancreatectomy) and 29 had only palliative surgery. TNM staging was I in 10 cases, II in 9 cases, III in 14 cases and IV in 20. Wilcoxon test and Mantel Cox test were used for statistics.
22/24 patients who underwent resective surgery had high preoperative levels of Ca 19-9 (range 45-15000). In 11 patients (group A) postoperative Ca 19-9 fell down in the normal range and 11 patients had postoperative Ca 19-9 levels above the normal range in the whole follow up period (group B). In group B patients all died within 12 months while in group A after 12 months all were alive ($p < 0.0001$).
Serum Ca 19-9 levels raised again 1 to 7 months before progression of disease was clinically detectable. No significant reduction in Ca 19-9 levels was seen after palliative surgery.
Ca 19-9 is a reliable prognostic marker after resective surgery and is useful in showing recurrence of cancer after resection while not yet clinically evident.

Study supported by CNR grant. Project "Oncology" # 88-00804.44

### FP052 INSULIN SENSITIVITY AND GLUCOSE TOLERANCE AFTER SUBTOTAL PANCREATECTOMY

K.-G. Tranberg, S. Bengmark, J. Magnusson.
Department of Surgery, Lund University,
Lund, Sweden.

It is unclear if the sensitivity to insulin is preserved or altered after pancreatectomy. Thus, pancreatectomized patients have been shown to have both increased and decreased sensitivity to insulin as compared with type I diabetics and to be insulin resistant as compared with normal subjects (Nosadini et al; Yki-Järvinen et al.). The aim of the present study was to determine insulin secretion, insulin sensitivity and glucose tolerance together with changes in plasma glucagon levels shortly after subtotal pancreatic resection.

Twelve consecutive, non-diabetic patients with carcinoma of the pancreatic head were studied after potentially curative surgery. The distal pancreas was stapled off, without pancreato-jejunal anastomosis, leaving about 15 % of the pancreas in situ. Brief infusions of insulin (10 mU/kg) and glucose (25 g) were given before and 4 days after operation.

Post-operatively, fasting levels of blood glucose, C-peptide and plasma insulin remained unchanged, whereas fasting levels of pancreatic glucagon were decreased. The insulin response to glucose was severely reduced. The hypoglycemic action of insulin as well as glucose tolerance was similar to that observed before operation, which contrasted with the insulin resistance and glucose intolerance observed after pancreas-preserving intraabdominal procedures of similar size (Magnusson et al.).

It is concluded that an acute reduction in pancreatic mass preserves insulin action and glucose tolerance shortly after surgery. It is suggested that the decrease in glucagon levels is at least partly responsible for the preservation of insulin action after subtotal pancreatectomy.

References:

Nosadini et al. Diabetes 1982; 31: 346.

Yki-Järvinen et al. Metabolism 1986; 35: 718.

Magnusson et al. Scand J Gastroenterol 1989; 24: 539.

### FP053 COMBINATION OF INTRAOPERATIVE RADIATION WITH RESECTION OF CANCER OF THE PANCREAS

T.Hiraoka, R.Uchino R, K.Kanemitsu, I.Kamimoto, N.Saitoh, C.Takada, K.Takamori, S.Tashiro, Y.Miyauchi
Kumamoto Univ. Med. Sch., Kumamoto, Japan

The utility of intraoperative radiation therapy (IORT) as an adjuvant to the surgical resection of pancreatic cancer was studied.

Since 1976, as our first trial with this combined therapy, we have applied IORT with 30 Gy of electron beam with 8 meV to 15 patients to prevent local recurrence around the celiac axis and superior mesenteric artery after standard pancreatectomy. However, the combined therapy did not show improvement in survival rate comparing to 19 patients with standard operation alone. Autopsy of 3 patients with the combiend therapy did not show involved lymph nodes in the radiation field, but showed local recurrence around the aorta outside the radiation field.

By comparison, we performed extended operation without IORT on 9 patients with almost complete dissection of the lymph nodes around the aorta from diaphragm to the level of the inferior mesenteric artery. This extended surgery did not imporve survival time, and autopsy showed local recurrence in spite of dissection of lymph nodes.

Therefore, since 1984, we have performed IORT with a dose of 30 Gy, 9 meV and extended radiation field from diaphragm above to the inferior mesenteric artery below, following extended operation on 14 patients. Five year cumulative survival rate of these cases was 33.3%. Four autopsies showed improvement of local control rate.

We were encouraged to continue this approach for the cure of pancreatic cancer.

### FP054 RESULTS OF DIFFERENT TREATMENT MODALITIES IN NON-RESECTABLE PANCREATIC CARCINOMA

U. Sulkowski, J. Meyer, H. Bünte
Department of General Surgery, Münster
University Hospitals, Münster, FRG

During the last 14 years 512 patients with carcinoma of the exocrine pancreas were treated at the Department of General Surgery of Münster University Hospitals. Only 61 could be resected with curative intention (11.9%); 451 were only treated palliatively. In our series three different treatment modalities were used: 1. Surgical palliation (1974-1982;n=22o) 2. Endoscopic palliation (1983-1985;n=132) 3. Multidisciplinary palliation (1986/87;n=99). The period of surgical palliation with biliary drainage procedures like choledochojejunostomy being the most frequent operations (n=98), is characterized by a high perioperative mortality (8.5%). The median survival time was 4.5 months. During the period of endoscopic palliation the "perioperative" mortality was reduced to 4.5%; the median survival time increased to 6.2 months. With the introduction of a multidisciplinary concept (surgery, endoscopy, chemotherapy), 35 patients herein underwent polychemotherapy according to the EAP-scheme (5 partial remissions), no further improvement could be observed (mortality 3.o%, median survival time 6.o months). However, in-hospital stay was significantly prolonged. According to our results we recommend endoscopic palliation as the treatment of choice in non-resectable pancreatic carcinoma. Other regimen like chemo-, immuno- or radiotherapy should only be used in prospective and controlled clinical trials.

**FP055** RADICAL RESECTION FOR AMPULLARY CANCER REMAINS THE BEST OPTION FOR LONG TERM SURVIVAL

JRT Monson, GP McEntee, JH Donohue, R Shorter,
DC McIlrath, JA van Heerden, DM Nagorney,
Mayo Clinic and Foundation, Rochester, MN 55905, USA.

Patients with ampullary cancer are frequently elderly, frail and debilitated. Radical resection should be undertaken only when a low operative mortality and a reasonable long term survival rate can be anticipated. To evaluate our results, 104 consecutive radical resections performed between 1965-1989 for ampullary cancer were retrospectively reviewed. **Materials:** There were 59 men and 45 women with a mean age of 64 years (range 35-85). The mean duration of symptoms was 7.8 weeks. 32.7% of patients were not jaundiced, while 26.9% of patients were anemic. 42.3% reported significant weight loss. Eighty seven (83.7%) patients underwent pancreaticoduodenectomy (PD), while 17 (16.3%) underwent total pancreatectomy. **Results:** The postoperative mortality was 4.8% (five patients), and reoperation for immediate post-operative complications was required in 5 patients. The 3 and 5 year survival rates were 46.9% and 30.3%, and 8 patients died of tumor beyond 5 years. Only one of 17 (6%) patients remains alive following total pancreatectomy although an additional 4 survived more than 4 years. In contrast, 38 of 87 (43.7%) patients remain alive following a PD. Of these, 27 are tumor free between 4 and 21 years following surgery. **Conclusion:** Radical resection for ampullary cancer can be performed with an acceptable morbidity and mortality. Since the long term survival following PD may be superior to total pancreatectomy, this should remain the procedure of choice for ampullary carcinoma.

### FP056 LOCAL RESECTION FOR CARCINOMA OF AMPULLA OF VATER

J Hollinshead, M Jones
Concord Hospital, Sydney, Australia

Carcinoma of the Ampulla of Vater is a relatively uncommon cause of obstructive jaundice. The most efficacious treatment is pancreaticoduodenectomy. In elderly ill patients considered most unlikley to survive pancreaticoduodenal resection, the lesser procedure of local resection offers a possible alternative.

Eight elderly, poor surgical risk patients (mean age 71 years) presenting with obstructive jaundice were diagnosed on endoscopy as having localised ampullary tumour which were removed by local resection.
The 30 day operative mortality was nil, however, one patient died five months post-operatively without leaving hospital as the result of septic complications. There were no other significant complications in the remaining seven patients. Histopathology showed 5 patients had well differentiated papillary carcinoma, 2 had invasive adenocarcinoma and 1 squamous cell carcinoma. The outcome of the five patients with papillary adenocarcinoma has shown three are alive and well, 58, 34 and 15 months post-operatively; one patient died at 22 months of cardiac problems free of tumour while the final patient died at 17 months from widespread tumour. Of the three patients with non-papillary tumour, one died in hospital (above) while the other two died of widespread tumour at 6 and 12 months. None of the 8 patients have been troubled by the recurrence of obstructive jaundice even though the line of resection was involved with tumour in three patients.

Local resection of the Ampulla of Vater has a low operative morbidity and mortality and offers excellent palliation of obstructive jaundice for patients with tumours localised to the ampulla. In patients with papillary adenocarcinoma of the ampulla, there is the potential for long term palliation and possible cure.

FP057 THE INCIDENCE OF ACUTE MUCOSAL GASTROINTESTINAL LESIONS IN PORTAL HYPERTENSION

I.F.S.F. Boin; L.S. Leonardi; F. Callejas Neto; E.A.Chaim

Unicamp Medical School-Campinas-SP,Brazil.

The authors present the incidence of acute mucosal gastrointestinal lesions (AMGL) in a total of 215 patients with portal hypertension, 102 with schistossomiasis in the liver (S) and 113 cirrhotics (CI).

All patients were admitted for endoscopic control of varices oesophageal gastrics and were grouped following Child's Classification:

S = A-81; B-21; C-0 (n= 102)

CI = A-44; B-57; C-12 (n= 113)

The mean age of "S" patients is 40,6 years old and of "CI" patients is 47,3 years old, with male predominance in both groups.

The observed incidence of AMGL is greater in the "CI" group ($p \angle 0{,}001$). In this group the observed incidences is not statiscally different ($p \angle 0{,}05$) between the "A" patients and the "B" + "C" patients.

References:

McCormack TT et al. Gut 1985; 26(11):1226.

Nagamine K et al. Jpan J Surg 1986; 16(3):218.

### FP058 MORPHOLOGIC AND FUNCTIONAL CHANGES IN THE INTESTINAL MUCOSA IN THE PORTA-CAVAL SHUNTED RAT IS COUNTERACTED BY MESENTERIC VEIN STENOSIS

P. Sharma, F. Bengtsson, M. Bugge, K. Johansen, B. Weström, S. Lundin, B. Jeppsson. Department of Surgery, Clinical Pharmacology, Lund University, Sweden

Histologic simplification of the small bowel mucosa has been reported after portacaval shunt (PCS) in the rat. This may have implications for the metabolic pertubation and encephalopathy seen after PCS. We, therefore, studied morphology and intestinal absorption of the mucosa in PCS rats. Since PCS + mesenteric vein stenosis (PCS-MVS) has been shown to lower the elevated plasma ammonia levels after PCS, we also studies similarly in PCS-MVS rats.

Method 8 end-to-side PCS, 8 PCS-MVS (ext diameter 0.96 mm) and 9 sham-operated rats were used. 6 weeks postop two mucosal absorption marker molecules, 1-deamino-cysteine-8-D-arginine vasopressin (dDAVP;mw 1000) and FITC-Dextran (mw 70 000) were administered by gastric intubation. Blood samples were taken 1 and 4 hours later for analysis of marker. Proximal and distal small bowel and colon were taken for histology.

Results

| Bowel histology | Sham | PCS | PCS-MVS |
|---|---|---|---|
| Prox intest. | normal | complete loss of surface epithelium | normal |
| Dist intest. | " | " " " | " |
| Colon | " | normal | " |

Both dDAVP and FITC-Dextran occurred in lower conc in plasma in PCS compared to sham and PCS-MVS. There was no difference in plasma absorption marker conc between sham and PCS-MVS.

Discussion and conclusion PCS in the rat is accompanied by a complete loss of small bowel mucosa surface epithelium without signs of inflammatory reaction or changes in the remaining mucosa. This mucosal damage was absent when a mesenteric vein stenosis was added to the PCS. The changes of the small bowel mucosa in PCS rats were associated with a loss in absorptive capacity of macro-molecules (mw 1000-70 000). The changes may have patho-genetic implications in PCS-encephalopathy and be of therapeutic importance in shunt-surgery.

### FP059 NONINVASIVE DIAGNOSIS OF PORTAL SYSTEM THROMBOSIS

M.Elwertowski, K.Zieniewicz, M.Busz

J.Pawlak, B.Michalowicz, P.Malkowski

Institute of Surgery Warsaw POLAND

The aim of our study was to evaluate utility of complex CT & US Doppler evaluation of patients with portal hypertension. Out of 119 patients with portal hypertension & esophageal varices diagnostic modalities revealed portal system thrombosis in 53 cases. Ultrasonic studies were performed with Hitachi EUB 40 & Toshiba 270a ( colour Doppler ), CT ( dynamic studies ) with Somatom DRH 2000. Our results were confirmed by DSA, operations and autopsies.

Out of 25 patients with complete portal vein thrombosis US diagnosed properly 22 ( CT - 26 with 2 false positive errors ). Cavernous transformation of portal vein present in 9 patients was properly diagnosed in US in 8 cases ( CT - 8, with one error ). Partial thrombosis ( or perimural thrombus ) found in 15 patients was correctly diagnosed in US in 10 cases, while CT demonstrated 14 of them. Reversed flow in portal vein present in 4 patients was demonstrated correctly in US in all cases while in only 1 case in CT.

Incorporation of colour Doppler helped to eliminate most of doubtful results in ultrasonic study as well as facilitated diagnosis of reversed flow in portal vessels. Ultrasonography used as an initial diagnostic study helped to incorporate the most adequate mode of dynamic CT vascular studies. CT proved to be superior in cases of partial thrombosis.

### FP060 THE ENDOSCOPIC TREATMENT OF BLEEDING GASTRIC VARICES

G. E. Gerunda, F. Zangrandi, D. Neri,
M. Bisello, F. Barbazza, F. Bedendo,
A. Maffei-Faccioli. Institute of Second
Surgical Pathology, University of Padua,
Italy

INTRODUCTION: Bleeding from gastric varices is a rare and serious condition because of treatment difficulties and high mortality.

PATIENTS AND METHODS: 34 patients with bleeding gastric varices were admitted to our Institution in emergency conditions. Mean age was 53.4+/- 2.1, mean Child-Pugh score was 9.1+/-2.1 (8-13). The etiology of liver disease was alcoholic in 53% of patients. All of them underwent an emergency endoscopic sclerotherapy; in 13 patients (39%) the liquid used was Polidocanol 2% (intra and peri-varicose), in 8 (22%) saline solution (40-60 ml perivaricose) and Polidocanol 2% (about 5ml intravaricose), in 13 (39%) a solution (about 4 ml) of N-Butil-2-Cyanoacrylate (Bucrilate) and Ultrafluid Lipiodol (ratio 1:1).

RESULTS: Cessation of bleeding was observed early in 67% of patients: in particular in 38% of group 1 (3 patients out of 8 continued bleeding and underwent emergency operations, 5 were successfully treated with balloon tamponade). In 62% of group 2 (out of 3, one underwent an emergency operation and 2 were treated with balloon tamponade). In 100% of group 3 (1o vs 3o $P<0.05$). The 30 days mortality of the three groups were 47%, 38%, 0% respectively (1o vs 3o $P<0.02$). No different morbility was observed in the three groups. After 12.3+/-4.9 months of follow-up, of the 21 survivors only 1 patient (in Group 3) was operated on. No gastric variceal rebleeding was observed in the other 20 patients.

CONCLUSIONS: Rapid cessation of bleeding from gastric varices without significant morbility may be obtained with Bucrilate. However, it has to be strongly underlined that Bucrilate is a liquid that is very difficult to manage and dangerous for endoscopic equipment.

### FP061 A PROSPECTIVE RANDOMISED CONTROLLED TRIAL COMPARING SOMATOSTATIN AND INJECTION SCLEROTHERAPY IN THE CONTROL OF ACUTE VARICEAL HAEMORRHAGE

S A Jenkins, J N Baxter, S Ellenbogen
C Makin, A Kingsnorth, I Gilmore
A Morris & R Shields
University of Liverpool, Liverpool, UK

Previous reports have suggested that somatostatin (SRIF) may be of value in the control of bleeding from oesophageal varices. Therefore, the aim of this study was to compare the efficacy of SRIF with injection sclerotherapy in the control of acute variceal haemorrhage. Eighty consecutive patients admitted with endoscopically proven, severe variceal haemorrhage were randomised to either emergency sclerotherapy or SRIF (bolus dose of 250 µg followed by a continuous infusion of 250 µg/h for 5 days). The aetiology of the portal hypertension, classification according to Child's criteria and blood transfusion requirements were similar between the two groups of patients. Forty-one patients were randomised to sclerotherapy and 39 to SRIF. Initial control of bleeding was achieved in 40 of the 41 patients receiving sclerotherapy but rebleeding occurred within the 5 day period in 6 patients. Overall control of bleeding was achieved in 30 of the 39 patients randomised to SRIF. There was no significant difference ($p = 0.58$; Fisher's exact test) between the two forms of treatment. Furthermore, somatostatin was equally as effective as sclerotherapy in controlling variceal bleeding in patients with severe liver dysfunction (Child's C) or those actively bleeding at the time of their initial endoscopy. The hospital mortality was similar between the two groups of patients. The results of this trial suggest that somatostatin infusion may be as effective as sclerotherapy in the control of acute variceal haemorrhage.

FP062 **Surgery For Acute Variceal Haemorrhage - Survival After Oesophageal Transection For Failed Injection Sclerotherapy**

P.D.Willson R.Kunkler S.D.Blair

K.W.Reynolds

Surgical Gastrointestinal Unit, Charing Cross Hospital, London W6.

Continued haemorrhage from oesophageal varices, despite adequate injection sclerotherapy and tamponade has a mortality of approximately 90%. Between 1978 and 1989 thirty patients with acute variceal haemorrhage uncontrolled by initial treatment underwent early emergency oesophageal transection.

Mean age was 50 years (range 21-70). Male:Female ratio was 21:9. In 70% portal hypertension was due to alcoholic cirrhosis. Other cirrhoses accounted for 27% and portal vein thrombosis 3%. Immediate preoperative Pughs grading were A;2, B;6 and C;22 patients. Downgrading between admission and transection from A to B occured in 1 patient and from B to C in 5 patients.

Oesophageal transection stopped variceal haemorrhage in 29/30 patients. Rebleeding within thirty days of surgery occured in 5 patients from gastric varices. Post operative bleeding also occured from perioesophageal vessels (2), gastrotomy (1), and an oesophageal ulcer caused by prolonged tamponade (1). 7 patients developed post operative hepatic failure, 5 renal failure and 4 both hepatic and renal failure.

Overall there was a 37% thirty day survival folowing oesophageal transection. This was directly related to the preoperative Pugh grade. Survivors were 2/2 grade A, 5/6 grade B and 4/22 grade C. Three month survival for grade B patients dropped to 2/6 whilst remaining unchanged for other grades. Oesophageal transection is effective at stopping variceal bleeding and gives reasonable early survival related to grade, but it does not remove the underlying disease and therefore late mortality following transection is disappointing in all but Grade A patients.

### FP063 GASTRO OESOPHAGEAL STAPLING WITH TRANSABDOMINAL PORTO AZYGOS DISCONNECTION IN THE MANAGEMENT OF ACTIVE VARICEAL BLEEDING

**S.K. MATHUR, A.N. NALVI, D.F. MIRZA, B.A. KWARNI, S.B. SALNIS, R. GONDHLEKAR Dept. of Surgery, G.S. Medical College & K.E.M. Hospital, Bombay, INDIA.**

A Variety of procedures have been described to control the bleeding from Gastro-oesophageal varices in emergency. Endoscopic variceal sclerotherapy (EST) has been show to be most popular and effective method. However patients who continue to bleed inspite of EST require emergency surgery. We are presenting our experience of control of variceal bleeding with Gastro-oesophageal Stapling and Transabdominal porto-azygos disconnection in patient where EST has failed. The study include 28 patients of portal hypertension with age ranging from 6 to 63 years. The etiology of portal hypertension was cirrhosis in 11 (Child A-1, B-2 and C-8) patients, extrahepatic portal venous obstruction in 10 and non cirrhotic portal fibrosis in 7 patients. Operative procedure included ligation of splenic artery without splenectomy in 17 cases and splenectomy in 5 cases along with devascularisation of upper half of stomach and lawer 7-10 cm of oesophagus in all cases. A transgastric oesophageal transection was done in 14 cases using E.E.A. stapler and subcardiac gastric stapling was done in 10 cases using SGIA stapler and combination in 4 cases. Variceal bleeding could be controlled in all cases. 4 patients developed post operative complications (oesophaged leak-3 and stricture-1). Postoperative mortality was 28.5 % (8/28). During follow up ranging from varices, however two patients had bleeding from congestive gastropathy. 4 patients (20 %) developed recurrent oesophageal varices gr I at 6 and 12 months. In conclusion, gastro-oesophageal stapling with transabdominal porto-azygos disconnection is an effective procedure to control active variceal bleeding in patients with failure of sclerotherapy with low risk of rebleeding on long term follow up.

### FP064 RANDOMIZED TRIAL COMPARING DISTAL SPLENORENAL SHUNT AND SCLEROTHERAPY IN THE PREVENTION OF VARICEAL REBLEEDING

G.P.Spina, R.Santambrogio, E.Opocher, F.Cosentino, C.Grugni, G.Rubis Passoni, S.Bruno, A.Zambelli, San Paolo Institute of Biomedical Science - University of Milan, Italy

In 1984 we initiated a prospective controlled trial comparing endoscopic sclerotherapy (ES) with the distal spleno-renal shunt (DSRS) in the elective treatment of variceal hemorrhage in cirrhotic patients. The study population comprised 66 patients with cirrhosis and portal hypertension referred from October 1984 to March 1988 to our Department. These patients were drawn from a pool of 239 patients who were electively treated for portal hypertension by surgery or endoscopic sclerotherapy during this interval. Patients were assigned to one of the 2 groups according to a random number table: 34 to DSRS and 32 to ES.

In ES group, varices were completely eradicated in 25 patients. During the postoperative period, no DSRS patient died, while 1 ES patient died of uncontrolled hemorrhage after the first sclerosis session. One DSRS patient had mild recurrent variceal hemorrhage and had patent DSRS by angiography. Five ES patients suffered at least one episode of gastrointestinal bleeding: 2 from varices and 3 from esophageal ulcerations. No patient had episodes of hepatic encephalopathy. Seven ES patients developed transitory dysphagia. Long-term follow-up was complete in 100% of patients. However, in ES group, 1 patient was successfully submitted to liver transplantation because of liver failure. Four-year survival rates for shunt (79%) and ES (65%) groups were similar. One DSRS patient rebled from duodenal ulcer, while two ES patients rebled from varices and three from hypertensive gastropathy. Two DSRS and 2 ES patients have evolved a mild chronic encephalopathy; 5 DSRS and 3 ES patients suffered at least one episode of acute encephalopathy. Four ES patients had esophageal stenosis that were successfully dilated.

Preliminary data from this trial seem to indicate that DSRS with a correct portal-azygos disconnection in a subgroup of patients with good liver function is more effective in the prevention of variceal rebleeding than ES, but, unfortunately, without any improvement in patients' survival.

FP065 DISTAL SPLENORENAL SHUNT (DSRS): 10 YEAR EXPERIENCE

A. Maffei-Faccioli, G.E. Gerunda
Institute of Second Surgical Pathology,
University of Padua, Italy

INTRODUCTION: The most important criticism to be found with Warren's operation is the loss of shunt selectivity over the time resulting in marked decrease of hepatopetal flow. To prevent this effect the original technique has been modified with the introduction of splenopancreatic disconnection (SPD). The aim of this study was the analysis of our experience in DSRD over a 10-year period.

MATERIALS: Seventy patients, selected from 265 with proved variceal bleeding, underwent DSRS with or without SPD. The alcoholic cirrhosis was the cause of portal hypertension in 57%. The operative mortality was 12.7% (Child A+B 1.7%, C 66%). The mean follow-up time was 46.1+/-31.6 months (range 2-120).

RESULTS: A definite decrease of varices was observed in all patients, and only one, mortal, variceal rebleeding (1.6%) occurred). A late perfusion was observed in 91% of our series, a worsening, compared to preoperative phase, in 23%. The persistent hepatic cytolysis and incomplete SPD were the msot significant prognostic factors for a worse perfusion (38.1 vs 11.5% and 42.8 vs 11.5% for presence/absence of cytolysis and DSRS/DSRS-SPD, respectively). SPD was also able to decrease the negative effect of cytolysis. The Post Shunt Encephalopathy was clinically present in 6.5%, but after EEG evaluation it increased to 24.6% with a significant influence of worsening of perfusion 61.6 vs 13.9 - P<0.01), active cytolysis (47.6 vs 17.2 - P<0.02) and incomplete SPD (42.8 vs 14.3 - P<0.05). The overall 5-year survival was 57%, but a higher late liver-related mortality was observed in patients with a lack or worsening of perfusion and without SPD.

CONCLUSIONS: DSRS-SPD today still holds a fundamental role, even if surely more restricted than before, in the therapeutic baggage that a Centre specializing in this field must be able to offer.

FP066 SUGIURA PROCEDURE VS. PORTACAVAL SHUNT IN PREVENTING RECURRENT VARICEAL BLEEDING. INTERMEDIATE RESULTS OF A PROSPECTIVE RANDOMIZED STUDY.

C SMADJA, G. BORGONOVO, F. KAHWAJI, D. GRANGE, D. FRANCO. Groupe de Recherche sur la Chirurgie du Foie et de l'Hypertension Portale, Hôpitaux Bicêtre, Le Kremlin Bicêtre, Louise Miche, Evry, Paul Brousse, Villejuif, France.

The purpose of this work was to compare the results of the Sugiura procedure and of portacaval shunt for the prevention of recurrent variceal bleeding in cirrhotic patients. Between April 1984 and May 1989, 54 cirrhotic patients with a recent history of variceal bleeding were randomly assigned to Sugiura procedure (26) or portacaval shunt (28). The Sugiura procedure included all steps described by Sugiura but was performed by a single abdominal approach. Esophageal transection was done by mechanical stapling. There were 23 side-to-side portacaval shunts and 5 mesocaval shunts using a 18 mm diameter dacron prosthesis. Alcoholism (96 %) was the commonest cause of cirrhosis. In 21 patients (39 %) surgery was performed after failure of previous esophageal sclerotherapy and/or of medical treatment. Randomization was performed the day before surgery, 2 to 8 weeks after the last bleeding episode. Cumulative risks of recurrent bleeding and encephalopathy, and survival were calculated according to Kaplan-Meier. Comparisons between the two groups were done by the log-Rank test. The cumulative risk of recurrent variceal bleeding was not different after Sugiura procedure (9 %) than after portacaval shunt (3 %). Encephalopathy was significantly more frequent after portacaval shunt (33 %) than after Sugiura procedure (9 %; $p < 0.05$). Two-year survival was better after Sugiura procedure (91.2 %) than after portacaval shunt (75.8 %). This difference however was not significant. These intermediate results suggest that the Sugiura procedure is as efficient in preventing variceal bleeding as portacaval shunt and results in significantly less encephalopathy. It may also be associated with better survival.

### FP067 COMPLICATIONS OF THE AZIGO-PORTAL DISCONNECTION AND SPLENECTOMY ASSOCIATED WITH SCLEROTHERAPY IN SCHISTOSSOMIASIS

L.S. Leonardi; I.F.S.F. Boin; F. Callejas Neto; E.A. Chaim
Unicamp Medical School-Campinas-SP,Brazil

The authors, to improve the results of Portal Hypertension surgery in schistossomotic patients, analysed a group of 19 patients, during the period from 1981 to 1989.

The imediate complications were disphagia in 4 patients (20,8%), epigastric fullness in 2 (10,4%), gastric fistula in 2 (10,4%), urinary infection in 2 (10,4%), bronchopulmonary complications in 2 (10,4%), wound infection in 4 (20,3%), postoperative fever in 3 (15,6%) and diarrhea in 1 patient (5,2%).

Late complications were hepatic insufficiency in 1 patient (5,2%) and bleeding recurrence in 3 patients (15,6%) at the 1st month, 2 years and 5 years, respectively.

Two patients died, after hepatic insufficiency and other after duodenal fistula (pyloromiotomy dehiscence) with a mortality rate of 10,4%.

In conclusion, out the high rate of general complications, the bleeding recurrence in 15,6% of the patients and ascite in 10,4%, it was not observed any case of hepatic encephalopaty.

References:

Chung RS, Camara DS. Am J Surg 1984; 148:389.

Sugiura M, Futagawa S. Arch Surg 1977; 112:1317.

**FP068** A NEW TREATMENT MODALITY FOR BUDD-CHIARI SYNDROME: EXPANDABLE VENOUS STENTS

R.R. Lopez, C.W. Pinson, K.G. Benner,

L. Hall, J. Rosch

Oregon Health Sciences University

Portland, Oregon, USA

The goals of treatment of Budd-Chiari Syndrome are relief of portal hypertension, relief of inferior vena cava syndrome, if present, and preservation of hepatic function. Traditional portocaval or mesoatrial bypass shunts have offered some success, but with high rates of complication. Venous balloon angioplasty has recently been reported, but the incidence of restenosis is high.

A 26-year-old Middle Eastern male with severe ascites and lower extremity edema but with preserved hepatic function had: a suprahepatic caval web (15 torr gradient); an intrahepatic caval stenosis (27 torr gradient); occluded right and middle hepatic veins; and stenosis of the left hepatic vein (24 torr gradient). Under angiographic control, web and caval stenosis were balloon dilated and modified Gianturco expandable metallic stents were placed. The left hepatic vein was dilated twice and stents placed. The gradients were reduced 50%, 100% and 75% respectively. There were no complications and the stents have continued to expand without migration over 6 months. Edema and ascites resolved, hepatic function remains good, and the patient has returned to work.

This technique of management may provide a new, safe, effective and relatively inexpensive form of treatment for selected patients with Budd-Chiari Syndrome.

### FP069 A STUDY OF CARCINOEMBRYONIC ANTIGEN IN BILE DUCT CANCER AND INTRAHEPATIC DUCT STONES

C-G Ker,J-S Chen,K-T Lee,P-C Sheen
Division of Hepatobiliary Surgery
Kaohsiung Medical College Hospital, Taiwan, ROC

Bile duct neoplasm was frequently occurred in the patients of hepatolithiasis in Taiwan. Thirty-nine patients were consisted of 16 patients of hepatolithiasis, 7 patients of hepatolithiasis with bile duct carcinoma and 11 bile duct carcinoma were studied for assay the concentration CEA in bile and demonstration of CEA in the bile duct. Another 15 patients of gall bladder stones were subjected as control. Those patients were studied with immunoperioxidase stain of CEA in bile ductal glands with relation of the concentration of CEA in serum and bile.

The bile concentration of CEA in control group (n=5) hepatolithiasis (n=6), hepatolithiasis with bile duct carcinoma (n=7) and bile duct carcinoma (n=11) were 4.09 ± 4.12, 70.49 ± 81.62, 137.73 ± 66.15, and 144.31 ± 117.31 ng/ml (M ± SD) respectively. The demonstration of positive CEA stain on the bile duct in each group were 13.33%, 81.23%, 85.71% and 90.19% respectively. No strong positive (2+ or 3+) could be found in control group. But strong reaction was frequently found in hepatolithiasis and bile duct carcinoma with the positive rate of 50% (8/16) and 82% (9/11) respectively.

Therefore, we strongly suggest that the probability of combined neoplasm was increased if the CEA value was unusually elevated in the patient of hepatolithiasis.

### FP070 DIAGNOSIS OF HEPATOCELLULAR CARCINOMAS USING ENHANCED ULTRASOUND AND PATHOLOGICAL INVESTIGATION

M.Nakagawa, K.Takasaki, S.Kobayashi, F.Hanyu,
Tokyo Women's Medical college, TOKYO, JAPAN.

Up to the present time, Ultrasound is the most useful and important means of diagnosing liver tumors. However, it cannot detect very small liver nodules. Recentry we have been working with a new method of enhanced Ultrasound. This involves injecting $CO_2$gas into the hepatic artery. By this safe and easy method which needs no pretreatment, diagnosis is improved and more information is obtained. First, we want to show the diagnostic ability of enhanced Ultrasound and to compare it with plain Ultrasound and CT. This investigation was carried out using 71 recognized nodules from resected liver specimens. Out of 43 nodules under 2cm.using plain Ultrasound, only 23 were detected, using CT only 6, but using enhanced Ultrasound, all 43 were detected.

Next, we will compare enhanced Ultrasound findings with histopathological findings.

Hepatic tumors are classified by enhanced Ultrasound into 2 categories - positive and negative enhanced. The present study examined 46 nodules resected from 32 patients. Of these, 33 nodules were positive enhanced and 13 were negative enhanced.

The histopathological examination of these two groupes of resected specimens revealed that the nodules of the positive enhancement type were macroscopically a yellow-white tone, and mostly of nodular type. Pathologically, they demonstrated expansive growth, septum formation, diffective Glisson's sheaths and pronouned cellular atypia, and were largely of the thick trabecullar type. Nodules of the negative enhancement type were macroscopically of the massive type, brown-toned and not encapsuled. Pathologically, they were of the thin trabecullar type with replacment growth, presenting no septum formation in the tumors, with intact Glisson's sheaths and less pronounced cellular atypia.

Negative enhanced nodules were well differentiated and probably show the early stages of hepatocellular carcinomas.

FP071

### ULTRASOUND-GUIDED FINE NEEDLE ASPIRATION CYTOLOGY OF THE LIVER, BILIARY TRACT AND PANCREAS

M. Gilg, H.U. Baer, J. Scheidegger, H. Kraft, L.H. Blumgart
Department of Visceral Surgery, Diagnostic Radiology and Pathology, Inselspital, Berne, Switzerland.

Preoperative diagnosis using ultrasound guided fine needle aspiration cytology was prospectively evaluated in 59 patients with possibly cancerous lesions of the liver, biliary tract or pancreas. 21 patients with mass lesions of the pancreas and 33 with lesions of the liver were studied. 5 patients with lesions of the extra hepatic biliary tract were also examined.

In all 59 patients the mass could be visualized on ultrasound and punctured without any complications.

In 43 of the 59 specimens a positive result was found and was in all instances confirmed by definitive histology. There were no false positives. In 14 instances a negative result for carcinoma was confirmed at final histology. There were 2 false negative tests.

It is concluded that preoperative fine needle aspiration cytology guided by ultrasound is reliable in the investigation of carcinoma of the liver, biliary tract and pancreas provided that the mass can be well visualized by ultrasound. In our study the sensitivity was 95%, specificity 100% and accuracy 96%. Because there were no false positive results the method can be regarded as a reliable index of the presence of tumor and is thus of value in preoperative assessment and in treatment planning for patients with suspected malignancy.

FP072

### CYTOLOGICAL DIAGNOSIS OF EXTRAHEPATIC BILIARY STRICTURES

LA Desa, AB Akosa, S Lazzara, P Domizio,
T Krausz, IS Benjamin
HPB Surgery Unit, RPMS, Hammersmith Hospital,
London, England

One hundred and seventeen patients who presented to the Hammersmith Hospital with extrahepatic biliary strictures over a nine year period had 206 cytological examinations of the bile duct or bile (153 non-operative, 53 intra-operative), to establish the presence of malignancy. A final diagnosis of cholangiocarcinoma was made in 88 patients - on histological grounds in 38 patients and on the results of investigations and the subsequent clinical course in the remaining 50 patients. Twenty-nine patients had benign biliary strictures. The cytological techniques employed were fine needle aspiration of the bile duct (FNA, n = 102), or brushing (n = 24) of the bile duct, or exfoliative cytology of bile (n = 80). Forty-one patients with malignancy had two or more examinations with differing cytological results between samples in 20 cases.

The overall sensitivity was 72%. There was only one false positive result, giving a patient predictive value of positive cytology of 98%, and a predictive value of negative cytology of 53%. Intra-operative cytology was more sensitive than pre-operative examination (80% of all results versus 42%). Overall the sensitivity of FNA (67%) was greater than that of brush cytology (40%). Exfoliative cytology had the least sensitivity (30%), because of the high percentage of unsatisfactory samples obtained.

We feel that cytological confirmation of cholangiocarcinoma should be sought in all suspicious cases of extrahepatic biliary stricture. A positive diagnosis not only aids in the selection of further relevant investigation for resectability, but also justifies palliative stenting in cases deemed irresectable.

FP073 TRANSCYSTIC CHOLEDOSCOPY

P. Chiotasso, M. Lallement, C.H.U. Toulouse,
France.

The calibre of new optic fibers is now sufficiently thin to allow their insertion through the cystic duct into the choledocus without openning the common bile duct.
The purpose of this prospective study was to control that transcystic choledoscopy is technically possible and to appreciate the preliminary results.
It was used routinely after a peroperative cholangiography, in order to compare their respective results.
Since october 89 to january 90, 15 consecutive cholecystectomies for lithiasis were included in the study. They were 12 females and 3 males, mean age being 62.6 years. 7 of 15 cases had a biliary retention define as an elevation of alkaline phosphatases and or bilirubine.
In 14 of 15 cases, the insertion of the fiber through the cystic duct was possible; once, the technique was abandonned relate to a large oedematous hepatic pedicle due to a pancreatitis. 6 times, the cystic duct was shortened to allow a better insertion of the fiber.
There was no complication in the postoperative course.
In 3 cases the peroperative cholangiography shown a common bile duct stone confirmed by the transcystic endoscopy.
In 3 other cases there was a doubt on the cholangiography of which 1 patient had a common bile duct stone detected by the transcystic examination, giving a total of 4 stones found in this series.
On the other hand, 3 of the 7 biliary retentionnal syndromes were cleared by the transcystic endoscopy.
We conclude that this method is technically possible and could take the place of peroperative cholangiography.

### FP074 THE DIAGNOSTIC VALUE OF INTRAOPERATIVELY ULTRASOUND MEASURED COMMON BILE DUCT DIAMETER

J.J.Jakimowicz, H.Rutten, Catharina Hospital Eindhoven,

E.J.Carol, Carolus Hopsital, 's Hertogenbosch.

Presence of the common bile duct dilatation found at surgery (diameter > 12 mm) belongs to the so called "absolute" indications for common bile duct (CBD) exploration. In course of prospective study evaluating intraoperative sonography and intraoperative cholangiography the inside diameter of the common bile duct was routinely measured, registered and the data computerized. Data from 810 patients were available for evaluation and were compared with an outcome of the other diagnostic procedures as intraoperative Sonography and Cholangiography and finally with the operative findings. Statistical evaluation: There is a statisticly significant difference in the duct diameter P < 0,001 between the two groups of patients with and without CBD pathology. The outcome of the statistical evaluation for different diameter is as follows.

| | ≤4 mm - >4 mm | ≤5 mm - >5 mm | ≤6 mm - >6 mm |
|---|---|---|---|
| Predictive value neg.test | 99% | 98,5% | 96% |
| specificity | 71% | 85% | 95% |
| Predictive value pos.test | 50% | 66% | 84% |
| sensitivity | 98% | 96% | 87% |

Relation between the CBD diameter and the pressure value measured by means of Micro Transducer Tip catheter in a seperate group of patients are discussed.
Concluding: Intraoperatively Ultrasound measured diameter of the common bile duct is significantly higher in patients with CBD pathology. If the inside diameter of the duct is smaller or equal to 5 mm the common bile duct pathology is rarely encountered. In patients with a CBD diameter larger than 5 mm and no signs of pathology at the intraoperative Sonography intraoperative cholangiography is indicated. Intraoperatively measured Ultrasound diameter is a simple and reliable test for presence or absence of ductal pathology.

### FP075 ENDOSCOPIC TREATMENT OF POSTOPERATIVE BILE LEAKAGES.

FERETIS GH., BLIOURAS N., SKALTSAS S.,

HAMALAKIS G., KOURTESSIS A., KEKIS B.

ATHENS MEDICAL CENTER, ATHENS, GR.

Fourteen patients (8 females, 6 males) ranging from 8-72 years old, were referred to us, for endoscopic treatment of postoperative bile leakages. All of them had postoperative persisting (2 months) either external (12 patients) or internal (Bile Ascites 2 patients) biliary fistulas. On either ERCP or injection of contrast through the fistulous tract, no obstruction of the bile flow distaly to the site of bile leakage was detected.

Endoscopic treatment consisted of Endoscopic Papillotomy (EPT) and either Nasobiliary tube (NBT) or Endoprosthesis (EPR) insertion. EPT was performed in 12 patients, EPT plus NBT in 4 patients, EPT plus EPR in 8 patients, and NBT insertion without EPT in 2 patients. Rapid decrease of the amount of bile leakage and closure of external fistulas took place in approx. 2 weeks in 12 patients. In the two patients with bile ascites the later was absorbed within 15 and 17 days respectively. All endoscopic procedures were performed without morbidity or mortality. It is suggest that endoscopic bileduct drainage is a safe and an effective method of management of postoperative biliary fistulas.

### FP076 WALLSTENT ENDOPROSTHESES USED FOR THE PALLIATION OF OBSTRUCTIVE JAUNDICE DUE TO MALIGNANCY

A Adam, N Chetty, M Roddie, IS Benjamin*
Departments of Diagnostic Radiology and
Surgery*, Hammersmith Hospital, Du Cane Road,
London W12 OHS, England

A study was performed to assess the effectiveness and safety of self-expanding stainless-steel endoprostheses (Wallstent, Medinvent SA, Lausanne) used for the palliation of 32 patients with biliary obstruction caused by malignant tumours (21 at the hilum, 6 in the common hepatic duct and 5 in the common bile duct).

The endoprostheses are introduced over a 7F delivery catheter which causes minimal trauma to the liver, and expand to a maximum internal diameter of 10mm. The patients were followed up for 3 to 14 months and the effects and complications of stent insertion were recorded.

There was one case of haemobilia which resolved spontaneously. No cases of stent migration, occlusion due to bile encrustation, or tumour ingrowth were observed. The sole case of septicaemia was treated successfully. Two stent occlusions due to tumour overgrowth were relieved by a second percutaneous drainage procedure. No procedure-related deaths occurred but 3 patients died within 30 days.

The above results suggest that Wallstent endoprostheses achieve satisfactory palliation of malignant biliary obstruction with an acceptable complication rate.

FP077 **ENDOSCOPIC TREATMENT OF BILIARY STENOSIS WITH AN EXPANDABLE METAL STENT. SIX MONTHS FOLLOW-UP.**

*P.P.L.O.Coene, P.Fockens and K.Huibregtse. Dept. of Gastroenterology, Academic Medical Centre, Amsterdam, The Netherlands*

The main problem in treating biliary stenosis with endoprosthesis is clogging. In search of a solution to this problem, large diameter expendable stents have been developed. We report the six-months results with the endoscopically inserted WALLSTENT$^{tm}$, which has an diameter in compressed state of 9 Fr and a length of 10 cm. After expansion the diameter is 30 Fr , the length decreases to 6.8 cm.

Between May and July 1989 a WALLSTENT$^{tm}$ was placed in 33 patients who had symptomatic extrahepatic bile duct stenosis. The group consisted of 17 male and 16 female, median age 71 years (range 45-89). 26 had pancreatic cancer, 2 had ampullary tumor, 2 had cholangiocarcinoma and 3 had other diagnosis.

The stents were successfully placed in all patients. Jaundice disappeared in all but 1 patient, who was later found to have multiple liver metastases. Of the 33 patients, 12 have well functioning stents, median follow-up being 197 days; 2 patients had their stents removed when they underwent a Whipple procedure; 1 patient presented with cholangitis after 124 days and received a double surgical bypass for tumor ingrowth in the duodenum; another patient had cholangitis after 209 days and a conventional endoprosthesis was inserted through the WALLSTENT$^{tm}$; 17 patients died, 1 of septicaemia, 2 were jaundiced but had no fever an 14 died of non-stent related cause. So far no stent occlusion with biliary sludge has been documented. Tumor ingrowth through the mesh has occurred.

We conclude that the WALLSTENT$^{tm}$ can be easily placed in distal and mid-CBD strictures and that all patients have good initial drainage. With a long duration of patency, the WALLSTENT$^{tm}$ offers excellent palliation especially in those who survive more than 6 months.

### FP078 THE USE OF SELF EXPANDABLE STENTS FOR PALLIATION OF MALIGNANT BILIARY OBSTRUCTION

JS Laméris, EJ Hesselink, M van Blankenstein and OT Terpstra. Dep of Radiol, Gastroenterol and Surgery. University Hospital Rotterdam, NL

Patency of biliary stents depends, among others, on their diameter. Self expandable stents combine the advantages of a large diameter of the stent and a small-sized delivery catheter. To study the effectiveness of a stainless-steel self expandable stent (Wallstent, Medinvent SA, Lausanne) transhepatic installment was performed in 34 patients with a malignant biliary obstruction, which was located at the hilum in 15, in the common hepatic duct in 7, and in the common bile duct in 12 patients. All patients were endoscopic failures or had a history of repeatedly clogged endoscopically placed endosprostheses. All stents were installed over a 7 FR delivery catheter. Their length varied from 5-10 cm at a maximum diameter of 10 mm. Stent placement was successful in all. Balloon dilation of a stenotic common bile duct before stent placement caused transient hemobilia in one patient. No other complications were seen. The follow up time varied from 1-11 months. Seven patients died, six without signs of recurrent jaundice (median survival 11 wks). One patient with hilar cholangio carcinoma died after 10 wks with signs of jaundice and cholangitis due to tumour overgrowth. Two patients with irradiated hilar cholangio carcinoma developed recurrent jaundice due to tumour overgrowth distal to the stents after 12 and 16 wks. Obstruction caused by tumour ingrowth after 6 months was documented in a patient with biliary obstruction due to lymph node metastases from a colorectal cancer. In all other patients the stents are functioning well 1-8 months (median 4 months) after placement. Our preliminary results shows that large-bore self expandable stents can be placed transhepatically in a safe manner and that the long term patency rate seems superior to that with conventional stents. Long follow up data are needed to decide whether and in which patients the Wallstent should replace the use of plastic stents.

### FP079 THE ROLE OF ULTRASOUND GUIDED DRAINAGE IN MANAGEMENT OF HEPATOBILIARY INJURIES

F. Mekky, W. Shehab, O. Shafey
Alexandria University Hospital, Egypt

This study included fourteen patients presented after hepatobiliary surgery for liver trauma.

Ultrasonography reveals the presence of intra-obdominal collections. Diagnostic ultrasound guided aspiration was done and samples were subjected to bacteriological study and antibiotics were given accordingly. Six patients had intrahepatic abscesses, five following liver injury and one following hydatid liver excision. Two were small that needed simple aspiration, the other four needed percutaneous ultrasound guided pig-tail catheter insertion. Seven patients had bilomas. Two intrahepatic following repair of liver injury and only aspiration was needed. Five had extrahepatic bilomas, two following repair of liver injury and three following biliary surgery. These five patients were managed by catheter drainage. The last patient had sterile right subphrenic haematoma following repair of liver injury and only aspiration was needed. The mean cure time in this study was nineteen days.

### FP080 SELECTIVE ARTERIAL EMBOLISATION FOR HEPATIC TRAUMA

J.Pain, J.Karani, E.Howard

King's College Hospital, London, UK

The decision to either perform a laparotomy after hepatic trauma or to treat with conservative measures can be difficult. Surgery is indicated for ongoing haemorrhage which may be arrested by the Pringle's manoeuvre, arterial ligation, packing or, in extreme cases, vena caval occlusion. We have investigated the role of selective arterial embolisation in haemorrhage control, using CT scanning and angiography to assess the extent of liver damage and the magnitude of blood loss. Embolisation has stopped arterial haemorrhage and controlled large intrahepatic arterio-venous shunts.

We illustrate this technique with four cases and compare the indications and results with three further cases in which embolisation was not performed.

Early deaths after hepatic trauma are mostly related to uncontrollable haemorrhage. Selective arterial embolisation may prove to be a useful technique in reducing this mortality.

### FP081 EVALUATION OF BLOOD SUPPLY OF AND DRUG DELIVERY TO LIVER TUMORS USING BROMODEOXYURIDINE

H. Taniguchi, A. Oguro, T. Daidoh, A. Itoh, N. Tsukuda, Y. Shioaki, T. Yamaguchi, K. Sawai, T. Takahashi

First Department of Surgery, Kyoto Prefectural University of Medicine, Kyoto, Japan

Because bromodeoxyuridine (BrdU) is incorporated into the DNA sinthesizing (S-phase) cells, we can know the blood supply of the liver tumor by the injection of BrdU into either the hepatic artery or the portal vein, and also the delivery of anticancer drugs acting at S-phase when they are injected to these routes.

We evaluated blood supply of and drug delivery to liver tumors using BrdU in patients with metastatic liver cancer (21 colorectal cancers, 2 esophageal cancers, and 1 gastric cancer) and 8 hepatocellular carcinomas. At the time of hepatectomy, 200mg of BrdU was injected into the hepatic artery after exclusion of the portal vein for 7 metastatic and 2 primary liver tumors (A-group). The same dose of BrdU was infused into the portal vein after clamping the hepatic artery for 8 metastatic and 3 primary tumors (P-group). BrdU in the same dose was administered into both hepatic artery and portal vein for 3 metastatic cancers (AP-group). In addition, 200mg of BrdU suspended in 2ml of Lipiodol, a lipid contrast medium, was injected into the hepatic artery by Seldinger's method for 6 metastatic amd 3 hepatocellular carcinoma, 2 weeks before hepatectomy (L-group). The liver tumors were stained with avidin-bintin-peroxydase complex method using anti-BrdU monoclonal antibody after liver resection. The periphery of all metastatic tumors in A- and AP-group and 4 in P-group were labeled by BrdU. In cntrast, 4 metastatic tumors in L-group were stained even in the central area. All of hepatocellular carcinomas were stained from periphery to the deepest area. However, the labeling index of hepatocellular carcinoma was lower than that of metastatic liver cancer in all groups.

In conclusion; the metastatic liver cancer had both arterial and portal blood supply as well as hepatocellular carcinoma, and the delivery of anticancer agents acting S-phase using Lipiodol is excellent.

**FP082** HIGH DOSE INTRAARTERIAL INFUSION OF CISPLATINUM WITH EXTRACORPOREAL REMOVAL USING DIRECT HEMOPERFUSION UNDER HEPATIC VENOUS ISOLATION - AN EXPERIMENTAL STUDY ON THE PHARMACOKINETICS

Y. Maekawa, Y. Ku, M. Saitoh, M. Tominaga,
H. Ohyanagi, Y. Saitoh
1st Department of Surgery, Kobe University
School of Medicine, Kobe, Japan.

We have developed a new method of direct hemoperfusion (DHP) under hepatic venous isolation (HVI) in an attempt to reduce extrahepatic distribution of anticancer drugs during regional chemotherapy. Our previous studies have proved the efficacy of this system in use of adriamycin and mitomycin C (Ku, 1989). Accordingly, cisplatinum (CDDP) was applied to this system and evaluated pharmacokinetically. CDDP (2 mg/kg) was given continuously to mongrel dogs through the hepatic artery in 10 minutes. Plasma levels and tissue concentrations were compared between the animals treated by CDDP infusion alone (group I, n=3) and those treated additionally by the DHP of 20 minutes under HVI (group II; n=4). Plasma drug levels (mean±SD) were determined at the inlet and outlet of DHP and right external jugular vein (systemic level). Tissue concentrations were determined at 30 minutes after the end of drug infusions. Systemic levels reached a peak at 10 minutes after the initiation of drug infusion in both groups I and II, the values being 3.92±1.59 μg/ml and 1.40±0.19 μg/ml, respectively. The highest values at the inlet and outlet in group II were 4.90±1.11 μg/ml and 1.72±0.38 μg/ml, indicating effective adsorption by the DHP. Estimated removal rate was 44.7±9.6%. Mean tissue concentrations of the liver, heart and kidney were 1.97 μg/g, 0.50 μg/g and 2.40 μg/g of wet weight, respectively in group I. In contrast, tissue concentrations in the heart and kidney were reduced remarkably in group II animals, the values being 0.86 μg/g and 0.21 μg/g, respectively.

In conclusion, extraregional distribution of CDDP can be significantly reduced by the DHP under HVI. By this system, dose escalation of CDDP may be safely performed in the intraarterial chemotherapy for the liver tumors.

References:
Ku Y et al. J Jpn Surg Soc 1989;90:1758.

### FP083 AUTOLOGOUS KUPFFER CELL-HEPATOCYTE CO-CULTURES: A NEW RESEARCH TOOL

S. Singh, M.E. Bailey

University of Surrey and The Royal Surrey County Hospital, Guildford, Surrey, UK.

The use of isolated cells is an established research method. Kupffer cells have been used for more than a decade (Seglan 1973, Munthe-Kaas *et al* 1975).

A method of selectively isolating kupffer cells and hepatocytes from the same liver has been developed. This has not previously been reported. Previous workers have performed co-culture work on cells derived from two different animals and despite in-breeding some antigenic changes may occur, making interpretation of the results difficult (West *et al* 1989).

The kupffer cell isolation is a modification of a previously described procedure (Page and Garvey 1979) and the hepatocytes were isolated by a technique developed in our laboratory. This procedure consists of a combination of in-situ and biopsy liver perfusion with calcium free buffer, 0.5% collagenase and subsequent pronase incubation of the tissue providing the Kupffer cells, followed by centrifugation on a preformed Percol gradient.

The viability of the hepatocytes was always greater than 80% and the kupffer cells greater than 95% using Trypan Blue exclusion. Kupffer cells were identified by latex bead phagocytosis and Peroxidase staining. Hepatocytes were identified by their characteristic morphology.

Subsequent Transmission Electron Microscopy has shown the formation of specialised junctional complexes between the kupffer cells and hepatocytes thereby confirming their functional status.

**References**

Seglen PO Exp. Cell Res 1973; 82: 391-398.

Munthe-kaas AC, Berg T, Seglen PO, Seljelid R J Exp Med 1975; 141: 1-10.

West MA, Billiar TR, Curran RD, Hyland BJ, Simmons RL Gastroenterology 1989; 96: 1572-1582.

Page DT, Garvey JS J of Immunological methods 1979; 27: 159-173.

**FP084** FLOW CYTOMETRIC ANALYSIS OF DNA CONTENT IN HEPATOCELLULAR CARCINOMA

T.C. WU, T.J. LIU, J.L. Lan, L. HO
TAICHUNG VETERANS GENERAL HOSPITAL,
TAIWAN, R.O.C.

The relationships between DNA ploidy and survival have been reported in various tumors with controversial results, although the material was limited to hepaticollular carcinoma. For evaluating whether DNA content is an independent prognostic factor in this tumor, we analysed 47 surgically resected hepatoma tissue paraffin blocks from 1985 to 1987, using flow cytometry to determine it's ploidy pattern. The patients included 39 males, 8 females, aged from 17 to 71 years with a mean of 57 years. The survival time was from 1 months to more than 48 months. In 19 patients survival time was longer than 24 months, 9 of them showed aneuploid (47%) and 10 (53%) showed diploid in DNA pattern. The reminder survived less than 24 months, in whom diploid was found in 22 cases (78%) and aneuploid in 6 cases (22%)(p=0.2111). Our data showed DNA ploidy pattern can not predict the outcome of hepatocellular carinoma.

### FP085 TARGETED TREATMENT OF PRIMARY LIVER CELL CARCINOMA

J R Novell, P Mistry, K Reddy, A Hilson, K E F Hobbs
Academic Departments of Surgery and Medicine,
Departments of Radiology and Nuclear Medicine,
The Royal Free Hospital School of Medicine, London, UK.

Forty-one patients with unresectable primary liver carcinoma (PLC) have been treated using either a Lipiodol-epirubicin emulsion or I-131 Lipiodol (CIS UK) administered via the hepatic artery at angiography.

Thirty-five patients received up to 90 mg/m$^2$ of epirubicin emulsified with Lipiodol administered at 2-3 monthly intervals (range 1 - 9 treatments). There were no complications resulting from angiography. Commonly observed side effects were pyrexia, transient elevation of liver function tests, and abdominal discomfort and nausea at the time of cannulation. Leucopaenia occurred in only one patient (3%). Thirteen patients are alive 2 - 18 months from the start of treatment, a cumulative survival rate of 37%. Twelve have static disease on serial CT scans. Fifteen patients have died from progression of their tumour, and two from unrelated causes. Two patients in established liver failure died within 48 hours of treatment, and two cirrhotic patients died following attempted hepatic resection. One patient has undergone successful liver transplantation following Lipiodol therapy. The mean survival for the series is 5.0 months, against an expected survival of under 2 months untreated (Okuda et al, 1985).

Six patients received I-131 Lipiodol in a mean dose of 700 MBq (range 520-1160 MBq). Selective retention within tumour was seen in all patients. No patient developed haematological depression or other systemic side effects. One patient has died from extrahepatic disease at 3 months; 5 patients remain alive and well between 1 and 6 months. One has undergone resection of two tumour nodules which proved to be completely necrotic.

Lipiodol-targeted chemotherapy is administered at longer intervals than systemic chemotherapy, with fewer and less severe side effects; survival is comparable to that with systemic therapy. I-131 Lipiodol exhibits even lower toxicity combined with greater cytotoxic potential. We are currently engaged in further prospective trials of Lipiodol-targeted therapy.

Reference: Okuda K, Ohtsuki T, Obata H et al. Cancer 56:918-928 1985.

### FP086 INTRAARTERIAL IMMUNOCHEMOTHERAPY COMBINED WITH PARTIAL HEPATECTOMY FOR PREVENTING THE INTRAHEPATIC RECURRENCE OF HEPATOCELLULAR CARCINOMA

S.Arii, J.Tanaka, Y.Yamazoe, K.Fujita
Y.Hattori, N.Kan, Y.Nio, Y.Adachi,
T.Sasaoki, N.Funaki, S.Maetani and
T.Tobe
1st Department of Surgery, Kyoto
University School of Medicine

Intrahepatic recurrence(recurrence) after partial hepatectomy is one of a main cause of death in the patients with hepatocellular carcinoma(HCC). It is, therefore, urgently needed to clarify the predictive factors, for recurrence after surgery, thereby undertaking the prophylactic treatment for the patients with high possibility of the recurrence. In the present paper, we proposed the predictive factors for the recurrence, and introduced our strategy for preventing the recurrence of HCC after partial hepatectomy. 1). Study on the predictive factors for the recurrence. Using 132 patients treated with a variety of partial hepatectomies, cumulative non-recurrence rates were caluculated in relation to each of 12 clinicopathological factors, and multiple regression analysis was carried out. 2). Intraarterial immunochemotherapy to prevent the recurrence. This is composed of 3 factors; preoperative administration of immunopotentiatos (OK432),postoperative intraarterial infusion of OK432 and anticancer agents selected by in vitro sensitivity test, and transfer of autologuous cytotoxic T lymphocytes augumented and proliferated by crude IL2 and tumor extracts. Results were as follows. 1) Significant predictive factors were portal involvement, 3 and more tumors, more than 5cm in diameter, and capsular infiltration. 2) The preventive therapy was carried out to 14 patients with high risk of the recurrence. The 1 year and 2 year-non-recurrence rate were 82%, and 82%, respectively, while in control group 1 year and 2 year-non-recurrence rats were 43% and 36%, respectively. In conclusion, intraarterial immunochemotherapy seems to be promising prophylactic for the partially hepatectomized patients with high risk of the recurrence.

### FP087 UNRESECTABLE HEPATOCARCINOMA IN CIRRHOSIS: CHEMOEMBOLIZATION WITH MMC MICROENCAPSULATED

R. Doci, RA Audisio, F. Montalto,
L. Bellegotti, F. Garbagnati, L. Gennari
Istituto Nazionale Tumori, Milan

39 cirrhotic patients with unresectable HCC were treated by hepatic arterial chemoembolization with microencapsulated Mitomycin (MCCmc). 32 were males and 7 females with a median age of 57 years (range 47-79); 25 pts were Child A and 14 Child B; 21 and 18 were Okuda I and II respectively. The median diameter of the HCC was ≤6 cms. 17 pts had a single neoplastic lesion, 18 multiple and 4 had diffuse HCC. A total of 84 courses was administered (median 2/pt, range 1-4/pt). Neither treatment related mortality nor major toxicity were observed; only minor complications were detected: pain 35%, temperature 33%, ascitis 15% which always resolved spontaneously. The overall objective response was 40%. A CR was achieved by 5 pts (14%); 9 pts had PR (26%) and the median duration of the response was 25 and 5 months respectively. The actuarial 1-year survival rate was 42% and the median survival was 10 months. Patients who achieved the best results had Child A cirrhosis and Okuda I HCC.

Chemoembolization with MCCmc can be considered as a safe and useful treatment for unresectable HCC in cirrhotic pts because of its high feasibility, lack of severe toxicity, high response rate and excellent life quality provided.

**FP088** Changes in liverfunction after chemo-embolisation in patients with liver-cirrhosis and primary hepatocellular carcinoma.

W. Lauchart, H. de Groot, A. Littauer, R. Viebahn, J. Pirschel.

Dep. of Surgery and Radiology, Tübingen University, F.R.G.

Resectability of hepatocellular carcinoma (HCC) in cirrhosis is limited due to reduced liver function. Chemoembolisation (CE) offers in irresectable situations a palliative treatment modality. In a prospective study we evaluated the effect of CE on liver-function in patients with HCC and end-stage liver cirrhosis.
CE was performed in 10 patients with Child B/C-stage cirrhosis and biopsy proven HCC, injecting Adriamycin mixed with ethiodized oil (Lipiodol) directly into the tumor feeding hepatic artery(ies). Assessment of liver function was done by studying galactose elimination capacity (GEC), fractional indocyanine green clearance (IGC) and cholinesterase (CHE)-activity in addition biochemical parameters of liver cell integrity/function (GOT, GPT, GLDH and bilirubin) were determined.
The maximum of cell injury has been observed within 2 days after CE, with distinct increase of GOT, GPT and GLDH-activities, declining within 1 week towards baseline levels. However liver function parameters were reduced for a considerable longer period, CHE-activity, ICG- and GEC-tests reached pre-CE levels only at about 4 weeks.

In conclusion CE offers a well tolerated form of palliative treatment in irresectable HCC in liver cirrhosis. Despite liver function impairment due to CE biochemical and functional parameters recovered within 4 weeks after CE.

### FP089 CHEMOEMBOLISATION OF LIVER METASTASES WITH ALBUMIN MICROSPHERES LOADED WITH A PLATINUM COMPOUND: AN EXPERIMENTAL STUDY IN THE RAT

H Boutkan (1), H P J M Noteborn (1)

A Meinema (2), W Prevoo (1)

M Reuvekamp (1), J G McVie (1)

J A van Dongen (1)

(1) Department of Experimental Therapy and Experimental Surgery, Netherlands Cancer Institute, Amsterdam, The Netherlands (2) Department of Applied Chemistry, TNO, Zeist, The Netherlands

Treatment of liver metastases by chemoembolisation (administration of carriers loaded with chemotherapeutics) offers theoretical advantages above systemical or selective arterial administration of the free drug: lower systemic toxicity, lower organ toxicity, possibility of administraton of higher drug doses, achievement of higher intratumoral drug levels and the possibility of administration of poor water soluble drugs.

TNO1 is a poor aequous soluble platinim compound with higher antitumor activity and lower nephrotixicity in comparison with cisplatin. In a rat model with liver metastases of a CC531 colonic carcinoma, systemic and organ levels of the drug and anti tumor activity were determined after chemoembolisation of the metastases with human serum albumin microspheres loaded with TNO1 after administration via the gastroduodenal artery. Preferential arterial blood flow towards the metastases was visible. Systemic platinum peak levels after intraarterial administration were six times lower than after systemic administration of the microspheres. Platinum liver levels after arterial administration were two times higher than platinum kidney levels; after systemic administration, this was the opposite. Intratumoral platinum levels were eight times higher than platinum levels in the tumor surrounding normal liver tissue. Antitumor effect was microscopically examined: enhanced necrosis until total necrosis of metastases was seen in comparison with control animals without treatment or after treatment with empty microspheres.

In conclusion: chemoembolisation of liver metastases with human serum albumin microspheres with drugs incorporated into the matrix offers possibilities which should be studied in patients.

### FP090 NEO-ADJUVANT THERAPY FOR LIVER TUMOR BY ACTIVATED HEPATIC MACROPHAGE

S.Arii, T.Sasaoki, Y.Adachi, K.Monden
S.Itai, N.Funaki, H.Higashitsuji, and
T.Tobe
1st Department of Surgery, Kyoto University School of Medicine

For the purpose of the development of neo-adjuvant therapy against the sinusoidal spreading of liver tumor, the present study was designed to investigate the tumoricidal activity of hepatic macrophage(HM) located in sinusoid. Using Wistar rats, HM were isolated by the Method of Munthe-Kaas. Tumoricidal activity was measured with $^{51}Cr$-releasing assay and inhibition of $^{3}H$-TdR incorporation by using K562 and P815 cells as targets. Furthermore, superoxide ($O_2^-$), tumor necrosis factor(TNF) and interleukin-1(IL-1), which also possibly relate to the cytotoxicity, were determined. $O_2^-$ was measured with cytochrome C reduction method. TNF and IL-1 were determined with bioassay. Results were as follows. 1). HM from non-treated rats(control) exhibited 13.5±2.5% cytotoxicity against K562 in $^{51}Cr$-releasing assay, and intraperitoneal injection of OK432 significantly increased to 21.9±2.5%. Moreover, the addition of IL-2 to the culture of the HM from OK432-injected rats significantly increased to 33.6±7.3%, and additional administration of LPS to the culture led to the further enhancement(38.6± 4.9%). In $^{3}H$-TdR incorporation into P815, HM from the OK-432 injected rats inhibited the incorporation to 78.2 ±18.2%(control 63.5±6.5%). 2). The results about the chemical mediator and monokines are shown in the table.

| | control | OK432 | IL-2 | LPS |
|---|---|---|---|---|
| $O_2^-$ (nM) | 35.3±6.9 | 54.2±3.4 | 63.5±6.5 | 65.7±8.0 |
| TNF(%cyto) | 19.3% | 32.3% | N.D | 90.5% |
| IL-1(cpm) | 6598±582 | N.S | N.D | 50426±5210 |

In addition, administration of carageenan(macrophage inhibitor) increased the metastatic nodules(> 200) in the liver of the rats injected with AH130 tumor cells into portal vein, while OK432 inhibited the tumor growth(1.6± 3.6). (control 54±65). Thus, these suggested that appropriate enhancement of HM suppress the implantation and growth of cancer cells.

FP091 **UNRESECTABLE COLORECTAL LIVER METASTASES (LM) : A CONTROLLED MULTICENTRIC TRIAL OF INTRA-ARTERIAL HEPATIC CHEMOTHERAPY (IAC) VS STANDARD PALLIATIVE TREATMENT**

D. ELIAS*, Ph. LASSER*, M. HUGUIER, J.M. HAY, J. ESCAT, A. LAPLANCHE*, J.M. OLLIVIER, Ph. ROUGIER*
Groupe d'Etude et de Recherche sur les tumeurs hépatiques (GERTH) and Association Universitaire de Recherche en Chirurgie (AURC). (*) Inst. G. Roussy (IGR) 94805 Villejuif Cedex, France.

A multicentric randomized trial was conducted comparing IAC through implanted pump with continuous FUDR 0.3 mg/kg/day(d)x14 d every 4 weeks (w) (81 pts) to standard (Sd) IV bolus 5 FU (500 mg/m$^2$/d every 4 W) and/or symptomatic treatment (82 pts). Only patients (pts) with unresectable (multiple or not) isolated LM were included. Pretreatment pts caracteristics were well balanced between the treatment groups and 163 (98 %) of the 166 patients were available for analysis. In IAC-group 11 pts did not recieve IAC ; in Sd-group 41 (50 %) received IV 5 FU. Survival (S) was significantly improved for pts treated with IAC (LgR-p<0.02).

| Treatment | Pts | Median S (Mo) | 1-YR-S | 2-YR-S |
|---|---|---|---|---|
| IAC | 81 | 14 | 61 % | 22 % |
| SD | 82 | 10 | 44 % | 10 % |

Toxicity for IAC consisted mainly of chemical hepatitis (62,5 % at 1 year) and sclerosing cholangitis (35 % at 1 year). Only two significant prognostic factors were selected by a multivariate study : the extent of the hepatic involvment by tumor and the investigators experience.
**Conclusion** : These data indicate a significant moderate advantage for IAC. Better selection of patients according to the main prognostic factors must be considered whether to use IAC in clinical practice. Decreasing liver toxicity and improvement of extra-hepatic disease control are still required.

(Supported by : Association pour la Recherche Contre le Cancer and Caisse Primaire d'Assurance Maladie).

**FP092** COMPARING SURGICAL TREATMENT OF HEPATIC METASTASES FROM COLORECTAL CANCER TO NON OPERATIVE TREATMENT

L. Cozzaglio, F. Bozzetti, P. Boracchi,
R. Doci, L. Gennari
Istituto Nazionale Tumori, Milan.

The role of hepatic resection in metastatic colorectal cancer is still controversial since no controlled trials have compared surgical to medical therapy in homogeneous groups of patients. We have retrospectively compared the survival of two groups of patients with metastases confined to the liver (stage I and II by Gennari classification) after radical resection of colorectal cancer, treated by surgery (group 1) or chemotherapy (group 2). Surgery included major resections (14 pts) and minor non anatomical resections (25 pts).

Chemotherapy was delivered to 31 patients by systemic (14 pts) or intrahepatic route (3 pts) or both (14 pts), mainly using 5-FU, for a median time of 13 months. Treatment related mortality was nil. We have analyzed the distribution (chi-square test) of the patients by the main prognostic factors (age, sex, stage, time of onset of metastases, serum total bilirubin and alkaline phosphatase, site and stage of primary) and then we have tested all the factors for a univariate (log-rank test) and multivariate (Cox's regression model) survival analysis.

The most favourable prognostic factor was surgery and the survival curves between the two treatments were statistically different ($x^2$ =10.37, p=0.001). The survival rate at 36 months was 47% in group 1 and 23% in group 2. The risk of death (Hazard ratio) was 50% reduced in patients treated by surgery.

These data indicate that in limited metastatic liver disease from colorectal cancer, surgery has a beneficial role comparing medical therapy.

**FP093** A REVIEW OF LETHAL LIVER INJURY IN PORT-OF-SPAIN, TRINIDAD

1. C. RAGOONANAN, S. MAHADEO

Sangre Grande Hospital,

Trinidad, West Indies

Fifty-seven cases of blunt liver and associated injuries were reviewed. Most cases were victims of Road Traffic Accidents.

Although extra-abdominal injuries appeared to be the major cause of mortality in this study - an accepted teaching[1 2], - intra-abdominal injury alone was responsible for 42 percent of overall mortality. Of this group, lacerations to the liver appeared to be the cause of death in 50 percent, or 21 percent of overall mortality.

Liver injury involving the retrohepatic vena cava and hepatic veins remains a difficult problem with high mortality in most centres[3]. Simple to moderate lacerations, however, are effectively managed with low mortality[2 4].

We submit that the large and probably avoidable mortality associated with intra-abdominal injury is due to the widening disparity between our mobile emergency health services and an increasing motor traffic density.

References:

Richard H. Carmona - AM. J. Surg. Vol. July 1982; 144: 88-94

Walt A.J. - AM. J. Surg. 1978; 135: 12-8

Madding G.F. - Surg. Ain. N. Am. 1977; 57: 275-89

Leader BMJ - 20th October, 1979

FP094 **THE NON-OPERATIVE MANAGEMENT OF BLUNT LIVER INJURIES**

M. Hollands, T. Wilson, J.M. Little
Westmead Hospital, Sydney, Australia

This study was carried out to consider the role of non-operative management for patients with liver injuries. Comparisons of a number of parameters were made between patients treated operatively and those treated conservatively:

| Parameter | Conservative (55) | Operative (184) | P-Value ($X^2$) |
|---|---|---|---|
| Shock | 8 | 80 | <0.001 |
| Unconscious | 9 | 82 | <0.001 |
| Distended | 2 | 49 | <0.001 |
| Assoc. Injuries <2 | 30 | 37 | <0.001 |
| Coagulopathy | 1 | 63 | <0.001 |
| Grade Liver Injury >I | 4 | 85 | <0.001 |
| Length of Stay <15 Days | 29 | 73 | 0.08 |
| Transfusion <2 unit | 25 | 26 | <0.001 |
| Died | 2 | 59 | <0.001 |

2 patients treated conservatively required subsequent laparotomy for continued haemorrhage. No patient had a concealed intra-abdominal injury (e.g. pancreas or hollow viscus) which was missed.

In conclusion patients for whom a conservative plan of management is appropriate are not comparable to patients who require an operation. The low morbidity and mortality experienced by these patients reflects their injury status rather than the type of treatment. When such a plan of action is decided upon there remains a risk of concealed haemorrhage or unsuspected intra-abdominal injury.

### FP 0 9 5 LIVER TRAUMA AT GEVHER NESİBE HOSPİTAL

A. Bilge, E. Sözüer, M. Ogan

Erciyes University, Medical school, Kayseri, Turkey

Between November, 1976 and January, 1990, Gevher Nesibe Hospital of Erciyes University admitted 1428 multiple travma patients of whom 168 also had liver trauma. This paper rewievs the treatment, morbidity and mortality of 168 patients with liver trauma. The main age of patients (141 M, 27 F) was 26 years (2-65).127of168 patients(75.6 %) suffered blunt liver, the remaining(24.4 %) penetrating trauma.

The 41 patients with penetrating trauma underwent emergency laparatomy and treated surgically. 75 of 78 (96.1 %) patients with blunt trauma who had peritoneal lavage had a true positive result.The patients with blunt trauma were resuscitated and underwent laparatomy. At laparatomy, 89.8 % of patients were still bleeding from their liver injury. 103 of patients(81.1 %) required only minor surgical treatment wheras 7(5.5 %) patients required major surgical treatment; 2 right hepatic resection, 2 segmental resection, 5 hepatic arteriel ligation. In 15 (11 %) patients with simpler injury and 2 with major injury, only drainage was performed. The simpler forms of injury (class 1,2 and 3) were easily treated and yielded good results whereas treatment for major injuries involving lobar destruction and vena caval injury (classes 4 and 5) yielded poor results.

In this study the overall mortality of 168 patients was 30.35 %. In patiens with simpler injury the overall mortality was 26%, whereas in patients with major injury was 66%. The overall mortality for blunt liver injury 33.1 %, and for penetrating trauma 24.4 %. There were 34 complications in all patients. The common complications were pulmonaire problems(12), acute renal failure(7) and sepsis(6).

**FP096** MANAGEMENT OF BLUNT LIVER INJURIES

R. W. Strong
Princess Alexandra Hospital
Brisbane. Australia.

The management of blunt liver injuries can be a formidable challenge even to the most experienced hepatic surgeon. Fortunately, the majority of cases are Types I and II (after Aldrete) and cause no particular problem to the surgeon. The real challenge comes in managing Types IV, V and VI injuries.

Since 1960, 317 liver injuries have been treated at the Princess Alexandra Hospital, Brisbane. Penetrating trauma has been the cause in only 30 cases (9%). This paper reviews 200 blunt liver injuries treated during the past two decades. Major associated injuries occurred in 84% of patients. There were no deaths related to the liver injury in Types I, II and III. The overall mortality was 16% and the mortality specifically related tothe liver injury was 7%. Thirty eight patients (19%) required some form of liver resection.

The improvements in anaesthesia, resuscitation and intensive care together with the evolution of techniques in surgical management including adequate exposure, proper mobilisation and appropriate use of packing has been the mortality of the highly lethal Type VI injury decrease from 71% in the seventies to 20% in the eighties.

Aldrete et al. Am. Surg. (1979) 189, 466-74.

**FP097** LIVER TRAUMA. 77 OPERATE PATIENTS

J.González, F.Navarrete, J.Alvarez, L.Vazquez, J.Pérez-Lozana, M.A.Antolinez, L.Estrada
Hospital Covadonga,Oviedo,Spain

From January 1979 to December 1988, we collected 77 patients with hepatic trauma out of a total of 261 abdominal trauma cases that needed laparotomy.
The average age was 35.06 +/- 17.81 (X+/-SD) with a range of 5-76 years: 65(84.42%) males and 12(15.58%) females. 58 cases (75.32%) were suffering from blunt trauma and 77.59% of them were traffic accidents. There were 59 cases (76.62%) with associated lesions, 32 patients (45.56%) had both intra and extraabdominal lesions: 25 (42.37%) rib fractures, 17(28.81%) extremity fractures and 17(28.81%) spleen ruptures. In two patients there were CBD lesion and in two others gallblader lesion.
The most common clinical presentation was shock in 48 cases (62.34%) and abdominal pain in 35(45.45%). Peritoneal lavage as a diagnostic complementary test, was performed in 21 cases (27.27%) with positivity in 95.24% of them.
The usual method of treatment carried out was suture in 51 cases (66.23%). In 12(15.58%) hepatic resection was performed. In two patients we did CBD and in two others vascular (porta, cava) repair. 13 patients with capsular lacerations were treated using drainage. 21 patients (27.27%) were reoperated in the immediate postoperative period, 14 of those with hepatic complications.
Of the 60 survivors, 35(58.33%) had complications, pulmonary complications in 25 cases (71.43%) and abdominal infections in 26(74.29%).
The overall mortality rate was 22.07% (17 cases). Intraoperativelly 9(52.94%), 7 by exsanguination. Individual factors in relationship with mortality included blunt trauma, shock on arrival and severity of hepatic lesions according to Calne grading. There was no connexion between the factors of age, associated lesions and the amount of lesions.
CBD: Common bile duct.

Reference

Calne RY, Wells FC, Forty J. Br.J.Surg.1982;69:365-368.

FP098

### PANCREATIC TRAUMA -

### EVEN TODAY A SURGICAL CHALLENGE

K. Radebold, A. Ungeheuer, J. Lange
Dept. of Surgery, Techn. University Munich, W. Germany

Introduction: Two reasons are responsible for a continuous high mortality rate of pancreatic trauma: no adequate diagnosis is established when the patient arrives at the hospital, secondly, the pancreatic lesion remains undetected during a first operation because of other intraabdominal injury. Only a prolonged clinical course makes attentive to pancreatic trauma.
Patients: From January 1st 1979 until January 1oth 1990 21 patients with a traumatic lesion of the pancreas were transferred to the Dept. of Surgery, Technical University Munich; 5 women and 16 men with an average age of 33,6 years.
In 14 cases the rupture of the pancreas was caused by a car accident, 3x by a drop of high altidude, 2x by a stab wound or a gun shot.

The trauma of the pancreas was accompanied by a rupture of the spleen (8x),of the liver (5x) and a leackage of the duodenum (2x).
7 of 21 patients (33%) were first operated on because of other intraabdominal injury, before clinical deprevation made attentive to a pancreatic lesion.
Surgical treatment: In three cases of pancreatical contusion drainage was sufficient. 15 patients had a leftside resection of the pancreas; twice the lesion could be oversewed, in one case total duodenopancreatectomie was absolutely necessary.

Results: The overall letality was 23,8%;in cases with delayed diagnosis 38%.

Conclusion: An early diagnosis is indispensable to decrease morbidity and mortality of pancreatic trauma: by initial ERCP, followed by a CT scan; intraoperatively an inspection of the pancreas by opening the bursa omentalis and a Kocher manoeuver are of high importance.

### FP099 THE USE OF THE PREOPERATIVE APACHE II SCORE IN MAJOR SURGERY FOR LIVER TUMORS.

M. GAGNER, C. VONS, D. FRANCO,
R.L. ROSSI, J.W. BRAASCH
DEPARTMENT OF SURGERY, LAHEY CLINIC,
BURLINGTON, MA, USA & HOPITAL PAUL-BROUSSE,
VILLEJUIF, FRANCE

Major hepatic lobectomy carries a mortality of <5% and a morbidity <30%. The APACHE II score, a severity of disease classification comprises the sum of points given for age (A), physiological assessment (PA), and chronic health evaluation (CHE) and has been shown to predict mortality in ICU patients. The objective of this study was to evaluate the ability of the APACHE II score to predict morbidity & mortality in major surgery for liver tumors. Methods: We reviewed the charts of 65 patients who had simple or extended right hepatectomies for liver tumors from 01/80 to 09/89. The patients were divided into 3 groups according to the preop APACHE II score; low (0-3), mid (4-7) and high (≥8). Results: The postop morbidity and operative mortality increases as the preop APACHE II score increases. Patients in the low score group had a morbidity rate of 37% and no mortality, those in the mid score group had a morbidity rate of 55% and a 3% mortality, and those in the high score group had a morbidity rate of 83% and a 17% mortality. We also looked at the APACHE II score minus the age points (APACHE II-A) to look at the effect of only PA and CHE.

| APACHE II-A | n | MORBIDITY | MORTALITY |
|---|---|---|---|
| 0 | 20 | 4 (20%) | 0 (0%) |
| 1-2 | 29 | 17 (59%)* | 0 (0%) |
| ≥3 | 16 | 11 (69%)* | 2 (13%) |

*p <0.01 Fisher's exact test

The patients with no points for PA and CHE (age being the only risk) have a much lower rate of morbidity (20%) and no mortality as opposed to those who have points for PA or/and CHE who have a 3 fold risk. Conclusion: The preop APACHE II score correlates with postop morbidity & mortality in major surgery for liver tumors. The effect of chronic health evaluation points and physiologic assessment points on morbidity & mortality are greater than the effect of age points alone. It may be used by clinicians to predict morbidity & mortality and may identify a high risk group of patients who perhaps should be considered for therapeutic alternatives.

### FP100 NUTRITIONAL ASSESSMENT IN PATIENTS WITH OBSTRUCTIVE JAUNDICE

G.Tagliabue*E.Faleschini*F.Palma**
F.Colturani*D.Comi R.Colombo
M.Brugnani G.Latartara
*Istituto Pat.Chir. Univ. Milano
Servizio Dietologia Osp. L.Sacco
**Lab. Biochimica Osp. Cà Granda -MI-

Malnutrition is significantly associated with operative risks in jaundiced patients affected by malignant hepato biliary and pancreatic disease. Preoperative nutritional assessment in patients with percutaneous transhepatic biliary drainage (PTBD) could thus be desirable.Fifteen subjects (9 M-6 F,aged 51-95 years,mean 66) affected by pancreatic or hepatobiliary cancer eligible for intervention were assessed. Nutritional intake and nutritional status were evaluated by anthropometric parameters, biochemical tests and delayed hypersensitivity skin test. Results: caloric intake 2300-4150 Kcal,lipidic % 29-48; BMI 17.3-23.4;mid-upper arm muscle circumference <15°percentile in 93%;Bioelectric Impedance Analisys:body fat % <standard in 60%;CHI:muscle loss in 66%;biochemical data low levels Ht and Hb (86%),Fe (66%),prealbumin (80%), Zn (26%),Mg (13%),P (53%);high levels GOT,GPT (86%),γGT(100 %),Cu (60%);normal levels albumin,transferrin; delayed hypersensitivity skin test:anergy (80%),low levels lymphocytes (33%).
This study confirms the existence of malnutrition in jaundiced patients with PTBD. In spite of high caloric intake,they show decrease in muscolar mass and body fat. Moreover delayed hypersensitivity response is depressed with occurrence of anergy.

References:

1) Halliday A.W. et al. J Parent Ent Nutr 1988;12:43

**FP101** EFFECTS OF INTRAPORTAL NUTRITION IN MAN

E. Regalia, F. Bozzetti, G. Bonfanti,
L. Cozzaglio, A. Barbieri, G. Facchetti,
F. Montalto, L. Gennari.
Istituto Nazionale Tumori, Milan

There are only few experimental investigations on the feasibility and potential advantages of intraportal nutrition in animals and only a preliminary experience in man (Piccone et al 1980).
The purpose of this study was to compare some biochemical parameters of protein metabolism in patients who received portal or systemic nutrition after elective surgery for colorectal cancer. 20 patients were randomized to receive post-operatively for a week an infusion of glucose (100 gr), aminoacids (100 gr), lipids (10 gr) via portal (catheter in the gastroepiploic vein) (10 pts) or systemic (10 pts) route.We evaluated the basal concentrations of some visceral proteins (total protein, albumin, cholinesterase,transferrin, RBP, pre-albumin) and acute phase proteins (fibronectin, $\alpha$-2 macroglobulin, $\alpha$-1 anti-trypsin,$\alpha$-1 acid glycoprotein, haptoglobin, ceruloplasmin) and their variations in the first week post operatively and the nitrogen balance.Statistical analysis was performed by the student t test. There were no differences in the changes day per day of the visceral and acute phase proteins after surgery in the two groups of pts, but in the portal group there was a significantly better recovery of the level of cholinesterase, total protein and albumin at the end of the infusion versus the systemic group ($p < 0.002$, 0.005 and 0.03 respectively).The nitrogen balance was significantly better during intravenous infusion in the first 3 days after surgery ($p < 0.002$), but when the phase of reaction to the trauma started to decline, the portal infusion was significantly better than the systemic one ($p < 0.007$).These data, which require further investigation, suggest that short-term portal nutrition is feasible and safe and may be beneficial, as compared to a systemic nutrition, as regards protein metabolism.

References:
Piccone VA, Le Veen MM, Glass PG, et al. Surgery 87:263-70,1980.

### FP102 EXPERIMENTAL POST-HEPATECTOMY AND TPN STEATOSIS

A. W. Szawlowski(1), J. Simony-Lafontaine(2), B. Saint-Aubert(1), C. Astre(1), H. Joyeux(1).

Lab Nut & Exp Oncol(1) & Dep Pathol(2)

Cancer Inst., University of Montpellier

France

Short-term effects of 80% hepatectomy (HPX) and total parenteral nutritution (TPN) with or without lipids, on postoperative hepatic steatosis in dogs were studied. Dogs received (per kilogram body weight per 24 hours) either lipid (24 dogs) or non-lipid (24 dogs) isocaloric (36 kcal) and isonitrogenous (0.24 g) TPN for 24, 48, 72 or 96 h postoperatively. There were 3 HPX and 3 Sham-operated controls for each TPN course. In the lipid-fed group, 70% of nonprotein energy was carbohydrate (dextrose) and 30% was lipid (10% Intralipid) while in the non-lipid-fed group 100% of nonprotein energy was carbohydrate.

Postoperative hepatic steatosis in liver biopsies taken at necropsy after TPN was considered mild (+), moderate (++), or intensive (+++) depending on whether fat globules occupied $<25\%$, 25-50%, or $>50\%$ of the section. The occurrence of steatosis differed significantly in HPX versus Sham-operated dogs, Mann-Whitney test ($p < 0.002$), whereas the differences between lipid-fed and non-lipid-fed dogs within those groups did not. The intensity of steatosis was comparable in the two TPN groups.

Our results suggest that 80% HPX encourages the development of hepatic steatosis more than does moderate short-term TPN. The intensity of the steatosis is equally influenced by HPX and TPN.

### FP103 CLINICOPATHOLOGICAL STUDY OF MICROMETASTASES AROUND MACROMETASTASES IN HEPATIC METASTASES

M. Miyazaki, T. Isono, I. Udagawa,
H. Koshikawa, J. Matsumoto, T. Koyama, K. Okui

1st Dept. of Surgery, Chiba University, Japan

For the treatment of hepatic metastatic tumors, hepatic resection has been selected as the first choice as possible. However hepatic resection has induced 20-50% of 5 year survival. The most common cause of postoperative recurrence is metastasis in the remnant liver which would most remarkably influence the survival. The aim of this study is to investigate the histological micrometastasis around macroscopic metastatic nodules in resected specimen of hepatic metastatic carcinoma and to analyze the clinical significance of micrometastasis.

The subject was 23 patients who underwent hepatectomy more than segmentectomy for hepatic metastases from gastrointestinal carcinoma. The micrometastasis was defined as discontinuous from macroscopic metastasis and less than 1 mm in diameter. The micrometastases were found in 8 of 23 cases, 34.8%. They were 0.2 to 1.0 (0.52±0.35, Mean ± SD) mm in size and 1 to 19 (4.1±6.0) in number. Their distances from the margin of macroscopic metastases were 2.0 to 29.0 mm and three of 8 micrometastases (37.5%) were in different subsegments from those of macroscopic metastases. Seven of 8 micrometastases were histologically found in the portal vein, one in hepatic vein and sinusoid. These results could reveal that surgical margin in hepatectomy for hepatic metastases requires at least 3 cm and that wedge resection is not suitable for the choice of the operative procedure for hepatic metastases because of unsatisfactory surgical margin.

### FP104 OCCULT HEPATIC METASTASES OF COLO-RECTAL CANCER: ROLE OF HEPATIC CT SCAN AND ULTRASONOGRAPHY IN THEIR DETECTION

L. Sarli, N. Pietra, F. Carreras,
E. Longinotti, M. Gafà, A. Peracchia
Il Clinica Chirurgica, Parma University
Italy

Occult hepatic metastases (OHM) of colo-rectal cancer are those not evident to the surgeon at laparotomy (1). In this study we first evaluated the accuracy of hepatic CT scan and ultrasonpgraphy (US), commonly performed prior to surgery, to detect hepatic metastases. To this end we examined 850 patients operated on for colo-rectal cancer. We detected hepatic metastases in 166 patients at laparotomy. The CT and US accuracy rate proved to be in correlation with intraoperative measurements of lesion diameters and is reported in the following table.

| | diameter<2cm | 2-4cm | >4cm | total |
|---|---|---|---|---|
| CT accuracy (%) | 42.8 | 75 | 90.1 | 78.4 |
| US accuracy (%) | 43.7 | 71.4 | 93.3 | 79.8 |

Secondly, we evaluated the extent to which these examinations were able to rule out the presence of OHM. We believe that hepatic metastases detected after curative surgery without any simultaneous local recurrence must be considered as already present, but occult, at surgery, 466 patients who underwent curative resection were subjected to a routine hepatic US and CT follow-up. Hepatic metastases were detected in 49 patients: 28 had simultaneous local recurrence and so 21 must have had OHM at time of surgery. The following pre-operative investigations were unable to detect hepatic metastases in these 21 patients: 16 hepatic US and 5 hepatic CT scans. Sensitivity of these examinations, in the light of those 21 diagnostic errors, decreased to 69.2% for US and to 69% for CT.

This study shows that hepatic US and CT scan are not sufficient to rule out OHM.

1) Finlay I G, McArdle C S, Occult hepatic metastases in colo-rectal carcinoma. Br.J.Surg. 1986; 73, 732-5

FP105 DEFINITIVE VALUE OF INTRAOPERATIVE LIVER ULTRASOUND IN SURGERY FOR GASTROINTESTINAL TUMOURS

H Boutkan, W Luth, F Barkhof, M A Cuesta
S Meijer
Department of Surgery and Department of Radiology, Free University Hospital, P O Box 7057, 1007 MB Amsterdam, The Nether-Lands

Intraoperative liver ultrasound is a reliable method to detect liver metastases in gastrointestinal tumors. During operation fine needle biopsy can confirm the diagnosis of malignancy. The limitations seen with trans abdominal ultrasound (disturbances of costal bones, subcutaneous fat and intraluminal bowel gas) are not seen with intraoperative ultrasound with direct placement of the transducer on the liver surface. Intraoperative ultrasound offers possibilities to locate and visualize branches of portal veins, hepatic arteries and hepatic veins. Also the exact number of metastases in the different segments can be determined as other hepatic lesions can correctly be diagnosed and located. In the Free University Hospital intraoperative ultrasound was consequetively performed in 34 patients with gastrointestinal and hepatic tumours and compared with the preoperative investigations (CT-scan, MRI and ultrasound). Of the 34 patient 32 were evaluable. In 9 cases, intraoperative ultrasound offered a definitive diagnosis different from the preoperative examinations. In 7 cases (22%) this led to alteration of the policy during operation, for instance smaller resections than planned preoperatively. But also in some cases the diagnosis was cysts instead of metastases. Examples will be demonstrated.
In conclusion: intraoperative ultrasound of the liver is of definitive value during the operation for gastrointestinal tumours and is superior to preoperative imaging! It should be recommended that surgeons use this technique in cooperation with the radiologist.

### FP106 THE VALUE OF CONTRAINDICATIONS TO RESECTION OF COLORECTAL SECONDARIES TO THE LIVER

R. Stangl, A. Altendorf-Hofmann, J. Scheele
Department of Surgery, University Hospital, Erlangen, FRG

Three factors are currently stressed as to provide a substantial contraindication to resection of colorectal liver metastases: extrahepatic disease, more than three individual nodules, and inability to achieve a clear margin of at least one centimeter. The importance of satellite metastases is controversial (Ekberg et al. 1986, Cobourn 1987)

In our series of 218 potentially curative liver resections performed from 1960 through 1988, 152 patients (70 %) fulfilled one ore more of these criteria (margin 5 - 9 mm: 47, ≤ 4 mm: 87; ≥ 4 individual metastases: 22; satellite nodules counting for a total of ≥ 4: 5; extrahepatic disease: 26).

Excluding 30 day mortality, median survival time for this "high-risk" group was 31 months, and five-year survival and tumour free survival were 38 and 33 percent, respectively. This was slightly different from 46 and 37 percent in patients unaffected by either factor ($p<0.05$). However, 15 of 54 "high-risk" patients operated prior to 1985 have survived five years, three of 11 undergoing surgery before 1975 have survived 15 years, and the two resected in the 1960's both lived longer than 23 years.

In contrast to common recommendations, these results do not justify to established either criterion as absolute contraindication to surgery. They are qualifiers of prognosis (Adson 1989, Saenz et al. 1989), but do not preclude surgical success. Even in the presence of extrahepatic disease tumour-free survival of more than five years has been observed in our series, as is reported by others (Hughes et al. 1989).

References:
Ekberg M, Tranberg KG, Andersson R, et al: Br J Surg 1986; 73: 727 - 31
Cobourn CS, Makowa L, Langer B: 1987 Surg Gynecol Obstet 1987; 165: 239-246
Saenz NC, Cady B, McDermott WV jr, et al: Surg Clin North Amer 1989; 69: 361-70
Hughes KS, Scheele J, Sugarbaker PH: Surg Clin North Amer 1989; 69: 339-60
Adson MA: Arch Surg 1989; 124: 1023-1024

### FP107 MULTIVARIATE ANALYSIS OF 100 PATIENTS RADICALLY RESECTED FOR HEPATIC COLORECTAL METASTASES

R. Doci, P. Bignami, F. Montalto,
F. Bozzetti, L. Gennari
Istituto Nazionale Tumori, Milan.

One hundred patients with hepatic metastases from colorectal cancer underwent radical liver resection. A typical lobectomy was performed on 50 patients and non anatomic resection on the others.

Mortality was 5% and major morbidity 11%. The actuarial 5-year survival for stages I, II and III was 42%, 34% and 15% respectively (p=.0002). The overall actuarial 5-year survival was 30%.

The prognostic importance of different characteristics of the patient and of the tumour was evaluated at first by monofactorial and then by multivariate analysis. Among the considered factors: age of patient, site of primary, disease-free interval between treatment of primary and of hepatic metastases, preoperative CEA levels, multiplicity and number of metastases, did not influence the prognosis, while sex (p=.024), stage of primary (p=.026), extent of liver involvement (p=.00003), distribution of metastases (p=.01) and type of surgery (p=.028) significantly affected the prognosis as single factor. The multivariate analysis found that only the extent of liver involvement and the stage of primary significantly influenced survival.

The results of present study suggest that the extent of liver involvement may be a significant, even though not absolute, indicator of late results and that the staging system adopted shows a good prognostic correlation.

### FP108 PRIMARY RESULTS OF THE UICC TNM FIELD STUDY LIVER METASTASES

F. Köckerling, P. Hermanek, F.P. Gall,
C. Schneider
Department of Surgery University of Erlangen

The UICC TNM field study liver metastases, a clinical follow-up study using unselected data, is designed in order that it can be used as the basis of a generally accepted classification and staging system for liver metastases with broad application. The study design is that of a multi-institutional (n = 45) open prospective study. There are two study versions, version A with an extended protocol and version B with minimal requirement. For any cancer reported by the institutions, data on all patients presenting with liver metastases at the time of initial diagnosis, treatment or during follow-up must be submitted. This also include all untreated patients diagnosed elsewhere and later referred to the participating institution for treatment. Between January 1st 1988 until October 15th 1989 there are 653 patients collected in this study, 461 documented with version A and 192 with version B. Primary tumor site was colon (n = 288; 44,1 %), rectum (n = 153; 23,4 %), stomach (n = 71; 10,9 %), pancreas (n = 52; 8,0 %), oesophageus (n = 11; 1,7 %) and other (n = 78; 11,9 %). Synchronous hepatic metastases appeared in 248 cases (53, 8%), metachronous in 213 (46,2 %). Clinical evaluation concerning the extent of exhepatic disease at time of diagnosis of liver metastases showed local regional tumor manifestation in 212 cases (32 %), other distant metastases in 54 cases (8 %) and both in 125 cases (19 %). Consequently liver metastases as sole tumor manifestation was only found in 243 (37 %) patients.Curative resection rate in the extrahepatic disease group was 15 %, in the group without extrahepatic disease 47 %.

FP109

### SURGICAL REINTERVENTION AFTER PREVIOUS RADICAL HEPATIC RESECTION FOR COLORECTAL METASTASES

M. Rees, R. Stangl, G. Plant,
A. Altendorf-Hofmann, J. Scheele
Basingstoke District Hospital, Basingstoke, UK.
University Hospital, Erlangen, FRG.

Radical excision of hepatic metastases from colorectal origin is associated with good long term prognosis in up to a third of patients. Recurrence of disease is usually widespread but may be isolated within the liver and/or lung in a small number of patients. We report the outcome of 17 patients who underwent further hepatic and/or pulmonary resection at a mean follow-up of 17 months after previous hepatic resection. Eight patients underwent isolated hepatic resection, 5 isolated pulmonary resection, 3 combined hepatic and pulmonary resection and 1 patient underwent combined hepatic and adrenal excision. One patient died post-operatively from hepatic failure after extensive hepatic resection. Five patients have died from progression of disease a mean 14 months after reintervention. Of the 11 survivors, 1 patient has local recurrence 2 years on but 10 patients remain free of disease a mean 21 months (range 4-57 months) after their second operation.

These results demonstrate that in a selected group of patients who recur after previous resection of hepatic metastases from colorectal cancer, further excision may lead to worthwhile disease free survival and possible cure.

**FP110** RESECTION OF NOT COLO-RECTAL HOEMATOGENOUS HEPATIC METASTASES.

G.BENATI,F.G.MUSAJO,G.MANGIANTE,L.MARCHIORI, A.TENCI,M.MAINENTE,A.ASNICAR,I.DAL DOSSO,V. COSTA,N.NICOLI.
CLINICA CHIRURGICA UNIVERSITY OF VERONA
VERONA ITALY (DIR. A. DAGRADI)

From 1970 to 1989 218 hepatic resections have been performed for metastatic desease at the Clinica Chirurgica of the University of Verona.44 resections in 39 pts were undertaken for not-colorectal hoematogenous metastases(11 endocrine t.,6 breast t.,5 pancreatic t.,5 masenchymal t.,3 ovary t.,3 eye melanoma,2 gastric t.,2 not-known origin t.,1 lung t. and 1 kidney t.).In the last group metastases were single in 19 pts. and multiple in 20.22 anatomical resections and 22 non-anatomical resections were performed.During hepatic procedure abdominal extrahepatic metastases were resected in 3 pts.In 4 pts repeated hepatic resections were performed.We had 2 hospital deaths and only one major complication(1 bile leak,that required surgical repair).
Presently 29 pts are alive,9 dead(7 for tumor spread)and 1 lost to follow-up.11 pts survived more than 36 months from the hepatic procedure:5 with endocrine t.(203,157,70,38,36 mo),3 with mesenchymal t.(62,37,36 mo),1 with breast t.(55 mo),1 with ovarian t.(80 mo)and 1 with lung t.(42 mo).Only 2 of the"long-survivors"are dead,both carcinoids at 70 and 36 mo after hepatic procedure.
Although our results are not conclusive as the ather reports from the literature it seems that in selected cases surgery can achieve long term survival also for not colorectal hepatic metastases.

**FP111** **SIXTY HEPATIC RESECTIONS FOR LIVER METASTASES (LM) FROM EXTRA COLO-RECTAL CANCERS (ECR)**

D. ELIAS, Ph. LASSER, E. DESRUENNES, C. HARDY.
Institut Gustave-Roussy (IGR) 94805 Villejuif Cedex, France.

From 1983 to 1989, sixty partial hepatectomies have been performed in 54 patients for LM from ECR. Origins were very miscellaneous : breast : 12, apudoma : 12, testis : 6, stomach : 6, soft tissues : 3, ORL : 2, unknown origin : 2, kidney : 3, choroid melanoma : 2, gallblader : 2, small bowel : 1, anus : 1. Most of them received pre and post-hepatectomy chemotherapy. The post-operative mortality was 1,6 % (one case) and the post-operative morbidity was 18 %. With a median follow-up of 32 months, the survival at one, two and three years according to Kaplan-Meier for all the patients was respectively 74 %, 49 % and 45 %, and the survival without recurrence was respectively 72 %, 34 % and 27 %.

The results were very different according to the origin of the carcinoma. Currently, trends only can be concluded from this serie, because number of patients and follow-up are small. Benefic trends appeared for apudomas (also in case of palliative hepatectomy) and for kidney and stomach providing that indications will be strict (metachronous, strictly isolated to the liver and no more numerous than two LM). Doubtful trends appearead for LM from breast, testis and unknown origin carcinomas. Negative trends appeared for LM from melanoma, soft tissue, ORL and anus carcinomas.

FP112

### THE TREATMENT OF LIVER TUMOURS BY HEPATIC CRYOTHERAPY

R M Charnley, J Doran, D L Morris

Dept of Surgery, University Hospital, Nottingham

A new design of cryoprobe has been developed so allowing the treatment of hepatic tumours to be performed with only the destruction of a small rim of normal liver tissue around each lesion. The probe is placed into the centre of the lesion under intraoperative ultrasound control. Liquid nitrogen is passed down to the tip of the probe where it circulates. The iceball which forms around the probe is imaged easily by intraoperative ultrasound and its size is monitored. Cryotherapy continues until there is 10 mm clearance on each side of the tumour.

64 lesions (9 impalpable and only detected by intraoperative ultrasound) have been treated in 15 patients (12 with colorectal metastases) using this technique. There have been no major complications and the procedure has been well tolerated. In 8 of 10 colorectal patients followed for more than 2 months, serum CEA concentrations have fallen following cryotherapy, in some cases to baseline levels. Postoperative CT scans have demonstrated areas of destruction at the sites of treated metastases which often contain gas from tissue necrosis.

Although it is too early to comment on any actual benefit derived from cryotherapy, it has been shown that the procedure is very well tolerated and that tumour destruction does occur. It is envisaged that hepatic cryotherapy may be useful for treating those patients with a small number of unresectable colorectal liver metastases and unfit patients with primary liver tumours who are unfit for hepatic resection.

FP113

### PROXIMAL BENIGN STRICTURES OF HEPATIC DUCTS

**N. Kuzovlev and E. Galperin**

**1st Moscow Medical Institute, USSR**

Between 1973 and 1988 we observed 193 patients with benign strictures of hepatic ducts at a level of their confluence (114), one (24) or both (55) hepatic ducts. Most of patients were in a grave state prior to surgery: 80.5 % of them had long-term mechanical icterus and purulent cholangitis, 19.5 %- secondary biliary cirrhosis, 17 % - biliary sepsis, 10 % - cholangiogenic abscesses in the liver, in subphrenic or subhepatic spaces. 78 % of patients were hospitalized with restricture of hepatic ducts as a result of earlier performed operations.

When selecting the method for biliary reconstruction a length of unchanged wall of hepatic duct was taken into concideration as well as topographic relations between the left hepatic duct and the surrounding vessels. In 25 patients biliary-jejunal reconstruction was done after wide incision to the left hepatic duct, with precise apposition of sero-mucosal layers of intestine and the duct by separate sutures 5/0. In 168 patients biliary-digestive anastomosis was carried out with long-term stenting of the both ducts over an exchangeable transhepatic drain.

There were 14 deaths (7.2 %), good remote results were observed in 78 % of patients.

### FP114 BILIARY INTESTINAL ANASTOMOSIS IN THE TREATMENT OF IATROGENIC BILE DUCT STRICTURE

P.L. Yao, T.Z.Xu

Xinjiang Medical College, CHINA

There were 29 cases of iatrogenic common bile duct injury during the operation of cholecystectomy for the acute or chronic cholecystitis and cholelithiasis in the past 30 years 0.8% of such operations in the same period.

26 cases, the common bile duct of patient were interrupted and ligated during the time of operation. The injuries were found and treated right following the accident in 5 cases, 24 cases (82.8%) developed iatrogenic bile duct stricture post-operatively.

10 cases were male, 19 were female, age ranged from 22 to 68. The diagnosis of stricture was made mainly depending upon the operative history and PTC examination.

Reconstruction of biliary intestinal continuity was performed on all the patients in different period, among them, 19 cases (79.24%) were done a Roux-en-Y anastomosis between proximal end of common bile duct and jejunum, 4 cases (16.6%) the bile duct was directly anastomosed to duodenum.

Follow up study from 1 to 25 years, 19 cases (65.55%) recovered completely and doing thier ordinary works well, 8 cases are still suffering from a certain degree discomfort of abdomen, 1 case became a "plant-like" patient due to the accident of exdural spinal anesthesia, 3 cases were fatal because of hepatic failure.

FP115 TRANSHEPATIC DRAINAGE IN TREATMENT OF CONSEQUENCES IATROGENIC LESIONS OF BILE DUCTS.

L.Oleszkiewicz,M.Nienartowicz
J.Jurecki

III Clinic of Surgery Medical Academy

Wrocław,Poland

66 patients suffering from consequences caused by iatrogenic lesions/hurt,ligation,section/of the bile ducts were treated in our Clinic.The lesions,unprovided intraoperatively,inducted fistulas,abscesses and contractions of the bile ducts.Enabling free bile flow from the liver to intestine becomes in those cases a very difficult task.The efforts to solve this problem by operation have not been always successful.One of the operation ways was anastomosis of small intestine loop separated due to Roux en Y method with hepatic porta and the use of transhepatic drainage of the bile ducts and of the anastomosis.That kind of operation was introduced in our Clinic in 1986. Up to the end of 1988 8 patients were treated according to the mentioned method.One of the drain ends was enducted through intestine loop while the second one through the lobar duct and hepatic parenchyma.6patients had the drain enducted through the right hepatic lobe,one patient throughthe left hepatic lobe and one woman double drainage was appiled. The everage drain keeping lasted 12 months and during that time it was changed every 3 months. Bacteriologic examinations most often showed: E.coli, Streptococcus foecalis,Staphylococcus aureus.
A very good results of the treatment was obtained in 7 patients.In one woman, inspite of 5 operations undertaken in other hospitals,the bile flow from the liver to intestine was not satisfactorily protected and cirrhosis of liver took place.In the state mentioned above that woman became the patient of our Clinic.There transhepatic drainage was applied resulting in the improvement of the patient's state.Our observations allow to state that transhepatic drainage is an efficient way of treating consequences of the bile ducts lesions,especially the 'high' ones situated near hepatic porta.

### FP116 ROUX-EN-Y HEPATICOJEJUNOSTOMY FOR LONGTERM BILIARY ACCESS AND PERCUTANEOUS BALLOON DILATATION OF INTRAHEPATIC STRICTURES

JEJ Krige, SJ Beningfield*,
PC Bornman, J Terblanche
Departments of Surgery and Radiology*,
University of Cape Town and MRC Liver
Research Centre, Cape Town, South Africa

Seventeen symptomatic adult patients with bilateral intrahepatic strictures and associated stones (9 patients) underwent 75 post-operative percutaneous transjejunal biliary balloon dilatations between 1986 and 1989. The strictures were due to primary sclerosing cholangitis (PSC) (5 patients) iatrogenic (5 patients) Caroli's disease (2 patients) choledocal cyst (2 patients) and primary intrahepatic stones (3 patients). All patients underwent hepaticojejunostomy and construction of a 15 cm permanent jejunal access loop[1] and post-operative transjejunal biliary dilatation of the strictures and stone extraction using a biliary guide-wire, co-axial catheter and 7 Fr Gruntzig angioplasty balloon catheter combination.

Five patients with PSC and bilateral diffuse intrahepatic strictures underwent 25 dilatations (mean: 5, range: 2-10). Two patients are well without jaundice, 1 has died of cholangiocarcinoma and 2 have progressive disease despite a mean of 8 percutaneous biliary dilatations over 30 months. Five patients with iatrogenic strictures underwent 15 dilatations (mean: 3, range: 1-6) and are asymptomatic without residual stones. Seven patients who had Caroli's, choledocal cysts or primary intrahepatic stones underwent 20 dilatations (average: 2.9, range: 1-9). Two patients have residual intrahepatic stones and 1 patient has required a left lateral segmentectomy for removal of stones. Minor complications related to percutaneous dilatation occurred in 6 patients.

The combined radiological and surgical approach with hepaticojejunostomy and post-operative biliary dilatation provides an effective method of treating symptomatic patients with diffuse bilateral intrahepatic strictures and associated stones.

1. Krige et al. Br J Surg 1987;74:612-613.

### FP117 SURGICAL TREATMENT OF NON NEOPLASTIC DISEASES OF BILIARY DUCTS

L Cimino, D Iafrancesco, S Ballatore

M Pizzo, M Amati

Osp San Giacomo, Div di Chirurgia, U S L

Rome, Italy

The revision of the survey on the surgical treatment of non neoplastic diseases of biliary ducts in the years 1970 through 1987,gives the authors the cue to comment upon the various types of operations,the advantages and possible complications of the surgical techniques adopted,and the results obtained both in the immediate and long term period.
The 1458 cases which are the object of the present survey bring the authors to assert that the choice of the type of intervention to perform is made in relation to the cholangiographic report,both pre- and intra-surgical, taking account of the seat of the pancreatic bile duct lesion, of its dimensions,of the condition of the bile duct itself,of the presence of acute phlogosis or jaundice, and of the patient's general condition.
The authors furthermore maintain that the transduodenal papilloplasty of the Oddi sphyncter is among the operations the one to obtain the best results in the internal bile drainage as it less alters the anatomic and physiological integrity of the biliary-pancreatic confluence.
The 964 cases of papilloplasty of the Oddi sphyncter,the 176 cases of supra-duodenal choledochoduodenal junction, the 33 cases of hepatic and duodenal jejunostomy and all the other cases treated with diverse surgical techniques and the ones cured by endoscopy, make a vast matter of debate which the authors examine highlighting the type of pathology as cause of the operation and as outcome mainly in relation to biliary-digestive regurgitation;these account for the main cause of the residual dispeptic disorders.

References:
ARMSTRONG C.P., Surgical experience of deeply jaundiced patients with bile duct obstruction.Br.J.Surg.,71:234,1984.

FP118 **CARCINOMA OF EXTRA-HEPATIC BILE DUCT ANALYSIS OF 572 CASES**

B. LAUNOIS, R. REDING, J.L. BUARD, G. LEBEAU
Clinique Chirurgicale - C.H.U. de RENNES - FRANCE

572 cases of primitive carcinoma of extra-hepatic bile ducts (gallbladder and peri-ampullary tumors excluded) collected from 55 surgical centers and treated principally between 1974 and 1987 were retrospectively reviewed by means of a computerized analysis. 321 (56 %) of the lesions were in the upper third of the bile ducts (proximal carcinoma), with, among them 63, 93 and 165 being respectively types 1, 2 and 3. 72 (13 %) were middle third and 101 (18 %) lower third bile duct carcinomas, the remaining cases being diffuse lesions. Resectability, operative mortality and long term survival according to tumor localization and surgery (curative or palliative) are given in the table.

| LOCALISATION (RESECTABILITY RATE) | OPERATIVE MORTALITY | | MEDIAN SURVIVAL (MONTHS) | |
|---|---|---|---|---|
| | RESECTION | PALLIATIVE SURGERY | RESECTION | PALLIATIVE SURGERY |
| UPPER THIRD (31.9 %) | 15,5 % | 30.9 % (p<05) | 23 | 9 (p<.001) |
| MIDDLE THIRD (46.5 %) | 22.6 % | 18.8 %(NS) | 13 | 9 (p<.05) |
| LOWER THIRD (50.5 %) | 26.1 % | 18.4 %(NS) | 64 | 8 (p<.001) |

Over-all one year survival (operative mortality excluded) was 67.6 % after tumor resection vs 30.6 % after palliative surgery (p<.001). Long term results after surgical resection were correlated with local extension of the biliary duct carcinoma : 2 years survival rate was 65.6 % for stage T1, 48,0 % for T2, 28.9 % for T3 and 0 % for T4. Similarly, absence of lymph node invasion or hepatic metastases significantly improved survival. In conclusion, results of the French Surgical Association Survey confirm the benefical effect of tumor resection on long term survival in patients with extra-hepatic bile duct carcinoma particularly for upper third localization.

### FP119 DIFFERENTIAL DIAGNOSIS OF KLATSKIN TUMOURS: PREOPERATIVE DIAGNOSIS IS OFTEN INCORRECT.

LA WETTER, CA PELLEGRINI, LW WAY. DEPARTMENT OF SURGERY, UNIVERSITY OF CALIFORNIA, SAN FRANCISCO, U.S.A.

A presumptive diagnosis of Klatskin tumour is usually made when a focal stenotic lesion of the common hepatic duct is seen on a cholangiogram of a deeply jaundiced patient. Treatment usually proceeds directly from this point because proof of diagnosis is rarely possible by biopsy. Because the accuracy and consequences of this strategy have never been tested, we contrasted the preoperative diagnosis of Klatskin tumour with the final diagnosis in 99 patients (47 men, 52 women, average age 67) treated from 1986-1989. Preoperative investigations included US and CT scans, PTC, ERCP, and angiography. A presumptive diagnosis of Klatskin tumour was made when a PTC demonstrated a focal stenosis at or near the bifurcation of the hepatic duct in the absence of: 1) previous surgery that could have produced an iatrogenic stricture; 2) CT or other evidence of a malignant neoplasm of another organ; or 3) more diffuse irregularity of the ducts as seen in sclerosing cholangitis. The intrahepatic bile ducts proximal to the lesion were usually dilated, and there were no gallstones within the ducts.

The final diagnosis was different from sclerosing adenocarcinoma of the bile duct in 33 (33%) cases. The breakdown included 66 Klatskin tumours; 8 papillary bile duct carcinomas; 12 gallbladder carcinomas invading the bile duct; 4 metastatic tumours to the bile duct; 2 cases of Mirizzi syndrome; 2 granulomas; 4 cases of idiopathic benign focal stenosis; and 1 case of granular cell myoblastoma. Patients with papillary adenocarcinomas had an extensive filling defect of the duct, which was often thought by nonsurgeons to be unresectable. However, 7 of these 8 lesions could be completed excised (including 3 hepatic lobectomies), and the neoplasm was confined to the duct wall in 6 patients, who may well be cured. The outcome of resection of the benign lesions (eg, Mirizzi syndrome, idiopathic focal stenosis, and granular cell tumour) was also good.

These findings demonstrate the pitfalls of assuming that a focal stenosis of the hepatic duct represents a sclerosing adenocarcinoma. Despite extensive investigations, the preoperative diagnosis was incorrect in a third of cases. Most importantly, in 11 patients who had curative resections strong opinions were expressed preoperatively by nonsurgeons in favour of percutaneous stenting rather than surgery because the lesion was said to be too far advanced. This was especially noted in the patients with papillary bile duct tumours whose bulky lesions produced large filling defects that belied their curability. These findings show that reports of prolonged palliation of hilar carcinomas by stenting cannot be interpreted in the absence of proof of diagnosis, and stenting without exploratory laparotomy runs the serious risk of missing a chance to resect some highly curable lesions.

FP120 CANCER OF THE MAIN HEPATIC DUCT JUNCTION

T. Tsuzuki, M. Ueda, S. Takahashi
A. Sugioka
Keio University, Tokyo, JAPAN

Between January 1973 to December 1989, 52 patients with cancer of the main hepatic duct junction were admitted and 27 underwent extensive resection of the bile ducts with hepatic resection. The whole period was divided into two periods, the first period being 1973-1980 and the second period 1981-1989. The procedures of hepatic resection in the two periods were right trisegmentectomy in 2 and 2, right lobectomy in 1 and 2, left lobectomy in 9 and 8, left medial segmentectomy in 0 and 3, respectively. Along with left lobectomies, resection of right hepatic artery and portal vein, and part of bifurcation of the portal trunk were performed in 2 and 2 in the first period and resection of right portal vein was carried out in 1 in the second period.

A variety of complications occurred in the first period. The most severe complication was sepsis which was results of anastomotic leakage, resection for infected liver and extensive wound infection due to a drug-resistant staphylococcal strain. Complications were controlled except for staphylococcus sepsis which led to a death on the 42nd P.O. day. In the second period, disseminated intravascular coagulation was the only complication that required meticulous care. No patient died postoperatively.

The 26 patients who were discharged from the hospital lived with excellent quality of life. The 5-year actuarial survival rate calculated by the Kaplan Meier method was 19% and 4 patients lived more than 5 years. These long-term survivors were characterized by curative resection that indicated clear margins of the bile ducts and removal of metastasized lymph nodes. The 5-year actuarial survival rate in this group was 35%.

Cancer of the main hepatic duct junction is now resected without grave risk and long-term survival is the issue.

FP121

### CURATIVE RESECTION FOR CHOLANGIOCARCINOMA: FACT OR FANTASY?

IS Benjamin, RH Gompertz,
RCN Williamson and LH Blumgart.

Department of Surgery, Hammersmith Hospital, London.

Of 195 patients assessed for treatment of hilar cholangiocarcinoma during a ten year period 41 (21%) underwent elective tumour resection, 20 having liver resection and 21 local resection, with a 30 day mortality of 3 (7%). Of the 21 patients undergoing local resection, surgery was known to have been palliative at the time of operation in 7, and despite frozen section control, resection margins were also found to be involved on subsequent meticulous paraffin section in a further 5 patients, one of whom has subsequently undergone liver transplantation. Although no patients were knowingly subjected to palliative liver resection, 6 had involved resection margins on paraffin section histology. Of the 36 patients surviving 3 months or more 18 had clear and 18 had involved margins. Patients with tumour involving the resection margin had an actual median survival of 12.5 months compared to 19 months for those with apparently complete resection. Actuarial survival analysis showed a significantly increased survival in the group with clear margins (p=0.023, Mantel Haenszel). Follow up of 36 months or more is available for 7 patients who had clear margins: one died of recurrent tumour at 84 months, one of unrelated causes at 60 months and the remaining 5 are alive between 51 and 67 months after surgery. Resection, where possible, offers prolonged survival and, if complete, the only hope of cure.

FP122 PROGNOSIS AND MANAGEMENT IN PROXIMAL CHOLANGIOCARCINOMA

J.Funovics, B Teleky, D.Mirza, F.Herbst, Th.Pratschner
Department of Surgery I, University Vienna

Malignancies of the biliary drainage system are rare and are associated with a poor prognosis. From 1977 to 1988 256 patients (93 males, 163 females) were operated upon at the Department of Surgery I, University of Vienna of these 153 (4o m, 113 f) for gall bladder carcinoma, and 103 (53 m, 5o f) for bile duct carcinoma, excluding periampullary and intrapancreatic common bile duct carcinoma.

The 103 patients with bile duct carcinoma underwent radical resection in 29 %, palliative surgery in 53 %, 18 % having undergone surgical exploration only. Of these 103 cases, 53 were Klatskin tumors (15 radical surgery, 33 palliative surgery, 5 explorative laparotomy), and 50 involed the common bile duct and common hepatic duct (15 radical, 21 palliative, 14 exploration only). The tumor stage-distribution was $T_1$ = 5 (4.9 %), $T_2$ = 17 (16.5%), $T_3$ = 40 (38.8%), $T_4$ = 41 (39.8%). The 30 patients, who underwent radical resection included choledochal and bifurcation resection (n = 10), extended right hemihepatectomy (n = 12) and duodeno-pancreatectomy (n=8). Postoperative complications included 2 haemorrhages, 3 biliary fistulae, 2 intrabdominal abscesses, 1 wound infection, 1 multiple organ failure, with a postoperative mortalitiy of 9 %.

The 2 year survival rate following radical surgery was 40 %, compared to 7% following palliation or no surviver after exploration ($p<0.0001$). Histology revealed well differentiated carcinoma in 44%, moderately differentiated in 49 % and poorly differentiated in 7 %. The mean survival rate after radical surgery for extrahepatic biliary malignancy being 32 months, 6.7 months after palliation and 1.4 months after explorative laparotomy only. An improved prognosis can only be achieved with early diagnosis and radical surgical resection.

### FP123 LONG-TERM RESULTS OF ADDITIVE RADIOTHERAPY AFTER NON-RADICAL RESECTION OF KLATSKIN TUMORS

P.C.M. Verbeek, D.J. van Leeuwen,
M.N. van der Heyde, D. Gonzalez Gonzalez
Academic Medical Centre, Amsterdam, NL

In the AMC the treatment of hilar (Klatskin) tumors consists of hilar resection if feasible. Because radical resection is seldom achieved, resective surgery is usually followed by adjuvant radiotherapy. We assessed the longterm results in patients treated with adjuvant radiotherapy: either external radiotherapy (45 Gy) or the combination of external (45 Gy) and internal Iridium 192 (10 Gy) irradiation.
PATIENTS Between September 1983 and January 1990 64 patients were operated with the intention to achieve local tumor control and adequate biliary drainage. Lobar liver resections were added in 20 patients. 29 Patients received postoperatively radiotherapy (8 patients external; 21 patients external and internal radiotherapy) and 22 patients were treated with resection only, three of them had a curative resection. There were 13 postoperative deaths.
RESULTS 22 Patients who were treated with resection only, had a median survival of 8 $\pm$ 1.2 months and a mean survival of 13.5 $\pm$ 3 months. The median survival of patients with additive radiotherapy was 27 $\pm$ 5.5 months and a mean survial of 30.0 $\pm$ 4.8 months. Comparison of both therapeutic modalities demonstrated a statistically significant difference in survival ($P < 0.001$) in favour of the patients who were treated with additive radiotherapy.
CONCLUSION The present data suggest that: 1. Substantial improvement in survival is achieved with additive radiotherapy. 2. Additive radiotherapy after hilar resection is well tolerated.

### FP124 INTRAOPERATIVE RADIOTHERAPY IN THE TREATMENT OF UNRESECTABLE CHOLANGIOCARCINOMA

JRT Monson, DM Nagorney, LL Gunderson, JH Donohue, JA Martenson, Mayo Clinic and Foundation, Rochester, MN 55905, USA

Cholangiocarcinoma is frequently unresectable at presentation, thus, therapeutic options are limited. We have utilized an intraoperative electron boost (IORT) in combination with external irradiation and transhepatic stenting in 13 patients with unresectable lesions (12 treated with curative intent). Unresectability was based on extraductal spread in one patient while local extension from confluence of the hepatic ducts precluded resection in the remainder. All patients except one had transhepatic stents placed radiologically prior to surgery for relief of jaundice. All received external irradiation (EI) either before (2) or after (11) the IORT (4500 to 5075 cGy in 12 of 13). Only 1 patient received 5-FU during external irradiation. The IORT dose was 2000 cGy in 11 cases, 1500 and 1900 cGy in the other 2. There were no immediate postoperative hospital deaths though all have died. Overall median survival from tissue diagnosis was 16.5 months and 18.7 months in curative group (4-61 mo). In the curative group only 3 expired at <12 mo; local failure was documented in 6 of 12 (defined only in autopsy in 3). Four patients developed gastric outlet obstruction; four suffered significant upper GI hemorrhage. All had episodes of cholangitis and tube blockage (tube changes ranged from 0 to 15 with a mean of 3.5). Median survival of these selected patients may be enhanced by combined IORT and EI but complications are significant. Future applications of this combined therapy must focus on increased local control and palliation of biliary obstruction. Options to improve local control include routine use of high dose preop irradiation, 5-FU during external irradiation and evaluation of radiation dose modifiers with IORT (sensitizers, hyperthermia, etc). Biliary decompression with surgical bypass when feasible, instead of transhepatic stenting, might achieve better palliation of obstruction.

FP125 ASSESSMENT OF LEUKOCYTE IMMIGRATION AND ACTIVATION, NECROSIS, INFECTION AND PROGNOSIS OF ACUTE PANCREATITIS BY $99^{m}$-Tc-LEUKOCYTE SCINTIGRAPHY

M.Lausen,V.Gross,[1] J.Schölmerich,[1] U.Schöffel,

C.Schümichen,[2] W.Gerok,[1] E.H.Farthmann

Chir.,Med.[1] u.Radiol.[2] Univ.Klinik, Freiburg,

West-Germany

The course of acute pancreatitis (a.p.) depends upon the extent of leukocyte stimulation (Gross 1989) and infection of pancreatic necrosis (Beger 1986). The mechanisms and effects of leukocyte activation are not exactly known, so far. It may either reflect the systemic response to other noxes or may by itself lead to complications, local or systemic.

$99^{m}$-Tc-leukocyte scintigraphy using planar and SPECT imaging technique was performed in 28 patients with a.p. in the early phase of hospitalization to demonstrate local leukocyte immigration. The findings were correlated with the serum elastase concentration, the clinical course and the local complications necrosis and infected necrosis.

Fourteen patients had a mild (group 1), 11 a severe (group 2) and 3 a lethal (group 3) course. Scintigraphy was positive in 11 patients: all patients of group 3, 6 of group 2 and 2 of group 1. Necrosis was found by contrast enhanced CT in 8 patients, 6 of them having a positive scan. 3 patients developed infected necrosis, scintigraphy being positive in 2. Sensitivity for lethal or severe course, necrosis and infected necrosis of $99^{m}$-Tc-leukocyte scintigraphy was calculated 64%, 75% and 18%, respectively, specifity 86%, 75% and 94%. Serum elastase levels were significantly higher in patients with a positive scan. (442,5 µg/l versus 280,9 µg/l).

Leukocyte scintigraphy seems to be a reliable parameter to predict the clinical course of pancreatitis. The value in clinical decision making with respect to medical or surgical therapy is limited.

References:

Beger, HG., Bittner, R., Block, S., Büchler, M., Gastroenterology 1986; 91:433

Gross, V., Schölmerich, J., Lausen, M., et al.: Dig.Dis.Sciences in press

FP126

### CALCIUM ACCUMULATION IN THE PANCREAS: A MECHANISM FOR HYPOCALCEMIA IN ACUTE HAEMORRHAGIC PANCREATITIS?

P.M. Salminen*, T. Schröder**
IV Department* and II Department** of Surgery, Helsinki University Hospital, Helsinki, Finland

Calcium changes of serum, urine, ascites and pancreatic tissue were studied in 10 rats having haemorrhagic pancreatitis (group I). Other 10 rats underwent duodenotomy and served as controls (group II). Pancreatitis was induced by infusing oleic acid (FFA) into the pancreatic duct. Serum calcium (S-Ca), ionized calcium (B-Ca++) and urinary calcium were measured 12 hours after the induction of pancreatitis or duodenotomy. Tissue calcium was determined in the pancreas. Ascites calcium was also measured in group I.

Histology of the pancreas revealed signs of fulminant pancreatitis in the group I, whereas it remained normal in group II. Serum calcium and ionized calcium were significantly lower in group I (S-Ca 1.56 ±0.14 mmol/l; B-$Ca^{++}$ 0.99±0.08 mmol/l) when compared to group II (S-Ca 1.85±0.24 mmol/l, $p<0.001$; B-$Ca^{++}$ 1.23±0.08 mmol/l, $p<0.01$). Tissue calcium of the pancreas was especially high in group I (12 583±2436 mg/g) compared to group II (291±233 mg/g). The mean calcium concentration in the pancreas was over 40 times higher in group I than in group II. High tissue calcium concentration was associated with low serum total and ionized calcium. The mean ascites calcium value in group I was 1.29±0.27 mmol/l, in urine calcium values there was no difference between group I and II.

The result suggest that hypocalcemia in acute pancreatitis may be explained by excessive intracellular calcium accumulation.

FP127 **COMPARISON BETWEEN PMN-ELASTASE, CRP, ANTIPROTEASES AND LDH FOR THE PREDICTION OF SEVERITY OF HUMAN ACUTE PANCREATITIS.**

W.Uhl,M.Büchler,P.Malfertheiner,M.Martini
and H.G. Beger.
Depts of Surgery and Gastroenterology,
Univers of Ulm,FRG

PMN-Elastase has been shown to be a very sensitive and specific marker for tissue inflammation.Preliminary data exists on its role in acute pancreatitis (1). In former studies we have demonstrated high accuracy rates for C-reactive protein and antiproteases to detect pancreatic necrosis (2).The aim ofthis study was to find out the role of serum necrosis markers in comparison to CT scanning in the clinical management of severe acute pancreatitis.

**Patients:** 129 pats. with acute pancreatitis (male 87,female 42) entered the study. The mean age was 52.4 years (range 21-82). The etiology was alcohol overindulgence in 65 (50%), gallstones in 36 (28%) and other causes in 28 patients (22%). According to our classification (imaging, intraoperative findings, histology),83 (64%) and 46 (36%) pats. suffered from interstitial edematous (AIP) and necrotizing pancreatitis (NP), respectively.Mean Ranson score was 1.4 and 4.7 in AIP and NP, respectively.

**Methods:** For two weeks after the onset of acute pancreatitis we analysed daily serum CRP, α -2-macroglobulin, α-1-antitrypsin, LDH and PMN-elastase. In addition we performed CT and ultrasound twice weekly.The data underwent computerized analyis and the overall detection rate (accuracy) for pancreatic necrosis was determined.

**Results:** Within 5 days after start of acute pancreatitis the overall detection rates for necrotizing pancreatitis were 86%, 84%, 82%. 72% and 69% using serum CRP (cut off 120 mg/l), PMN-elastase (120 µg/l), LDH (270 U/L), α-2-macro-globulin(1.5 g/l), and α-1-antitrypsin (3.5g/l), respectively. In comparison contrast-enhanced CT scan (gold standard intraoperative findings) and ultrasound reached 88% and 38%,respectively.

**Conclusions:** Because there are highly qualified serum markers which indicate necrotizing pancreatitis, such as CRP, PMN- elastase or LDH, a stepwise approach seems reasonable to stage pats. with acute pancreatitis: 1) Use serum necrosis markers. 2) If they indicate necrotizing pancreatitis use contrast enhanced CT scanning.

References:

1.Büchler,M.et al,Digestion 38,9.1987

2.Büchler,M.et al,Int J Pancreatol 1,227-235,1986

**FP128** SEVERITY PREDICTION IN ACUTE PANCREATITIS USING THE HONG KONG CRITERIA

D I Heath, C W Imrie, Department of Surgery
Royal Infirmary, Glasgow, U K.

It has recently been demonstrated that an admission blood glucose (BS) 7.4 mml/l and / or urea (U) 11 mm/l predicts a severe attack of acute pancreatitis with a sensitivity of 75% and specificity of 80.3% (1), figures comparable to the existing multifactorial scoring systems). We have examined the validity of this approach by prospectively measuring BS and U on admission and at 24 and 48 hours in 122 patients with acute pancreatitis. Patients were classified retrospectively according to outcome (94 mild and 28 severe attacks). In the severe group the mean and standard error of serum concentrations of U on admission were 6.3 mm/l (0.6 mm/l) and for BS 6.3 mm/l (0.2 mm/l) and in the mild group 5.2 mm/l (0.2 mm/l) and 7.6 mm/l (0.3 mm/l). The Hong Kong criteria distinguished mild from severe attacks with a sensitivity of 33% and specificity of 83%. Reducing the cut-off values to 4.9 for U and 7.5 for BS gave a sensitivity and specificity of 81% and 34% on admission and 65% and 77% for peak values during the 48 hours of the study. Both fell short of the 78% and 86% achieved with the Glasgow scoring system. The on-admission values were not sufficiently sensitive to be of use as severity predictors in our study population, whilst peak values entailed a similar delay to, but had poorer sensitivity and specificity than the Glasgow scoring system.

References:

1. Fan S T et al. Gut 1989: 30: 1591-5.

FP129

### PROLONGED BOLUS CT SCANNING IN ACUTE PANCREATITIS

W. Maier, M. Buechler, R. Roscher
Departments of Radiology and Surgery
of the University, Ulm, FRG

Outcome and clinical course of acute pancreatitis shows a close correlation with the extent of intrapancreatic necrosis. So, we tried to define pancreatic parenchymal necrosis by morphometry of the glandular contrast enhancement on Computed Tomography(CT) in a total of 125 patients with proved acute pancreatitis. In 62 cases of necrotizing pancreatitis the extent of parenchymal necrosis was estimated by weighing the surgically removed necrotic tissue. In 63 patients with interstitial pancreatitis the diagnosis was proved at operation (at biliary surgery) in 12 and clinically in 41.

In interstitial pancreatitis the gland showed a homogeneous enhancement with normal or slightly altered time-density-curves on CT. In minor pancreatic necrosis small enhancement defects could be demonstrated. Total necrosis of the gland was characterized by complete loss of pancreatic enhancement on CT.

In summary, differential diagnosis of interstitial versus necrotizing pancreatitis could be made with a specificity of 98% and a sensitivity of 88%.

Exact assessment of the extent of glandular necrosis on CT was possible in 83% of cases.

### FP130 THE TREATMENT OF ACUTE PANCREATITIS IN THE ICU

V.Arsenić, L.Torde, G.Latovljev,

S.Nemčev, B. Grujin, Zrenjanin

Jugoslavija

The causes of acute pancreatitis are different.We can tell for sure that it isn't a primary sickness, that is, it is caused with other moments like biliary calculosis, alcocholism, inadekcate alimentation, abuse of drugs.

In the period 1983. - 1988. we treated 62 patients in our ICU, usually admitted after the first operation.Before admittance those patients were treated on the ward of surgery.

At the period of admittance such a patient is dehidrated, tachypnoic, cyanotic, with tachycardia, often oliguric and has a lots of laboratory disturbances.

Taking care of such a patient is a great challenge for any anesthesiologist as it has disturbances in almost all the great systems - cardiovascular, respiratory, renal and coagulation.

Using the monitoring we have, and with surveillance through a long period of time we had a mortality rate of 35.5%.We are not fond of our results but we also think that in conditions we have, our results are still satisfactory.

Literature can be obtained by the author.

FP131 THE DIFFERENCE IN STRATEGY IN CASE OF ACUTE PANCREATITIS

J. Manniste, M. Eivin, P. Alas,
A. Maesalu
Republican Hospital, Tallinn, Estonia

During the last 14 years we have observed 586 patients in different stages of inflammation with non-biliary aetiology (primary pancreatitis - I group) and 514 patients with secondary biliary pancreatitis - II group. By admitting the patients the task was to determine the aetiology of pancreatitis by using investigation of anamnesis, clinical tests, US-examination, endoscopy or laparoscopy, even ERCP which decided the choice of the strategy of treatment. In I group in 83 cases (14,2%) severe pancreatogenous schock occurred. In II group such clinical symptomatology developed only in 21 cases (4,0%). In these cases a comprehensive pancreonecrosis was treated by using maximal conservative tactics, in II group an early surgical treatment was done for removal of biliary pathology which caused severe pancreanecrosis. We have worked out a schematic guide of conservative treatment for pancreatic shock, which besides the basic-treatment, we practise the method of prolonged drug instillation into the coeliac arterial branch, plasmapheresis and hemosorbtion, hyperbaric oxygenation and other specific methods of detoxication. The surgery in the late period (necrosectomy, sequestrectomy, drainage) was used by I group in 123 cases (20,9%), with mortality rate 19,5% (24 patients). In II group emergency surgery was used in 199 cases, among them direct operations on the pancreas in 47 cases. Mortality rate was 8,5% (17 patients). The prognosis of treatment in II group is better than in I group.

FP132 NECROTIZING PANCREATITIS (N.P.) TREATED WITH RETRO-ENDOPERITONEAL DRAINAGE AND LAVAGE (R.E.D.L.) (R.E.D.L.) REPORT ON 263 CASES

C.Bassi, S.Vesentini, M.Falconi, R.Girelli, F.Nifosi', G.C.Lazzarin, S.Corra', G.Coviello, D.Lombardi and P.Pederzoli.

Surgical Department-University of Verona, Verona Italy.

The retroperitoneum is the main site of N.P. through which the toxins, responsible for the systemic lesions, are reabsorbed back into the bloodstream. From 1976 to 1989, 263 pts. suffering from N.P. were submitted to the R.E.D.L. procedure with the aim to irrigate selectively all the involved areas. The post-operative perfusion was performed with 8-10 litres of hypertonic solution plus aprotinin (1.000.000 U.I.K./500 ml for 24 hs). Cholelitiasys was recognized aethiological factor in 35.4% of the cases, alcohol in 35%, and post-surgical in 11.8%. Ranson's criteria averaged 4.5 points. CT data showed glandular involvement diffuse or multiple in the 75% of the cases. Infected necrosis was found in 143 (65%) on 220 pts. studied with coltures. This group (g.) showed a mortality rate of 25.8% vs. the 10,4% of the non infected. Out of the 263 pts., 38 (g.A) were operated within 48 hs from the attack (3 pts. died, 7.9%), 64 (g.B) between 48 and 96 hs (8 pts died, 12.5%) and 161 (g.C) afted 96 hs (33 pts. died, 22.4%), 124 pts. of this group had come to our centre after having been submitted to one or more operations. Elsewhere the mortality rate related to the pts. who underwent previous surgery is 30% whereas in the pts. first opereted by us the mortality rate decrease to 7.7%. Moreover the mortality rate of the pts. we treated from the onset is of 6.6% suggesting that in the midle-severe form admission to a specialized centre should be as early as possible.

### FP133 FAILURE OF PERCUTANEOUS DRAINAGE IN SEVERE ACUTE PANCREATITIS

N. Rotman, D. Mathieu, MC. Anglade,

D. Cherqui, A. Salvat, PL. Fagniez

Hôpital Henri Mondor 94000 Créteil, France

From 1981 to 1989, 14 out of 69 patients hospitalized at our institution for severe acute pancreatitis, underwent percutaneous drainage of peripancreatic collections under CT scan guidance. There were 9 men and 5 women. Their age ranged from 28 to 61 years (mean = 46 years). The main cause of pancreatitis was alcohol (8 cases). The other causes were : gallstones (2 cases), hyperlipidemia (2 cases), post-operative (1 case), unknown (1 case). Ranson criteria were available in 10 patients (mean = 3.6). Drainage was performed as a primary treatment in 13 patients and for removal of a residual collection after surgery in one patient. Percutaneous drainage was performed during the 1st week after onset of the disease in 1 patient, during the 2nd week in 6, during the 3rd week in 3 and during the 4th week or later in 4. Pig tail drains (14F) were inserted under CT guidance through a left lateral (12 cases) a right lateral (1 case) or an anterior (1 case) approach. The duration of drainage ranged from 2 to 40 days (mean = 12d). In all but one patient the collections were infected. Percutaneous drainage was effective only in 2 patients who did not require surgery. All other patients were operated on for removal of infected necrosis. Percutaneous drainage is effective only in cases of fluid collections with minimal necrotic debris but is not sufficient to drain infected necrosis.

### FP134 OPEN TREATMENT OF ACUTE NECROTISING PANCREATITIS

RP Bleichrodt, EJ Hesselink, HO ten Cate Hoedemaker and R van Schilfgaarde, University Hospital Groningen, The Netherlands

The strategy to be followed in the treatment of acute necrotising pancreatitis (ANP) is subject to controversy. Limited necrosectomy and tubedrainage is advised by most experts. However adequate drainage cannot be achieved with tubes resulting in ongoing sepsis, as can be seen from our own results. From 1971 to 1985 patients with ANP were treated with the Lawson procedure. Residual abscesses were found in 26% and 50% of the patients died as a result of sepsis and MOF. Since 1985 we therefore treated patients with acute necrotising pancreatitis by the open method.
Patients and methods: From 1985 to 1987 9 patients (mean age 51 y.) with ANP were treated by the open method. The median number of positive Ranson criteria was 6,2 (range 5-8). A limited necrosectomy was performed after performing a transverse laparotomy. The lesser sac was packed with gauzes. Reexplorations were performed at least once a day to evacuate fluid collections and necrotic tissue.
Results: One patient who suffered ANP after cardiac surgery died as a result of MOF. At autopsy no residual abcesses were found. Eight patients survived. Complications occurred in three patients: One patient had a bleeding from the lesser sac, one had a colonic fistula and one had a duodenal fistula. The lesser sac obliterated gradually in an average period of 45 days. Transient pancreatic fistulas developed in 4 patients.
Conclusion: The open treatment of ANP guarantees optimal drainage and thereby prevents ongoing inflammation and abscess formation resulting in decreased mortality.

**FP135** ZIPPER LAPAROSTOMY IN NECROTIZING PANCREATITIS.

JL G-Sabrido,JR Polo,E Valdecantos,J Ferreiroa,LG Bayon,M Lasala,R Morales,J Calleja.HG"Gregorio Marañón".Madrid,Spain.

Undrained necrotic tissue,early recurrence of septic foci and pancreatic related infections can lead to Multiple Organ Failure(MOF) in necrotizing pancreatitis(NP).66 patients were evaluated according to APACHE II score, Ranson's criteria,bacteriology of infected focus and final outcome.Eleven patients developed a single abscess,treated by percutaneous drainage.The mortality of this group was 9%. 55 patients were operated on. The surgical procedure(drainage) was based on the provision of daily revisions at the ICU Department,leaving a cutaneous zipper laparostomy as abdominal closure.The patients were stratified in four categories according to findings at initial laparotomy:FTHP=Fulminant Total Hemorragic Pancreatitis;NIPN=Non-infected Pancreatic Necrosis;IPN=Infected Pancreatic Necrosis;MPA=Multiple Pancreatic Abscesses.

| NP Class | N'. | Ranson | APACHE II | Exp.Death | Ob.Death |
|---|---|---|---|---|---|
| FTHP | 4 | 8.5 | 36 | 75% | 75% (1) |
| NIPN | 19 | 5.7 | 24 | 40% | 10% (2) |
| IPN | 15 | 6.1 | 27 | 40% | 20% (3) |
| MPA | 17 | 5.8 | 25 | 40% | 18% (3) |

In conclusion,the mortality rate in NP can be reduced by daily abdominal revisions to achieve complete excision of infected necrotic tissue.The use of a cutaneous zipper closure toghether with epidural anestesia facilitates this approach.

**FP136** THE PREOPERATIVE APACHE II SCORE. A PREDICTOR OF MORBIDITY AND MORTALITY IN PANCREATODUODENECTOMY

M. Gagner, RL Rossi, JW Braasch
Dept. of Surgery, Lahey Clinic Medical Center, Burlington, MA USA

The pylorus preserving pancreatoduodenectomy (PPPD) carries a mortality (MORT) of <5% and a major morbidity (MORB) of <30%. The APACHE II score, a severity of disease classification comprises the sum of points given for age (A), physiological assessment (PA), and chronic health evaluation (CHE) and predicts MORT in ICU patients. The aim of this study was to evaluate the ability of the preop APACHE II score to predict MORB and MORT in PPPD. We reviewed the charts of 126 patients who had a PPPD from 01/81 to 09/89 and divided them in 3 groups according to their preop score.

| | n | MORB | MORT |
|---|---|---|---|
| LOW (0-3) | 47 | 11 (23%) | 1 (2%) |
| MID (4-7) | 57 | 14 (25%) | 3 (5%) |
| HIGH (≥8) | 22 | 13 (57%)*§ | 4 (18%)* |

*diff.from low, §diff. from mid, $p<0.05$ Fisher's exact test

The MORB and MORT is significantly higher in the high score group than in the lower groups. We also looked at the effect of CHE or/ and PA in patients <70 and ≥ 70 y.o.

| | Age <70 | | | Age ≥ 70 | | |
|---|---|---|---|---|---|---|
| | n | MORB | MORT | n | MORB | MORT |
| No CHE or PA | 41 | 9 (22%) | 1 (2%) | 12 | 2 (16%) | 0 |
| +CHE or +PA | 42 | 13 (31%) | 1 (2%) | 10 | 3 (30%) | 0 |
| +CHE and +PA | 14 | 6 (43%) | 2 (14%) | 7 | 6 (85%)*§ | 4 (57%)* |

*diff. from no CHE or PA, §diff. from +CHE or +PA, $p<0.05$ Fisher's exact test.

The MORB and MORT is significantly higher in patients with CHE and PA points. Also, the MORB doubles and the MORT quadruples when patients ≥70 y.o. have CHE and PA points. Conclusion: The preop APACHE II score correlates with postop MORB and MORT in elective PPPD. Elderly patients with CHE and PA points have a prohibitive MORB and MORT when undergoing PPPD, but when CHE and PA points are absent MORB and MORT is comparable to younger patients. It may be used by clinicians to assess the preop risk and may identify a high risk group of patients who perhaps should be considered for therapeutic alternatives.

### FP137 ALTERED FLUIDITY OF PLASMA MEMBRANES OF LIVER AND KIDNEY IN SEPSIS

M.Yoshida, J.Tanaka, J.Tamura,
K.Fujita, T.Kasamatsu, T.Tobe
First Department of Surgery, Faculty
of Medicine, Kyoto University, Japan

The present study was conducted to investigate the changes of the plasma membrane fluidity of the liver and the kidney in sepsis that is the main cause of multiple organ failure.

Male Wistar rats weighing 200-250g were used in all experiments. Sepsis was induced by cecal ligation and puncture. As a control, sham operation was performed. The time course of the plasma membrane fluidity of the liver and the cortex of kidney in sepsis or control was studied. The plasma membranes were isolated according to the method of Maeda et al. To evaluate the fluidity, fluorescence polarization was measured using 1,6-diphenyl-1,3,5-hexatriene. In order to investigate whether cell injury factor exists in blood, normal or sepsis rat serum, lipopolysaccharide and superoxide were added into the suspension of normal liver plasma membranes, and the direct effect into the membrane fluidity was studied.

The fluorescence polarization values of the liver plasma membranes were increased after cecal ligation and puncture; 0.198±0.014 (mean±S.D.), 0.210±0.011, 0.213±0.015 at 0, 48 and 72 hours later, respectively. Those of the cortex of kidney were increased in the same way. The fluorescence polarization value of the liver plasma membranes was decreased when superoxide was increased, and it was increased when the concentration of lipopolysaccharide added was increased. It was not significantly changed when normal or sepsis rat serum was added.

In conclusion; in sepsis, a derangement of fluidity of the liver plasma and renal cell membranes occurs along with time, presumably provoking an initiation of multiple organ failure.

### FP138 ALTERATIONS OF SPECIFIC METABOLIC FUNCTIONS AFTER LIVER COLD STORAGE IN ISOLATED RAT HEPATOCYTES

C VONS, S HILLAIRE, N THIBAUT, JP PEGORIER, MA IVANOV, C MELCION, A CORDIER, D FRANCO. Laboratoire de Toxicologie du Centre de Recherches de la Société Rhône-Poulenc, Vitry ; CNRS, Meudon ; Hôpital Louise Michel, Evry, France.

Metabolic activity of cultured human hepatocytes isolated from cold stored organ donor livers has been questioned. The aim of this work was to compare specific metabolic functions of rat hepatocytes isolated from fresh livers (controls: n=6), or from livers stored at 4°C during 19 hours with Eurocollins solution (Euro: n=6) or modified University of Wisconsin (MUW: n=6) solution, allowing a prolonged preservation of human liver grafts. Hepatocytes were isolated by two-step collagenase perfusion at 37°C, and assessed during the 24 first hours of culture. Were determined: 1- endogenous ketone body production (KB) and glucagon stimulation, 2- activity of 2 Cytochrome P450 monooxygenases, ethoxyresorufin O-deethylase (EROD) and pentoxyresorufin O-depenthylase (PROD). Results were as follows:

| | Basal KB production (nmoles/24h/mg prot) | Glucagon Stimulation % | Enzymatic Activities (pmoles/min/mg prot) EROD | PROD |
|---|---|---|---|---|
| Controls | 965±129 | 104 ±30 | 6.3±1.3 | 13.3±2 |
| Euro | 1059±225 | 90 ±12 | 2.6±0.2* | 7.2±0.9* |
| MUW | 1261±34 | 72.5±13 | 2.0±0.1* | 6.7±0 * |

* $p < 0.05$

Ketone body production and its stimulation by glucagon in isolated rat hepatocytes was not altered by liver cold storage. EROD and PROD activities were significantly reduced whatever the preservation solution used. Before studying metabolisms in human hepatocytes isolated from cold stored organ donor livers, the influence of cold storage on metabolisms in isolated hepatocytes should be assessed in animal experiments.

### FP139 ALLOPURINOL INHIBITS CATABOLISM OF HYPOXANTHINE AND IMPROVES RESYNTHESIS OF ATP AFTER 60 MIN OF NORMOTHERMIC ISCHEMIA IN RATS.

Witold Karwinski M.D., Odd Søreide M.D., Mikael Farstad M.D. Ph.D., Rune Ulvik M.D. Ph.D., Depts. Surgery and Clin. Biochemistry, University of Bergen, Norway.

We have previously shown that 60 min normothermic ischemia in rats is reversible. Bile excretion varies with the liver cell concentration of ATP both during the preischemic, ischemic and post-ischemic periods and seems to be a good indicator of liver function (J Surg Res 46:99-103, 1989, Eur Surg Res, in press). Ischemia is induced in the median and left lobes by clamping of the hepatic artery and portal branches. The bile duct is cannulated for sampling of bile excretion in median and left lobes. Biopsies are taken from the anterior margin of median lobe to determine concentrations of adenine nucleotides (ATP, ADP, AMP) and its metabolites hypoxanthine and xanthine using HPLC technique. In the present experiment i.v. Allopurinol was given in order to study the potential beneficial effect on liver energy status after 60 min of normothermic ischemia. GROUP I was given Allopurinol, 100 mg/kg, 50% during the preischemic period and 50% as a bolus prior to reperfusion. GROUP II was given Allopurinol 100 mg/kg as a bolus prior to reperfusion. GROUP III received Allopurinol 100 mg/kg as infusion prior to ischemia. The control group (GROUP IV) was treated by placebo. A summary of ATP concentrations during the reperfusion period is given in the table.

| | 20 min | 60 min | 120 min |
|---|---|---|---|
| ATP (umol/g) | | | |
| GROUP I | 0.85 ± 0.34 | 1.18 ± 0.45* | 1.01 ± 0.54* |
| GROUP IV | 0.56 ± 0.25 | 0.49 ± 0.22 | 0.43 ± 0.20 |

*, $P < 0.05$

ATP and bile flow in GROUP II and III did not differ from that of controls. Hypoxanthine and xanthine concentrations are currently under analyses and data will be presented. In conclusion, the timing of administration of Allopurinol seems to be important as the drug administrated in two doses, before ischemia and prior to reperfusion, was most effective in improving liver function.

FP140 ANTI-ISCHAEMIC PROTECTION OF LIVER

G V Ivanov
Tolbukhina Street 35/1-49, Novosibirsk
USSR

Activation of lipid peroxidation and pancreatic enzymes is the important point in pathogenesis of liver ischaemic damage.

It has been shown in experiment on 96 dogs that treating with the anti-oxidant a-tocopherol before the operation allows prolongation of complete safe liver ischaemia up to 30 minutes. In control groups all the animals died after 30 minutes of hepato-duodenal ligament clamping (HDL-clamping).

Local pancreatic hypothermia and treament with 5-Fu also prolong the safe period of complete liver ischaemia up to 30 minutes.

Complex anti-ischaemic measures with the use of local pancreatic hypothermia, 5-Fu and a-tocopherol allow animals to survive after 50 minutes of HDL clamping.

The anti-ischaemic measures described here have been used in patients. The maximum period of HDL-clamping during large liver resections in patients was 40 minutes.

**FP141 INTERMITTENT PORTAL TRIAD CLAMPING (PTC) LONGER THAN 90 MINUTES (min) DURING HEPATECTOMY**

D. ELIAS, E. DESRUENNES, Ph. LASSER
Institut Gustave-Roussy (IGR) 94805 Villejuif Cedex, France.

Among 174 hépatectomies for malignant tumors performed from 1983 to 1989, the last 110 were systematically performed under intermittent PTC. Each PTC lasted 20 min and there was a 5 min space of time between two PTC. At this moment the 2 raw surfaces of the liver were simply handly compressed. In 20 cases (18 %), the total duration of PTC exceeded 90 min. Etiologies and post-operative clinical and biological courses of these cases were retrospectively studied. The liver parenchyma was abnormal in 68 % of the cases (mainly post-chemotherapic fibrosis). The hepatectomies were difficult (50 % of median hepatectomy resecting segments IV, V and VIII) or the raw surfaces very large (after systematized extended hepatectomy : 7, or after more than four metastasectomies : 3). The mean duration of the intermittent PTC was 108 $\pm$ 18 min (range : 90-150 min) and in 2 patients it lasted more than 140 min). The mean blood loss was 2.3 $\pm$ 1.7 l (range : 0.8-8.8 l). There was no post-operative mortality.
**Morbidity** : Thirteen patients had uneventfull postoperative course and left the hospital on the 12.4 nd postoperative day. There were 2 pleural effusions, 1 phlebitis, 1 wound abcess and 2 subphrenic abcess (mean hospitalization duration : 14.7 days). Only one patient had a transitory hepatic failure after resection of the whole liver except segments I and II (hospitalization duration : 37 days). Biologic posoperative parameters did not show dramatic modifications.
**Conclusion** : Intermittent PTC longer than 90 min was well tolerated by the liver parenchyma during hepatectomy. It permitted to save blood loss during technically difficult resections (due to location of the tumor or/and to the abnormal texture of the liver parenchyma) or during large raw-surfaces hepatectomies. Probably the duration of this intermittent PTC should be improved over again.

### FP142 DOES THE ANDROGENIC-ANABOLIC HORMONE HELP THE REGENERATIVE PROCESS OF THE LIVER? AN EXPERIMENTAL STUDY IN RATS

E. Gadžijev

University dep. of gastroenterologic surgery, Ljubljana, Yugoslavia

The paper presents the follow up of the morphometric data and measurements of the nonspecific plasma cholinesterase (nsChE) activity after 2/3 hepatectomy (Htc) in rats treated with androgenic-anabolic hormone.

Depo-Testosterone was given to rats 14 days before Htc. 24, 48 and 72 hours after operation the animals were sacrified, blood and liver tissue samples were taken, morphometric measurements and nsChE activities were studied in the liver and plasma in experimental and control groups of animals. Changes in nsChE activity occuring at different time following Htc, a comparison between the activity in plasma and in liver homogenate, and between experimental and control group of animals were discussed.

The growth of residual liver mass was more marked in experimental rats within the initial 24 hours only, whilst after 48 and 72 hours post Htc it was more expresed in control group. During the period of three days after Htc there was a fall in the enzyme activity in plasma and rapide rise in liver homogenate. It was more evident in control group of animals.

Our results permit suggestion that in the regenerating liver the secretory function rather than the biosynthetic is the cause of slow normalisation of nsChE in plasma after Htc. It seems that androgenic-anabolic hormone in a way interfered with the regenerative processes in rat liver after 2/3 Htc.

### FP143 HEPATIC BRANCH VAGOTOMY CAN SUPPRESS LIVER REGENERATION IN RATS

M. Ohtake, T. Sakaguchi*, K. Yoshida and T. Muto
First Department of Surgery, First Department of Physiology*, Niigata University School of Medicine, Niigata 951, Japan

The role of the vagus nerve in liver regeneration after partial hepatectomy was examined by comparing the effects of hepatic branch vagotomy with those of hepatic branch sympathectomy in rats.

Under ether anesthesia, male rats weighing about 180g received selective section of the hepatic vagus branch or the hepatic sympathetic branch after partial hepatectomy (70%). The liver weight as a percentage of body weight and several plasma scores indicating liver function were estimated 0, 3, 7 and 14 days after the hepatectomy. The data were ANOVA analyzed and specific values were evaluated by Duncan's multiple range test.

The liver weight as a percentage of body weight was decreased significantly ($p<0.05$) 7 days after vagotomy compared to the controls, but no change in the percentage was observed in the sympathectomized rats. Plasma liver scores such as total protein, albumin, SGOT, SGPT, LDH, direct and total bilirubin, total cholesterol, GGTP, ZTT, glucose and insulin were unchanged; only alkaline phosphatase was reduced ($p<0.05$) 7 days after vagotomy.

These observations led us to speculate that the vagus nerve is specifically active in stimulating liver regeneration.

References:

Lamar C Jr, Holloway LS Jr. Acta Hepato-Gastroenterol 1977; 24: 7.

Kato H, Shimazu T. Eur J Biochem 1983; 134: 473.

### FP144 IMPAIRMENT OF LIVER REGENERATION BY OXYGEN FREE RADICALS

D.Foschi, L.Castoldi, M.Musazzi, A.Lesma and V.Rovati.
Inst.of Biomedical Sciences"L.Sacco"
University of Milan.Italy.

The aim of our study was to investigate the effects of oxygen free radicals generated by ischemia-reperfusion on liver hyperplasia after partial hepatectomy (PH).
**Material and Methods.**Female, S-D rats, 200 g were used.Under ether anesthesia, groups of 8-10 animals were submitted to ischemia ( 5 min ) of the hepatic artery and the portal vein or sham operation. After 60 min reperfusion, 80% PH was performed according to Higgins.The following were determined:i) H3 thymidine incorporation into DNA after 24 hours ( µCi/mg DNA);ii) liver weight (% of the preoperative value) 1,3,5 and 7 days after PH ;iii) lipid peroxidation ( thiobarbituric acid reactive substances, TBARS µg/mg) 3 days after PH. The effects of delayed (24 hours) ischemia-reperfusion (I-R), of verapamil (0.5 mg/kg i.p.), allopurinol ( 50 mg/kg/die, p.o.,starting 3 days before PH) and Superoxide-dismutase (15,000 UF/Kg i.p.) (SOD) were determined 3 days after PH by liver weight measurement and TBARS levels. The results were expressed as mean+SEM,and differences between groups were evaluated by the Wilcoxon test for unpaired data.Values with $p<0.05$ were considered significant.
**Results.**I-R significantly ( $p<0.01$) reduced H3-thymidine incorporation into DNA ( 18±4 vs 42±9 µCi/mg)24 hours after PH.The liver weight was significantly reduced by I-R in the first 5 days after PH ( - 20%, $p<0.05$). There was also a significant increase of TBARS ( 4.7±0.7 vs 2.1±0.5 µmol/mg, $p<0.05$) 3 days after operation.
Delayed I-R had no significant effects on liver weight and TBARS levels. Inhibitors of oxygen free radicals and SOD significantly prevented reduction of liver regeneration and TBARS increase.
**Conclusions.** Our results suggest that oxygen free radicals generated by ischemia-reperfusion influence the rate of liver regeneration for a short period after PH.

### FP145 EFFECTS OF ANTICANCER AGENTS ON CELL CYCLE OF REGENERATING HEPATOCYTES AFTER PARTIAL HEPATECTOMY IN RATS

J.Tamura, J.Tanaka, K.Fujita, M.Yoshida, T.Kasamatsu, T.Tobe
First Department of Surgery, Faculty of Medicine, Kyoto University, Japan

In treatment of hepatic malignancy, anticancer agents are frequently administered at perioperative period of hepatic resection as adjuvant therapy. However, the possible effects of chemotherapy on liver regeneration have not been fully investigated. The present study was aimed to investigate effects of anticancer agents on cell kinetics following partial hepatectomy. Three anticancer agents, cisplatin (1 and 2mg/kg), doxorubicin (0.2 and 0.5mg/kg), and mitomycin (0.2 and 0.5mg/kg) were administered to male Wistar rats subjected to 70% partial hepatectomy, and the effects were evaluated by total DNA contents of the liver, and flow cytometric (FCM) analysis of hepatocyte cell cycle using two color staining of anti-bromodeoxyuridine monoclonal antibody and propidium indide. Total DNA content of regenerating liver 7 days after hepatectomy showed a significant inhibition by the administration of these agents (p<0,01). In FCM study, effects of these agents on hepatocyte cell cycle were delay of the peak or decrease of the proportion of S phase hepatocytes, and/or polyploidization of nuclei, demonstrated by an accumulation of 8c and 16c nuclei in DNA histograms. The inhibition of total DNA contents was prominent in cisplatin groups. The polyploidization was remarkable in mitomycin groups. At six weeks after hepatectomy, although total DNA contents recovered to the level of control, polyploidization still remained. Serum transaminase levels were not significantly high in drug administered groups, suggesting these agents had no cytocidal effects to proliferating hepatocytes at the given dose. These inhibitory effects must be taken into consideration at the decision of dose or period of adjuvant chemotherapy, especially when massive hepatectomy is performed.

**FP146** THE EFFECT OF REGENERATING LIVER CYTOSOL, INSULIN AND GLUCAGON ON LIVER REGENERATION AFTER PARTIAL HEPATECTOMY IN THE RATS WITH LIVER CIRRHOSIS

Kuhn Uk Lee, Associate professor,
Seoul National University Hospital,
Seoul, Korea.

The regenerative activity of the liver parenchyma after partial hepatectomy was examined in twenty normal inbred male wistar strain rats and fifty five rats with cirrhotic liver.
Liver cirrhosis was tried to develop by pretreatment of phenobarbitone and intermittent oral ingestion of carbon tetrachloride ( Mclean et al 1969 ).
Regenerating liver cytosol(RLC) was prepared in the remnant liver 24 hours after two-third hepatectomy in the normal rats by ultracentrifugation (LaBreque and pesch1975).

The rats with cirrhotic liver were divied into three groups, and each groups was injected with RLC, insulin and glucagon, and saline after two third hepatectomy respectively. Also the rats with normal liver were divided into two groups, and each group was injected with insulin and glucagon, or saline after one-third hepatectomy.
The parameter for regeneration was the incorporation of $^{3}$H-thymidine into DNA of the liver cell.

In cirrhotic liver groups, $^{3}$H-thymidine uptake into DNA of the liver cell was increased by cytosol, and decreased by insulin and glucagon, but it was not statistically significant probably due to the wide range of value and the difference in the degree of liver cirrhosis.
In normal liver groups, the exogenous supply of insulin and glucagon did not stimulate but significantly suppress the rate of regeneration normally controlled by endogenous pancreatic hormones after one-third hepatectomy.

References:

Mclean EK, Mclean AEM, and Sutton PM. British J Exp Path 1969:50:502.

LaBreque DR, Pesch LA. J physiol 1975:248:273.

**FP147** THE EFFECT OF IMMUNOLOGICAL RESPONSE ON WOUND HEALING IN RAT WITH LIVER CIRRHOSIS

Toshiharu Makishima, Hideaki Saito,
Yasuhiko Morioka, First Department of Surgery,
Tokyo University, Tokyo, Japan

We examined the relation of cell-mediated immunity with wound healing in liver cirrhosis.

Liver cirrhosis was induced in male Wistar rats by a basal diet supplemented with 0.06% dimethylaminoazobenzene (DAB) over a four week period. Rats fed with a basal diet were served as control. Midline laparotomy of 2cm was performed. We counted the number of lymphocytes, and measured the collagen content and the bursting pressure of the abdominal wound. In addition, the delayed hypersensitivity response was measured using dinitrofluorobenzene (DNFB) applied to the ear.

The rats with cirrhosis showed significant decrease in the number of lymphocytes, the collagen content and the bursting pressure of the wound in comparison with the control rats. Also the rats with cirrhosis showed a depressed DNFB response. The number of lymphocytes infiltrating in the ear correlated with that of lymphocytes in the abdominal wound. There were positive correlations between lymphocyte number and collagen content in the wound. Moreover, the bursting pressure had a significant correlation with the number of lymphocyte in the wound.

The data strongly suggests that depressed cell-mediated immunity impairs would healing in cirrhosis.

References:

Lundberg C. Int. J. Tiss. Reac. 1984:6:477
S. Tajima Immunology 1985:54:57
Janet M. Peterson Surgery 1987:102:300
Rhonda S. Fishel Ann. Surg. 1987:206:25
Adrian Barbul Ann. Surg. 1989:209:479

FP148 ISOLATED LIVER PERFUSION WITH MITOMYCIN C IS EFFECTIVE IN THE TREATMENT OF LIVER TUMOURS IN RATS

A Marinelli, R Dijkstra, PJK Kuppen, CJH van de Velde.
University Hospital, Leiden, The Netherlands

The tumour tissue concentration is an important factor in the effectiveness of most cytostatic drugs in anti-cancer treatment. When maximum tolerable doses of mitomycin C (MMC) were administered with isolated liver perfusion (ILP; 4.8 mg/kg) and hepatic artery infusion (HAI; 1.2 mg/kg) a 5 times higher tumour tissue concentration could be achieved in liver metastases of the syngeneic colorectal carcinoma CC531 in WAG rats with ILP. To determine the bonus effect of this higher tumour tissue concentration, liver tumour bearing rats were randomly assigned to 5 groups: 1. control; 2. sham HAI; 3. sham ILP; 4. HAI with 1.2 mg MMC/kg; 5. ILP with 4.8 mg/kg. For each experiment 6-8 rats were used per group. In a cell kinetic study the rats were sacrificed 24 hours after treatment and tumours were prepared for flow cytometric (FCM) DNA-analysis. In a pharmacodynamic study the rats were laparotomized at day 0, 14, 28, and 42 (day of sacrifice) for sequential tumour measurement. Results: 1. Cell kinetic study:

| | G0/G1(%) | S(%) | G2/M(%) |
|---|---|---|---|
| control/sham HAI/sham ILP | 64 | 30 | 6 |
| HAI with 1.2 mg MMC/kg | 60 | 29 | 12 |
| ILP with 4.8 mg MMC/kg | 32 | 62 | 6 |

In the 4.8 mg/kg ILP group the percentage of cells was significantly decreased in the G0/G1 phase (-32%) and increased in the S phase (+32%), while in the 1.2 mg/kg HAI group the G2/M was increased significantly compared with control rats. 2. Pharmacodynamic study: 1.2 mg/kg via HAI did not result in significant tumour growth delay, while in the ILP with 4.8 mg MMC/kg group in 5 out of 7 rats no tumour could be detected on day of sacrifice. Conclusion: With ILP, in contrast to HAI, the MMC concentration in tumour tissue was high enough when the MTD was administered to block DNA synthesis and to achieve complete remissions.

**FP149** HYDATID DISEASE: SURGICAL TREATMENT IN GREECE

T.Syrakos, S.Vasdekis, A.Malakoudis, K.Papazoglou, S.Hatzikokoli-Syrakou, G.Orfanos, E. Hadjiyannakis.
2nd Surgical Dpt.,Tzanion Gen. Hosp. Pireaus,
Surgical Dpt., Gen. Hosp., Veria, Greece.

Hydatid liver disease (HD) presents with a relative frequency among the Greek rural population and there is still controversy on the type of optimum surgical procedure. The aim of this study was to present the experience of two Greek district hospitals and the 7 year follow-up (range 2-12 years) of patients with HD.

Between 1976 and 1987 ninety four patients with HD were diagnosed and operated upon. The hydatid cysts were located in the liver in 82 (87%) cases, in the kidneys in 3 cases, in the spleen in 4 cases and in the remaining 5 cases elsewhere (lungs, muscles etc.). They were all operated and a solution of 2-4% formalin was used as scolicidal agent. Operative techniques, in hepatic localisation of HD, involved omentoplasty in 24(29%), cystopericystectomy with or without liver resection in 22(27%), drainage in 18(22%), myoplasty in 8(10%), capitonnage in 8(10%) and marsupialisation in 2(2%). There were no toxic reactions from the use of formalin. Peroperative morbidity was 18% (17/94) and mortality 2% (2/94). During the seven years follow-up there were 6 liver recurrencies of the disease.

We conclude: 1.The surgical treatment of HD should be individualised according to the localisation and size of the cyst.2.The application of low concentration formalin solution as scolicidal is quite safe. 3.Long term follow-up of these patients is mandatory.

FP150 HYDATID DISEASEOF THE LIVER IN IRAQ

NAZAR H.KZZAZI, FRCS (Eng.,Ed.,G)
CONSULTANT SURGEON
AL-YARMOK TEACHING HOSPITAL
AL MUSTANSIRIYA MEDICAL SCHOOL

Forty patients with Hydtid Cyst of the liver were successfully treated by surgery in our department for the period Jan. 1985-Jan. 1989.
The age incidence ranged from 3-80 years, the M:F Ratio was 3:7
33 patients (82.5) presented with mass in the upper abdomen, 4 patients (10%) with obstructive jaundice due to rupture of the hydtid into the biliary passages, and 3 patients (7.5%) presented with the signs and symptoms of infected cyst.

The cyst affected the righd lobe of liver alone in 25 cases (62.5%), and the left lobe alone in 4 cases (10%) , and both lobes were affected in 11 cases (27.5%).
22 patients (55%) had single cyst and 18 (45%) had multiple cysts, 7 patients (17.5%) had recurant cysts.

The Diagnosis was made entirely by ultrasound scanning.
Surgical treatment, included laparatomy, killing the parasite by the injection of the cyst with 2% formaline or 70% alcohol, then incision of the ectocyst and evacuation of its contents by suction, closure of any biliary comm. unication, and treating the residual cavity by closed systen tube drain until drainage stops with occational checking of the cavity by sinography .
No specific post operative complication and no mortality was reported.
All cases of hydatid disease in Iraq are caused by Ecchinococus Granulosus.

FP151

### HEPATIC HYDATID DISEASE AND ITS SURGICAL MANAGEMENT

K. Tevfik, C. Recep, B. Settar,
T. Gündüz, Y. Kemal, SSK Ankara
Hospital, I Surgery Clinic, Ankara,
Turkey

HYDATID DISEASE due to echinococcus granulosus which mostly occupies a place in the liver still remains common in developing countries and is one of the major medical problems of our country, Turkey. At the same time, socio-economic dimensions loaded to the territory is significantly important.

In this retrospective study, we examined 181 patients with hepatic hydatid cysts treated surgically in our clinic (having 30 beds) between 1984 and 1988. 68 of 181 were men and 156 of 181 were primary hydatid cysts. We ascertained the pre-operative diagnostic methods, ways of surgical treatment, postoperative early morbidity and mortality rates, postoperative early complications and recurring rates in a follow-up period of 8 months-4 years. We discussed the medical and socio-economic dimensions of hydatid disease.

RESULTS : 27 cases (11.83) had total cystectomy, 74 (31.89) had tube marsupialisation, 90 (39.79) had partial cystectomy + capitonage, 25 (10.77) had partial cystectomy + tube marsupialisation, 10 (4.-51) had partial cystectomy + blumbage, 4 (1.78) had splenectomy, one (0.43) had left lobectomy and finally one (0.43) had fistulaectomy + tube drainage. (The numbers within parenthesis are the percentages = %). Our MORTALITY is 2 (1.10%). The recurring rate in the mentioned follow-up period is %4.41 (8 cases). Our results seem to be delightful and statistical values are significantly meaningful.

COMMENT : Hydatid disease can be evaluated by taking individual, general and environmental preventive precautions. With the ERADICATION of that disease, one major medical problem of the developing countries will be solved. Millions of dollars of economical burden which will be unloaded from the territory can not even be discussed.

KEY WORDS : Hydatid disease, Surgical management, Eradication

FP152 HYDATID CYSTS OF THE LIVER
-a surgical management of 136 cases

Pantoflicek J.,Kaspar S.,Zak R.
The Department of General Surgery,
Hospital Sokolov,Czechoslovakia.

From November 1983 to November 1987 136 patients were operated with hydatid cysts of the liver at the Department of Surgery, Regional Hospital Medea, Algeria.6o of them were males and 76 were females. The most frequent sign was upper abdominal pain 95(70%), followed by hepatomegaly 89(65%) from the 136 cases. Diagnosis was made by sonography, serology (Cassoni test) and clinical examination.

Parcial pericystectomy we used as the most frequent surgical technique in 77 cases(56%), total cystestomy in 11 cases(8%), hepatic resection (segmentectomy) in 12(9%) cases, omentoplasty in 10(7%) cases,various techniques were used in 26 cases(marsupialisation, drainage, capitonage etc). Cholecystectomy was made in 13 cases for gallstones ,common bile duct exploration was made in 7 cases, for CBD stones in 3 cases and ruptured cyst in the biliary tree in 4 cases.Peroperative cholangiography was used in 21 cases for detection of CBD stones, biliary fistulisation in cyst and bile leakage during operation.

Most common postoperative complication was biliary fistulisation in 11 cases, followed by infection complications in 9 cases and cholangitis in 4 cases.We had 1 early and 2 late postoperatives deaths for complications of portal hypertension.The average hospitalization stay was 9,3 days.

We have found the best technique partial pericystectomy , which is simple, safe and in uncomplicated cases has minimal postoperative complications.

FP153 DIAGNOSIS AND MANAGEMENT OF THE LIVER HYDATIDOSIS COMPLICATED WITH BILIARY FISTULA

Xu Ming Qian
Research Unit of Hydatidology of
The People's Hospital of Xinjiang, Urumqi
People's Republic of China

A total of 1002 patients with liver hydatidosis were treated by operations between 1953-1988. Of these, 65 cases were complicted by biliary fistula in the hydatid cyst.

The diagnosis: 1. Hydatid disease prevails in pastoral areas. 2. A history of occult pain in the liver region and sudden occurrence of cholecystalgia or obstructive jaundice, as a result of liver abscess or even AOSC. 3. Hepatomegaly and/or the hydatid cyst projecting over the costal margin may be palpable. 4. Immunological tests make a specific diagnosis. 5. Ultrasound exploration has qualitative, quantitative, localized and numerable significance. 6. Roentgenography, radioisotopic scanning, CT and PTC were assistant distinction of the pathological changes.

The management: The hydatid cyst complicated infection such as liver abscess. For AOSC drainage of purulent bile and daughter cysts and cyst fragments, incision of choledoch was preferred, followed by removal of hydatid cyst.

Management of residual cavity left in the liver:
1. Closed ectocyst suture. 2. Great omentum flap filling.
3. Closed external drainage with catheter

### FP154 SURGICAL APPROACH FOR HEPATIC ECHINOCOCCOSIS

BLIOURAS N., SKALTSAS S., FERETIS CH.,
DARAS B., KEKIS B.

ATHENS MEDICAL CENTER. ATHENS, GR.

This study was performed to determine the appropriate surgical approach for hepatic echinococcosis and the value of the antiechinococcus antibody titer in the patients with hepatic disease.

Patients and Methods: 17 patients with H.D. were subjected to exploratory laparotomy. After the cysts were located and indentified they were circularly isolated with laparotomy sponges soaked in povidone solution. The echinococcus cyst is punctured with surgiport disposable trocar manufactured by United States Surgical Corp. This disposable trocar is utilized because it gives the surgeon the capability of evacuation and lavage simultaneously using hypertonic saline solution and povidone solution because of the 2-way access by use of the stopcock.
Of the 17 patients 5 were marsupializations, 7 omentoplasties, 2 left hepatic lobectomies, and 3 cystectomies.

Results; No deaths were noted. The average length of hospital stay was 12 days. Biliary fistulas were noted in 2 patients treated with endoscopic drainage.
A decrease in the antibody titer occured in 16 patients. U/S and CT scan studies 18 months postoperatively revealed no signs of spillage contamination or recurrence of the disease.

Conclusion: In patients with H.D. there is an increased risk of spillage and recurrence during the evacuation of the cysts. The Surgiport Trocar assures "closed drainage system evacuation" with simultaneous lavage with parasitecide solution thus significantly shortening the length of the procedure.
The degree of damage is determined easier after the evacuation of the contents of the cyst, because it is easier to view possible interval communication with the intrahepatic bile ducts.
Measurments of the antibody titers pre and postop in conjuction with C/S and CT scan studies are safe criteria for the success of the procedure.
The Surgiport trocar is judged to be effective in the evacuation of the cysts without spillage and contamination of the ebdominal cavity prior to any surgical procedure performed: conservative or radical.

### FP155 MANAGEMENT OF HYDATID DISEASE : SCOLICIDAL AGENT INSTILLATION VERSUS PERI-OPERATIVE CHEMOTHERAPY

R.S. Stubbs

Department of Surgery, Wellington School of Medicine, Wellington, New Zealand

The conventional approach to preventing recurrent hydatid disease (Echinococcus granulosus) after surgery has included the use of a scolicidal agent instillation into the cyst prior to its opening. Such a practice is associated with its own risks because of the potential for all currently recommended agents to result in subsequent sclerosing cholangitis. A case involving the use of silver nitrate instillation will briefly be presented to highlight the problem. An alternative approach to preventing recurrent disease after surgery might entail the use of peri-operative albendazole therapy.

Pre-operative albendazole 400 mg twice daily was given for 4 weeks prior to surgery in 5 patients. In all instances the cyst(s) were collapsed at surgery and in only one instance was there any detectable viable hydatids found in the material evacuated. In this patient the overwhelming majority of the material was dead, but some viable protoscolices were detected. Albendazole was for this reason continued for two 4-weekly cycles post-operatively.

<u>Conclusion</u>. The recommended use of scolicidal agent instillation at the time of surgery for hydatids should be reconsidered in the light of the emergence of relatively effective peri-operative chemotherapy.

### FP156 TREATMENT OF RECURRENT HYDATID DISEASE WITH ALBENDAZOLE

T. Wilson, D. Cossetto, S. Gruenwald,
V. Antico, M. Hollands, J. M. Little.
Westmead Hospital, Sydney, Australia.

Increased morbidity and mortality associated with surgical treatment of complicated, recurrent or disseminated hydatid disease has prompted the use of chemotherapeutic agents as an aid in the management of such patients. In this study 5 patients with recurrent and 1 with widely disseminated abdominal hydatid disease caused by *echinococcus granulosus* were treated with up to six 28-day courses of **albendazole** 400 mg per oral twice daily.

Serial **ultrasound scanning** performed after each 28-day course showed disappearance of daughter cysts and cystic septation, increased echogenicity and 78 ± 26% reduction in estimated volume of the largest cyst within 2 cycles of therapy in 5 of 6 patients. 1 patient with pelvic disease extending into the buttock and ilium was followed with CT scan and did not show response until completing 4 cycles of therapy. The 5 patients with recurrent hydatid disease have shown no evidence of further cyst viability. The patient with disseminated hydatid disease appears to have recurrent activity in a retroperitoneal cyst. This has not required further treatment. The only side-effect of treatment has been transient minor abnormalities of liver function.

In conclusion, **albendazole** appears a safe and effective agent in the treatment of recurrent hydatid disease. Serial ultrasound scanning shows that most of the cysticidal activity occurs within the first 2-3 months of therapy.

### FP157 SCOLICIDAL AGENTS CAN CAUSE SCLEROSING CHOLANGITIS

A. Abbassi, B. Shadmehr, M.H.Ghaffarinejat,
P. Shishineh & F. Saidi
Departments of Surgery and Pathology,
Beheshti University of Medical Sciences,
Tehran, Iran.

In an attempt to prevent local recurrence of hydatid cysts of the liver, it is customary to inject scolicidal solutions into the unopened cyst, or instill such agents into the pericyst cavity after removal of the parasite. The clinical efficacy of such a maneuver aside, severe and fatal cholangitis has been reported, presumably the result of entry of the scolicidal agent into the biliary tree ( Khodadadi 1981, Teres 1984, and Belghiti 1986).
The local effects of four commonly used scolicidal agents have been studied experimentally by injecting into the common ducts of guinea pigs 0.5 cc aliquots of 2% (1%) formalin, 0.5 % cetrimide, 10 % hypertonic saline and 0.5 % silver nitrate solutions, using normal saline as control. Surviving animals were sacrificed at intervals of two, four, eight and sixteen weeks and their intra and extra hepatic biliary systems studied histologically. A total of 40 animals were used in each group.
The highest frequency and greatest severity of inflammatory reaction and/or late stricture formation of bile ducts was seen with formalin and the lowest with silver nitrate solutions. Hypertonic saline and cetrimide solutions were also irritating to the bile ducts, but in an intermediary range of severity.
The efficacy of any scolicidal agent must be matched against its in vivo toxicity. The preliminary injection of a scolicidal agent into a multivesicular hydatid cyst of the liver is of doubtful value as countless daughter cysts are not reached. Instillation of an agent into the evacuated pericyst cavity is more likely to prevent local recurrence of echinococcal disease. In either case, entry of a corrosive scolicidal agent into the biliary tree can damage the biliary ducts ending in diffuse and irreversible stricture formation.

Khodadadi, D. J. Int Surg 66 : 361 - 362, 1981.

Teres, J. Am J Surg 148 : 694 - 697, 1984.

Belghiti, J. Arch Surg 121 : 1162 - 1165, 1986.

**FP158** ACUTE PANCREATITIS AS A COMPLICATION OF INTRABILIARY RUPTURE OF HYDATID CYSTS OF THE LIVER

A.Agorogiannis and I. Agorogianni

Surgical Department,General Hospital of Larisa, Larisa-Greece.

During the last 12 years (from Jan 1978 to Dec 1989)at the surgical department of the General Hospital Of Larisa,we operated on 2323 patients with benign deseases of the biliary tract.Of these patients 228 (9.9%) had acute pancreatitis (AP),103 had hydatid desease of the liver and the other 2220 had gallstone desease of the biliary trct.Of the 103 patients with hydatid desease 13 presented with intrabiliary rupture (12.6%) and 4 of them (3.8%) had,as a complication of their hydatid desease,severe acute pancreatitis.To all 4 cases the AP was discovered at the time of operation which initially was intented to treat only the hydatid desease and the obstruction of the common bile duct.
The purpose of this paper is to draw attention to the unusual complication of acute pancreatitis after intrabiliary rupture in hydatid desease.The authors treated these 4 cases with deserving success,by choledochotomy,Kehr's drain,cholecystectomy,opening and evacuation of the hydatid cyst,partial cystectomy and partial closure (capitonage),omentoplasty and multiple drainage of the cyst and the peritoneal cavity.
Conclusions:The condition is very rare.The Surgeon must keep it in mind especially in areas were the echinococcus is endemic.During the last 15 years there hane been pablished 8 cases in the international literature.Whilst the essential role played by the obstruction and reflux would indeed appear to be of primordial importance,the possibility must also envisaged of the sudden and massive reflux of hydatid fluid into the common bile ductIntrabiliary rupture of the hepatic hydatid cyst results from the high-pressure gradient between the cyst and the lumen of the bile ducts (200-300 mm of water).In ruptured cysts debris will inevitably trickle down to the common bile duct and behave in the same way as gallstones.The pathophysiology of acute pancreatitis secondary to intrabiliary rupture is similar to that associated with gallstones.Spasm of the sphincter of Oddi would favourise the passage of this fluid into the duct of Wirsung,resulting in acute necrotic hemorrhagic pancreatitis.The principles of treatment are the same as in acute pancreatitis due to cholelithiasis.

### FP159 RECURRENT LIVER ALVEOLAR ECHINOCOCCOSIS (AE) DISEASE AFTER ORTHOTOPIC LIVER TRANSPLANTATION (OLT)

J Chipponi, P Lointier, J J Piech
Ch Ferrier, D Pezet, J Beytout
M Cambon, P Dechelotte
Chirurgie Generale et Digestive, Hotel Dieu, Clermont-Ferrand, France

We describe for the first time a 47 year old man with end-stage liver AE who developed recurrent disease on the liver graft 2 months after successful OLT. During surgical procedure adhesions were noted between the liver and diaphragm with AE deposits on the peritoneum and spleen. The number of blood units used was 25 and that of fresh-frozen plasma was 35. Immunosuppression was done with cyclosporine, steroids and azathioprine.

Recurrence was discovered upon CT scan examination because of hepatic pain, cholestatic jaundice. CT features were multiple pseudocystic necrotic areas in the liver and the spleen without enhancement after IV bolus. Enzyme-linked immunosorbent assay (ELISA) for E multilocularis was positive. Diagnosis was made by guided bipsy which showed typical 'parasitic granuloma'. Chemotherapy with Albendazole was given without signs of drug intolerance. Our patient is doing well with amelioration of clinical symptoms, distinct morphological changes in liver graft specimen biopsies, but insignificant CT scan changes for 7 months.

Nothing is known today about the evolution of AE residual foci of extrahepatic parasitic tissue in patients receiving immunosupressive therapy.

This case suggests that postoperative chemotherapy with Albendazole should be performed immediately after operation.

**FP160** *THERAPY RESISTENT GIARDIA-LAMBLIA CONTAMINATION OF THE BILIARY TRACT -HOW CAN A SURGEON ASSIST?*

*M. Szeleczky, I. Rózsa, T.Szécsi*

*Budapest, Hungary*

*Giardia lamblia contamination of the biliary tract is quite often found in Hungary, and the incidence is growing. Systematic treatment /i.e. per os, suppository, intravenous infusion/ relieves the infection and the symptoms in several patients only temporarily. The gallbladder is often the source of the infection in these cases. Even if no stones are detected, the removel of the gallbladder can be indicated in case of considerable bilious complaints. This procedure seems particularily justified, if repeated traditional treatment for giardiasis fails to cure the infection.Authors performed cholecystectomy for considerable bilious complaints on ten patients during the last four years, when therapy resistent Giardia lamblia contamination of the biliary tract had been proved. After cholecystectomy a thin plastic tube was inserted through the cystic duct stump into the common bile duct.100 mg Metronidazol solution,containing o,5 g of the drug, was given under pressure control through the plastic tube daily from the 1st to the 5th postoperative day.The bile samples taken before removal of the tube prooved protozoon free. On follow-up study 6 months-2 years after the operation, six of them were free of complaints and no Giardia was found in their duodenal juice.In four patients the follow-up examination is to be completed.*

FP161

### HEPATOCELLULAR CARCINOMA IN AFRICA CLINICAL PRESENTATION AETOCOLOGY AND PROBLEMS OF MANAGEMENT

R. Ndoma-Egba, University Teaching Hospital, Calabar, Nigeria

Between 1980 and 1988, a total of 65 cases of primary hepatocellular carcinoma were seen in the University of Calabar Teaching Hospital. Evaluation of these cases regarding age, sex and clinical symptoms was carried out. Results showed a male to female ratio of 2:1 with the highest incidence occurring in the age bracket 20 - 40 years. Jaundice was present in only 42% of cases while abdominal pain and abdominal mass were present in 85-90% of the patients. Determination of HBS Ag status showed serpositivity in 85% of cases confirming earlier reports (Otu, 1987).

In spite of its high incidence, the diagnosis and treatment of HPC is still rudimentary in Africa. The reasons are multifactorial viz: late presentation, problems of superstition, ignorance and poverty. Even when the patient presents early, diagnostic tools are few and far between. Lack of intensive care facilities militate against heroic surgery limiting such treatment to only palliative procedures. The urgent need for preventive measures including vaccination against Hepatitis B virus and education against multiple injections is emphasised.

In the long term, the future of improved surgical management of this condition in this sub-region would depend on the development of centres of Excellence for Hepato-Pancreato-Biliary surgery supported initially (financially and with personnel) by our more fortunate brethren from the developed world.

REFERENCE:

1. Otu A A CANCER 60: 2585, 1987.
2. EDINGTON GM, GILES HM. Pathology in the Tropics ed 2 London; Edward Arnold, 1076. 589-598.
3. DOLL R. MUIR C. WATERHOUSE J. 1970 Cancer incidence in five continents. Acta Un Int. Contra Cabcrum 2:1
4. HEPATITIS B VACCINE. Lancet 1980. 2: 1229-1230.
5. BERMAN C. 1951 Primary Carcinoma of the Liver. A study in incidence, clinical manifestations, pathology and aetiology. Lewin, London.

### FP162 RESECTION OF THE LIVER USING BALLOON CATHETER (SURGICAL ANATOMY AND TECHNIQUE)

M.Sh.Israelashvili, Sh.S.Toidze
Tbilisi State Medical Institute,
Tbilisi, USSR

In adhesion and overhanging of the tumor in the portal fissure area, exposure and ligation of lobar portal peduncles are associated with great difficulties or are sometimes even impossible (Adson,Beart; Fortner;Foster). In these cases bleeding from afferent vessels may be successfully prevented using a balloon catheter introduced into the corresponding (lobar or segmental) branch of the portal vein through one of jejunal veins. This method of liver resection was developed by us in 1976 (USSR Author's Certificate No. 534241). Clinical trials were preceded by investigation of surgical anatomy of human liver vessels (415 preparations), experiments on dogs (32 animals) and elaboration of operative technique on 27 human cadavers. Over past 15 years we performed more than 200 operations in hepatobiliary area including extensive liver resections. In 21 cases it was necessary to use the method of transportal transcatheter occlusion of the portal triad in liver resection. (All patients had topical lesions of the organ). It was established that the use of a balloon catheter in liver resection presents no technical difficulties, it facilitates temporary exclusion of hardly accessible elements of the portal triad in the portal fissure area without their exposure and, finally, it permits to define boundaries of the liver part to be removed, preventing in this way possible exclusion of vessels in the liver part to be retained.

References:

Adson M, Beart R. Surg Clin N Amer 1977: 57: 339

Fortner J, Maclean B, Kim D. et al. Cancer (Philad.) 1981: 47: 2162

Foster J. Surg Gyn Obstet 1971: 132: 693

FP163 

### LIVER RESECTION IN PRIMARY HEPATOCELLULAR CARCINOMA

**E. Galperin and M. Israelashvily**

**1st Moscow Medical Institute;**

**Tbilisi State Medical Institute**

Autonomity of blood supply of liver lobes, sectors and segments was studied in 278 human organocomplexes using the methods of corrosion, roentgenography and preparation. Principle attention was paid to the group of anastomoses involving accessorial portal vein and the gallbladder veins. On 64 human cadavers was elaborated a simplified technique for controlled liver resections with the use of finger exposure of hepatic pedicles and their rapid ligation without significant normothermal ischemia of the retained parts of the liver. Local and distant spreading of the primary liver carcinoma was analysed in 151 patients. Surgical intervention was performed in 100 patients including liver resection in 33. Hemihepatectomy (16) with cholecystectomy and dissection of fat and lymph nodes of the portal fissure and hepatoduodenal ligament proved to be radical operation. When primary carcinoma grows to segments 4th, 5th or 8th liver resection should be initiated with separation of the gallbladder from the retaining part of the organ to prevent possible dissemination of cancer cells via gallbladder veins from the tumour-affected lobe. Average life expectancy of patients after hemihepatectomy with cholecystectomy was 38.3 ± 4.6 months and significantly higher than after hemihepatectomy without removal of the gallbladder (21.7 ± 2.1 months). In five patients with marginal growth of primary carcinoma in segments 2d,3d, 6th or 7th, an atypical resection was performed as a segment- or a bisegmentectomy, with remote results comparable with those after controlled resections.

### FP164 ANGIOGRAPHIC ARTERIO-VENOUS SHUNT AND VENOUS THROMBOSIS IN THE PROGNOSIS OF HEPATOMA

YM.Shyr, CH. Su, JH. Chiang, WY. Lui
Section of General Surgery, Department of Surgery, Veterans General Hospital
Taipei, Taiwan, Republic of China

Angiograms and clinical data for 249 patients with hepatoma were evaluated, with particular attention paid to the arterio-venous (A-V) shunt and venous thrombosis (including occlusion). There were 43 cases (17.3%) with A-V shunt and 140 cases (56.2%) with venous thrombosis. A statistically significant difference in survival was noted among the unresectable patients with and without these vascular changes. Furthermore, in the resectable patients, presence of venous thrombosis also showed a poor prognosis. A-V shunt did not influence the survival in the unresectable patients with venous thrombosis, and 93.0% of A-V shunt was associated with venous thrombosis. this seemed to imply that these two vascular changes might be the same entity. In the presence of either A-V shunt or venous thrombosis, the survival rate in the surgical group was still better than that of medical groups treated by either transarterial embolization (TAE) or conservative methods. Most of the A-V shunt (81.4%) and venous thrombosis (56.4%) occurred in the diffuse type of hepatoma, and the prognosis for unresectable patients was the worst in this type, with a mean survival time of 3 months.

Since A-V shunt and venous thrombosis are factors in poor prognosis and occur frequently, careful preoperative evaluation of these vascular changes is mandatory. However, in patients with localized tumor, coexisting with such vascular involvement, surgical resection is still recommended.

### FP165 PROGNOSIS OF HEPATOCELLULAR CARCINOMA AFTER RESECTION: A WESTERN EXPERIENCE IN 131 PATIENTS

B.Ringe, C.Wittekind, A.Weimann
G.Tusch, R.Pichlmayr
Medizinische Hochschule Hannover, FRG

Patients with hepatocellular carcinoma (HCC) have been shown to benefit from partial hepatectomy. The prognosis, however, depends on the tumor stage at the time of diagnosis. In a retrospective study of 131 patients with liver resection for HCC various prognostic factors were analyzed including pathological classification according to the TNM system as introduced by the U.I.C.C..

Histological types of tumor were HCC without coexisting liver disease (69), HCC associated with fibrosis, cirrhosis or other hepatic abnormalities (42), fibrolamellar carcinoma (14), and mixed hepato-cholangiocellular carcinoma (6). The following kinds of partial hepatectomy had been performed: left or right hepatic lobectomy (56), extended lobectomy (35), anatomical or atypical segmentectomy (29), and bilobar resections (11).

The overall actuarial survival rate at 5 years was 35.8 % including a 30-day mortality of 12.2 % ( median survival 28.7 months, disease-free survival 13.3 months). Uni- and multivariate analysis identified the following factors to be significantly associated with improved survival after resection: patient´s age 30-50 years, HCC without underlying liver disease, fibrolamellar carcinoma, solitary tumor growth, unilobar intrahepatic location, absence of vascular invasion, portal vein thrombosis or extrahepatic spread (pN0, pM0), primary tumor categories pT 2/3, stage groups II/III, and curative operation (R0).

In conclusion, tumor classification as proposed by the U.I.C.C. is useful for the assessment of prognosis after partial hepatectomy. The results obtained demonstrate clearly that liver resection is the treatment of choice in selected patients with hepatocellular carcinoma.

FP166 THE OPERATIVE RISK OF MAJOR HEPATIC RESECTION IN CIRRHOTIC PATIENTS WITH HEPATOCELLULAR CARCINOMA

C SMADJA, L CAPUSSOTTI, H BOUZARI, D GRANGE, M DELLEPIANE, D FRANCO. Hôpital Bicêtre, Le Kremlin Bicêtre ; Ospedale Mauriziano, Turin, Italie ; Hôpital Louise Michel, Evry ; Hôpital Paul Brousse, Villejuif, France.

Major hepatic resection is generally considered hazardous in the management of hepatocellular carcinoma (HCC), since patients with cirrhosis are particularly prone to postoperative liver failure after hepatectomy. Between 1983 and 1989, hepatic resection was carried out on 101 patients with HCC and liver cirrhosis. Eighteen (18 %) of them had major hepatectomies. There were 15 males and 3 females with a mean age of 57 years (range 35 to 72). Seventeen patients were Child-Pugh's class A and one class B. Mean tumor diameter was 81 $\pm$ 22 mm (M $\pm$ SD), the largest one being 125 mm. According to Couinaud classification, there were 14 right hepatectomies, 2 left hepatectomies and 2 right or left hepatectomies extending to segment I. During resection, hepatic blood inflow occlusion was used in one third of the patients. Mean operative blood transfusion was 3.5 $\pm$ 2.4 units of packed red cells. There were no operative death. Four patients (22 %) developed complications. One patient required emergency reoperation at the 24th postoperative hour for a subphrenic hematoma. One patient had a self-limited external biliary fistula. One patient had a right pleural effusion and one had transient ascites. All patients were discharged from hospital.
These results indicate that major hepatic resection may be performed safely in selected patients with HCC and cirrhosis. This is most likely related to the fact that in patients with a good liver function and a large tumor, major hepatic resections remove a limited amount of non neoplastic liver parenchyma.

**FP167** RESULTS OF TREATMENT OF PATIENTS WITH HEPATOCELLULAR CARCINOMA WITH SEVERE CIRRHOSIS OF THE LIVER

M. L. Jekić. Surgical Service,
Clinical Hospital, Zemun-Belgrade,
Yugoslavia

We retrospectively classified patients with hepato-cellular carcinoma by the results of tests of 15-minute indocyanine green retention (ICGR15). Of the 101 patients with hepatocellular carcinoma admitted to our service in the past 10 years, 18 patients were assigned to group 1 (ICGR15 $<$10.0%), 40 to group 2 (ICGR15 10.1-20.0%) and 43 patients to group 3 (ICGR15 $\geq$ 20.1%). Of the total 101 patients, 55 underwent liver resection.

We regarded the cirrhosis of the patients in group 3 as severe and studied their survival rate classified by the treatment used. Of these 43 patients, 24 patients were treated by liver resection, about half of whom were treated pre-operatively by transcatheter arterial embolization (TAE). Another 3 patients were treated pre-operatively by both TAE and portal vein embolization (PVE). At 2 years, and again at 3 years, the survival rate was significantly higher when TAE was used pre-operatively than with resection alone. We think that the possibility of performing needed liver resection will be enlarged by pre-operative PVE and that liver resection can be done more safely after use of PVE in some patients.

References:

Iwatsuki S., Shaw B W., Starzl T E., :Experience with 150 liver resections. Ann. Surg. 197:247,1983

FP168

### HIGH RATE OF RECURRENCE OF HEPATOCELLULAR CARCINOMA AFTER CURATIVE RESECTION IN CIRRHOTIC PATIENTS.

J.Belghiti, O.Farges, L.Berthoux, V.Vilgrain, J.P.Benhamou, F. Fékété
Service de Chirurgie HI Beaujon 92118 Clichy France

The long term results of the surgical treatment of hepatocellular carcinoma (H.C.C.) associated with cirrhosis have been disappointing mainly because of the high rate of intrahepatic recurrence. It is difficult to separate recurrence due to inadequate resection, unrecognized multifocal tumors and new primary tumors. In order to determine if the recurrence rate of HCC might be improved by a careful evaluation of extension and a curative resection, a series of 47 patients was analyzed. This series operated on from 1982 had the following caracteristics : preoperative CT scan after intra-arterial injection of ultrafluid lipiodol showed a single tumor; the tumor was resected with a margin of > 1 cm; no residual tumor was determined by intraoperative US examination.

Cirrhosis was secondary to alcohol abuse in 18, posthepatitic B in 19, hemochromatosis in 5 and undetermined in 5. Serum alphafoetoprotein (AFP) was normal in 18 (38%). Diameter of the tumor was < 5cm in 25 (53%) and capsula formation was present in 30 (63%). Overall cumulative survival rates at 1, 2 and 3 years were 57 %, 38 % and 28% respectively. Recurrence was observed in 28 patients (60%). The probability of recurrence was respectively of 52%, 69 % and 84 % at 1, 2 and 3 years. No significant difference in recurrence was found according to the capsule formation, and the etiology of the cirrhosis. Patients with tumors > 5 cm and those with abnormal preoperative AFP serum level had a significant higher rate of recurrence than those with tumors < 5 cm and those with normal AFP serum level (p<. 05). Recurrence was confined to the liver in 21 (75%) but only 3 had a recurrence near the resected margin.

In conclusion: In cirrhotic patients, after curative resection of HCC, despite a 30% survival rate at 3 years there is more than 80% rate of tumor recurrence. The high rate of intrahepatic recurrence away from the resected margin suggest that in most of our patients, recurrence is probably due to a new cancer in the residual cirrhotic liver. Transplantation should be considered in patients with resectable tumors.

### FP169 CLINICAL FEATURES AND PROGNOSIS OF PERIPHERAL CHOLANGIOCARCINOMA

S.T. Kim, T.S. Kim, K.S. Suh

Seoul Nat'l Univ. Hosp. Seoul, KOREA

Because of the predominance of hepatocellular carcinoma compared to cholangiocarcinoma in primary liver carcinoma and the difficulty in differentiation, most of the past clinical studies of primary liver cancer did not distinguish these two types of cancer.

So we studied the clinical features and the cumulative survival rate in 34 cases of histologically proven intrahepatic peripheral cholangiocarcinoma (excluding hilar carcinoma) during 11 years from Jan. 1977 to Dec. 1987.

Of the 34 cases, 29 were males and 5 females with a male to female ratio of 5.8:1. The age distribution was from 34 years to 81 years with the peak incidence in 6th decade and the average age was 54.1 years. Alpha-fetoprotein level was not elevated in all cases except 1 case, but serum CEA level was elevated above 5ng/ml in 12 cases out of 17 cases (71%). The HBs Ag was positive in 7 cases out of 31 cases (23%) and liver cirrhosis was present in 7 cases out of 34 cases (21%). Of the 12 resected cases, clonorchis sinensis was found in 3 cases (25%) and 1 case of intrahepatic stone was found.

Of the 34 cases, curative resection was done in 11 cases, palliative resection in one case and other palliative procedure in 2 cases (operability:41.2%, resectability:32.4%). There was no operative motality.

Twenty nine patients were followed and divided into two groups, curative resection(+) and curative resection(-) group. Cumulative 5 year survival rate was 16% in overall patients and 45% in the curative resection(+) group. All the patients of the curative resection (-) group were dead within 1 year.

As a result, if the cuative resection is done, the prognosis of periphral cholangiocarcinoma will be better than hepatocellular carcinoma because of less frequent association with liver cirrhosis.

FP170 LIVERREGENERATION AFTER PARTIAL HEPATECTOMY IN MAN: A PROSPECTIVE COMPUTERTOMOGRAPHIC APPROACH

R.Maas, U.Meyer-Pannwitt*, G.Krupski, D.Henne-Bruns*, B.Kremer*
University Hospital Hamburg-Eppendorf, Dept.of Radiology and Dept.of Surgery(*), FRG.

The quality of the computed tomography (CT) scanners of the 3. or 4. generation offers the opportunity of precise estimation of livervolume in man during the process of regeneration after partial hepatectomy (PHP).

Since Nov.88 the preoperative volume of the liver of 38 patients, who where selected because of benign or malignant tumors for PHP, was measured. The patients underwent follow-up CT`s ten days, three, six and 12 months post surgery. In suspect liver lesions needle biopsies were performed. 19 patients had to be excluded for certain reasons (e.g.: despite all modern imaging methods 5 patients presented inoperable during laparotomy).

More than 81 CT`s were performed and analysed. Preliminary results reveal: unlike in the literature there is:

- no clear correlation of amount of resection and the process of regeneration in our patients
- even after extensive resection a period longer than 6 months to complete regeneration
- evidence of post-surgical alterations of the liver-parenchyma with increase of volume and loss of density lasting 10 days to 3 months.

**preliminary results in 12 patients**

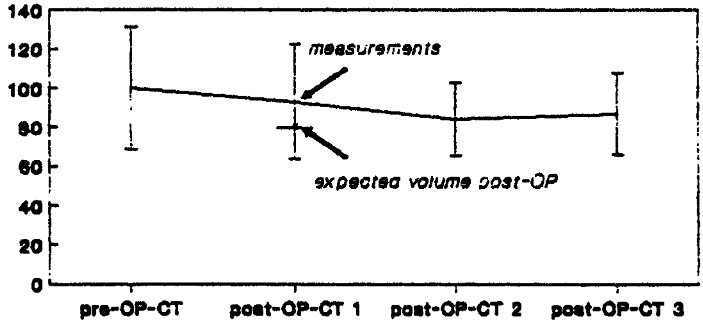

References:

1.Breimann,R.S et al:Volume determinations using computedtomography. Am. J. Roentgenol. 1982; 183:329

2.Letourneau,J.G.et al:Upper abdomrn: following partial hepatectomy. Radiology 1989; 166:139

### FP171 PANCREATIC JEJUNAL ANASTOMOSIS FOR CHRONIC PANCREATITIS.

F. Callejas Neto; E.A. Chaim; J.C. Pareja; L.S. Leonardi; I.F.S.F. Boin

Unicamp Medical School, Campinas-SP, Brazil

The authors present the results of 9 years' experience employing the pancreatic-jejunal side-to-side derivation for chronic pancreatitis on 40 patients. In 39 of these patients (97.5%) the chronic pancreatitis was associated with alcoholism.

All patients were submitted to a pancreatic-jejunal side-to-side derivation.

Only one patient (2.5%) had post-operative complication: anastomotic dehiscence with fistula. In all cases, no significant alteration were observed in the pancreatic function, and pain relief was remarkable.

Six patients had some residual pain, but it was not significant. Of these patients, two (5%) continued drinking; in two (5%) cases, ERCP diagnosed retention cysts which way not have been adequately treated during surgery; one patient (2.5%) showed common bile duct stenosis; and one (2.5%) with very mild pain which was not investigated during the post-operative period.

The mortality rate was two (5%) (respiratory failure) in patients with severe desnutrition as result of pancreatic ascites.

In conclusion, the authors recommend this surgical technique as a good option for the surgical treatment of chronic pancreatitis, with emphasis on the satisfactory results with the pain relief observed and absence of alteration in the pancreatic function.

### FP172 THE PANCREATIC DUCT OCCLUSION (PDO) WITH FIBRIN SEALANT (FS) FOR THE PROTECTION OF THE PANCREATIC-DIGESTIVE ANASTOMOSIS

H.W. Waclawiczek, O. Boeckl

Ludwig-Boltzmann-Institute for Experimental and Gastroenterological Surgery, Landes-krankenanstalten Salzburg, Austria

A dehiscence of the pancreatic-digestive anastomosis occurs even when a healthy lienal pancreas segment following head resection due to carcinomas must be anastomosed with the intestinal tract. The incidence of postoperative fistulas amounts to 5-35%. A reduction of these postoperative complications could already be achieved by PDO with prolamin (ETHIBLOC) because a quick irreversible exocrine insufficiency results and the for the healing process of the anastomosis disturbing exocrine secretion is eliminated; but there is still ongoing controversy concerning the possibility of endocrine damage with the PDO.

Therefore the goal of our experimental study in domestic pigs (n = 28) was to block the exocrine secretion only temporarily by PDO with FS in order to ensure a safe protection of the anastomosis. Our long-term studies up to 6 months postoperatively showed that a high local concentration of aprotinin (fibrinolysis inhibitor) of 20,000 I.U./ml added to the FS is necessary to block the exocrine secretion for the first 5 postoperative days. Contrary to PDO with prolamin there resulted only a lower-grade interstitial fibrosis of the exocrine tissue but no damage of the endocrine function. The healing of the pancreatic-digestive anastomoses was undisturbed.

So far this new and effective method was employed successfully in 41 patients within the scope of Whipple's operations due to pancreatic head carcinomas. No pancreatic fistula occurred, none of the patients died. In the observation period up to 3 years only 3 (7.3%) of the 41 patients became diabetic.

Therefore this method can be recommended for the protection of the pancreatic-digestive anastomosis in Whipple's operation.

FP173 NEW ASPECTS IN THE TREATMENT OF ACUTE FULMINANT NECROTIZING PANCREATITIS

A N Severtsev, E I Brekhov, I Yu Kuleshov
Surgical Clinic, Hospital No 51
Moscow, USSR

Necrotized fulminant pancreatitis which happened according to literature in 10% of patients and according to the data of our Clinic in 4% of patients is an actual and unresolved problem in modern surgery. The aim of this study is to work out and use in clinical practice the method which will allow the monitoring and adequate treatment of such patients.

The method was worked out in 1986 and was called 'Dynamic omentopancreatostomy'. The main idea of the method is to create the space ('chimney') over the pancreas. This space can easily be removed by simple manipulation, but it provides the means of escape for the exudate. At the same time the pancreas is isolated from its environment and can easily be revised. This can be done by means of a special device ['Khirurgia' (Moscow); 1988, No 12: 58 (rus.)] which is used in 'Marsupialization' of the pancreas.

From 1986 through 1988 this method was used in 12 patients. 5 of these patients developed abortive process of acute pancreatitis despite the initial severe stage of the patients. The progress of the pathological process was noted in 7 patients which required numerous sanations (up to 6) of the pancreas in all the patients. 3 patients died.

The obtained results open up great possibilities of the method in the treatment of fulminant necrotized pancreatitis.

**FP174** TREATMENT OF PANREATIC FISTULAS USING FIBRIN SEALANT.

V. Costantino, C. Pasquali, C. Sperti
F. Di Prima *, A. Alfano D'Andrea,
S. Pedrazzoli. Clinica Chirurgica 1,
Padua, & Radiology* Cittadella, Italy

Fistulas are a frequent complication in pancreatic surgery. Their treatment should include an adequate drainage, the functional suppression of the pancreas, a careful evaluation of all nutritional parameters and surgical treatment in selected cases. We performed all the above conservative techniques in order to achieve a good healing of the pancreatic fistulas we observed, and we used a human fibrin sealant to feel their tracts.
We treated 11 patients with pancreatic fistulas: 4 after pancreato-duodenectomy, 3 after left pancreatectomy, 1 following the excison of an insulinoma in the head of the pancreas, 3 after acute pancreatitis among which 1 following necrosectomy and drainage, 1 after percutaneous drainage of pseudocysts and 1 following cysto- jejunostomy. All our patients received an adequate nutritional support and had their pancreatic secretion reduced by pharmacological treatment. In addition they all underwent repeted x-ray controls in order to position as accurately as possible a proper drainage. As soon as a regular tract and a low outflow was obtained, the patient underwent sealing treatment. We used a double lumen cathether under x-ray control which allowed a selective injection of the sealant at the origin of the fistula. The tract was afterwards completely filled.
In 8 cases we obtain a good healing with a single injection. 3 cases required 2 treatments. The sealant is self-shaping and its pressure prevents the outflow of the secretions of the fistula that can therefore flow in their physiological direction. The compenents of the sealant itself, finally, support the growth of a good scar tissue. The results we obtained by this technique can be considered satisfactory as some patients recovered without undergoing a surgical treatment which would have been otherwise required.

FP175 POSTOPERATIVE COMPLICATIONS FOLLOWING COMBINED HEPATECTOMY AND PANCREATODUODENECTOMY

J.Tanaka, S.Arii, J.Tamura, M.Yoshida, K.Fujita, T.Kasamatsu, M.Imamura, T.Manabe, T.Tobe
First Department of Surgery, Kyoto University School of Medicine, Japan

Bile duct cancer has characterestics of direct invasion into liver, local lymphnode involvement, perineural invasion along vessels and intramural spreading, thereby, resulting in local recurrence by conventional radical operation. Thus, it is likely to consider that extensive operation of combined hepatectomy (Hx) and pancreatoduodenectomy (PD) is a hallmark for possible radical treatment of malignancy of biliary tract. In the present study, 16 such cases for 5 years in our department were reviewed, particularly payed an attention at postoperative complications. Eleven male and 5 female patients (age; 30-73, mean: 64y.o.) underwent combined Hx (extensive lobectomy; 8, lobectomy; 5, segmentectomy or partial resection; 3) in addition to PD. Among these cases, 4 superradical operations of en bloc resection of hepatoduodenal ligament(HDLx) followed by arterial and venous reconstruction were included. Intraoperative blood loss was 1,440-5,350g (mean; 3,320). Operative deaths were seen in 4 cases among the 16 cases, 3 HDLx and 1 lobectomy cases, of which causes of deaths were 2 liver abscess and 2 leakages of pancreaticojejunostomy, presumably the formar to be due to incomplete arterial reconstruction. Other minor complications were 6 cases of high fever suspected to be due to cholangitis, 4 wound abscess. By these radical operations, all performed operations were defined as curative operation. Almost all patients revealed an existence of fatty liver, but without major clinical symptoms. Conclusions: Since Major and life-thretening complications are seen to some extent following combined hepatectomy and pancreaticoduodenectomy, the operative indication should be carefully considered.

FP176

### PANCREATIC PSEUDOCYSTS: A PLEA FOR IMPROVED MANAGEMENT.

A. D'EGIDIO, M. SCHEIN, C.G. BREMNER
Department of Surgery, University of Witwatersrand/Hillbrow Hospital, Johannesburg, South Africa.

The developement of pseudocysts can be related to a necrotic process after an acute attack of pancreatitis ("Post-necrotic pseudocyst"), or to an obstruction of the pancreatic duct in chronic pancreatitis ("Retentional pseudocyst").
64 patients with pancreatic pseudocysts were divided in three groups. Group I: Post-necrotic cysts in acute pancreatitis (35 patients). Group II: Post-necrotic cysts in acute on chronic pancreatitis (18 patients). Group III: Retentional cysts (11 patients).
Pseudocysts were diagnosed in all patients by Ultrasonography. CT scan studies were obtained in 34 cases. E.R.C.P. was performed in all patients with chronic pancreatitis and in seven patients with acute pancreatitis. Follow-up ranged from 1 month to 76 months, with a mean of 12.8 months.
In Group I, seven patients with symptomatic immature cysts were treated by percutaneous drainage with catheter placement, without recurrence.
In Group II, 11 patients were treated conservatively and seven patients underwent internal drainage procedure, without the recurrence of pseudocyst.
In Group III, two patients were managed conservatively, internal drainage was performed in five patients, pancreatic resection in one patient and internal drainage + Pewstow operation in three patients. Recurrence of pseudocyst in three patients treated by internal drainage and in one treated conservatively, who required subsequent Pewstow operation. No recurrence and good pain control in patients treated by pancreatic resection or with the adjunct of a Pewstow procedure.
<u>Conclusions</u>: Percutaneous drainage is the treatment of choice for symptomatic acute pseudocysts. Internal drainage is adequate if there is no obstruction of the pancreatic duct. Retentional cysts may need more than just drainage alone.

### FP177 LATE RESULTS FOLLOWING SURGICAL INTERVENTION FOR CHRONIC PANCREATITIS WITH PSEUDOCYSTS

S.Karácsonyi, Gy.Farkas, Á.Pap*,

E.Szederkényi

Departments of Surgery and Medicine*

Albert Szent-Györgyi Med. University

Szeged, Hungary

Between 1982 and 1988, 257 patients were treated for chronic pancreatitis at our Department. Of the 257 patients suffering from chronic pancreatitis in 158 cases pseudocysts were found. The illnes was 3 times more frequent among man. The surgical intervention for chronic pancreatitis with pseudocyst (n=158): cysto-gastrostomy 63 (died 3; 4.4%) cysto-duodenostomy 12, cysto-jejunostomy 46, distal resection 19, proximal resection 5, external drainage 13 (died 5; 38%). Of the original 158 patients 150 survived the surgical intervention. We could check the progress of these patients over a period of one-four years. We based our investigations on the following parameters: Clinical (n=150/150): subjective complaints, change in body weight, frequency and quality of stool, ability to work. Laboratory (n=150/62): Blood sugar load, Amylo-Tolerance Test (ATT) Lipiodol Test, Secretin-Cholecystokinin Test and/or Lundh Test. 74% of them were complaint-free, while 26% had complaints of varying severity. The change in weight was about 3 kilograms. As regards ability to work 74% replied positively and 26% negatively. We analysed the changes in sugar metabolism before and after surgical intervention. We observed that after all types of surgical intervention (decompression, distal and proximal resection) the sugar metabolism deteriorated. The results of ATT dropped after both drainage and proximal resection. Similarly the lipiodol and Lundh test showed worse results after drainage while after proximal resection the results improved. After distal resection all three laboratory parameters showed a significant improvement ($p<0.01$).

### FP178 CYSTGASTROSTOMY VERSUS CYSTJEJUNOSTOMY

R.A. Prinz, K.A. Newell, T. Liu
Loyola University Medical, Maywood, IL, USA

Use of cystagstrostomy (CG) and cystjejunostomy (CJ) for treatment of pancreatic pseudocysts (PC) is often dictated by personal bias. To compare the efficacy of these two operations, 33 patients with CG were compared to 59 patients with CJ. The groups were comparable in age (CG mean 49 yrs. vs. CJ mean 45 yrs), sex (28 males, 5 females vs. 49 males, 10 females), etiology (18 alcohol, 8 biliary, 6 idiopathic and 1 traumatic vs. 45 alcohol, 4 biliary, 8 idiopathic and 2 postoperative), location (30% head, 47% body, 23% tail vs. 45% head, 32% body, 23% tail), previous pseudocysts (25% vs. 25%), symptomatology, and frequency of bacterial cyst colonization (29% positive cultures vs. 32%). Cysts treated with CG were larger (mean diameter 12.4 $\pm$ 2.9 cm) than cysts treated by CJ (mean diameter 7.2 $\pm$ 1.7 cm.) ($p < .05$). Mean duration of surgery was 150 $\pm$ 55 min. (CG) vs. 270 $\pm$ 64 min. (CJ) ($p < .05$). Mean blood loss was 350 $\pm$ 120 cc. (CG) vs. 725 $\pm$ 275 cc. (CJ) ($p < .05$) Mean blood transfused was 75 $\pm$ 55 cc. (CG) vs. 250 $\pm$ 110 cc. (CJ). Cyst recurrence was 9% (CG) vs. 7% (CG). Postoperative GI bleeding occurred in 9% of CG and 2% of CJ. Infectious problems with CG included 3 wound infections and 1 septicemia, while those with CJ included 5 intra-abdominal abscesses, 3 wound infections and 2 pneumonias. 3 patients died with CG (2 from GI bleeding and 1 from multiple organ failure), while 2 died from CJ (both intra-abdominal sepsis). CONCLUSION: CG was used in significantly larger pseudocysts and was associated with significantly less blood loss and less operating time then CJ ($p < .05$). Morbidity and mortality from CG and CJ were comparable although GI bleeding was more common with CG, and intraabdominal abscess was more common with CJ. Since CG can usually be performed more quickly and with less blood loss, it should be considered whenever anatomically feasible.

FP179 THE TREATMENT OF PANCREATIC PSEUDOCYSTS: A CHANGING STRATEGY

F. Mosca, P.C. Giulianotti, G. Sartoni, A. Guadagni, A. Campatelli, G. Di Candio, F. Farina, T. Balestracci, A. Pietrabissa

II Department of Surgery Pisa University-Italy

Much debate still exists about the proper treatment of pancreatic pseudocysts (PSC). Alternative, not surgical, therapeutical modalities are under evaluation. In this paper percutaneous ultrasound-guided drainage is compared to surgery. The limits and indications of both treatments are then discussed. Between Jan 1969 and Aug 1989, 83 pts were admitted at our institution with a diagnosis of PSC.

GROUP 1 (surgery) 53 cases (acute PSC 21, 14 M - 7 F, mean age 50 $\pm$ 18.14; 32 PSC in chronic pancreatitis, 27 M - 5 F, mean age 44.7 $\pm$ 10.16). 45.3% of PSC were located in the head of the pancreas. A significant prevalence of cephalic PSC was noted in chronic pancreatitis as compared to acute PSC. Mean PSC volume was 11.6 $cm^3$ (min 4, max 30). A complication occurred in 43.4% of pts ( infections 8, biliary stenosis 6, splenic vein thrombosis 5, choledocal fistula 1, portal vein erosion 1, aortic erosion 1, slpenic artery erosion 1). Surgical procedures were: internal drainages 11(ID), pancreatic resections 14 (PR), external drainages 28 (ED). In 39 cases addictional surgery was needed.

GROUP 2 (us-drainages) Between Oct 1982 and Aug 1989, 30 pts underwent us-drainage. 19 (63.3%) had acute PSC (11M - 8 F, mean age 52.9 $\pm$ 15.45). 11 (36.7%) had chronic PSC (10 M - 1F, mean age 49.7 $\pm$ 14.26). 20% of PSC were located in the head of the pancreas, head and body 7.4%, body 16.6%, body and tail 23%, tail 33%. Mean PSC volume was 7.4 $cm^3$ (min 4, max 14). In 10 cases the PSC was infected.

RESULTS

GROUP 1 Operative mortality rate was 3.7% and morbidity 28.3%. ED had the highest morbidity and mortality rate. Mean follow-up was 10.1 years (1pt lost, 16 pts dead for unrelated causes). 14 pts (28.5%) needed further surgery for PSC recurrence or progression of pancreatic disease. In the acute PSC subgroup results were excellent in 68%, good in 16% and bad in 16%; in the PSC in chronic pancreatitis 40% excellent , 27% good and 33% bad.

GROUP 2 There was no mortality. Morbidity rate was 10% (sepsis 1, bleeding demanding surgical exploration 1, sonographic puncture failure requiring surgical drainage 1). Mean drainage time was 64 days ( max 190 days). Mean follow-up was 2.2 years (min 0.2, max 6). In the acute PSC subgroup 2 pts had small asymptomatic PSC recurrence which did not require treatment. 2 pts had cholecystectomy. 2 pts, who had pancreatic cancer discovered after drainage, subsequently underwent duodenopancreasectomy. In the chronic PSC subgroup 2 pts had recurrence of PSC (1 underwent caudal pancreasectomy). In the acute PSC subgroup excellent results were obtained in 53%, good in 35% and bad in 12%; in the chronic PSC subgroup excellent results were obtained in 67%, good in 22% and bad in 11%. A longer follow-up is needed to definitively evaluate longterm results of percutaneous procedure

### FP180 Endoscopic Pancreas Drainage in Chronic Pancreatitis and Pancreatic Pseudocysts

M. Dohmoto, K.D. Rupp, G. Hohlbach

Med. Univers. of Luebeck, D-2400 Luebeck

Ratzeburger Allee 160, FRG

There are extremely few reports on endoprostheses inserted endoscopically for drainage in cases of pancreatitis with stenotic pancreas ducts and pancreatic pseudocysts. Workup is similar to that for endoscopic bile duct drainage. Selective transpapillary penetration of the pancreas duct is performed using a guidewire and a 7 French dilatator. The therapeutic aims for Endoscopic Retrograds Pancreas Endoprothesis (ERPEP) are similar to those of surgical intervention, i.e. elimination or reduction of pain, establishment of excretory pancreatic function and delay of necrotic inflammatory progress. During a period of 33 months 16 cases were treated by ERPEP using a pigtail catheter, with successful outcome in 11 (5 males, 6 females) ranging in age from 19 to 89. In these 11 cases chronic pancreatitis had been caused by alcoholism in 6 cases, pancreas divisum in 2 cases. All cases complained of continuous abdominal pain which increased postprandially. 11 cases of large pancreas pseudocysts were treated by transgastral (4 cases) or via papillary (5 cases) endoscopic placement of an endoprosthesis and drainage. 7 French endoprosthesis 3-8 cm in length were inserted directly into the cysts. 2 cases were performed trans-duodeno-cystotomy of pancreas head cyst using coagulator and papillotom. On the first two days after the ERPEP procedure S-amylase and U-lipase showed remarkable increases, but returned to normal within 2 weeks. One patient developed late complications due to obstruction of the endoprosthesis, but replacement was successfully performed twice in this cases.

The main indications for ERPEP are disturbance of pancreas secretion, pancreas fistula and pancreas cyst. In all cases the continuous and postprandial pain disappeared and the resulting increase in appetite resulted in weight increases of 2-13 kg. After disappearance of the symptoms and abnormal endoscopic findings after a period of 5-12 months the drainage tubes were removed. No postoperative dislodgement of prosthesis or no serious complications were encountered.

FP181 RECURRENT HEPATITIS B INFECTION AFTER LIVER TRANSPLANTATION

J.G.O'Grady, A.T.Cohen, H.Smith,
S.Davies, T.C.Tan, Roger Williams
The Liver Unit, King's College School
of Medicine and Dentistry, London, UK

Graft dysfunction as a consequence of hepatitis B virus infection caused or contributed to death in 11 of 35 (31%) patients who survived at least 60 days after liver transplantation. In most instances loss of the graft resulted from an unique clinical and histological syndrome recently described and termed by us fibrosing cholestatic hepatitis. The patient population was heterogeneous for a number of variables which could potentially influence the outcome after transplantation with respect to recurrent disease.

In the present study, univariate analysis indicated treatment with azathioprine ($11/_{25}$ vs $0/_{10}$) and the absence of co-existing delta virus infection ($10/_{24}$ vs $1/_{11}$) were significantly associated with loss of the graft. Factors which did not correlate with graft loss were the co-existing presence of hepatocellular carcinoma ($6/_{11}$ vs $5/_{24}$), status of hepatitis B virus replication prior to transplantation ($7/_{18}$ vs $4/_{17}$) or the use of hepatitis B virus immunoglobulin after transplantation ($3/_{10}$ vs $8/_{25}$). Until larger studies allowing multivariate analysis are possible we recommend that azathioprine is not included in long-term immunosuppression regimens in hepatitis B patients after liver transplantation.

### FP182 EARLY DIAGNOSIS AND SURGICAL TREATMENT OF HEPATOCELLULAR CARCINOMA IN CIRRHOSIS AND PORTAL HYPERTENSION

F. Cuan-Orozco, K.-J. Paquet, W. Rambach, Dept. Surgery, Municipal Hospital, D-6330 Wetzlar, Dept. of Surgery and Medicine, HEINZ-KALK-Hospital, D-8730 Bad Kissingen, F.R.G.

Over the last five years a policy of systematic screening for small hepatocellular carcinoma (HCC) in patients at risk has led to an increasing number of resection in patients with cirrhosis. A remarkable progress in the surgery of HCC in cirrhosis has been acomplished through (a) better understanding of the surgical anatomy of the liver, (b) the definition of new types of liver resection aimed at reducing the amount of parenchym removed by still being oncologically satisfactory, (c) the reduction of intraoperative blood loss by various technique of clamping efferent and afferent vessels, (d) the systematic use of intraoperative ultrasonography, (e) the intraoperative substitution of blood loss by preoperatively taken own blood of the patient and (f) the prevention of postoperative variceal bleeding by preoperative sclerotherapy and/or shunt operation and the formation of ascites. - From the 1st of January 1982 to the 1st of January 1989 124 patients were admitted to our hospital because of HCC in cirrhosis. 75 patients (72%) were multilocular and 26 (20%) in an advanced stage, p.i. with a lumen of at least 8cm and often localized centrally. In most of these patients a chemoembolisation was performed. 23 patients with a lumen of their unilocular tumours not more than 5cm and CHILD-PUGH A and B were considered for operation. 9 bisegmentectomies and 14 segmentectomies were performed. Main postoperative complications were the formation of ascites, bronchopneumonia and rebleeding; in one case a relaparotomy was necessary. 4 patients (16%) died; main causes of death were liver failure and sepsis. The five year survival calculated according to the method of KAPLAN-MEIER is about 50%, the ten year survival 35%. - Thus, surgical resection has become an established treatment for small HCC in cirrhosis and portal hypertension and can prolong survival.

**FP183** NEUROPEPTIDE ALTERATIONS IN THE RAT BRAIN FOLLOWING PORTOCAVAL SHUNTS

R. Rieger, W. Pimpl, C. Humpel*, C. Haring*, G. Hasenöhrl, A. Saria*

Ist Surg. Dept. and Ludwig-Boltzmann-Inst. for Experimental Surg., LKA Salzburg, and Neurochemistry Unit of Dept. of Psychiatry, University of Innsbruck, Austria

Portocaval shunts (PS) as experimental models for hepatic encephalopathy are associated with a number of metabolic abnormalities which are suspected to cause brain dysfunction. So far little is known about the effect of PS on the metabolism of regulatory peptides which can act as neurotransmitters or neuromodulators within the central nervous system.

Material and Methods:

32 Wistar rats weighing 200 to 400 g were used in this study. In 16 rats end-to-side PS were created, 11 underwent sham operations and 5 rats served as controls. 40 to 60 days after surgery the animals were sacrificed and the brains immediately taken out and dissected into 8 regions. In each region the tissue concentration of substance P (SP), vasoactive intestinal polypeptide (VIP), neurokinin A (NKA) and calcitonin gene related peptide (CGRP) was measured by RIA.

Results:

In all rats of the shunted group liver atrophy and hyperammonemia occurred. In this group all four investigated neuropeptides showed a statistically significant increase in tissue concentrations in certain brain regions when compared to the sham operated group. SP was elevated in the med. oblongata (133%), hypothalamus (241%), thalamus (193%) and striatum (132%), VIP was elevated in the hippocampus (407%) and amygdala (245%), NKA in the hippocampus (152%) and striatum (132%) and CGRP in the med. oblongata (186%) and amygdala (727%).

Conclusions:

Portocaval shunts with total deprivation of portal blood flow lead to a long term increase in the tissue concentrations of SP, VIP, NKA and CGRP in different brain regions. Association between these biochemical abnormalities and the clinical syndrome of portosystemic encephalopathy seems possible.

**FP184** ANALYSIS ON FUNCTION OF THE CULTURED HUMAN HEPATIC MACROPHAGE ESPECIALLY IN LIVER CIRRHOSIS

N.Funaki, S.Arii, K.Monden, T.Sasaoki S.Itai, Y.Adachi, H.Higashitsuji, J. Tanaka and T.Tobe
1st Department of Surgery, Kyoto University School of Medicice

The widely recognized characteristics of weakened host defense ability and altered metabolism in liver cirrhosis consist of main risk factors for surgery. The present study was aimed to investigate the pathophysiological abnormalities in liver cirrhosis as mentioned above by analyzing the function of the human hepatic macrophages(hHM) to produce various chemical mediators which are key factors for maintenance of immunoreguratory system and homeostasis. After digestion of liver specimens removed surgically (7 cirrhotic and 22 non-cirrhotic specimens) by collagenase, hHM were collected by centrifugation and purified by attachment on culture dishes. Superoxide ( $O_2^-$), prostaglandin E2(PGE2), and interleukin-1(IL-1) were measured using the culture supernatant during 1 hour incubation with opsonized symosan as stimulant. $O_2^-$ release was assayed by SOD inhibitable cytochrome c reduction method. PGE2 was measured with RIA. IL-1 activities was determined by the murine thymocyte proliferation. The results were as follows. Quantities of the released $O_2^-$ increased in dose dependent manner with the increased opsonized zymosan and significantly decreased in cirrhotic livers compared to non-cirrhotic specimens. In contrast, there were no significant differences in both PGE2 and IL-1 activities between the patients with and without liver cirrhosis, although dose dependent increases in both mediators were observed in response to increase of opsonized zymosan added to the culture. One possible reason for those insignificant changes was conspicuous individual differences.

In conclusion, hHM from cirrhotic livers showed the decreased $O_2^-$ producing activity which possibly led to weakened bacteriocidal activity, and then, primary culture of hHM developed here may be useful technology to investigate the biological significance of hHM in normal and injured livers.

FP185 HEPATOCYTE TISSUE ENGINEERING USING CONSTRUCTS OF SYNTHETIC POLYMER NETWORKS AND CULTURED CELLS

J.P. Vacanti,*+ D. Ingber,*+ L. Cima,^ J. Stein,* J. Gilbert,*
L. Johnson,*B. Schloo,# R. Langer*^
*The Children's Hospital Medical Center,+Harvard Medical School,
^The Massachusetts Institute of Technology and #The Deborah
Heart and Lung Center, Boston and Cambridge,
Massachusetts and Browns Mills, New Jersey
USA

Hepatocyte transplantation has been an attractive conceptual alternative to whole organ liver tranplantation or segmental grafting (Ebata and Mito, 1985; Demetriou et al, 1986). We have been designing man-made polymer constructs which serve as a temporary three-dimensional scaffolding to deliver large organized cell masses for hepatocyte implantation (Vacanti et al, 1988). Approaches combining cell and molecular biology, materials science, and surgical research have been developed. Polymer design parameters include configuration to keep large masses of cells alive by diffusion until neovascularization occurs, optimal cell attachment, cell shape changes to signal proliferation and differentiated function, tensile strength, and biodegradation by hydrolysis. For hepatocyte implantation we have tested many sites and are currently working with small bowel mesentery to utilize its large surface area. We have created new tissue laminates of mesenteric leaves alternating with felt-like sheets of polymer-hepatocyte constructs. To date, 200 rat implantation experiments and 900 histologic specimens have been submitted for analysis. 57 mesenteric implantation experiments have been performed. (N = 180 animals). Hepatocyte loading on polymers in culture has varied between 30 to 600 million cells per rat. (Average equals 60 to 100 million cells per rat). A 150 gram rat accepts 36 $cm^2$ of polymer material loaded with cells. Engraftment has been achieved in 96 % of cases. Histologic analysis from 5 to 97 days reveals neovascularization, histologically normal appearing nests and clusters of hepatocytes. Liver specific function has been documented in situ using immunofluorescent staining for albumin, and partial replacement of function at a distance has been observed in the Gunn rat model of glucuronyl transferase deficiency. In summary, implantation systems to generate functional new hepatic tissue using cultured cells pre seeded onto bioerodable synthetic polymer constructs have been developed. We have demonstrated long term engraftment, vascularization, and function. Further optimization is necessary to achieve sufficient mass of functional cells to cure a metabolic deficiency state. This approach may show promise not only for hepatocyte implantation, but also as a delivery system for genetically altered cells, and for replacement of other tissue types by using other cultured cell types.

References:
Ebata H, Mito M. Transplantation 1985; 39: 77-79.
Demetriou AA, Whiting JF, Feldman D, et al. Science 1986; 233: 1190-1192.
Vacanti JP, Morse MA, Saltzman WM, Domb AJ, Perez-Atayde A, Langer R. Journal of Pediatric Surgery 1988; 23: 3-9.

**FP186** VIABILITY AND TRANSPORT FUNCTIONS OF ISOLATED RAT AND HUMAN HEPATOCYTES AFTER STORAGE OF LIVERS AND ISOLATED CELLS IN UW SOLUTION

G.W. Sandker, B. Weert, M.J.H. Slooff, G.M.M. Groothuis, Dept. of Pharmacology and Therapeutics, University of Groningen, Dept. of Surgery, University Hospital, Groningen, The Netherlands. In co-operation with The Groningen Human Liver Cell Research Group

Isolated hepatocytes are a valuable tool to study liver function. In order to be able to extend experiments over a prolonged time period and in order to preserve scarce material like isolated human liver cells and to reduce the number of experimental animals, we investigated the following questions. Is the UW preservation solution suitable to preserve whole livers for cell isolation and is UW solution suitable to preserve isolated hepatocytes (rat and human) for a prolonged time period?
Rat hepatocytes were isolated directly and after 24 hours of preservation of rat livers in UW. Human hepatocytes were isolated after storage times of the whole organ up to 40 hours. Viability and transport functions of the cells were measured directly after isolation and after 18-22 hours of cell preservation in UW solution (rat and human) at 0°C. As a control, rat hepatocytes were stored for 22 hours in Krebs buffer. The viability was tested by electron microscopy, exclusion of Trypan Blue (TB) (membrane integrity) and the capacity to reduce the tetrazolium dye MTT (mitochondrial function). Transport functions were determined measuring the capacity for uptake and storage of three model compounds (taurocholic acid, vecuronium and ouabain).
Preservation of rat and human livers in UW had no influence on the yield and viability of the isolated cells. In addition, after storage of isolated rat and human hepatocytes in UW solution no change was observed in viability with TB and MTT, in morphological appearance and in transport of the model compounds. On the contrary, after storage of rat cells in Krebs buffer the viability, transport functions and the morphology of the cells were strongly deteriorated.
In conclusion: UW solution is suitable to preserve livers for cell isolation and to store isolated rat and human hepatocytes with good maintenance of viability, morphology and transport functions.

FP187 RESTORATION OF LIVER METABOLISM BY BRIEF HYPOTHERMIC PERFUSION AFTER PRESERVATION : $^{31}P$ NMR STUDIES IN RAT LIVER

A. Busza, B. Fuller, C. Lockett & E. Proctor
Academic Departnents of Physics applied to Surgery and Surgery, Royal College of Surgeons & Royal Free Hospital Medical School, London, UK.

Cold preservation of livers for transplantation is routinely achieved by flushing the organ with a chilled synthetic solution and packing in ice. We have previously studied the loss of liver ATP, fall in tissue pH and degradative dephosphorylation during storage in rat liver using $^{31}P$ NMR spectroscopy ( Fuller <u>et al</u>, 1988 ). We have now applied the technique to investigate whether brief <u>in vitro</u> reperfusion can restore liver metabolism with the aim of improving early hepatic function following transplantation.

Livers were flushed as described ( Fuller <u>et al</u>, 1988 ) and placed on a continuous perfusion circuit, designed to allow controlled perfusion at 8–10° C within the vertical bore of an 8.5 T magnet attached to a Bruker AM 360 spectrometer. Initial spectra for ATP plus ADP, phosphomonoesters, inorganic phosphate (Pi) and hepatic pH were recorded using an Haemaccel-based perfusate. The livers were removed and stored in ice for 24 hours. ATP plus ADP fell to 0, Pi increased (x5) and pH fell. During 30 minutes cold perfusion, pH returned from 6.9 to 7.3, ATP recovered from 0 to 85% and Pi fell from 500 to 120% of pre-storage values ( means for n=5 ). This shows that (1) it is possible to repair preservation damage by brief reperfusion and (2) this may yield a predictive test for function of stored livers in future. Other studies are underway to assess restoration of metabolism using different storage solutions.

Fuller B, Proctor E, Busza A <u>et al</u>. Transplantation 1988:45:239.

### FP188 LIVER ISCHEMIA FOR HEPATIC RESECTION: WHERE IS THE LIMIT ?

C.Huguet,A.Gavelli,P.Addario,S.Bona,M.Lasserre
Princesse Grace Hospital, Principality of Monaco

The concept of high sensitivity of the liver to normothermic ischemia has been challenged in several recent reports, but the upper limit of tolerance is still unknown.
Since June 1984, we performed 45 hepatic resections with the use of portal triad clamping (25 cases) or hepatic vascular exclusion (20 cases). Mean duration of continuous vascular clamping was 42 ± 15 min.(±sd)(range 15-75 min). Patients were divided into 3 groups depending on the duration of liver ischemia: group A = < 30 min. (mean 21 min.; 9 cases), B = 30-59 min.(mean 41 min.; 27 cases) and C = ≥ 60 min.(mean 65 min.; 9 cases). Major hepatectomies accounted for 44% in group A, 70% in group B and 89% in group C. Serum levels of transaminases, bilirubin, alkaline phosphatase, proteins and prothrombine were compared preoperatively and on the 1st, 2nd, 7th and 14th postoperative day. Percutaneous liver biopsy has been performed 3 to 9 months after operation in 4 pts. belonging to group C.
Overall operative mortality rate was 6.7%, and major complications occurred in 14/45 pts. (31%). All deaths were recorded in group B. Morbidity rates were 11% (group A), 33% (B), 44% (C) and mean hospital stay after surgery was of 18, 20 and 22 days respectively. Comparing only major hepatectomies, morbidity was 35% in groups A+B and 38% in C. The mean duration of operation and the amount of blood transfusions were respectively 236, 267, 317 min. and 1030, 960 and 2390 ml. in the three groups. No significant difference was found in liver function tests, except for prothrombinemia which was significantly lower only on the first postoperative day in group C (54 ± 10%) compared to groups A (79 ± 19%) and B (77 ± 18%), ($p < 0.005$). All four biopsies performed in pts. submitted to ischemia of more than 60 min. showed no chronic damage to the liver.
Biologic and histological data support that prolonged hepatic ischemia does not affect short term outcome of hepatectomy. Morbidity is probably related to the difficulty of hazardous extended resections requiring more blood transfusions rather than to the duration of vascular clamping itself. Though the limit of tolerance of human liver to normothermic ischemia is yet not known, vascular occlusion beyond 1 hour is still compatible with normal liver regeneration and survival in our experience.

### FP189 TEMPORARY HEPATIC DEARTERIALISATION IN THE TREATMENT OF HEPATIC METASTASES FROM MALIGNANT CARCINOID TUMOURS

M C ALDRIDGE, D CASTAING, H BISMUTH

Unite de Chirurgie Hepato-Biliaire,

Hopital Paul Brousse, 94800 Villejuif, FRANCE.

Between 1977 and 1989, 24 patients with hepatic metastases from malignant carcinoid tumours underwent surgical treatment and in 20 this consisted of temporary hepatic dearterialisation and chemotherapy (Streptozotocin + 5FU). The patients comprised 12 men and 12 women with a median age of 59 yr (range 28-71 yr). The location of the primary tumour was: small bowel in 11 (46%), pancreas in 5 (21%), lung in 4 (17%) colon or rectum in 2 (8%) and unknown in 2 (8%). 5HIAA and serotonin were elevated in 13 patients (54%) and 11 suffered from the carcinoid syndrome. Dearterialisation was performed by division of all arterial attachments to the liver and placement of a sling around the hepatic artery. Temporary dearterialisation was performed at monthly intervals by occluding the hepatic artery for 16 hrs followed by 5 days of intravenous chemotherapy.

Operative mortality (at 30 days) was 10% (2/20). The effect of dearterialisation was evaluated in 15 patients (3 followed-up for less than 6 months were excluded). At 6 months, there was a reduction in the volume/number of metastases in 6 (40%) patients and stabilisation in 4 (27%). A benefical effect was seen, therefore, in 67% of patients at 6 months and in 7 patients (47%) at 12 months. Following dearterialisation, carcinoid symptoms disappeared in 5/7 symptomatic patients (71%). 5 HIAA and serotonin were elevated in 11 of the 15 patients and decreased post-operatively in 6 (54%) becoming normal in 2. Survival at 5 years was 62.8% and 9/15 patients are still alive between 1 and 11 years after the diagnosis of metastases. Temporary hepatic dearterialisation is an effective treatment for carcinoid liver metastases but is associated with an operative mortality of 10% and long-term survival is reduced in patients with peritoneal deposits or cardiac involvement.

**FP190** FINE FOCUSED PROBES FOR SPARK-GENERATED ELECTROHYDRAULIC LITHOTRIPSY (EHL) OF BILIARY STONES - AN *IN VITRO* STUDY.

SEJ Edmunds, AA Santos, ML Wilkinson

Gastroenterology Unit, UMDS

Guy's Hospital, London, SE1 9RT, UK

The large common bile duct (CBD) stone remains a therapeutic challenge to the interventional endoscopist. Endoscopic sphincterotomy and Dormia basket clearance fail to remove 8 - 15% of such stones (Katon 1988). Spark-generated EHL probes have been used to fragment CBD stones both *in vivo* and *in vitro*, but require accurate positioning by incorporation into a basket or balloon catheter (Sievert 1987). We have evaluated an ACMI AEH-2A EHL unit, with focused fine probes (5F) suitable for use in a "Mother-baby" duodenoscope system under direct vision, for the fragmentation of biliary stones *in vitro*. METHODS: Seven CBD stones (0.16-5.56g) and 34 gallstones (0.52-14.11g; 18 cholesterol, 9 pigment and 5 mixed type) were assessed. Successful fragmentation was defined as a >50% size reduction. RESULTS: All CBD stones and 32 gallstones were fragmented, power range: 9-246J (median 45J). The 2 failures were 6.32g and 10.28g cholesterol gallstones. Lithotripsy energy correlated with stone size for CBD stones ($p<0.005$, $r=0.93$), all gallstones ($p<0.05$, $r=0.35$), cholesterol gallstones ($p<0.005$, $r=0.63$) and mixed stones ($p<0.005$, $r=0.56$), but not pigment-only stones. Three probes were used, lasting for a mean 1828J. CONCLUSION: Fine calibre EHL probes are effective in fragmenting a wide range of biliary tract stones. The size of cholesterol containing stones and CBD stones predicts the energy required for fragmentation.

References:

Katon RM. Gastrointest Endosc 1988; 34: 281-282.
Sievert CE. Gastrointest Endosc 1987; 33: 233-235.

### FP191 RECURRENT GALLSTONES FOLLOWING NON-SURGICAL TREATMENT: RISK, CHARACTERISTICS AND TREATMENT IMPLICATIONS

M.L. Petroni, P.M. Goggin, A. Lanzini, D. Facchinetti, K.W. Heaton, T.C.Northfield. St. George's Hospital, London, UK; University of Brescia, Italy ; Royal Infirmary, Bristol, UK.

Gallstone recurrence is the biggest barrier to acceptance of non-surgical treatment. The aims of this study were to assess the size of the risk and the characteristics and natural history of the recurrent stones in order to determine the most appropriate retreatment regimen. We analysed results from 100 patients (84F, 16M) with confirmed gallstone dissolution during bile acid therapy, followed for up to 13 years. Actuarial recurrence rate was 46% at 6 years and remained constant therefore. Recurrent stones were radiolucent and in functioning gallbladders in 90% of cases. 84% of primary and 91% of recurrent stones were multiple; and those who had multiple primary stones were likely to have multiple recurrent stones ($p<0.05$).The diameter of 83% of the recurrent stones was < 6 mm at diagnosis, by comparison with 50% of the primary stones ($p<0.05$). In 11/39 cases, recurrence was accompanied by biliary symptoms despite early re-treatment, and in seven of these the symptoms occurred within 6 months of the diagnosis of recurrence. We conclude that recurrent stones require early retreatment because a high proportion develop symptoms at an early stage; that 90% are suitable for non-surgical retreatment; and that bile acid therapy alone is the most suitable treatment, without the need for lithotripsy, because the majority are multiple and small.

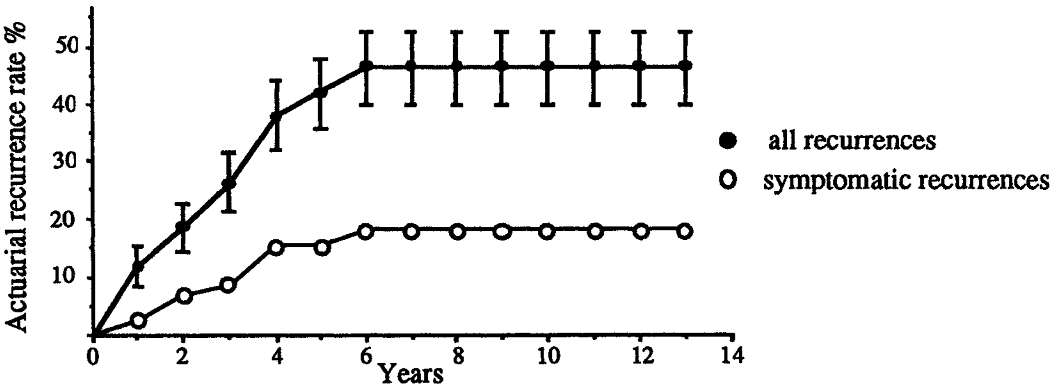

FP192 LAPAROSCOPIC CHOLECYSTECTOMY

JM Sackier, G Berci, EH Phillips,
M Paz-Partlow
Cedars-Sinai Medical Center
Los Angeles, CA, USA.

Non-surgical techniques have been introduced in an attempt to replace cholecystectomy as the treatment modality for gallstones. All aim for reduced morbidity but in none is the gallbladder removed. Utilizing special equipment, it is now possible to perform cholecystectomy with vision provided by the laparoscope connected to a colour TV camera and video monitors. It is a safe technique when thin, elective cholelithiasis patients, who have not undergone previous upper abdominal surgery, are chosen. The cystic artery and duct are secured and divided between hemoclips and cholangiography is performed routinely.

The advantages to the patient are earlier mobilization, less post-operative discomfort, earlier return to work and better cosmetic results. There is no ileus nor basal atelectasis, and many patients do not require any post-operative analgesia at all. To date, 122 patients have been treated with this technique. An additional two patients were considered for laparoscopic cholecystectomy (L.C.), but were converted to open surgery due to anomalous anatomy and unsuspected common bile duct stones. One patient developed acute pancreatitis after L.C. probably due to synchronous ERCP and another had a bile leak noted and dealt with immediately by open surgery. In one patient, it was possible to remove common duct stones through a choledochoscope introduced percutaneously into the common duct via the cystic duct. Mean length of stay was 1.5 post-operative days (range 1-3). We believe this new technique will become the procedure of choice for the selected patient, but it is vital that proper training and peer review be instituted.

FP193

### THE FIRST RESULTS OF EXPERIMENTAL LIVER TRANSPLANTATION WITH HTK PROCURED ORGANS IN THE PIG

J ERHARD(1) W NIEBEL(1) R LANGE(1) R SCHERER(2) A MARICHAL(2) R ZOTZ(3) FW EIGLER(1) HJ BRETSCHNEIDER(4)

(1)Dep. of General Surgery,(2)Inst. of Anesthesiology,(3)Dep. of Gastroenterology, University clinic Essen, W. Germany
(4)Inst. of Physiology, University clinic of Göttingen, W. Germany

Since February 1987 we perform an experimental course of orthotopic liver transplantation in the miniature pig. In 47 cases we transplanted the liver after a procurement with the HTK solution of Bretschneider, Göttingen (Fa. Köhler-Chemie, Alsbach, W. Germany).METHOD: We operated on the donor in general anesthesia. The isolated in situ organ perfusion was performed pressure controlled via the hepatic artery and the portal vein. The perfusion-quantity ranged from 100-300 ml/kgbw.cold solution.After the varied cold storage time the recipient operation was done with a veno-venous shunt.In group I (pig 1-10) we used a short preservation time to a max. of 10 hours. In group II (pig 11-30) we prolonged the preservation time to a max. of 24 hours. GroupIII (pig 31-40) consisted of organs with a preservation time to a max. of 40 hours, and group IV consisted of organs with variables in prefusion technique and preservation time (min.13, max. 24 hours). Beside the survival time we investigated the parameters of the liver-function histological examination of the liver tissue and blood tests.RESULT: Depending on the quality of the perfusion technique and the time of second warm ischemia a short survival-time (to 24 hours without of any intensive care in the postoperative period) was to attain in the 40 hours group. In the group II the rate of long term survivors was 30% (more than 48 hours). In group I and II the results of active liver-function tests and blood tests demonstrated a functionating organ. In group III all pigs died of liver failure later on whereas the tests proved 1 hour after the reperfusion a moderate but sufficient organ-function.CONCLUSION: The HTK solution is suitable for a sufficient liver procurement in the pig model to 24 hours and more. The success of long term surviving depends on multiple factors just as intensive care in the postoperative period. Even in the group with a 40 hours ischemic time of the liver the function tests prove a sufficient organ function.

FP194 HEPATIC PRESERVATION: COMPLETE OR PARTIAL ARREST OF METABOLISM

G.M. Ehrenfeld, M. Zaima, H.-J. Klomp,
K. Bhatt, N. Chandel, C.E. Broelsch
Chicago, USA

As the ability to treat neoplastic and congenital diseases increases, greater demand is placed on hepatic preservation. In instances of exclusion and donor procedures, the liver is subjected to disparate stressors. While significant progress has been made in the domian of organ preservation[1] , the mechanism of action of seemingly vital ingredients are poorly defined. Furthermore, the use of such preservation agents for short periods of time is illdefined.

Owing to the central role of carbohydrates in hepatic metabolism we have investigated the substitution of rafinsoe, in a modified $Na^+$UW solution, by mono-, di- and triose sugars, known to participate in intermediary metabolism. In the isolated perfused rat liver, at 24 hours of cold storage, resultant $VO_2$, LDH release, pH changes and energetic state paralleled that observed for comercial UW solution.

Whether raffinose or other carbohydrates function passively, as a colligative agent to balance $H_2O$ and ion fluxes, or actively, as a progenitor metabolite is unclear. Further studies are being conducted to elucidate the mechanism of action of such components.

References:

1. Jamieson, N.V.; Lindell, S; Sundberg, R.; SOuthard,J.H. Belzer, F.O. 1988, Transplantation, 46,, 512-516.

### FP195 THE AFFERENT CIRCULATION, ULTRASTRUCTURE AND INTRACELLULAR ELECTROLYTE CONTENT OF THE TRANSPLANTED LIVER PRESERVED BY P.A.P. METHOD

F. Jakab, Z. Ráth, M. Balázs , I Sugár, T. Glasz. Department of Surgery of St John's Hospital, Semmelweis Medical University, Department of Morphology, Budapest, Hungary

The relationship between the changes in portal and hepatic arterial blood flows is a much disputed question. In the practice of trans-plantation the alteration of liver blood flow can be evaluated only in connection with the ultrastructure of the transplanted liver and with the changes of the intracellular electrolyte contents in the liver. In the present experiments liver transplantations were carried out on mongrel dogs by Starzl's method. The donor liver was preserved by PAP (Portal versus Arterial Perfusion) method. The circulation of hepatic artery and portal vein was measured by Hellige electromagnetic flowmeter before removal of the recipient's liver and after the completion of all vascular anastomoses of the newly inserted liver, e.g. in the recirculatory phase of the OLTX specimens were taken for the study of ultra-structure and for the determination of cellular ion content by energy dispersive X-ray micro-analysis.

The control hepatic artery flow (HAF) was 241+23 ml/min. the average portal vein flow (PVF) was 517±47 ml/min. before the removal of the recipient's liver. In the recirculatory phase of OLTX the HAF increased to 414+39 ml/min. by 71±12% (p<0,001). The PVF decreased by - 40,2+3,5%(p<0,001). The toal hepatic inflow (HBF) showed a small, but not significant decrease during the recirculation.

In the recirculatory phase of OLTX mainly "endotheliitis" damage of endothelial and Kupffer cells, sinusoidal fenestration can be observed. The Chloride ion content of liver cells increased, the Potassium level after transient decrease returned to normal value, the Sulphur content transiently decreased. The Calcium ion - according to indirect evidences - increased in the liver cells. The exact mechanism of the electrolyte content changes is not clear.

### FP196 EXPERIMENTAL REDUCTION HEPATECTOMY WITH THE ARGON BEAM COAGULATOR

S. Stylianos, M.A. Hoffman, R.J. Rohrer, N.N. Jacir,
I. Bhan, B.H. Harris
Tufts University, The Floating Hospital and the
Kiwanis Pediatric Trauma Institute,
Boston, Massachusetts USA

Continuing shortage of suitable organs for pediatric liver transplantation has led to techniques for ex-vivo reduction of adult-size donor grafts. Increased operating time, blood loss after engraftment, and the frequency of septic complications limit the clinical usefulness of these techniques. This study reports use of the argon beam coagulator for hemostasis and minimizing tissue destruction at the cut surface of reduced-volume hepatic grafts in both experimental and clinical settings.

In this pilot study, pigs weighing 50-60 kg underwent donor hepatectomy by standard procurement techniques. The livers were reduced to 25% of the original volume by ex-vivo extended right hepatectomy following hilar dissection. The entire raw surface was treated with an electrosurgical beam conducted by argon gas. Histologic examination of the coagulated liver surface was performed to assess depth of tissue destruction. The effectiveness of tissue coagulation was tested by perfusion with autologous blood through the hepatic artery, portal vein, and inferior vena cava at pressures up to 200 mm Hg with outflow occlusion, and the bile duct with normal saline at 40 cm $H_2O$ pressure. Hemostasis at the coagulated surface was unaffected by high pressure perfusion even with outflow occlusion. The eschar depth of 1-3 mm seen at histopathology confirmed minimal tissue destruction.

The argon beam coagulator was then used to prepare reduced-volume hepatic grafts for two pediatric transplant patients. Each donor weighed three times more than the recipient, and the left lobe of the donor liver was used in each instance. There was no postoperative bleeding, infection, or bile leakage.

We conclude that based on laboratory and early clinical experience, the argon beam coagulator is a significant technical advance in the preparation of reduced-volume hepatic grafts.

FP197 APPLICATION OF REDUCED SIZE LIVER TRANSPLANTS AS SPLIT GRAFTS, AUXILLARY ORTHOTOPIC GRAFTS AND LIVING RELATED SEGMENTAL TRANSPLANTS

C.E. Broelsch, J.C. Emond, P.F. Whitington, J.R. Thistlethwaite, A.L. Baker, J.L. Lichtor
University of Chicago, Chicago, Illinois, USA

Ortthotopic liver transplantation using reduced-size grafts has been reported by us to be as successful as full size grafting. More recently we have used reduced-size grafts as split liver transplants, where 2 recipients share one organ (n=28), as an auxiliary orthotopic transplant (n=1), and as a living related segmental transplant (n=1).

Our series of split livers was performed in 28 recipients, 23 being infants weighing between 2 and and 12 kg. and 5 being adults. Five patients received the left lobe, 9 patients received the left lateral segment and 14 patients received the right lobe. Patient survival was 67% and graft survival 56%. Patient survival from full sized transplants during the same period (6/88-10/89) was 84% (p=.298) and graft survival was 76% (p=.126). Primary non-functioning grafts (4% vs. 5.5%), arterial thrombosis (11% vs. 8%) occurred at a similar rate, while biliary complications were higher (21% vs. 4%). Auxiliary orthotopic grafting was performed in a 23 month old infant with ornithine transcarbamylase deficiency. The recipient's left lobe was removed and replaced by a cadaveric graft, comprising segments 2 and 3. Biliary drainage was provided by a separate Roux-en-Y loop. Metabolic correction occurred with normalization of blood ammonia by one week, and serum aminoacids and urinary orotic acid by two weeks while receiving full dietary protein.

Following thorough consideration of ethical and legal implications, a protocol for living related segmental transplantation was instituted and the first transplant performed on a 21 month old, 12 kg. infant with biliary atresia. From the mother, 27 years and 69 kg., a 460 gr left lobe was removed and reduced by segment 4 to replace the recipients liver. During donor hepatectomy at the time of portal vein clamping, a rupture of the spleen occurred requiring its removal. Following an uneventful transplant procedure, a subcapsular hematoma of the transplant required reoperation. Our clinical investigation will assess 20 of such procedures performed in infants in elective conditions.

**FP198** CLINICAL LIVER TRANSPLANTATION: RECIPIENT HEPATECTOMY FACILITATED BY EARLY APPLICATION OF VENO-VENOUS BY-PASS

G.Gozzetti,G.L.Grazi,A.Mazziotti,E.Jovine, A.Frena, U.Grandi,R.Bellusci,A.Cavallari, R.Paladini*,M.Zanello*.
2° Dept. of Surgery and Dept. of Anaesthesiology*, Univ. of Bologna,Italy.

Since the introduction of liver transplantation (OLT), very few technical modifications changed Starzl's original procedure.Over the past 3 years we have performed 59 OLTs in 51 adult patients. In November 1988 we started applyng the veno-venous by-pass in the early phases of the recipient hepatectomy,after only a minimal dissection of the hepatic pedicle and before the section of the retrohepatic ligaments. The final steps of the mobilization are thus carried out with the patient on by-pass. A satisfactory hemostasis of the retroperitoneal tissues can be achieved with the complete exposure of the retrohepatic space. To verify the effectiveness of our technique we have reviewed the records of 51 primary OLTs comparing operative time,by-pass time, PRBC and FFP replacements of two groups of patients: in group A (20 pts.) the by-pass was started just before the hepatectomy; in group B (30 pts.) the by-pass was started earlier. One operative death was excluded. No difference regarding the operative time was noticed (640.75±105.81 min. vs. 633.8±119.54: p=0.83 NS). As expected the by-pass time was significantly different between the two groups (114.4±40.39 min. vs.150.67±50.21:p 0.01). No differences were seen regarding PRBC (ml.5907.50±5765.76 vs. 6531.17±3852.35: p=0.64 NS) and FFP (ml. 5803.50±3251.50 vs. 7411.33±3608.77: p=0.70 NS) replacement. IN CONCLUSION, although there were no differences in the operative parameters between the two groups,the early application of the veno-venous by-pass made surgery easier, consenting a safer dissection and a more accurate hemostasis of the retroperitoneal tissues with an ideal surgical exposure. The technique did not require a prolonged operative time and did not increase the amount of fluid request or the operative risk.

FP199

### EFFECT OF A PORTASYSTEMIC SHUNT ON SUBSEQUENT LIVER TRANSPLANTATION

M. Aboujaoude, C. Ghent, D. Grant,
R. Mimeault, W. Wall, Depts of Surgery
and Medicine, University Hospital, London,
Ontario, Canada

Although less popular today, many patients with variceal bleeding were treated in the past by portasystemic shunting (PSS). Consequently, significant numbers of patients with PSS are now presenting with end-stage cirrhosis for which the only treatment is transplantation. To determine if PSS influences the outcome of subsequent orthotopic liver transplantation (OLT) we reviewed 193 consecutive transplants performed in 174 patients. Twenty-seven (28 OLT) patients had previous PSS from 4 days to 21 years prior to OLT. There were 12 portocaval (PC), 8 mesocaval (MC), 4 central splenorenal (CSR) and 4 distal splenorenal (DSR) shunts. Five shunts were occluded before OLT, (1 PC, 2 MC and 2 DSR) and 1 MC was stenosed. The age, indications for OLT, status of the patient before OLT and the ABO compatibility between donor and recipient were similar in both groups. The number of units of packed red blood cells (PRBC) and fresh frozen plasma (FFP) transfused intraoperatively, the duration of anaesthesia, the post-OLT biliary complications and the 5 year actuarial patient survival are shown in the table:

| | Shunt | Non shunt | p value |
|---|---|---|---|
| mean PRBC (units) | 9.1 | 9.2 | N.S. |
| mean FFP (units) | 11.0 | 12.3 | N.S. |
| duration of anaesthesia hrs (mean) | 8.1 | 7.8 | N.S. |
| biliary complications | 22 % | 5.4% | <0.013 |
| patient survival | 76 % | 70 % | N.S. |

We conclude that a prior PSS does not adversely affect survival after OLT but there is an increased incidence of biliary complications.

### FP200 SURGICAL COMPLICATIONS AFTER 110 LIVER TRANSPLANTATIONS (LTx) IN A MULTICENTER PROGRAM INVOLVING 10 DIFFERENT STAFF SURGEONS

V.S. Turrión, N.P. Mora, J. Herrera, F. Pereira, E. Vicente, J. Vázquez, J. Murcia, M. Gámez, M.L. Santamaría and J. Ardaiz

Intrahospital Liver Transplant Unit, Clínica Puerta de Hierro - Hospital La Paz - Centro Especial Ramón y Cajal, Madrid, Spain

From Jan. '86 to Dec. '89, 110 LTx were performed in 92 patients (62 adults and 30 pediatric). Age range was 17 mo to 62 yr. Indications for LTx were biliary atresia (n=18), primary biliary cirrhosis (n=7), sclerosing cholangitis (n=1), chronic hepatocellular failure (n=47), metabolic disorders (n=9), neoplasia of the liver (n=4) and others (n=6). Thirteen patients required veno-venous bypass during anhepatic phase. Arterial reconstruction was carried out with hepatic-to-hepatic anastomosis in 93 cases. Aorto-aortic anastomosis was performed in 32 LTx (requiring donor aortic graft). Biliary reconstruction consisted of 78 duct-to-duct anastomoses and 32 choledocojejunostomies. Donor and recipient operations were performed by 10 different staff surgeons from 3 centers, each of whom had directed the procedure in several cases. **Results:** Operative time range was 4.5-16 h (mean=10.25 h). Intraoperative blood use was 11.15 red blood cell units and 20 fresh frozen plasma units. Of the 92 patients, 1 (1.08%) died intraoperatively; surgical complications requiring abdominal reoperation arose in 60 cases (65.2%), and postoperative bleeding was present in 24 (26%). Incidence of vascular complications was: arterial thrombosis, n=13 (11.8%); hepatic artery aneurysm due to invasive candidiasis, n=3; and portal stenosis, n=1. Total biliary complications were 28 (25.4%), only 10 (9.09%) requiring reoperation. Intestinal obstruction occurred in 6 patients and, finally, 2 developed postoperative abdominal wound hernia. Length of hospital stay was 30±10 days and one year actuarial survival is 68%, with 2 - 3-yr survival in 64.25%. **Conclusions:** Patient actuarial survival and surgical morbidity appear to be similar to those reported by other authors. We conclude that a multicenter LTx program involving several staff surgeons with other surgical duties in addition to those of LTx may be a suitable way to perform LTx, and to optimize the use of medical resources in our public health system.

### FP201 LIVER TRANSPLANTATION FOR PARACETAMOL -INDUCED FULMINANT HEPATIC FAILURE.

KC Tan, D Potter, R Williams, RY Calne.

Kings College Hospital, London, UK
Addenbrookes Hospital, Cambridge, UK

The results of medical treatment for advanced fulminant hepatic failure (FHF) have been universally poor. With the improvement in survival rates for liver transplantation, it is now accepted as an important therapeutic modality for patients with advanced FHF. Although paracetamol overdose is a major cause of patients seen with FHF, seldom are these patients transplanted. Of the 42 patients transplanted for FHF in Pittsburgh only 2 were suspected of acetaminophen poisoning (S Iwatsuki et al 1989). This reluctance is perhaps due to the fact that almost all of these cases are attempts in committing suicide.

We report our experience of transplanting 5 patients with paracetamol induced advanced FHF. None of them had a history of previous suicidal attempts and ingested the drug at a time of acute depression. All of them met our risks criteria for liver replacement. Three of these patients survived at 4, 11 and 16 months posttransplant and all have returned to a normal lifestyle. The other two patients died from infectious complications. Over the same period 52 other patients were admitted into our unit with paracetamol induced FHF. There was an overall survival rate of 60%. However, of the 10 patients that met our risks criteria but were not transplanted because of sepsis or the lack of a suitable donor liver only one survived.

We conclude that liver transplantation should be considered an important therapeutic modality for patients with paracetamol induced FHF. However it is important to screen these patients for a history of previous suicidal attempts.

Reference : Iwatsuki S et al. Trans Proc 1989: 21 : 2431

### FP202 LIVER TRANSPLANTATION FOR PRIMARY HEPATIC MALIGNANCY

**T Ismail, L Angrisani, J Buckels, P McMaster**
**Queen Elizabeth Hospital, Birmingham, UK.**

The results of orthotopic liver transplantation as a curative therapy for primary hepatic malignancy have been disappointing. We analysed retrospectively our data to determine the influence of pre-operative evaluation, histological type and staging on outcome.

Between 1982 - 1989, 134 patients suspected of having a liver neoplasm were referred. In 105 (78%), a primary hepatic neoplasm was confirmed histologically. Twenty-nine liver transplants were performed in 28 of these patients (26.6%). 20 patients (71%) survived at least 30 days (mean 18.5 months; range 1 - 84) and 9 are currently alive. CT Scan proved to be superior to intra-operative assessment (86 vs 58%) in diagnosing tumour positive nodes. Patients with tumour negative nodes had a better prognosis. Postoperative stage I/II (Fortner) had a 2 year actuarial survival of 70% compared with no survivors beyond 18 months for stage III disease. Non-cirrhotic hepatocellular carcinoma had the best prognosis; central cholangiocellular carcinoma had the worst outcome.

Because of the advanced stage of disease at the time of presentation, the value of liver transplantation in primary hepatic malignancy is limited. However, for those presenting with advanced disease (stage I/II) significant benefit can be achieved.

FP203 THE IMMUNOSUPPRESSIVE CAPACITY AND MODE OF ACTION OF THE NEW IMMUNOSUPPRESSANT 15-DEOXYSPERGUALIN (DOS) IN ORTHOTOPIC RAT LIVER TRANSPLANTATION

Gassel, H.-J., Ellwanger, S., Engemann, R., Hamelmann, H.
Dept. of General Surgery, University of Kiel, Arnold-Heller-Str. 7, D-2300 Kiel, West-Germany

The introduction of Ciclosporin A (CsA) greatly improved the results of clinical liver transplantation. However, significant side effects necessitate the search for alternative drugs. In this study the new immunosuppressant DOS was compared to CsA in orthotopic rat liver transplantation (ORLT) both for prophylactic ( initial) and recsue treatment in a rejector strain combination. ORLT was performed in the DA (RT1a) to LEW (RT1l) combination using an arterialized transplantation technique (n=42). For initial treatment either DOS or CsA were administered from d0 to d13 after ORLT, for therapy of an ongoing rejection from d5-d19 and d7-d21 respectively. At differential time intervals differential white blood counts were performed and bone marrow smears were analysed. Initial therapy (d0-d13) with DOS as well as with CsA led to long-term survival of more than 80% of the LEW recipients (untreated control rats:MST 10.3d). An ongoing rejection was reversed by both drugs when started therapy at d5 after ORLT. However, onset on d7 after ORLT led to only 16 % long-term survival in the CsA treated group.In contrast, DOS therapy could revert the strong rejection in 84% of the animals. During DOS therapy a significant reduction in the number of granulo-, mono-, and lymphocytes was seen in the peripheral blood with a simultaneous reduction of the granulopoesis in the bone marrow. 1) After allogeneic ORLT both CsA and DOS induce long-term survival when treatment starts initially or very early. 2) In contrast to CsA DOS can be used to revert an ongoing rejection even in later stages. 3) A bone marrow depressive effect seems to be one mode of action of DOS. 4) Due to the different mechanisms of action a combination of both drugs with reduction of the single dosages seems to be helpful.

### FP204 SELECTIVE BOWEL DECONTAMINATION (SBD) PREVENTS INFECTION AFTER LIVER TRANSPLANTATION (LTx)

C. Rosman, IJ. Klompmaker, GJ. Bonsel'
RP. Bleichrodt, JP. Arends, MJH. Slooff
Liver Transplant Group Groningen and
Institute for Medical Technology
Assessment Rotterdam, the Netherlands

Gram negative bacterial and fungal infections, are a major cause of morbidity and mortality after LTx. The aim of this study is to investigate the efficacy of SBD as infection prevention in a series of 43 adult LTxs (1985-1989). Methods: SBD was established by giving oral doses (qid) of polymyxin B 100mg, tobramycin 80mg and amphotericin B 500mg starting 6-8 hours before LTx. This regimen was continued postoperatively for at least three weeks. Peri-operatively parenteral tobramycin and cefotaxim were administered during 48 hours. To assess the efficacy of the SBD regimen, quantitative cultures from pharynx, faeces, bile, urine, sputum, wounds and all abdominal discharges were taken twice weekly. A diagnosis of infection was based on positive cultures in combination with symptoms and signs of infection. SBD was considered successful if from day five after LTx faecal concentrations of gram negative bacteria and yeasts were less than $10^3$/gr faeces. Results: Bacterial and fungal infections were present after 19 LTxs (44%). SBD was successful (SBD-S) after 27 LTxs (63%). SBD failed (SBD-F) after the remaining 16 LTXs (37%). These two groups did not differ in patient diagnosis, peroperative blood loss, operating time, type of biliary reconstruction and number of rejections. In the SBD-S group significantly less LTxs (0/27) were complicated by gram negative or yeast infections as compared to the SBD-F group (6/16, P<0.01). Infections with gram positive micro organisms were not different in both groups. Overall survival and hospital stay were not affected by success or failure of SBD. Conclusion: SBD was effective in only 63% of the LTxs. However, if successful, a significant reduction in the incidence of gram negative and yeast infections could be observed.

FP205

### SIGNIFICANCE OF MUCUS SECRETION IN ROUTINE COMMON BILE DUCT EXPLORATION

Y. Y. Jan, M. F. Chen
Chang Gung Medical College, Taipei, Taiwan, R.O.C.

Fifteen patients with profused mucus secretion in common bile duct during surgery for hepatolithiasis were encountered from 1977 to 1988. There were 6 men and 9 women. Age ranged from 46 to 88 years old. Clinical presentation with repeated cholangitis was (13/15) 86% and two patients with septic shock. Pre-operative diagnosis with mucobilia was made in 3 patients during intrumentation (ERCP or PTBD).

They all underwent surgery because of cholangitis. Besides, common bile duct exploation, other operative treatments included 8 hapatic resection and 4 intraheptic tubing. Operative mortality is (1/15) 6.6% due to septic shock, curative hepatic resection achieved in 5. Three died 15, 16, 17 months after surgery for tumor metastasis and cholangitis. Other 7 needed repetition choledochofiberscopy for removal of the persistent mucus secretion. Intraluminal radiation therapy was done in 4, remission could be obtained, survived from 2 to 4 years after the procedures. Histopathological examination revealed intrahepatic bile duct papillary adenocarcinoma in 13 and two mucinous producing cystic tumor of the liver.

Copious mucus secretion within the extrahepatic bile duct was a rare cause of jaundice and cholangitis. It may be found in routine common bile duct exploration. The importance of the presence of mucus secretion within the commo bile duct is the possible association with itnrahepatic bile duct carcinoma.

**FP206** PREOPERATIVE AND POSTOPERATIVE CHANGES IN PATIENTS WITH OBSTRUCTIVE JAUNDICE

Y.Arıtaş, I.Can, Z.Yılmaz, N.Bengisu

Erciyes University,Medical School,
Department of Surgery,Kayseri-Turkey

Surgery for biliary tract obstruction is still associated with significant morbidity and mortality. In this study, fifteen jaundiced patients with malignant bile duct obstruction and fifteen patients with benign obstructive jaundice were assessed for their immune status in preoperative and postoperative $7^{th}$ days. Fifteen other patients with cholelithiasis were taken as a control group. Cell-mediated immunity tested by T-cell count and delayed type cutaneous hypersensitivity reactions to PPD, showed strong depressions in jaundiced patients in preoperative periods and after drainage ($p < 0.01$). The humoral component of the immune system was judged by B-cell count and quantitative determinations of serum immunoglobulins G, M and A, by single radial immunodiffusion technique. In cancer patients,common consistent changes were reduction of serum immunoglobulin G levels and elevation of B-cell count in preoperative and postoperative periods. Wound infection developed in 33 percent of these malignant patients.

FP207 THE INFLUENCE OF BILIARY OBSTRUCTION ON RETICULOENDOTHELIAL FUNCTION AND MORTALITY IN GRAM-NEGATIVE SEPSIS IN THE RAT

R. Andersson. Department of Surgery, Lund University, Sweden.

Chronic biliary obstruction has been suggested to influence the postoperative course and incidence of postoperative complications. The present experimental study aimed at evaluating the influence of E.coli peritonitis on the reticuloendothelial function and mortality in animals subjected to common bile duct ligation (CBDL).

Male Sprague-Dawley rats weighing about 300 g were allocated to either sham celiotomy with mobilization of duodenum and isolation of the common bile duct without ligation, or to extrahepatic obstruction of the common bile duct with isolation and double ligation with 6-0 silk and excision of 5-8 mm of the middle part of the common bile duct, in order to prevent re-canalization. Two weeks after operation the animals were randomized to receive an intraperitoneal (i.p) injection of either $10^9$ colony forming units of E.coli (1.0 ml) or 1.0 ml sterile saline. In the first set of experiments (n = 15 per group), the influence on mortality was evaluated. In the second set of experiments (n = 6 per group) clearance and organ distribution of intravenously injected E.coli were determined. 5 h after the i.p. injection of sterile saline or live E.coli, radiolabelled, heat-killed $^{125}$I-E.coli suspension was injected into the femoral vein, followed by arterial sampling at 2, 8 and 15 min after which samples from the liver, spleen and lungs were taken.

Mortality significantly increased from 2/15 (sham + E.coli) to 8/15 among animals with both E.coli peritonitis and CBDL ($p < 0.05$). Clearance of radiolabelled E.coli from the blood was significantly delayed in CBDL animals, both with or without a concomitant peritonitis. The liver vastly dominated the organ uptake, without difference between the various groups. Pulmonary uptake significantly increased in septic animals, most exaggerated in the group with CBDL together with E.coli peritonitis.

Thus, CBDL increases mortality in a concomitant E.coli peritonitis and decreases blood clearance of radiolabelled bacteria. No influence on liver uptake could be demonstrated.

**FP208** 

### T Lymphocyte Function in Obstructive Jaundice and Following Biliary Drainage: Comparison of Animal Model and Patients.

**B J Rowlands, M Hoper, S Ranjbar, D McCluskey, R Thompson. Dept of Surgery, The Queen's University of Belfast, UK.**

Patients with obstructive jaundice have a high incidence of post-operative infective complications. This study evaluates cell-mediated immunity in an animal model (rat) of biliary obstruction (BDL) for 21 days and following internal biliary drainage (ID) for 7, 14 and 28 days. Patients with obstructive jaundice were also assessed preoperatively and 6 weeks following relief of their jaundice and were compared to age and sex matched controls. Cell-mediated immunity was assessed by a mitogen stimulation test of T lymphocytes.

**MAXIMUM PHA STIMULATION**

| **Animals (n=10 per group)** | | **Patients** | |
|---|---|---|---|
| Controls | 27193±10027 | Controls n=24 | 29950±5989 |
| 21 BDL | 10927± 5880 | Patients Pre-op n=18 | 19049±6876 |
| 7 ID | 12974± 3101 | Patients Post-op n=18 | 26393±4465 |
| 14 ID | 21748± 2809 | | |
| 28 ID | 34110±10346 | | |

There is significant depression of lymphocyte function in both animals and patients with obstructive jaundice. Following relief of the jaundice there is a gradual improvement in lymphocyte function, but 4 weeks are required to return to control levels. Patients returned to normal after 6 weeks' drainage. The return of lymphocyte function is delayed compared to other biochemical liver function tests. Short term external biliary drainage is unlikely to affect depressed lymphocyte function or influence susceptibility to infective complications.

**FP209** *BLOOD T3 and T4 LEVELS IN PATIENTS WITH BENIGN OBSTRUCTIVE JAUNDICE*

*Mehmet TUĞCU, Hasan KAPLAN, Enis YETKİN, Alp GÜRKAN, Mustafa BAŞÖZ*

*General Surgery Dept Medical School Ege U. Izmir/TURKEY*

*It is known that serum T3 and T4 levels decrease generally in patients with liver diseases(1,2,3).*

*In this preliminary report, we present the results of a clinical research upon surgically correctable benign obstructive jaundice and probable hypothyroidism in the base of the low T3 and T4 levels in peripheral blood.*

*The patients were choosen randomly among the cases with obstructive jaundice who have no known endocrine pathology and detectable thyroidal disease by physical exam and thyroid scanning.*

*23 cases(14 choledocholithiasis, 6 benign biliary tract stricture 2 hydatid cyst ruptured into common bile duct, 1 Oddi stenosis) were analysed pre- and postoperatively(21st day).*

*Results,*

| | In 10 cases(*) | In 13 cases |
|---|---|---|
| T3 | 40-75 ng/dl | 95-175 ng/dl |
| T4 | 1.8-4.0ng/dl | 4.8-12.0 ng/dl |
| T.Bil | 3.80-24.80 mg/dl | 2,0-17.60 mg/dl |
| Mean | 10.84 mg | 6.36 mg |

*T3 and T4 levels turned to normal levels in 9 of these cases. It remained low in only one case.*

*This findings suggest that the obstruction of biliary drainage is one of the important factor upon decreased peripheral thyroidal hormon levels. The correction of biliary obstruction provides to turn of blood levels into normal values.*

*(*) Normal values T3(80-200 ng/dl)*

*T4(4.5-11.5 g/dl)*

1.Hegedus L Metabolism 35:6,495-498,1986
2.Ella GH,Sepersky RA:Dig Dis Sci.28:11,971-975,1983
3.Hepner GW,Chopra IJ: Arch Intern Med 139:1117-1120,1979

### FP210 IMPROVED SURVIVAL IN SURGICAL JAUNDICE WITH TINOSPORA CORDIFOLIA, AN IMMUNOMODULATOR

R. Bapat, N. Rege, R. Koti, N. Desai, S. Dahanukar
Dept. of G.E. Surgery and Dept. of Pharmacology,
Seth G.S.M. College & K.E.M. Hospital, Bombay, India.

Patients with surgical jaundice are susceptible to infection, during preoperative drainage. Experimental studies have proved immunosuppression as the predisposing factor for infection in cholestasis. A clinical study planned to confirm these findings revealed a significant depression of phagocytic and microbicidal activities of neutrophils isolated from the patients with surgical jaundice compared to normal values. This justifies the use of an immunomodulator in these patients. Tinospora cordifolia (TC) an Ayurvedic drug was found to consolidate host defenses in animal model of cholestasis. A single blind randomized trial was carried out to evaluate the effect of TC on prognosis of surgical jaundice. Gp I (n=15) was treated with PTCD and antibiotics while Gp II (n=15) received TC in addition to PTCD and antibiotics for three weeks. Tests to determine liver functions (including antipyrine half life) and host defenses were carried out before therapy, during preoperative drainage and before surgery. All patients, had palliative biliary-enteric anastomosis.

Based on the antipyrine data, 39.6% survival was predicted for Gp I and 46.6% for Gp II. Phagocytic and microbicidal activities of neutrophils improved significantly in Gp II. Postoperatively Gp I had 39.6% survival as predicted; the percentage survival improved to 92.4% in Gp II.

Thus introduction of TC in therapy improved percentage survival in surgical jaundice by bolstering host defenses.

Reference

Thatte U.M., Dahanukar S.A. Phytotherapy Research 1989 : 3 : 43

FP211

### ESSENTIAL FATTY ACID METABOLISM IS ABNORMAL IN OBSTRUCTIVE JAUNDICE

MW Scriven, MA Thomas, MS Manku#,
JCM Stewart#, DF Horrobin#, MCA Puntis.
University Of Wales College Of Medicine,
Department Of Surgery, Cardiff, UK.
#Efamol Research Institute, PO Box 818,
Kentville, Nova Scotia, Canada.

The essential fatty acids (EFA) are polyunsaturated fats (PUFA) of 2 types, the n-6 derived from linoleic acid and the n-3 from alpha-linolenic acid. The elongation and desaturation of EFA are probably controlled by the same enzyme systems in both types. Several factors, including atopy and diabetes, are known to influence the activity of the first and rate limiting enzyme, delta-6-desaturase. The aim of this study was to establish whether obstructive jaundice influences EFA metabolism.

Sixteen fatty acids in 3 lipid fractions were measured in 6 patients with obstructive jaundice. The mean age was 66 (58-75) and the mean bilirubin 286μmol/l (103-492). None had coexisting disease known to affect EFA metabolism. The 3 fractions were plasma phospholipids (PL) and cholesterol ester (CE), and red cell (RBC) membrane phospholipid.

There was a significant* decrease in linoleic acid metabolites, especially arachidonic acid, precursor of the prostanoids. This was found in all 3 fractions, as was an elevation in the ratio of linoleic acid to arachidonic acid.

There was a significant depression of PUFA in all 3 fractions and a significant rise in both saturated and monoenoic acids, in plasma PL and RBC PL. RBC membrane 22 carbon PUFA were also significantly depressed.

The pattern of fatty acids in plasma and RBC membrane is abnormal in obstructive jaundice, indicating depressed delta-6-desaturase activity. Changes in EFA metabolism may contribute to the excess mortality and morbidity in these patients, which may improve with EFA supplements.

* All statistical analysis by Wilcoxon test using $p<0.05$

FP212 THE METHOD OF MEASUREMENT FOR BILIRUBIN FALL DEGREE AFTER BILIARY DECOMPRESSION AND ITS ESTIMATION

T. Shimizu*, Y. Tsuchiya, S. Hasegawa,
O. Sato, K. Tsukada, K. Yoshida, T. Muto
Shinrakuen Hospital* & 1st Dept of
Surgery, Niigata University School of
Medicine, Niigata, Japan

We found the rule 15 years ago that serum bilirubin levels decreased lineally on the semilogarithmic graph-paper after biliary decompression when the patient does not have any signs of cholangitis or drainage tube trouble. The rule could be formulated as $Y=ae^{bx}$, where 'b'; the decreasing rate of serum bilirubin. We investigated the pathophysiology of 579 patients with severe obstructive jaundice using this bilirubin decreasing rate 'b'.

The 'b' value reflected postoperative complications and operative death rate clearly. The pancreato-duodenectomy (PD) was performed in 62 patients. Among 33 patients who showed excellent 'b' value, only one had postoperative complications. Three patients, out of 17 who showed good 'b' value were complicated. Eleven patients, out of 15 who showed worse 'b' value had severe complications. No patient with poor 'b' value was considered to tolerate PD operation. Under the 26 histological examinations, the degree of ductal cell degeneration was closely correlated to the 'b' value. However, the other degree of histological findings was not correlated to that. This ductal cell degeneration continued for several months and was considered to be a histological manifestation of cholangitis. In clinical studies based on the 'b' value, the cholangitis was classified into hepatic injury type and renal injury type.

We considered that the degree of ductal cell degeneration determined the 'b' value and this degeneration was caused by the cholangitis of hepatic injury type.

### FP213 CHANGES IN BILIRUBIN AND BILE SALTS AFTER THE RELIEF OF OBSTRUCTIVE JAUNDICE

MW Scriven, MA Thomas, MCA Puntis.
Department Of Surgery, University Of Wales
College Of Medicine, Cardiff, UK.

Serum bilirubin levels fall gradually after the relief of obstructive jaundice. This decline has been examined by Little (1986) who found that the clearance of bilirubin followed an exponential pattern. Parameters derived from this analysis correlated with measures of outcome and were therefore of prognostic value.

We are examining plasma bile salt clearance to see if this can be characterized in the same or a similar way. If it can, the resulting parameters may provide an earlier and perhaps more sensitive indicator of outcome.

We have measured total plasma bile salt levels in a group of jaundice patients. The mean bilirubin at the time of the relieving procedure ranged from 157-311 µmol/l (mean 230). The fall in bilirubin was consistent with Little's analysis, giving the mean estimated time of reaching the normal range as 31 days (range 21-42). The bile salt levels ranged from 42-151 µmol/l (mean 114) and followed a different clearance pattern. From the rapid initial decline the estimated return to the normal range (under 6 µmol/l) varied from 2-15 days (mean 11 days).

Reference

Little JM, Robotin M.
Serum Bilirubin After Surgery For Obstructive Jaundice.
Aust.N.Z.J.Surg. 1986 ; 56 : 35-37

### FP214 PREVENTION OF POSTOPERATIVE COMPLICATIONS IN JAUNDICED RATS. INTERNAL BILIARY DRAINAGE VERSUS ORAL LACTULOSE

JW Greve, JG Maessen, WA Buurman, DJ Gouma. Department of surgery, Academic Hospital, Maastricht, The Netherlands

Surgery in patients with obstructive jaundice is associated with many postoperative complications. Preventive treatments with external biliary drainage were unsuccessful. Preoperative internal biliary drainage and oral lactulose, an anti-endotoxin, were shown to prevent complications, however, it is still unknown which treatment is prefered. In this study, we compared the effect of both treatments on the outcome of a severe surgical trauma in rats with experimental biliary obstruction.

Methods: Four experimental groups of each 10 rats were studied: bile duct ligation (BDL), sham operation (SHAM), BDL and oral lactulose (3 days prior to surgical trauma, 3x2 ml 30% lactulose syrup per day), BDL and internal biliary drainage (3 weeks prior to the surgical trauma). Two weeks after the initial operation, except in the drainage group, a surgical trauma was simulated by bilateral renal ischemia. Thereafter renal function (BUN) and survival time were studied during 1 week.

Results: All BDL rats died within 6 days after renal ischemia, resulting in a significantly decreased survival time compared to SHAM. Deterioration of renal function was also significantly increased in BDL rats. Compared to BDL, oral lactulose significantly increased survival time, but did not significantly improve renal function. Internal biliary drainage significantly decreased renal impairment and increased survival time.

TABLE: values are mean ± SD

| | Survival (hr) | Change of BUN (mmol/l) | | Statistics (Student T) |
|---|---|---|---|---|
| | | day 0-1 | day 1-2 | vs BDL |
| BDL | 54±35 | 54.7± 4.2 | 43.9± 1.0 | |
| SHAM | 134±49** | 41.6± 4.7** | 28.3±20.9# | # p<0.05 |
| BDL-LACT | 125±53* | 47.8±12.6 | 24.1±21.4# | * p<0.01 |
| BDL-DRAIN | 156±35** | 34.0±15.6** | 1.5±19.2** | ** p<0.001 |

Conclusion: The results suggest that preoperative internal biliary drainage is the preventive treatment of choice in jaundiced patients. However, in patients where adequate internal biliary drainage can not be obtained, oral treatment with lactulose may attribute to reduce postoperative complications.

### FP215 BILIARY ACIDS AND RENAL FUNCTION IN JAUNDICED PATIENTS

V. RIBEIRO, T. C. NORTHFIELD, M. KNIGHT Hospital Santo António-PORTO
St. George's Hospital LONDON- UK

Renal failure and sepsis still representtoday the major complication in the treatment of surgical jaundice. Recently bile salts and bile acids were said to benefict these patients.

Under antibiotic covereage and per-operative Manitol we randomized 31 patients. Group I (16 patients) no further treatment, grou II (15 patients) received chenodeoxicolic acid (15 mg/Kg/day) for 2 days. Patients were evaluated for renal function pre and post-operativelly for 2 days, and sistemic endotoxemia using a quantitative assay (chromogen S-2423) pre, per and post-op, as well as portal vein.

Both groups were identical for age, bilirrubin levels, weight loss, malignancy, albumin and creatinin cl. Complications, systemic and infetious were identical, as well as systemic endotoxemia. Portal endotoxemia was significantly lower in the treatment group (p 0,05). Mortality was similar in both groups.

In conclusion chenodeoxicolic acid significantly lowered portal endotoxemia in these patients, but fails to improve significantly the renal function and the complications in jaundice.

### FP216 POSTOPERATIVE RENAL FUNCTION IN OBSTRUCTIVE JAUNDICE - A PROSPECTIVE RANDOMISED STUDY

J.Pain, C.Cahill, J.Gilbert,
J.Trapnell, M.Bailey
King's College Hospital, London, UK

The role of preoperative lactulose and bile salts in the protection of postoperative renal function in patients with obstructive jaundice has been evaluated in a prospective randomised trial.

One hundred and three patients undergoing surgery for obstructive jaundice (bilirubin >100μmol/l) were randomised into three groups: preoperative oral lactulose (n=35), oral sodium deoxycholate (n=32) and controls (n=35). All patients received intravenous fluids commencing the night prior to surgery. One patient in the control group and none in the treatment groups developed postoperative renal failure. Postoperative deterioration of renal function in patients with normal preoperative function was significantly more common in the control group than in the treatment groups ($p<0.02$). The incidence of renal failure and impairment was lower in the control group than in previous studies, and we believe this is due to the introduction of adequate preoperative hydration. Additional protection can be provided by the preoperative administration of either lactulose or sodium deoxycholate.

| | Control Group | Lactulose Group | Bile Salt Group | |
|---|---|---|---|---|
| Preoperative alone | 7 | 10 | 6 | |
| Pre- and postoperative | 4 | 4 | 3 | |
| Postoperative alone | 8* | 1 | 2 | * p<0.02 |

Table: Incidence of pre- and postoperative renal impairment

FP217 COMPARATIVE EVALUATION OF EARLY CHOLECYSTECTOMY VERSUS INTERVAL CHOLECYSTECTOMY IN ACUTE CHOLECYSTITIS

T.K. Malik, S. Madan, R. Prakash,
R. Malik. Dept of Surgery, Maulana Azad
Medical College, New Delhi, India

In this prospective study 100 patients were taken up for study in each group. Those patients to be taken up for early cholecystectomy were subjected to ultrasonographic examination & DISIDA scanning for confirmation of clinical diagnosis and put on the next operation list for cholecystectomy under antibiotic cover. Peroperative saline biliary manometry was done in all cases to debit the incidence of retained CBD stones and CBD explored when necessary. It was seen that there was no significant increase in technical difficulty and post operative morbidity and mortality were comparable with the conservative group. The overall hospital stay was reduced by approximately 10 days thereby reducing hospital costs. On the other hand in the conservative group 2 patients had to be operated on because of failed conservative treatment and management and 1/3rd had to be readmitted while awaiting elective surgery due to failed conservtive management. We agreed with McArthur et al (1975) Norrby et al (1983) in that there is a clear benefit in doing early cholecystectomy for cholecystitis than interval cholecystectomy.

In conclusion early cholecystectomy has everything to gain and nothing to lose as compared to interval cholecystectomy.

We will also discuss the statistical incidence of cholecystitis in the City of Delhi in terms of age, sex, economic status, obesity, fertility and possible aetiopathology and type of stone.

References:

Lethinen J., Alhava EM, Aukes: Scand Jr. Gastroent.13:673-678, 1978.
Norrby S., Herlin P., Holmin T., Sjohahl R., Tagerson C: Br. Jr. of Sur G.70:163-165, 1983.

FP218

### MIRIZZI'S SYNDROME: SCINTIGRAPHIC DIAGNOSIS

A Alarco, J Perez Palma, A Herrero
J J Afonso, H Diaz, E Glez Reinmers
A Conde, F Glez Hermoso
Hospital Universitario de Canarias
La Laguna, Tenerife, Spain

Obstruction of the cystic duct by a stone may lead, in some instances, to partial common bile duct obstruction. This entity has been termed Mirizzi's syndrome. Bilio-biliary fistulization of the cystic duct into the common bile duct may occur, either spontaneously or due to surgical procedures. It is therefore important to assess the diagnosis preoperatively, a difficult matter since clinical and biological parameters are similar in patients with acute cholecystitis with and without Mirizzi's syndrome. In the last years, however, ultrasound, computerized tomography, scintigraphy and ERCP have shown their usefulness in the diagnosis of this entity. In the present paper we analyse the clinical and biological features of 18 patients affected by Mirizzi's syndrome (out of 205 patients operated on for acute cholecystitis), and also the diagnostic capacity of HIDA-DISIDA scintigraphy in this entity. We have restrospectively analysed 205 cases of acute cholecystitis wich have been operated on in the last ten years, in wich preoperative scintigraphy of the biliary tract was performed, previus i.v. administration of 3-5 mci of 99m-Tc HIDA-DISIDA. In 18 cases, a Mirizzi's syndrome was detected (14 females, 4 males) at the time of operation. Neither clinical features (pain in the right upper quadrant, vomiting, and, fever), nor biochemical parameters (leukocytosis, elevation of phosphatase alkaline, bilirubin an GGT) were different in patients with and without Mirizzi's syndrome. By contrast, scintigraphy offered a highly sensitive and specific pattern, consisting of: 1.- Non-visualization of the gallbladder; 2.- Moderate prestenotic dilatation of the main hepatic bile duct; 3.- Delayed excretion into the duodenum. Thus, in our experience, 1. prevalence of Mirizzi's syndrome among patients affected by acute cholecystitis is 8.78%, and 2, 99m Tc HIDA-DISIDA scintigraphy is a highly sensitive and specific diagnosctic procedure in this entity.

### FP219 THE USE OF DYNAMIC Tc99m HIDA SCANNING IN THE DIAGNOSIS OF ACALCULOUS GALLBLADDER DISEASE

M.Winslet, C.Hall, K.Harding, M.J.R. Lee, J.P.Neoptolemos
Departments of Surgery & Nuclear Medicine, Dudley Road Hospital, Birmingham B18 7QH, United Kingdom.

Patients with chronic upper abdominal pain for which no cause can be found by conventional investigation (upper GI endoscopy, ultrasound, OCG), present a major management problem. A proportion of such patients may have underlying acalculous biliary disease. Standard Tc99m HIDA scanning with a dedicated computer, combined with cholecystokinin provocation, may identify patients with occult biliary dyskinesia.

Twenty-three patients (M=7, F=16, mean age 52 (22-76) yrs) with chronic upper abdominal pain were studied. All patients had a normal endoscopy and ultrasound examination. Oral cholecystography and ERCP, performed in 13 patients, was normal.

Computerised dynamic Tc99m HIDA scanning with synchronous I/V CCK (75 iu) administration was normal in 9. Abnormal gallbladder filling was present in 7 patients with abnormal emptying in a further 7.

| | Latent period (min) | Ejection period (min) | Ejection fraction (%) | Ejection rate (%/min) |
|---|---|---|---|---|
| Normal | 0.2±0.1 | 5.0±0.6 | 55.5±4.1 | 10.0±0.5 |
| Poor filling | 3.8±2.0 | 9.3±1.4 | 52.0±5.5 | 6.1±1.5 |
| Poor emptying | 5.8±2.1 | 26.3±4.3 | 18.2±3.4 | 0.8±0.3 |

Nine patients have undergone cholecystectomy to-date. Four had evidence of chronic cholecystitis with cholesterosis in a further five. All are asymptomatic at a median 7 (6-15) months.

Computerised Tc99m scanning may be used to objectively determine the presence of occult biliary dyskinesia.

FP220 ACUTE ACALCULOUS CHOLECYSTITIS

J.C.U. Coelho, A.C.L. Campos, M. Moreira
A.A. Moss Jr., G.V. Artigas

Federal University of Paraná, Curitiba,

Paraná, Brazil

Thirty-two patients with acute acalculous cholecystitis are presented. The age of the patients ranged from 1 to 80 years, with an average of 46.3 years. Acute acalculous cholecystitis occurred during the postoperative period in only four patients. Three patients were receiving total parenteral nutrition and 16 patients had one or more associated medical diseases. One patient had acute acalculous cholecystitis due to mechanical obstruction of the cystic duct caused by a diafragmatic hernia. The most frequent signs and symptoms were right upper quadrant abdominal pain, nauseas, vomiting, fever, abdominal mass, and jaundice. All patients were subjected to cholecystectomy. Nine (28.1%) gallbladder specimens had gangrene. Pericholecystic perfuration was observed in four patients (12,5%), free perfuration in one patient (3.1%), and empyema of the gallbladder in one patient (3.1%). Bacteria were cultured from 18 of 24 bile specimens. E. coli was the most common organism isolated. The overall postoperative mortality and complication rates were 15.6% and 40.6% respectively. The average hospital stay was 16.4 days.

FP221 "MINI-CHOLECYSTECTOMY" FOR CHOLELITHIASIS. COMPARATIVE STUDY OF THREE DIFFERENT ABDOMINAL INCISIONS IN A COUNCIL HOSPITAL

Badia JM, Martínez-Ródenas F, Pié J, Vila JM, Escalante J, Moreno J.

Dept. of Surgery. Hospital Municipal de Badalona. Badalona. Spain.

Until recently, cholecystectomy has been the only available treatment of gallstone disease. Over the past decade alternatives to cholecystectomy have been developed, but surgical treatment continues to be the only therapy that controls the disease quickly and permanently. "Mini-cholecystectomy" (MC) through a transverse right subcostal incision has evolved as a surgical technique that can offer a more comfortable postoperative period to the patient, with low morbidity and a lenght of stay as short as 24 hours. We present a four year experience(1986-1989) in gallbladder surgery in a Council Hospital, analizing the outcome of three different approaches to cholelithiasis and stressing some technical aspects of MC.
Four hundred and fifty six cholecystectomies were performed during this period. The incisions used were 230 (50.4%) medial laparotomies (ML), 173 (38%) transverse subcostal laparotomies (MC) and 53 (11,6%) right subcostal laparotomies (RSL) (Kocher incision). The three groups were comparable in age, sex, severity of gallbladder disease, incidence of common duct lithiasis and drainage of the subhepatic space. Operative cholangiogram was peformed on 466 (98,2%) of the patients, including those patients treated with MC. The overall surgical morbidity rate slightly dropped to 18,3% in 1989. The incidence of MC increased during the studied years from 23.6% in 1986 to 60% in the last year. The morbidity rate of MC (16.2%) was significantly lower than morbidity of ML (26.1%) and RSL (26.4%), $p<0.05$. Mean age of complicated patients was higher (56 years) than that of non complicated patients (49 years). The mean postoperative stay for non complicated patients was 6.6 days vs 10.2 days for patients who suffered complications ($p< 0.05$). Overall mortality rate was 0.2%.
We conclude that MC is a safe surgical procedure that could be particularly indicated in the elderly and in patients with concomitant respiratory disease. This technique is easily performed, allows an operative cholangiogram, and make compare favourably with the newer non-operative treatments for cholelithiasis.

FP222 LASERS IN THE SURGERY OF LIVER AND BILE DUCTS.

Litwin G.D.,

Kirpitchev A.G., Yakimenko A.P.

(Research Institute for Laser Surgery, USSR Ministry of Public Health,

Director Professor Skobelkin O.K.)

Using laser irradiation we have performed surgeries on bile ducts in 670 patients and on liver in 42 patients. We used Soviet CO-2 and YAG-laser devices with specially designed laser surgical instruments and accessoirs. Laser beam was used during different periods of operations (incision of abdominal wall, mobilization of gallbladder and coagulation of gallbladder bed, choledochotomy, biliodigestive anastomosis, liver resection and treatment of cavities after hydatidectomy). It is due to the use of laser beam that we managed to simplify the technique of surgical interventions, decrease trauma and control bleeding. The time of operation was not considerably increased. There were no specific complications. The achieved experience enables us to conclude that the use of laser beam serves for further development of the techniques of surgical interventions on bile ducts and liver and improve the results of surgical treatment. We also point out the promissing use of YAG-lasers.

**FP223** ROUTINE OPERATIVE CHOLANGIOGRAPHY. STILL IN PRACTICE?

BLIOURAS N., SKALTSAS S., KOURTESSIS A., HAMALAKIS G., KEKIS B.

ATHENS MEDICAL CENTER, ATHENS, GR.

Operative cholangiography (OC) was first described by Mirizzi over 50 years ago. The advantages of routine OC during cholocystectomy have been demostraded in numerous studies, although there is still controversy about whether it should be used selectively or routinelly. The aim of this study is to assess the technique and the practical value of routine OC in a large series of cases.

Operative cholangiography was performed on a routine basis in 1680 patients undergoing cholecystectomy for gallstones. All these, were elective operations without evidence of pathology of C.B.C. Eighty six patients (5,1%) where interpreted as having abnormal OC, common bile duct exploration was performed and the procedure revealed 67 patients with stones (3,9%) and 19 false positive examinations (1,13%). One year follow-up confirmed 8 false negatives O.C. examinations (0,47% of mentiont above 1680 patients).

Routine OC provides a map of the biliary system, it reveals coincident biliary disease and the frequent practice of the technique "makes perfect". Also reduses both the incidence of stones retained in the common bile duct after cholecystectomy and the incidence of negative common bile duct explorations.

The benefit of routine use of OC is the diagnosis of silent or unsuspected stone. Most authors give an incidence of 1-7%. The disadvantage with routine OC is the false-positive percent. To reduce this, meticulous attention to the technical aspects of performing the examination is essential for high quality radiographs and this can be achieved only by cooperation between the surgeon and radiologists.

### FP224 ARGUMENTS FOR SELECTIVE INTRAOPERATIVE CHOLANGIOGRAPHY

R.Engemann, D.Pickuth, P.Lubinus, E.Schweizer, W.Ahlendorf*, H. Hamelmann, Dept. of General Surgery and of Radiology*, Univ.- Hospital, D-2300 Kiel

Routine use of intraoperative cholangiography (IC) in elective cholecystectomy is still widely advocated and standard in our department, however is also discussed controversialy. The aim of our study was to prospectively investigate the impact of modern diagnostic concepts for cholelithiasis on the value of IC.
120 patients, undergoing cholecystectomy, were prospectively screened for the presence of six predefined risk indicators of choledocholithiasis: history of jaundice, pancreatitis, hyperbilirubinemia or hyperamylasemia, a dilated bile duct, or unclear findings in ultrasonography (US). We also evaluated the sensitivity of US, and of preoperative cholangiography (PC) and IC in the diagnosis of gallbladder and bile duct stones.
For the detection of gallbladder stones the sensitivity was 100% for US, 89% for cholangiography. Concerning the bile duct, we found a US sensitivity of 77%. Both PC and IC had a sensitivity of 100%. 2o% of all patients had at least one risk indicator. The presence of a risk indicator correlated significantly with the presence of choledocholithiasis (p< 0,01, chi-square-test). The negative predictive value of the total set of risk indicators was 100%.
Following our diagnostic concept, we could have avoided 80% of the IC without missing a stone in the bile duct. Thereby, costs and X-ray exposure could be reduced. PC or IC is recommended in all patients with one or more risk indicators or unclear findings intraoperatively. Routine use of IC in patients undergoing elective cholecystectomy is no longer necessary.

FP225

### MANAGEMENT OF BILIARY FISTULA ASSOCIATED WITH POST-CHOLECYSTECTOMY BILE DUCT INJURY

W. Schweizer, H.U. Baer, P. Gertsch, A. Huber, J. Triller, U. Scheurer, L.H. Blumgart

Department of Visceral and Transplantation Surgery, Inselspital, University of Berne, Switzerland

The incidence of bile duct injury during cholecystectomy is 0.1 - 0.3%. Of 175 patients with biliary stricture following cholecystectomy 28 had an established external biliary fistula. 13 of these had been submitted to multiple previous operations and in 9 a biliary repair had already been performed.

In 3 patients immediate drainage was performed because of intra-abdominal biliary collections. 19 patients had biliary obstruction distal to the fistula. In 6 cases definitive operation was performed early after admission. In the remaining 22 patients treatment was initially conservative: In 9 of these the fistula closed spontaneously within 3 to 9 weeks and followup at 10 to 48 months revealed no evidence of continuing biliary disease. 9 patients were subsequently submitted to operation because of strictures with jaundice or cholangitis. The mean interval between fistula development and operative repair was 3 months. In 4 patients a prepapillary concrement was removed endoscopically. 22 patients remained asymptomatic with normal liver function tests at the mean followup of 24 months.

Biliary fistula associated with intraperitoneal collections of bile should be managed by initial drainage and control of the fistula with subsequent repair if necessary. In a fistula with distal obstruction operative management is indicated. Endoscopic intervention offers an alternative in cases with prepapillary concrements.

In established fistula without distal obstruction conservative management will result in the closure of a significant proportion of cases.

FP226 PERCUTANEOUS CHOLECYSTOSTOMY AND CHOLELITHOLYSIS USING METHYL TERTIARY BUTYL ETHER. EXPERIMENT AND CLINICAL APPLICATION

Qinghua-Zhu, Guoshan-Su, Yongjia-Wu,
Dept of Surgery, Second Hospital,
Shanxi Medical College, Taiyuan,
People's Republic of China

Percutaneous transhepatic gallbladder drainage (PTGBD) was performed by using self-made trocars and tubes in 30 high risk patients with acute cholecystitis or cholangitis and the results were satisfactory. In patients with acute cholecystitis PTGBD was performed under ultrasonic guide. In selective patients cured by cholelitholysis, PTGBD was performed using oral cholecystography to show the gallbladder under fluoroscopic guide. The gallstone dissolution and influencing factors were studied in vitro and in removed gallbladders by self-made methyl tertiary butyl ether (MTBE). In vivo, the experiment demonstrated that MTBE is a safe and rapid gallstone dissolvent. Three cases of gallstones and six cases of residual stones in the common bile duct were treated with an infusion of MTBE through the percutaneous transhepatic cholecystomic tube and the T tube in situ respectively. Indications, toxicities and side effects as well as measures enhancing the efficacy of gallstone dissolution were discussed.

FP227 GALLBLADDER FUNCTION FOLLOWING ENDOSCOPIC SPHINCTEROTOMY

LA Desa, PA Grace, M Vipond, B Henderson, J Thompson. Royal Postgraduate Medical School, Hammersmith Hospital, London, UK

Endoscopic sphincterotomy without cholecystectomy is currently often the only treatment for patients with choledocholithiasis. Post-sphincterotomy gallbladder function, however, has not been studied. We thus undertook a prospective study of gallbladder function in six patients, following endoscopic sphincterotomy, utilising excretion biliary scintigraphy. Patients were studied if they had 1) a previous endoscopic sphincterotomy for ductal stones, 2) a patent cystic duct with gallbladder filling on endoscopic cholangiography, and 3) cholangiographically confirmed ductal stone clearance. None of the scintigrams showed gallbladder filling, despite delayed (120 mins) imaging.

This study shows a complete lack of gallbladder filling with post-sphincterotomy HIDA scanning, despite recent evidence of cystic duct patency. This is presumably a consequence of preferential bile flow through the incompetent lower common bile duct sphincter, the lack of any functional obstruction preventing reflux of bile into the gall-bladder.

This finding has important implications for 1) managing residual gall-bladder stones with dissolution therapy or extracorporeal shockwave lithotripsy, 2) promotion of chronic infective changes, even malignancy in the in situ gallbladder, 3) the usefulness of post-sphincterotomy HIDA scanning in evaluating gallbladder function, and 4) predicting changes in the bile acid turnover.

FP228 PROSPECTIVE STUDY ON CHANGES OF GALLBLADDER FUNCTION IN RESPONSE TO EXOGENOUS CHOLECYSTOKININ AFTER GASTRECTOMY

S.HIGASHIDE, K.INOUE, S.SUMI, R.DOI, A.FUCHIGAMI, H.MINOTE, K.TAKAORI, M.YUN, K.UCHIDA, T.TOBE
First Department of Surgery, Faculty of Medicine, Kyoto University, Kyoto, Japan

Although the occurrence of gallbladder dysfunction after gastrectomy has been reported, the mechanism for this phenomenon remains uncertain. The present study was performed to observe changes of gallbladder contraction in response to exogenous cholecystokinin (CCK) before and after gastrectomy.
Methods: After an overnight fast, bolus injection of caerulein (0.2µg/kg, intramuscularly) was given to patients with gastric cancer before(n=35), 1 month(n=27), 3 months(n=25), 6 months(n=26), and 12 months(n=30) after gastrectomy. Sonograms of the gallbladder were obtained by use of a realtime ultrasound unit at 10 min interval for 120 minutes. The gallbladder areas were measured by planimeter from photograms and were expressed as percentages of the area before injection.
Results: Maximal contraction rate of the gallbladder was signifcantly decreased 1 month after gastrectomy (55.2±5.8% of pregastrectomy value) compared to before gastrectomy (calculated as 100 %). Thereafter, maximal contraction rate was gradually recovered, showing 80.6±5.0%, 88.3±3.1%, and 91.7±7.4% at 3 months, 6 months, and 12 months after gastrectomy, respectively.
Conclusion: This study demonstrates that the sensitivity of gallblader to exogenous CCK is decreased after gastrectomy but recovered within 1 year, suggesting that CCK plays an important role in the gallbladderkinetics after gastrectomy.
Acknowledgement This study was supported by a grant from the Ministry of Education, Japan (#A,61440060).

### FP229 AN AUSTRALIAN EXPERIENCE WITH BILIARY AUDIT

**C.Battersby, A. Askew**
**Royal Brisbane Hospital - Australia**

Whether we like it or not, surgical audit will become increasingly important in clinical practice. Broadly, it may be used to indicate the outcome of surgical treatment; that is— morbidity and mortality. However, within this basic framework, cost effectiveness of a given method of treatment, the management of specific conditions (e.g. infective and other complications), the effect of certain interventions (e.g. endoscopy) and long-term results can all be monitored and analysed.

Experience will be presented from an audit of 290 biliary operatons for stone undertaken since 1986. Difficulties with the questionnaire and data collection will be discussed, and examples given of how the audit modified clinical practice - for example, with antibiotic policy. It was found that :

(a) Ultrasound had almost entirely replaced oral cholecystography in diagnosis.

(b) There was a duct exploration rate in cholecystectomy of 11.2%.

(c) The re-operation rate for retained bile duct stone was 2.4%.

(d) Positive gall bladder cultures were obtained in 23% of patients.

(e) There was an overall post-operative infection rate of 8.4%.

The conclusions are :

(a) It is important to define the object of the audit and avoid the temptation just to collect information.

(b) Someone must be vitally interested and take the time constantly to scrutinize performance.

(c) Methods should be redefined from time to time to allow for specific studies.

### FP230 POSSIBLE ETIOLOGY OF LITHOGENESIS AFTER TOTAL GASTRECTOMY AND TOTAL PARENTERAL NUTRITION

L. Sarli, M. Gafà, N. Pietra, E. Longinotti, F. Carreras, A. Peracchia. II Clinica Chirurgica, Universita di Parma, Italy

We recently observed a very rapid biliary lithogenesis after total gastrectomy (TG) and postoperative total parenteral nutrition (TPN) (1). Our observtion leads us to suggest that the lithogenesis concerns pigment stones. In the present study we evaluated bile composition and gallbladder contractility to provide information as to the possible etiology of this kind of lithogenesis. Serial ultrasonographic measurements of gallbladder diameters and bile analysis, using duodenal drainage, were performed every 12 hours during TPN and every day for 3 days after oral refeeding in 16 patients who underwent TG + TPN and in 12 control patients (6 gastric resections, 3 truncular vagotomies, 3 explorative laparotomies) who did not undego TPN. In all patients gall-bladder contraction was obtained using caerulein before oral refeeding to carry out gallbladder bile analysis. No patients showed cholelithiasis or biliary sludge at pre-operative sonography. None showed cholesterol crystals or pigment granules at analysis of gallbladder bile samples obtained during surgery. Analysis of bile before caerulein administration showed aggregates of pigment granules in all TG + TPN patients with sludge and only in 1 control; cholesterol crystals in 2 TG + TPN patients and in no controls. Analysis after caerulein showed pigment granules in all sludge positive patients and cholesterol crystals in 7 TG + TPN patients and in 1 control. Aggregates of pigment granules were found in 2 TG + TPN sludge negative patients before caerulein and in 4 TG + TPN and 1 control sludge negative after caerulein. Gallbladder diameters increased in all patients during fasting and only in 10 TG + TPN patients after oral refeeding.

These results lead us to suggest that the biliary pigment lithogenesis we observed in TG + TPN patients results from multiple factors working in concert: gallbladder stasis, bowel rest and biochemical changes of bile induced by TPN. On the contrary, none of these factors is by itself sufficient to induce lithogenesis.

(1) Gafà M., Sarli L., Miselli A., Pietra N., Carreras F., Peracchia A. Surg. Ginecol. Obstet. 1987, 165; 413-8.

FP231 OPERATIVE CHOLEDOCHOSCOPY - THE EFFECTIVE METHOD OF COMMON BILE DUCT EXPLORATION

D.Menzies and R.W.Motson, Colchester General Hospital, Colchester, UK.

Despite the recognised advantages of common bile duct stone identification with choledochoscopy a recent representative survey of British surgeons revealed that only one third routinely use choledochoscopy at common bile duct exploration (Cahill and Pain 1988).

A prospective analysis of 75 consecutive choledochoscopies has been performed to assess the effectiveness of the flexible choledochoscope in detection of common bile duct stones in a District General Hospital. Over a 5 year period 75 choledochoscopic common bile duct explorations have been performed. Following exploration T-tube cholangiography was used to identify missed common bile duct stones.

There were no cases of missed common bile duct stones. Two patients had common bile duct stones identified at choledochoscopy that proved impossible to remove. Both patients had transduodenal sphincteroplasties and subsequent cholangiography demonstrated clear bile ducts. No patient suffered any complication due to choledochoscopy.

Choledochoscopy is an effective, safe, and easy means of common bile duct exploration and should be the method of choice for this procedure.

References:

Cahill CJ, Pain JA. Br J Surg 1988:75,1169-72.

FP232 EARLY EXPERIENCE WITH EXTRACORPOREAL SHOCK-WAVE LITHOTRIPSY FOR GALLBLADDER CALCULI IN THAILAND

A. Pausawasdi
Siriraj Hospital, Bangkok, Thailand

Between June and November 1989 twenty patients with gallbladder calculi were treated by extracorporeal shock-wave lithotripsy (ESWL) alone in the Department. Piezoceramic elements (Wolf system) were used to generate shockwave energy. There were 3 men and 17 women. All stones were radiolucent, and in each case oral cholecystography demonstrated a functioning gallbladder (i.e. a patent cystic duct). Fragmentation of calculi was successful in 4 cases (20 per cent) and unsuccessful in the remaining 16. In each successful case the calculus was solitary and less than 5 mm in diameter; in 3 of the 4 it disappeared from the gallbladder within 3 weeks. Each of these patients had complete relief from biliary colic. In 8 of the 16 patients with unsuccessful lithotripsy, cholecystectomy has already been performed. All but one of these patients had multiple black pigment stones. In one patient a severely traumatised gallbladder was encountered at operation containing 150 ml of clotted blood within its lumen. This preliminary study indicates a limited role for ESWL in patients with multiple pigment gallstones which are prevalent in South East Asia.

FP233 GALLSTONE LITHOTRIPSY
TO SHOCK OR NOT SHOCK!

A. Darzi, E. El-Sayed, C. O'Morain, W.A. Tanner, F.B.V. Keane, Department of Surgery, the Meath Hospital, Dublin.

Extracorporeal shock wave lithotripsy (ESWL) is successful in fragmenting gallstones but less than 28% of patients with gallstone disease fulfil the criteria considered suitable for treatment. However no data exists to substantiate these conventional selection criteria. In this study, we employed a second generation EDAP lithotripter combined with dissolution therapy. The selection criteria were broadened to include patients with radiolucent stones of any size and number, and radioopaque stones less than 3 cm in diameter. To date 108 patients with gallstones have received treatment. All patients received up to six sessions of ESWL (6000 shock waves per session) as an outpatient procedure without sedation or analgesia. Early results have demonstrated complete gallstone clearance in forty nine patients (mean follow-up 6.2 months). The clearance rates were 10 percent within 2 months, 21 percent at 2-4 months, 38 percent at 4-8 months, 60 percent at 8-12 months, and 78 percent at 12-18 months. Of those patients with a successful outcome only 19 would have satisfied traditional selection criteria. There have been no significant complications except for one patient with mild acute pancreatitis that settled on conservative treatment, and two patients with acute cholecystitis. This study would suggest that the previously accepted selection criteria underestimate the number of patients suitable for gallstone ESWL and dissolution therapy.

**FP234** EXTRACORPOREAL PIEZOELECTRIC LITHOTRIPSY (EPL) FOR ACTURE OBSTRUCTIVE CHOLECYSTITIS

M Filimonov, Yu Starkov, V Vasilyev
2nd Medical Institute, Moscow, USSR

For the first time now EPL with the help of Wolf-Piezolith-2300 was used for treating 18 patients suffering from acute obstructive cholecystitis. 12 of them suffered from catarrhal cholecystitis and 6 from the phlegmonous form. EPL was performed within 3 days of the patient's admission with the purpose of unblocking the cystic duct, decompressing the gallbladder and finally relieving the acute cholecystitis.

The treatment was successful in 14 of 18 (78%) cases. Partial or total fragmentation of the impacted cystic duct stones and restoration of biliary drainage were achieved. Successful removal of obstruction invariably led to recovery from acute cholecystitis. In 8 cases - the patients suffering from catarrhal cholecystitis for up to 8 days - gallbladder contraction was restored after the acute phase was over. Small focus size, lack of damage to the surrounding tissue (Neisius et al, 1989), and painless lithotripsy with the Piezolith-2300 (Ell et al, 1988) allowed treatment of acute obstructive cholecystitis using extra-corporeal lithotripsy.

References:
D Neisius et al. J Lithotripsy & Stone Dis, vol 1 1989 26-33
Ch. Ell et al. N Engl J Med 319 (1988) 371-372

### FP235 A COMPARISON OF THE SHOCKWAVE FRAGMENTATION CHARACTERISTICS OF CHOLESTEROL AND PIGMENT GALLSTONES.

P. Goh, D. Ngiam, E. Sim
National University of Singapore, Singapore

The fragmentation characteristics of cholesterol gallstones have been well studied. However, the effect of shockwaves on pigment stones is an important consideration in the Orient where these stones constitute the majority of biliary stones.

The in vitro effects of shockwaves generated by a piezo-electric EDAP stone lithotripter on 20 cholesterol and 20 black pigment stones were studied. The machine was fired at 100% power and 2.5 Hz output. The stones were all of generally the same size and shape with a largest diameter ranging from 8.5 to 15 mm. Mean diameter of pigment stones was 10.18 mm and that of cholesterol stones was 10.50 mm. Composition of the stones were confirmed by biochemical analysis. The stones were immersed in air free distilled water suspended in condoms of one particular brand and specification.

The cholesterol stones were shattered into a fine powder in 40 to 80 minutes (mean 68 minutes). The pigment stones were shattered into a coarser powder in 40 to 50 minutes (mean 45 minutes). After fragmentation, no particle was larger than 2 mm which is the size required to pass easily down the cystic duct.

In conclusion, the study shows that black pigment stones can be shattered by shockwaves more easily than cholesterol stones. This has important implications in the wider use of extracorporeal shockwave lithotripsy in Oriental patients with gallstones.

References:
Newman RC et al. J Surg Research 1988; 44:573.
Staritz M et al. Euro J Clin Inves 1989; 19:142.

### FP236 GALLSTONE LITHOTRIPSY - WITH OR WITHOUT DISSOLUTION THERAPY

A. Darzi, C. O'Morain, W.A. Tanner, F.B.V. Keane.
Dept of Surgery, The Meath Hospital, Dublin.

Following extracorporeal shock wave lithotripsy (ESWL), it is not known whether fragments can be cleared from the gallbladder without the use of oral dissolution therapy (DT). In order to prospectively assess the efficacy of ESWL and DT, used alone or in combination, we randomised 31 patients to one of three treatment groups: ESWL alone, DT alone or ESWL and DT. All patients had symptomatic gallstones, functioning gallbladders, and the stone profile were comparable in each group. ESWL was administered using a piezo-electric lithotriptor (EDAP LT-01), and DT consisted of combined bile salt and terpene administration. Clearance was assessed using a combination of ultrasound and oral cholecystography. Each Patient was followed up for six months and those with less than 50% clearance at the end of 6 months were considered failures.

| Group | n= | Clearance Total | Partial (>50%) | Failures |
|---|---|---|---|---|
| ESWL | 11 | 0 | 0 | 11 |
| DT | 10 | 1 | 1 | 8 |
| ESWL+DT | 10 | 5 | 2 | 3 |

The number of patients with total or partial clearance in the ESWL+DT group was significantly greater than those in either DT ($p<0.02$) or ESWL alone ($P<0.05$). ESWL merely fragments gallstones, and DT is essential to achieve fragment clearance. This study demonstrates for the first time that gallstone clearance following ESWL appears to be DT dependent, and combination of the two modalities, is more potent than DT alone.

FP237 ULTRASONICALLY GUIDED PERCUTANEOUS TRANSHEPATIC MICROCHOLECYSTOSTOMY

B S Briskin, I B Kaprov, I R Platova
A M Minasyan
The Semashko Moscow Medical Institute
USSR

Ultrasonic control of transcutaneous transhepatic microcholecystostomy (RTM) has been an alternative to surgical cholecystostomy for the last 10 years. Since 1987-1989 we have performed 72 RTM with ultrasound control.

All surgical procedures were carried out using Bruel et Kjaer. 1846 sector puncture gauge working at 3 mHz. The drainage was performed by a one-stage method with 3 mm diameter stylet catheter with the basket at the end. RTM was indicated in 63 patients because of acute cholecystitis with high risk, and nine patients with acute acalculous (vascular) cholecystitis. Puncture drainage of perigallbladder abscess was performed in addition in 3 patients with acute cholecystitis. Transhepatic puncture of gallbladder under ultrasonic control was done in 14 patients suffering from acute calculous cholecystitis. After RTM and drainage of the gallbladder pain was controlled immediately. After control of the attack 32 patients underwent delayed surgical treatment; the remaining patients were discharged from the hospital without surgical procedures. After RTM in 1/1, 3% patients there was bile leakage into the abdominal cavity. They were treated surgically. No mortality was observed.

**FP238** NEW PROSPECTS IN GALLSTONE DISEASE SURGERY.

Brekhov E.I.,Kuleshov I.Y.,Severtsev A.N.
Surgical Clinic.Hospital N°51.Moscow. USSR.

Gallstone disease is a serious surgical problem demanding urgent as well as non-urgent surgery. Gall ducts and gallbladder operations require methods which are very often traumatic and technically difficult and may lead sometimes to the fatal outcome. That is why the working out of new surgical methods is very important for surgeons.

A number of new surgical methods worked out and used in our Clinic. Clinical experience is assessed in this work.

2765 gallstone operations were performed from 1984 through 1988. 31% of operations was performed urgently. 42% of patients developed complications of the main pathology.

1. A method of bloodless and quick laser photohydraulic cholecystectomy was worked out and used in our Clinic

(164 operations). 2.Special laser instruments were used in gall ducts operations. It saved time and make operations less traumatic (60 operations). 3.Methods of triangular cholecystoentero- and choledochoentero - anastomoses were worked out and used (15 operations). Linear "UDO-type" suture devices were used in operations. 4.Plasma flows were used in 56 patients in order to stop bleeding from the gallbladder bed. This permitted not to suture the gallbladder bed, to economize time and to simplify the operation significantly.

1.7% of patients died in post-operative period. All the fatal cases had complications of the main pathology and/ or severe concomitant diseases.

**FP239** PANCREATIC DUCT DRAINAGE WITH OR WITHOUT RESECTION FOR CHRONIC PANCREATITIS.

J.Van de Stadt, M.Cappello, M.Gelin,
M.Cremer, J.P.Lambilliotte.
Hôpital Erasme, ULB, Brussels, Belgium.

Out of 563 patients with chronic pancreatitis investigated at our institution from 1978 to Dec.1988, 77 underwent surgical pancreatic duct drainage. Systematic preoperative work-up included an endoscopic retrograde cholangiopancreatography associated with a computerized axial tomography or an ultrasonography of the pancreas Chronic pancreatitis was graded according to our previously described morphological classification (Cremer et al 1976),based on endoscopic pancreatography. Indication for surgery was intractable pain in a 96% of the cases. A side-to-side pancreaticojejunostomy without pancreatic resection was performed in a 54 patients and a pancreaticojejunostomy after resection of the tail of the pancreas in 23 patients. Intraabdominal surgical complication rate was 18%. There was no postoperative mortality. One year successful pain relief was achievedin 90% of the cases. Of 54 patients available for follow-up study, with a median duration of 18 months (range 6 months - 10 years), 26% (14/54) experienced relapsing major pancreatic pain, whatever the type of drainage performed. During this follow-up, diabetes and steatorrhea were worsened or de novo developed in 75% and 62.5% respectively after drainage associated with caudal pancreatectomy, when only in 30% and 17.5% respectively after conservative drainage ($P < 0.01$). Relapsing major pancreatic pain was treated by endoscopic therapy in 10 patients (sphincterotomy and/or prosthesis 9, cystoduodenostomy 1), or extracorporeal shock wave lithotripsy for pancreatic calculi in 4 patients with complete or partial pain relief in 3. Three patients were reoperated on: cystojejunostomy 2, combined cysto, biliary and gastric drainage 1. Six patients died during the follow-up, from causes unrelated to the pancreatic disease.
<u>Conclusion</u>: In our experience drainage surgery was effective in relieving intractable pain from chronic pancreatitis. Since drainage associated with caudal pancreatectomy was not found to improve pain control as compared with drainage alone, but well to increase endocrine and exocrine insufficiencies, side-to-side pancreaticojejunostomy should be considered as the technique of choice each time it is possible.

References: Cremer et al. Acta Gastro-Ent. Belg. 1976; 39: 522.

**FP240** CHRONIC PANCREATITIS:SURGICAL TREATMENT AND EARLY POSTOPERATIVE COMPLICATIONS

I.Klempa, I.Baca, H.Morr, J.Menzel

Allgemein-Chirurgische Klinik, ZKH

St.-Jürgen-Straße, 2800 Bremen, FRG

From 1983 to 1989, 91 patients with chronic pancreatitis underwent surgery in our department. They were 66 men and 25 women with 51±11 and 49±18 resp. years of age. Common symptoms were recurrent pain and symptomatic pseudocysts. 14 patients (15%) had primary diabetes mellitus. 25 patients showed clinical signs of jaundice and/or laboratory data of cholestasis. Except for pseudocysts, resective procedures were preferred. Cystojejunostomie or pancreatojejunostomie after distal resection were done in our own technique with neither Braun nor Roux-en-Y anastomosis. 29 patients underwent partial duodenopancreatectomy, 13 distal resection, 10 duodenum-preserving resection of the head of the pancreas, 4 patients had pseudocyst resection, 27 underwent internal cyst drainage, 4 had sphincteroplasty and 4 only a drainage procedure.
2 patients died postoperatively (2,1%), one from peritonitis due to additional resection of the transverse colon, the other one from sepsis due to pseudomembranous colitis. 6 patients had intestinal haemorrhagy during the first postoperative days, in 4 patients reoperation was necessary. 11 patients(12%) developped diabetes postoperatively. 3 patients showed respiratory insufficience and needed respirator treatment. A fistula from the pancreatic anastomosis developped in two patients. 3 patients underwent reoperation because of intraabdominal abscess. Overall postoperative morbidity was 27 %. Our results indicate, that radical resection as well as our own easy-to-do anastomosis for pseudocysts have a place in the surgical treatment of chronic pancreatitis and do not present particular risks.

FP241 IMMEDIATE AND LONG TERM RESULTS AFTER SURGICAL TREATMENT OF 127 CASES OF CHRONIC PANCREATITIS (PC)

B. SASTRE,B.CARABALONA,B.CRESPY,JC.SARLES, G.MICHOTEY - Hôpital Ste Marguerite, bd de Ste Marguerite Marseilles (FRANCE)

From 1960 to 1987, 127 patients (105 males and 22 females, mean age: 45,9) underwent surgical treatment for PC. The aim of this study is to assess the results of the surgical treatment intentionally turned towards conservative surgical procedures (CSP).91 patients benefited from either pancreato-digestive bypasses (84 cases) sometimes associated with other digestive bypasses and/or transhiatal splanchnicotomy (THS) or isolated biliary (5 cases) or gastric (2 cases) bypasses. 31 resections were carried out : 26 pancreatoduodenal resection (PDR) associated 3 times with TSH and 5 distal pancreatectomies. The conservative treatment was of other types in 5 cases. There were 5 post-operative deaths (3,9%), 1 after resection (6,6%) and 4 after CSP (p>0,7). Postoperative complications occured twice after resections (6,6%) and in 13 cases (16,2%) after CSP (p>0,3). A further surgical procedure was required in 3 cases after pancreatic resection (3/25,12%) and in 14 cases after CSP (14/71,19,7%) (p>0.5). In the late postoperative course 15 deaths occured but only 6 of them were directly bound to the evolution of the pancreatitis. 5 and 10 years overall survival probability after surgical treatment was respectively 81,5% and 64,7%. This probability was 70,4 and 60,4% after resections and 87,1% and 68,8% after CSP (p=0,29). After CSP 75% of good functional results were observed between 1 and 4 years and 60% afterwards. Although non statistically significant those results suggest that : - CSP and resections have the same operative risk, - late reoperations are more frequent after CSP,- the chance of late survival rate is better after CSP than after resections. Though it appears worthwhile to favor as far as possible CSP in the choice of surgical treatment of the PC.

**FP242** MINIMAL CHANGE CHRONIC PANCREATITIS

T.N. Walsh, J. Rode, B. A. Theis,
R.C.G. Russell.

The Middlesex Hospital, Mortimer Street,
London WIN 8AA.

The severity of pancreatic pain appears to be unrelated to the severity of damage found in the pancreas on imaging or histology. Amongst patients with severe pancreatic pain, there is a group of patients with minimal or equivocal findings on pancreatic investigation.

43 such patients referred by consultant gastroenterologist (68%), physician or surgeon (24%) and GP (8%) have been studied. 16 male [mean age and range in years at onset 30, 13 - 59] and 27 female [37, 14 - 65] patients described severe constant epigastric pain radiating to the back, aggravated by food, alcohol and associated with nausea and vomiting. Analgesic requirements were mild in 23%, moderate in 40% and heavy in 37%. Personality assessment was normal in 24 (60%) but possibly abnormal in 16, displaying aggressive, addictive, inadequate or depressive tendencies. Eleven had psychiatric assessment of whom 10 were assessed as normal. Investigations included ERCP (42), ultrasound (43) and CT (12), revealing pancreas divisum (7) or a prominent duct on ultrasound (6) but otherwise normal (21) or only equivocal changes (22). Fifteen patients underwent resection, as a primary procedure (7) or following unsuccessful drainage (8) and in two drainage only, resulting in improvement in 9, no improvement in 7 and deterioration in 1. Of those managed conservatively 15 are improved, 10 are unchanged and 1 worse. Histology of resected specimens revealed subtle changes in all but one pancreas of mild periductal fibrosis, duct proliferation, duct complex formation, vacuolation of the acinar cell or adenomatous nodule formation.

It is suggested that there is a syndrome of minimal change pancreatitis with defined clinical features and a distinct histology; such patients respond badly to surgical treatment and should, if possible, be managed non operatively.

**FP243** EXTRACORPOREAL SHOCK WAVE LITHOTRIPSY OF PANCREATIC DUCT STONES

Nijs HGT[1], Toom R den[1], Blankenstein van M[2], Schröder FH[3], Terpstra OT[1].

Depts. of [1]General Surgery, [2]Gastroenterology and [3]Urology, Univ. Hosp. Rotterdam (The Netherlands).

Chronic calcifying pancreatitis forms a major clinical problem, often requiring extensive surgery. Extracorporeal shock wave lithotripsy (ESWL) offers a new therapeutic option in this matter. We applied ESWL after endoscopic sphincterotomy to 7 patients (3M/4F, mean age 42 (range 22-55) yrs) with one or more impacted pancreatic duct stones (diameter largest stone 22 (12-30) mm). The Siemens Lithostar (Erlangen FRG) was used, with patients in prone position under fluoroscopic control. 4500 (1500-8000) discharges were delivered in 1-2 sessions. Successful disintegration of stones was achieved in 5/7 patients, relief of pain in 6/7 patients, total clearance of the pancreatic duct in 2/7 patients. Mean serum amylase at discharge was 101 U/l vs. 180 U/l before ESWL ($p<0.05$). One patient had an exacerbation of her pancreatitis immediately after ESWL, which resolved with medical management. None of the patients with fragmented stones had abdominal complaints at follow-up (mean 6 (1-16) months). In the 2 patients without stone disintegration an operation according to Puestow was carried out. Of these patients, one subsequently needed a partial pancreatectomy; the other patient still has chronic abdominal pain 16 mo after surgery. In conclusion: ESWL of pancreatic duct stones in combination with endoscopic techniques is the therapy of choice before considering surgery.

### FP244 UNUSUAL PRIMARY TUMOURS PRESENTING AS CANCER OF THE HEAD OF THE PANCREAS

G. LEBEAU, G. JAMIESON, M.P. RAMEE, J.P. CAMPION, B. LAUNOIS

Clinique chirurgicale CHU RENNES France

Cancer of the head of the pancreas is universally regarded as having a poor prognosis regardless of the type of therapy used. From 1972 to 1989, we have performed 116 "curative" resections for putative carcinoma involving the pancreatic head. In 10 cases when the resected specimen was subjected to full histology post-operatively, the pre-operative and intra-operative diagnosis was proven wrong. The correct diagnosis was : endocrine tumour of pancreas (4 cases) and one case each of malignant polyp of Gardner's syndrome, colloid carcinoma of pancreas, hemangiosarcoma of pancreas, lymphoma, anablastic carcinoma and schwannoma. In the 10 patients, 8 were female and the mean age for the group was 50 years ($\pm$ 13). Eight of the patients underwent a pancreaticoduodenectomy and two a total pancreatectomy. There was no post-operative mortality nor significant morbidity. The actuarial 5 year survival for the 10 cases is 42 %, compared to 8 % for our cases of cancer of the head of the pancreas. 6 patients are currently alive and well at a mean of 56 months since their operation.

In the absence of a definite pathological diagnosis at the time of surgery, we attempt to resect all tumours involving the head of the pancreas. We believe the experience reported here confirms this approach because apparently highly malignant tumours of the pancreas sometimes prove to be unusual tumours for which resection offers the prospect of cure.

**FP245** UNCOMMON TUMORS OF PANREATIC GLAND

C. Pasquali, C. Sperti, M. Rugge^
F. Di Prima *, D. Infantolino §,
S. Pedrazzoli. Clinica Chirurgica 1-
Padua, Radiology*, Pathology ^§
Cittadella^ & Castelfranco§, Italy

From 1964 to 1989 we observed in our Department 438 patients with neoplastic mass of the exocrine pancreas and 106 pancreatic apudomas (19.5% of 544 total cases of pancreatic neoplasms). Excluding insulinomas,gastrinomas and ductal adenocarcinomas, 38 (7%) of patients had a rare type of pancreatic tumor commonly referred or suspected to be of ductal origin. These included 13 cystic tumors, 13 acinar cell carcinomas, 8 clinically silent apudomas, 2 secondary tumors,1 sarcoma and 1 schwannoma. Among cystic tumors (7 adenomas, 5 adenocarcinomas, 1 papillary cystic tumor), 2 had liver metastases and 2 were unresectable; all adenomas and the papillary were resected. Among 13 aciner cell cancers 4 had liver spread and 7 local infiltration; 3 underwent resection. The patient with sarcoma had only exploration and radiotheraphy.The malignant schwannoma was resected despite extensive local involvement to surrounding organs. 2 secondary tumors (leiomyosarcoma and melanoma) were resected. 5/8 apudomas had liver metastases and all but 1 underwent resection of all gross tumors.
Excluding 7 benign cystic tumors 2/31 patients died after 24 months and 10 (33%) patients survived at least 2 years (3-8 years) after surgery .The malignant schwannoma is living 6 months after surgery without recurrence.
In 8.6% of cases pancreatic tumor supposed to be before surgery of ductal origin has a different histology and behaviour. In 58% of cases a resection is possibile and long term survival was not exceptional despite extensive disease may be present.

Study supported by CNR grant. Project "Oncology" # 88-00804.44

FP246 PANCREATICODUODENECTOMY FOR PERIAMPULLARY TUMORS OTHER THAN PANCREATIC DUCTAL CARCINOMAS

M. H. Shiu

Memorial Sloan-Kettering Cancer Center

New York, N.Y., U.S.A.

The therapeutic outcome and safety of pancreaticoduodenectomy for periampullary tumors other than pancreatic ductal carcinoma are examined in a retrospective study of 31 consecutive patients treated by the author in 1976-1989. There were 27 malignant tumors, and 4 benign lesions.

Pancreaticoduodenectomy was performed using a standardized technique (Shiu 1982, Shiu 1986). Pancreatic secretions were temporarily excluded or diverted from the pancreatic, biliary and gastric anastomoses by a purse-string tie of 3/0 polyglycolic acid suture on the pancreatic duct, with (n=16) or without (n=15) a polyethylene stent that drained externally.

The median postoperative hospital stay was 19 days (range 14-40). No patient died of the operation and none developed a pancreatic or biliary fistula. Complications included gastrojejunal stomal ulceration in 1, biliary tract sepsis in 2, wound infection in 2 and acute prostatic obstruction in 1. Chronic steatorrhea developed in 1 patient resected for chronic pancreatitis. Death due to metastasis occured in 13 patients at a median of 13 months.

| Survival Rate (Malignant lesions) | N= | 1-yr. | 2-yr. | 5-yr. |
|---|---|---|---|---|
| Ampulla of Vater | 8 | 7/8 | 6/7 | 2/3 |
| Common Bile duct | 5 | 5/5 | 3/3 | 0/3 |
| Duodenum | 4 | 3/4 | 2/4 | 1/4 |
| Stomach (invading duod.-panc.) | 3 | 2/3 | 1/3 | 0/3 |
| Gastrinoma/insulinoma | 2 | 2/2 | 1/1 | 1/1 |
| Lymphoma (ulceration and hemorrhage) | 2 | 1/2 | 0/2 | 0/2 |
| Melanoma | 2 | 1/2 | 1/2 | 1/2 |
| Leiomyosarcoma | 1 | 0/1 | 0/1 | 0/1 |
| TOTAL | 27 | 21/27 (78%) | 14/23 (61%) | 5/19 (26%) |
| **Survival Rate (Benign lesions)** | | | | |
| Cystadenoma | 1 | 1/1 | 0/0 | 0/0 |
| Localized pancreatitis (susp. cancer) | 3 | 3/3 | 3/3 | 1/1 |

These observations confirm that pancreticoduodenectomy can be performed with acceptable morbidity and therapeutic benefit for the patient with a periampullary tumor other than ductal pancreatic carcinoma.

References
Shiu MH. Surg Gynecol Obstet 1982 154: 497.
Shiu MH. Proc. of 1st World Congress H-P-B Surgery, Lund, 1986, Abst. 59.

FP247 RESECTION OF MICROGASTRINOMAS WITH METASTATIC LYMPHNODES

M. Imamura, Y. Hattori, H. Shimada,
J. Tanaka, S. Arii, T. Tobe
Kyoto University, Kyoto, Japan

It is often difficult to locate gastrinomas preoperatively, and in 30% of patients with Zollinger-Ellison syndrome (ZE) a gastrinoma (Goma) has not been found even by surgery (Zollinger 1985). We developed a technique which is useful for locating microgastrinomas (Selective Arterial Secretin Injection test ; SASI test : Imamura 1987) and the intraoperative secretin test with rapid radioimmunoassay of serum gastrin (IOS test : Imamura 1989) for estimating the extent of the resection of Goma.

In 13 patients with ZE, SASI test located Goma in each patients. Based on the results of SASI test, radical resection of microGoma was performed in 5 patients successfully (4 Whipple and one extirpation with lymphnodes dissection). Rate of true positive results was 100% in SASItest, though that of other diagnostic imaging techniques was less than 50%, and rate of either false-positive or false-negative rate of SASI test was 0%. IOS test was useful in estimating radicality of the operation during surgery. Pathological study revealed 22 Gomas in the pancreatoduodenal region of the 5 patients, although imaging techniques visualized only two tumors preoperatively. All of the patients had more than one microGoma and four of them had more than one metastatic lymphnode. All of the patients have been alive without any sign of recurrence for between 3 months and 3 years and half.

References:

Zollinger RM. Surgery 1985:97:49
Imamura M et al. Ann Surg 1987:205:230
Imamura M et al. Ann Surg 1989:210:711

FP248

### TOTAL PANCREATECTOMY IN DUCTAL ADENOCARCINOMA OF THE PANCREAS

B. LAUNOIS, J.M. FRANCI, N. KUNIN,

M.P. RAMEE, Y. MALLEDANT et J.P. CAMPION

Clinique Chirurgicale - C.H.U. de RENNES

FRANCE

We conclude that total pancreatectomy has increased the resectability rate, mainly in patients where the tumour has spread across the usual section of Whipple's procedure. It is useless when portal vein resection is necessary or multicentric cancer or neoplastic emboli are observed in the operative specimen.

(1) TRYKA A.R. and BROOKS ,
Histopathology in the evaluation of total pancreatectomy for ductal carcinoma
Ann. Surg. 1979, 190 : 373-381

FP249 ROLE OF INTRAOPERATIVE ULTRASONOGRAPHY IN DETECTING LIVER METASTASES FROM COLORECTAL CANCER.

A.K.Olsen, Central Hospital of Rogaland, Stavanger, Norway.

During the period of study, february 1987 - february 1989, 213 patients were admitted for elective surgery for colorectal cancer.

The examination consisting of preoperative ultrasonography associated with inspection and palpation of the liver during surgery was compared with intraoperative ultrasonography of the liver.

In 42 patients (19.7%) 238 metastatic tumours were found by preoperative ultrasonography, inspection and palpation during surgery. Using intraoperative ultrasonography 57 additional metastatic lesions were found in 19 of these patients. In 21 patients preoperative ultrasonography, inspection and palpation during surgery failed to detect small metastatic tumours. In these patients 59 tumours were diagnosed by intraoperative ultrasonography ($p<0.003$).

We conclude that detailed information regarding number, size and location of the metastatic lesions is provided by intraoperative ultrasonography and advocate the routine inclusion of this form of examination of the liver during surgery for cancer of the colon and the rectum.

FP250 REGIONAL CHEMOTHERAPY OF LIVER METASTASIS OF COLORECTAL CARCINOMA. RESULTS OF A PROSPECTIVE RANDOMISED STUDY

F. Safi, R. Roscher, H. G. Beger
University of Ulm, Dept of General Surgery, FRG

From 1982 to 1989 273 patients with hepatic metastasis of colorectal carcinoma were admitted to our clinic. 159 patients did not receive treatment: 69 patients with intra- and extra-hepatic metastasis (group I), 90 patients with isolated hepatic metastasis (group II). A further 88 patients were treated regionally (group III) and the remaining 26 patients underwent surgical resection of the metastasis (group IV). Since patients died under intra-arterial chemotherapy of extrahepatic spread of the metastasis, we started a randomised controlled trial to compare the efficacy of intra-arterial to continuous simultaneous intra-arterial and intravenous chemotherapy of liver metastasis. The first 20 patients of group III were treated only intra-arterially (pilot group). The other 68 patients of this group were stratified by primary tumor stage and the percent of liver involvement and were then randomly assigned before surgery to receive either intra-arterial (IA group n = 32 pat.) or intra-arterial and intravenous therapy (IA/IV group n = 36 pat.). Intervention: Fourteen-day continuous infusion of FUDR each month (0.2 mg/kg/day in all intra-arterially treated patients and 0.3 mg/kg/day in the intra-arterial and intravenous group). Results: The complete and partial response rate was 60%, 64% and 50% in the IA, IA/IV and pilot group respectively. 72% of the IA group, 75% of the pilot group and 28% of the IA/IV group developed extra-hepatic disease in a median follow-up time of 18 months (p 0.01). Hepatic and systemic toxicity in the IZ and IA/IV group were acceptable. The median survival time of group I was 6 months (range 1-44), of group II 11 months (range 1-57), of group III 24 months (range 3-59) and of group IV 28 months (range 6-66). No significant difference in survival was found between the IA and IA/IV groups. Depending on our response criteria we divided the 88 patients into two groups: responder and non-responder. The median survival time of responders was 30 months, of non-responders 50 months (p 0.001). These results show for the first time that the combination of continuous simultaneous intra-arterial and intra-venous therapy decreases the occurrence rate of extrahepatic meta-static spread in patients with isolated liver metastasis significantly. The survival time of patients treated with FUDR-therapy, especially in those who responded to the therapy, is significantly prolonged in comparison with patients who did not receive any treatment.

FP251 INTRA-ARTERIAL TREATMENT OF HEPATIC METASTASES.

J. González, J. A.-Cienfuegos, F. Pardo, C. Benito, V. de Villa, G. Zornoza, I. Bilbao, O. Fernández.
Departments of Surgery, Radiology and Oncology. Clínica Universitaria de Navarra. Pamplona. Spain.

We have treated 27 patients with unresectable hepatic metastases from colo-rectal carcinoma through hepatic artery infusion of carboplatin(CBDCA) and 5-fluorouracil (5-FU). The hepatic artery access was made by surgical implanted device (Implantofix) or through transfemoral catheter (Seldinger technique).
The treatment consisted in the infusion of 55 mg/m2 of CBDCA during two hours and 900 mg/m2 of 5-FU the remaining twentytwo hours, during five consecutive days each five weeks.
Patients age ranged from 34 to 80 years (m-59). 17 were male and 10 female. Liver involvment measured by computed tomography (CT) was lesser than 25% in 14 patients, between 25 and 50% in three patients, between 50-75% in 7 patients and greater than 75% in three patients. The medium of cycles was 7 (2-18).
With a medium follow-up longer than 16 months we have had (according with world health organization criterions):

- Objetive response in 11 patients (40%) with a medium duration of 17 months (3-24.5).
- Complete remission in 2 patients (follow-up of 6 and 11 months after finishing the treatment).
- In three cases the disease progressed.
- In the other eleven patients we have had no changes in their disease or a decrease in the tumor size lesser than 50%.

The medium survival in 16 months (3-30). The toxicity consisted in nausea and vomiting grade I-II in ten patients. None case of hepatitis, duodenitis, colangitis or cholecystitis has been reported. The complications due to the device were three infections and four obstructions.

Conclusion

Intra-arterial infusion in five days of CBDA and 5-FU is effective in the treatment of hepatic metastases from colorectal carcinoma.

### FP252 INTRAARTERIAL CHEMOTHERAPY FOR METASTATIC LIVER CANCER USING MULTIPLE ANTICANCER AGENTS SUSPENDED IN LIPIODOL

H. Taniguchi, A. Oguro, A. Itoh, T. Daidoh, N. Tsukuda, Y. Shioaki, K. Sawai, T. Yamaguchi, T. Takahashi

First Department of Surgery, Kyoto Prefectural University of Medicine, Kyoto, Japan

For the purpose of enhancing effect of intra-arterial infusion chemotherapy using anticancer agents suspended in Lipiodol, which is a lipid contrast medium, the combined cancer chemotherapy was applied to this therapy.

Fifty-four patients with unresectable metastatic liver tumors were treated. Thirteen patients were given doxorubicin (ADR) suspended in Lipiodol (ADR-LIP) or stylene maleic acid neocarzinostatin dissolving in Lipiodol (SMANCS/LPD) and infused into the hepatic artery in the one-drug suspension group, 11 patients received ADR and mitomycin-C (MMC) suspended in Lipiodol (AM-LIP) in the two-drug suspension group, and 30 patients were treated with 5-Fluorouracil or carmofur, ADR and MMC suspended in Lipiodol (FAM-LIP) in the three-drug suspension group. The response to the treatment was evaluated by the total tumor area on computed tomograms showing the largest tumor area, serum carcinoembryonic antigen (CEA) level, and survival after the treatment.

A reduction in tumor area was seen in 30.8% (4/13), 60.0% (3/5), and 73.3% (11/15) in one-, two-, and three-drug suspension group, respectively; decreases in CEA were noted in 20.0% (2/10), 60.0% (3/5), and 85.7% (18/21), respectively; and median survival was 102, 245, and 333 days, respectively.

It was concluded that the infusion of a combination of anticancer agents suspended in Lipiodol is effective in controlling metastatic liver cancers.

### FP253 A COMPARISON OF ARTERIALLY ADMINISTERED ADRIAMYCIN-LOADED ALBUMIN MICROSPHERES AND DRUG IN SOLUTION

J.A. Goldberg, D.J. Kerr, N. Willmott, C.S. McArdle.
Royal Infirmary & University of Strathclyde, Glasgow, Scotland, U.K.

The poor results of systemic chemotherapy for colorectal liver metastases has stimulated interest in regional chemotherapy. By loading anti-cancer agents into biodegradable particles which become trapped in the capillary bed following hepatic arterial injection, it may be possible to further increase tumour drug exposure, and hence anti-tumour effect, without increasing systemic toxicity (1). The aim of this study was to compare the anti-tumour effect of adriamycin-loaded albumin microspheres (ALAM) with that of the drug in solution.

Sprague-Dawley rats (mean weight 600 g) bearing three day old hepatic implants of Walker tumour were divided into 4 treatment groups (n=6). The animals were culled four days later, and the tumours excised, weighed blind and submitted for histology.

| **Group** | | **Tumour weight (g) mean ($\pm$sd)** |
|---|---|---|
| I | Sham operated control | 0.73$\pm$ 0.21 |
| II | Operated + IA "empty" microspheres (2 mg) | 1.01$\pm$ 0.27 |
| III | Operated + IA ADR (30 ug Adriamycin) | 0.74$\pm$ 0.12 |
| IV | Operated + IA ALAM (2 mg) containing 30 ug Adriamycin | 0.45$\pm$ 0.08 |

Tumours in the ALAM group (IV) were significantly smaller than tumours in group I, II and III ($p < 0.05$, Mann-Whitney test). This study suggests that adriamycin-loaded albumin microspheres are more potent than drug in solution.

I. Kerr DJ, Willmot N, Lewi H, and McArdle CS. The pharmacokinetics and distribution of adriamycin-loaded albumin microspheres following intra-arterial administration. Cancer 1988; 62: 878-882.

**FP254** INTERVENTION DIRECTED AT THE LIVER IN PATIENTS WITH MID-GUT CARCINOID TUMOURS - WHAT CAN BE ACHIEVED ?

O. Søreide, A. Bergan, A. Bakka, T. Berstad, A. Flatmark, L. E. Hanssen, E. Schrumpf, Surg. Dept. B and Med. Dept. A, National Hospital, Oslo, Norway

The role of surgery in patients with carcinoid tumours is not well defined. Two aspects must be considered: 1. What can be achieved in technical terms; and 2. The potential benefits in terms of symptomatic relief and increased survival. We report the results of an aggressive surgical policy in 65 pts. with mid-cut carcinoid tumour. Forty (62%) were symptomatic and in 23 (38%) the diagnosis was as chance finding. In 23 (38%) was a primary gut tumour diagnosed by preoperative imaging. 18 pts. (28%) had a classic carcinoid syndrom of whom 17 (84%) had liver metasteses, 5 (28%) had carcinomatosis and 7 (39%) ascites. Lymph node metastases were found in 82%, and 80% had distant spread. The overall frequency of liver metastases was 75%, of which 86% had diffuse bilateral spread and only 9% more well defined metastatic deposits. In 53 (81%) resection of the primary intestinal tumour was performed, 8 (12%) underwent abdominal exploration only. Intervention directed at the liver was initially carried out in 20 pts. (32%) (1 metastasectomy, 8 hepatic artery ligation, 11 embolisation) and during subsequent reoperations in 13 pts. (20%) (1 metastasectomy, 3 tumour enucleation, 8 hepatic artery ligation and 1 embolisation). The complication rate of the surgery directed at the liver was 33% (3/9) following the initial operation, 25% (2/8) after the first and 25% (1/4) after the second reoperation. Complication was noted in 18% (2/11) of patients undergoing embolisation. Survival data is currently under analysis. In conclusion, most patients with carcinoid tumours have advanced disease when diagnosed. Treatment directed at the liver can be offered to nearly half of the patients.

### FP255 VALUE OF OPERATIVE PANCREATOGRAPHY IN SURGICAL DECISION MAKING

LA Desa, RCN Williamson
Royal Postgraduate Medical School
Hammersmith Hospital London. UK

Full pancreatic ductography is essential for the proper diagnosis and surgical assessment of chronic pancreatitis and certain other pancreatic diseases, but endoscopic pancreatography (ERP) is sometimes unobtainable or inadequate. We have performed 115 operative pancreatograms during 109 operative procedures on 104 patients. Diagnoses were chronic pancreatitis (n=59), pancreas divisum (14), recurrent acute pancreatitis (11), acute pancreatitis with pseudocyst (6), pancreatic cancer (5), cholelithiasis (3) and unexplained abdominal pain (18). ERCP was performed on 77 occasions, with a successful cannulation rate of 88%, though the pancreatic duct was visualised in only 77% of cases.

Operative pancreatography was performed by various routes:

1. Retrograde from the major papilla (n=31) or minor papilla (4), or from the pancreatic neck after proximal resection (11).
2. Prograde after distal resection (29).
3. Ambigrade by needling the duct (18).
4. Cystography into a pseudocyst (21).
5. Intestinal loopography after a previous pancreatojejunostomy (1).

There was a failure rate of 4% and one false positive result. Marked discrepancy between the findings at ERP and operative pancreatography resulted in a change of plan in 32 patients. Operative pancreatography was invaluable when ERP was unsuccessful (failed cannulation or cholangiogram only, n=18) incomplete ('obstruction' preventing visualisation of the upstream duct, n=17) or not attempted (n=31). Operative pancreatography is technically easy, free from complications and very helpful in planning operative strategy, notably the choice between resection or drainage in chronic pancreatitis.

**FP256** THE USE OF ULTRASOUND DISSECTION IN PANCREATIC SURGERY

Mr. Hugh Thomas, Consultant Surgeon,
Prince Charles Hospital, Merthyr Tydfil.

The use of ultrasound to dissect away cellular tissue, leaving arteries with their blood supply intact, is a well known technical adjuvant in liver dissection. In the last few years this technique has been utlised in studying the arterial blood supply of the duodenum and pancreas. The blood supply of the duodenum and common bile duct is shown to be mainly dependent on the arcade of vessels emanating from the posterior branch of the gastro duodenal artery. Operative occlusion of other vessels supplying the duodenum did not alter the capacity of the duodenum to survive and remain pink.

By posterior surgical exposure and preservation, of this blood supply it has been possible to perform several duodenum saving pancreatectomies with minimal blood loss. Awareness of the presence and importance of this arterial arcade should popularise this operation. Conversely, the blood supply from this arcade of vessels to the duodenum can be divided and the duodenum itself removed leaving a rim of duodenal wall attached to the ampulla. This is readily implanted in jejunum advanced to take the place of the excised duodenum. This new procedure, not known to have been previously described, has been performed in several cases of duodenal cancer.

Acknowledgements

Dr. Bernard Charnley, Consultant Pathologist, Prince Charles Hosp.
Dr. H. El. Shaboury, Consultant Physician, Prince Charles Hospital
Mr. Jago, Sonning Med.
Mr. Martin Hill, Codmans, U.K.

FP257 THE FASCIAL ATTACHMENTS OF THE HEAD OF THE PANCREAS AS ENCOUNTERED IN THE WHIPPLE PROCEDURE

M.C.Brett, C.R.Mackie

Department of Surgery,
University of Liverpool, U.K.

In pancreatoduodenectomy, pancreatic mobilization is completed by dividing the fascial sheet attaching the uncinate process to the aorta and superior mesenteric artery. The surgical anatomy of this procedure is poorly described. Blood vessels traversing the fascia require division while others must be preserved. In a series of cadaveric dissections (fifteen to date) we identified this fascia, after mobilization of the portal/superior mesenteric vein. Traction on the pancreatic head showed it to be a strong layer fanning out from the uncinate process. Superiorly it is firmly attached to the coeliac axis and superior mesenteric artery origin; inferiorly it divides into two leaves, the anterior attached to the superior mesenteric artery in front of the duodenum, the posterior adherent to the aorta behind the duodenum. The uncinate process and superior mesenteric artery are separated by a gap of 1-2cm. A fatty layer between the two leaves was variably traversed from above downwards by an aberrant right hepatic artery, an uncinate branch from the transverse pancreatic artery, inferior pancreatico-duodenal arteries and upper jejunal arteries. Inferior pancreatico-duodenal arteries originated from aberrant right hepatic or jejunal arteries in five cases. Pulling the fascia taut also rotated the superior mesenteric/portal vein. In four cases the first jejunal venous tributary was exposed, two of these receiving pancreatico-duodenal veins. Jejunal and hepatic vessels are vulnerable during resection of the pancreatic head and a more precise knowledge of the surgical anatomy should enable the procedure to be carried out more safely.

### FP258 SPLENIC PRESERVATION DURING DISTAL PANCREATECTOMY FOR PANCREATIC DISEASE

M C ALDRIDGE, M J COOPER, R C N WILLIAMSON

Departments of Surgery, Royal Postgraduate Medical School and Royal Devon and Exeter Hospital.

The intimate relationship of the splenic artery and vein to the body of the pancreas makes splenectomy a routine part of distal pancreatectomy. Yet, splenic conservation is both desirable for immunological reasons and sometimes feasible. A personal series of 91 distal pancreatectomies undertaken for pancreatic disease between 1978-89 included 33 women and 58 men with a median age of 43 yr (range 17-78 yr). Conventional resection including splenectomy was performed in 58 patients for chronic pancreatitis (n=46), pancreatic carcinoma (n=7) or other neoplasms (n=5). Conservative resection with splenic preservation was performed in 33 patients (36%) for chronic pancreatitis (n=12), suspected pancreatitis (n=11, including 8 with pancreas divisum), neoplasia (n=5), recurrent acute pancreatitis (n=2), pancreatic trauma (n=2) or pancreatic duct stricture (n=1). Distal resection was a part of total pancreatectomy in 22 patients (chronic pancreatitis 15, cancer 7), including 4 reoperated for the postoperative complications of proximal pancreatectomy; there were 6 deaths in this group. Among the 69 patients undergoing distal resection only (conventional 40, conservative 29) there were no postoperative deaths. Complications of these conventional resections were reactive haemorrhage (3), gastrointestinal fistula (2) and a peripancreatic collection requiring percutaneous drainage (3). Complications of the conservative approach were adhesion obstruction (2), collection (1) and delayed wound infection (1); the splenic vessels were ligated (away from the splenic hilum) in 5 patients, but subsequent isotope scans and haematological indices did not indicate hyposplenism.

The spleen can safely be preserved in many distal pancreatectomies even for inflammatory disease.

FP259

### MEDIAL PANCREATECTOMY FOR BENIGN TUMORS. A REPORT OF 13 CASES.

N. Rotman, B. Sastre, D. Cherqui,
M. Kracht, JL. Sarles, PL. Fagniez
Hôpital Henri Mondor 94000 Créteil, France
Hôpital Ste Marguerite 13000 Marseille, France

The surgical treatment of benign tumors of the pancreas until now has consisted of either enucleation or formal pancreatectomy. Nonetheless, for tumors of the neck of the pancreas enucleation is not always feasible and extended pancreatectomies carry a risk of exocrine and endocrine pancreatic insufficiency. For these reasons we proposed a limited pancreatectomy consisting of a resection centered on neck of the gland with complete excision of the tumor. The cephalic section was sutured and a Roux-en-Y loop was anastomosed to the distal section of the pancreas. Thirteen patients were operated on by this technique : 7 cystadenomas, 4 endocrine tumors, 1 pseudo inflammatory tumor and 1 necrotic pseudocyst. No patient died. All patients have been followed up from 4 to 84 months. No patient developed diabetes mellitus. One patient had a postoperative pancreatitis which resulted in an exocrine insufficiency. One patient developed a pancreatic fistula which healed spontaneously and another patient was reoperated for an abscess at the site of pancreatico-jejunal anastomosis.
We conclude that limited pancreatectomy does not carry a higher operative risk than formal pancreatectomy and results in minimal impairment of exocrine and endocrine function.

**FP260** INTRAOPERATIVE SONOGRAPHY: NO MORE PROBLEMS IN LOCALIZING OCCULT INSULINOMAS ?.

C. Pasquali, C. Sperti A. Alfano D'Andrea, G. Liessi*, G. Pesce^,S. Pedrazzoli.
Clinica Chirurgica 1 ,Univ. of Padua, Surgery^ & Radiology* Castelfranco, I

Recently intraoperative sonography (I.S.) has shown to be helpful in localizing endocrine tumors not found at surgery. Since 1966 we observed 52 cases of organic hyperinsulinism and in 33 % of cases the tumor could be not felt by the surgeon. In 1983 after introduction of I. S., 16 patients were examined using this technique. Two of them had a previous uneffective surgical exploration. 4 of them had occult insulinomas that in 2 cases were associated to palpable adenomas. 2 patients had a nesidioblastosis.
The result of I. S. was correct in 15 cases with 13 true positive and 2 true negative exams. In 1 patient with nesidioblastosis we had a false positive. Tumors with a diameter < 0.3 cm. (2 cases) were not detect by I. S.
In the same group of patients we performed 8 transhepatic portal samplings (TPS) that correctly localized the tumor in 7 cases. CT-scan was correct in 8/13 cases and preoperative echo-scan was correct in 5/11 cases. All 16 patients underwent angiography; 5 insulinomas were correctly localized by this technique. NMR was performed in 6 patients with 3 positive results. All tumors found by using I. S. were also localized by one at least of the other imaging techniques. Nontheless, I. S. was able to detect 2 adenomas which could not be found with the surgical exploration of the pancreas. A patient with focal beta-cells hyperplasia localized in the body of pancreas, TPS was the only diagnostic technique which supported a useful information for surgical treatment.
I. S. can be considered a useful method to localize insulinomas, showing a sensitivity which was, in our series, higer than any preoperative imaging technique. Unfortunately, microadenomas and microscopic diseases of the endocrine cells, which occurred in 4/16 of our patients, can not be predicted by preoperative imaging investigations or by I. S.. In these cases TPS is the only way to get information about a diffuse or focal disease.

**FP261** IS LOCAL ISCHEMIA ONE OF THE TRIGGER MECHANISMS OF ACUTE PANCREATITIS?

[1]U. T. Hopt, [1]M. Büsing, [1]H. D. Becker, [2]K. Morgenroth

[1]Department of Surgery, University of Tübingen, FRG

[2]Institute of Pathology, University of Bochum, FRG

The initial events, which trigger the onset of acute pancreatitis are still a matter of debate. In animal experiments a number of procedures may induce acute pancreatitis. The significance of local ischemia, however, is still controversial. Pancreatic transplantation provides for the first time the possibility to clarify in man the effect of ischemia. In a consecutive series of 27 pancreatic transplantations a significant edema of the graft accompanied by a transient rise of pancreatic enzymes in serum were seen. In 5 of the patients ischemic damage resulted in a typical necrotising pancreatitis. In order to clarify the sequence of morphologic events of this ischemically induced damage, serial biopsies of 6 pancreatic allografts were taken at the end of cold ischemia as well as 30, 90 and 300 minutes after reperfusion. The biopsies were studied with semithin section microscopy, scanning and transmission electronmicroscopy. By light microscopy a more or less pronounced vacuolisation of single acinar cells was most striking at the end of cold ischemia. These vacuoles result from a striking dilatation of endoplasmic reticulum and from condensing vacuoles. Reperfusion of the graft induces a dramatic change in the morphological picture. Very soon strong perivascular infiltrates of leucocytes can be found. In addition, autophagolysosomes and single cell necrosis can be demonstrated. Because of defects in the basal membranes zymogen granulae appear in the intercellular fluid. The morphologic findings observed seem to correlate with the extent of the ischemic damage of the graft. Thus, ischemia and reperfusion of pancreatic allografts induces reproducable morphological changes, which resemble those found in different models of experimental pancreatitis. Local ischemia therefore must be regarded as one of the trigger mechanisms for development of acute pancreatitis in man.

**FP262 THE CONCENTRATION OF DIFFERENT BACTERICIDAL ANTIBIOTICS IN HUMAN PANCREATIC TISSUE.**

H.Frieß, M.Büchler, R.Isenmann, H.G.Beger
Depart. of Surgery, University of Ulm, FRG

In acute necrotizing pancreatitis bacterial contamination of pancreatic necrosis is the main prognostic factor and septic complications represent the most frequent cause of death.

Mainly gram-negative enteric germs are found in infected pancreatic necrosis (1). Recently it has been shown that mezlocillin and metronidazole concentrate sufficiently in the human pancreas (2).

In a further analysis we investigated the human pancreatic concentration of 8 antibiotics covering the bacterial spectrum of pancreatic infection.

Patients: Samples of pancreatic tissue, juice and cyst fluid were collected from 101 patients (78 male,23 female) undergoing surgery for chronic pancreatitis ( n=64), necrotizing pancreatitis (n=14), and pancreatic cancer (n=23). 30 min prior to laparotomy one of the following drugs was given i.v.: Piperacillin 4g (PIP), Cefotaxime 2g (CTX), Ceftixozime 2g (CFI), Netilmicin 150 mg (NET), Tobramycin 80 mg (TOB), Imipenem/Cilastin 500 mg (IMI), Ofloxacin 200 mg (OFX), Ciprofloxacin 200 mg (CIP).

Methods: The concentration of antibiotics in pancreatic tissue, juice, cyst fluid and in the corresponding serum samples was determined by HPLC.

The efficacy of the antibiotics was calculated by an efficacy factor containing the percentage and type of bacteria found in pancreatic infection, the median pancreatic tissue concentration (120 min) of the antibiotic drug and the respective minimal inhibitory concentration.

Results: Median pancreatic tissue concentrations, serum to tissue ratios and efficacy factors are shown in the following table:

| | NET | TOB | PIP | CFI | CTX | CIP | OFX | IMI |
|---|---|---|---|---|---|---|---|---|
| $C_{tissue}$(mg/kg) | 0.4 | 0.5 | 37.1 | 7.3 | 8.4 | 0.9 | 1.4 | 4.3 |
| $C_{serum}/C_{tissue}$ | 0.14 | 0.12 | 0.51 | 0.32 | 0.33 | 1.00 | 0.86 | 0.34 |
| efficacy factor* | 15.2 | 26.4 | 65.7 | 66.1 | 76.0 | 79.5 | 88.0 | 89.9 |

* 100,0 would be optimal

Conclusion: Antibiotic treatment, which is mandatory in necrotizing pancreatitis, should be based on tissue penetration results and the break points for corresponding bacteria. Aminoglycosides do not enter the human pancreas in sufficient concentrations. According to the calculated efficacy factor, Imipenem, Ofloxacin, Ciprofloxacin or Cefotaxim are the antibiotics, which should be used in patients with infected pancreatic necrosis.

References:

1) Beger H.G. et al, Gastroenterology 1986; 91: 433
2) Büchler M. et al, Infection 1989; 17: 20

### FP263 NEUROIMMUNE CHANGES IN CHRONIC PANCREATITIS: A NEW PAIN CONCEPT

H. Frieß, M. Büchler, E. Weihe, H.G. Beger

Depart. of Surgery, University of Ulm, FRG;

Depart.of Anatomy, University of Mainz, FRG

Pain is a major factor in chronic pancreatitis. By electron microscopy specific morphological alterations and changes in pancreatic nerves have been demonstrated in patients with chronic pancreatitis. (1). In a further analysis we were interested at neurotransmitters present in pancreatic nerves and the interrelationship between nerves and immune cells in chronic pancreatitis.

Patients: Pancreatic tissue (head and body) from 20 patients with chronic alcohol-induced pancreatitis (16 male, 4 female, mean age 42 years) was compared with that of 10 organ donors (8 male, 2 female, mean age 43 years).

Methods: Specimens were processed for routine light microscopic immunohistochemistry using antibodies against VIP and PHI (parasympathetic neurotransmitters),NPY and TH ( sympathetic) and Substance P and CGRP (sensory= pain transmitters), respectively. Specific antisera against pan-leucocytes, macrophages, B-lymphocytes and T-lymphocytes were used for inflammatoy cell analysis.

Special attention was paid to the inflammatory reaction adjacent to varicose nerve fibers.

Results: In chronic pancreatitis exactly those nerves which were found to be enlarged and ultrastructurally altered (destruction of the perineurial sheath) showed a striking increase of immunoreactivity for Substance P and CGRP. The neurotransmitter pattern of parasympathetic and sympathetic efferents was unchanged. Characteristically primary sensory afferents (immunoreactive for Substance P and CGRP) were surrounded by lymphocytes, macrophages and mast cells.

Conclusion: The particular increase of fibers stained for CGRP and Substance P, which are both known as pro-nociceptive and pro-inflammatory agents, may contribute to the generation and continuation of the pain syndrome.The neuroimmune cross-talk in chronic pancreatitis opens up a new concept of pathogenesis comparable with changes in other painful chronic inflammatory conditions.

References:

1) Bockman D. et al, Gastroenterology 1988: 94: 1459

### FP264 CLINICOPATHOLOGICAL STUDY OF THE PANCREATIC CANCER, PARTICULARLY NEURAL INVASION IN THE EXTRAPANCREATIC PLEXUS

M Kayahara, T Nagakawa, N Kadoya, H Kobayashi, Y Nakano, T Nakamura, K Maeda, N Ueda, T ohta, K Ueno, I Konishi, T Matsumoto, I Miyazaki

Department of Surgery II, School of Medicine, Kanazawa University, Kanazawa, Japan

The concept of neural invasion is widely accepted in the reference to pancreatic cancer. Our group pointed out that the retroperitoneal invasion, including neural invasion, was extended to the surrounding of the superior mesenteric artery. We have emphasized the radical retroperitoneal dissection is needed for the treatment of the pancreatic cancer. This report describes the clinicopathological significance of the neural invasion for the treatment of the pancreatic cancer.

This investigation was based upon 34 cases of the ductal carcinoma of the pancreas head. Resected specimens were serially cut 5mm in thickness. Pathological findings were defined according to the General Rules for Cancer of the Pancreas which was published by Japan Pancreas Society. To further investigate the neural invasion, serial sections 5micron in thickness were cut from four cases which had retroperitoneal infiltration (rpe) and free retroperitoneal surgical margin. Total of 1774 sections were evaluated.

Rpe occurred 29 (85%) of the 34 cases. Extrapancreatic plexus invasion revealed 21(72%) of the 29 rpe cases. The tumor size had no relation to the retroperitoneal infiltration and extrapancreatic plexus invasion. The localizations of extrapancreatic plexus invasion were as follows. Hepatoduodenal ligament plexus in 2 patients, pancreatic capitalis I plexus (Plx.pc-I) in 2, pancreatic capitalis II plexus (Plx.pc-II) in eleven , Plx.pc-I+II in 3, and other position in 3 patients.

The study of the serial sections resulted that the manner of the neural invasion was serial spread within perineural space, and at the branching point of the nerve, tumor cells within the nerve spread along the nerve branch.The carcinoma cells around the nerves infiltrated into the perineural space via a plane of least resistance.

Retroperitoneal invasion including extrapancreatic neural invasion was highly observed for the carcinoma of the pancreas, and the manner of the neural invasion was essentially serial spread. We conclude that radical retroperitoneal dissection is most important for the treatment of the pancreatic cancer.

### FP265 PANCREATOBILIARY DIVERSION AND ENTERECTOMY: DIFFERENTIAL EFFECTS ON PANCREATIC HYPERPLASIA AND NEOPLASIA

P. Watanapa, ID. Stewart, B.Flaks, PW.Davies,
RCN. Williamson

Hyperplasia and neoplasia have an established association in several gastrointestinal organs. Possible promotional effects of pancreatobiliary diversion (PBD) and 90% proximal small bowel resection (PSBR) were studied in a rat model of pancreatic carcinogenesis, induced by azaserine. The number of atypical acinar cell foci (AACF) was estimated, this being the putative premalignant lesion. Male Wistar rats, weighing 250-300g, were divided into 3 groups. Group A (n=18) underwent triple transection and reanastomosis of the small bowel (controls). Group B (n=11) underwent PSBR and group C (n=16) underwent PBD. Postoperatively, all animals received azaserine (20mg/kg/wk ip.) for 6 weeks and were killed at 6 months. Median pancreatic weight (mg pancreas/g body weight) was 2.2 for controls, 4.08 for PSBR ($p<0.001$) and 6.86 for PBD ($p<0.001$). The number and size of AACF in tissue sections were determined and mathematically transformed into three-dimensional data. PBD produced a marked increase in the number of acidophilic AACF per $cm^3$ (median 96 vs.0;$p<0.001$) and a 7-fold increase in their volume ($p<0.001$), but no such response was elicited by PSBR.

Both PBD and PSBR stimulate pancreatic growth but only PBD enhances tumour development in this rat model.

FP266 STUDIES ON THE HEMODYNAMICS OF THE PANCREAS IN THE CONSCIOUS STATE: INFLUENCE OF TRUNCAL VAGOTOMY

K. Takaori, K. Inoue, S. Sumi, R. Doi, M. Yun, S. Higashide, S. Minote, Y-J. Gu, M. Maung, T. Aung, K. Uchida*, T. Tobe
First Department of Surgery, Faculty of Medicine, *College of Medical Technology, Kyoto University
Kyoto, Japan

Changes of Pancreatic Blood Flow(PBF) in response to food, sham feeding and truncal vagotomy were observed in conscious dogs to elucidate as to if neural mechanisms are involved in the regulation of PBF. METHODS: PBF in conscious dogs were measured continuously by the thermoelectric method with a thin thermocouple implanted into the pancreatic tissue. After an overnight fast, food(meat meal, 40g/kg) was given to six normal dogs(1), seven dogs constructed with external esophagostomy as sham feeding(2) and four dogs with truncal vagotomy(3). RESULTS: (1) After food stimulation, PBF increased to attain the peak value of 65.2±6.2% above the basal flow and the significant elevation of PBF above the basal flow remained until 120 minutes approximately. (2) After sham feeding, PBF increased to show the peak rate of increase of 89.0±19.0%. In contrast to the normal feeding, however, PBF decreased rapidly without presenting the second phase of PBF response. (3) Although PBF showed still a significant increase even after truncal vagotomy, the peak rate of increase(28.2±7.1%) was significantly less than that observed after normal or sham feeding. CONCLUSION: While normal feeding causes biphasic response in PBF, only the initial phase of PBF response is observed after sham feeding. These facts suggest that the initial response is mainly controlled by the cephalic phase. The initial response of PBF was decreased but still elevated significantly above the basal value even after truncal vagotomy. It indicates that the cephalic phase is mediated not only through the vagal nerve but also through other pathways.

### FP267 BILILINGUS OR CHOLEGLOSSIA? - TOWARDS A BILIARY ESPERANTO

D. S. Johnson, Westmead Hospital,
Westmead, N.S.W. Australia

A brief history of biliary terminology from its origins will be traced. In particular the nomenclature arising from the rapid development of biliary surgery in the late 19th and early 20th centuries is examined, and the new terminology of that 20th century technology which has come to modify biliary surgical practices is discussed.

As the eastern world begins to encroach both economically and technologically on the western world one should expect new directions in the language of biliary surgery.

Perhaps the time is approaching for a basic Biliary Glossary to be written to assist non-English speaking participants at congresses such as the HPB to communicate. Or would this only be regressing to George Orwell's 1984?

